# CREST - a large and diverse superfamily of putative transmembrane hydrolases

**DOI:** 10.1186/1745-6150-6-37

**Published:** 2011-07-06

**Authors:** Jimin Pei, Douglas P Millay, Eric N Olson, Nick V Grishin

**Affiliations:** 1 Howard Hughes Medical Institute, University of Texas Southwestern Medical Center, 6001 Forest Park Road, Dallas, Texas, 75390, USA; 2 Department of Molecular Biology, University of Texas Southwestern Medical Center, 6001 Forest Park Road, Dallas, Texas, 75390, USA; 3 Department of Biochemistry, University of Texas Southwestern Medical Center, 6001 Forest Park Road, Dallas, Texas, 75390, USA

## Abstract

**Background:**

A number of membrane-spanning proteins possess enzymatic activity and catalyze important reactions involving proteins, lipids or other substrates located within or near lipid bilayers. Alkaline ceramidases are seven-transmembrane proteins that hydrolyze the amide bond in ceramide to form sphingosine. Recently, a group of putative transmembrane receptors called progestin and adipoQ receptors (PAQRs) were found to be distantly related to alkaline ceramidases, raising the possibility that they may also function as membrane enzymes.

**Results:**

Using sensitive similarity search methods, we identified statistically significant sequence similarities among several transmembrane protein families including alkaline ceramidases and PAQRs. They were unified into a large and diverse superfamily of putative membrane-bound hydrolases called CREST (alkaline ceramidase, PAQR receptor, Per1, SID-1 and TMEM8). The CREST superfamily embraces a plethora of cellular functions and biochemical activities, including putative lipid-modifying enzymes such as ceramidases and the Per1 family of putative phospholipases involved in lipid remodeling of GPI-anchored proteins, putative hormone receptors, bacterial hemolysins, the TMEM8 family of putative tumor suppressors, and the SID-1 family of putative double-stranded RNA transporters involved in RNA interference. Extensive similarity searches and clustering analysis also revealed several groups of proteins with unknown function in the CREST superfamily. Members of the CREST superfamily share seven predicted core transmembrane segments with several conserved sequence motifs.

**Conclusions:**

Universal conservation of a set of histidine and aspartate residues across all groups in the CREST superfamily, coupled with independent discoveries of hydrolase activities in alkaline ceramidases and the Per1 family as well as results from previous mutational studies of Per1, suggests that the majority of CREST members are metal-dependent hydrolases.

**Reviewers:**

This article was reviewed by Kira S. Markarova, Igor B. Zhulin and Rob Knight.

## Background

Membrane proteins play important roles in various biological processes such as transportation and signal transduction across the membrane. Although a significant fraction of proteins in genomes are predicted to be integral membrane proteins [1], our understandings of their function, mechanism and evolution remain limited due to experimental difficulties in assaying their activities and obtaining their structures. The low complexity of the hydrophobic membrane-spanning segments also renders transmembrane proteins difficult targets for computational sequence analysis and structure prediction [2].

Membrane-bound enzymes catalyze a variety of key reactions involving proteins, lipids or other substrates within or near lipid bilayers. A limited number of membrane-bound enzymes with known structures exist, including several intramembrane proteases [3] and members of the MAPEG (membrane-associated proteins in eicosanoid and glutathione metabolism) family [4]. A family of alkaline ceramidases, which are seven-transmembrane proteins without known structures, catalyze the hydrolysis of the amide bond in ceramide to form sphingosine [5]. Sphingosine, ceramide and their phosphorylated products are bioactive sphingolipid molecules involved in a number of biological processes [6]. Recently, a large group of putative transmembrane hormone receptors called progestin and adipoQ receptors (PAQRs) were found to be distantly related to alkaline ceramidases, raising the possibility that they may also function as membrane enzymes [7,8]. Interestingly, the bacterial homologs of these receptors have hemolysin activities [9].

In this study, we employed sensitive sequence comparison methods to search for homologs of alkaline ceramidases and PAQRs. Three additional known protein families were identified: the Per1 family of fatty acid remodeling hydrolases for GPI-anchored proteins [10,11], the SID-1 family of putative RNA transporters involved in systematic RNA interference [12,13], and the TMEM8 family of putative tumor suppressors [14,15]. In addition, we identified distant homologs comprising five distinct groups of proteins with unknown function. We unified these proteins into a superfamily of putative transmembrane hydrolases called CREST (alkaline ceramidase, PAQR receptor, Per1, SID-1 and TMEM8). They share a core structure of seven predicted transmembrane segments and five conserved residues (three histidines, one aspartic acid and one serine). Sequence conservation and mutational studies in the Per1 family [10] suggest that most members of this superfamily are metal-dependent hydrolases. Our analyses and predictions offer insights into functions and mechanisms for members in this diverse superfamily.

## Results

### Sequence similarity searches and homology inference for the CREST superfamily

Extensive transitive PSI-BLAST [16] searches (see Methods for details) uncovered statistically significant sequence similarities between alkaline ceramidases, PAQR receptors, bacterial hemolysins III, the SID-1 family, and the TMEM8 family. For example, a PSI-BLAST search starting with a human alkaline ceramidase ACER3 (NCBI gene identification (gi) number: 296439452, with an e-value inclusion cutoff of 1e-4) found a bacterial hypothetical protein (gi: 114706410 from *Fulvimarina pelagi*) with an e-value of 6e-7 in the fourth iteration. The PSI-BLAST search using this bacterial protein identified statistically significant similarities to PAQR receptors and various bacterial hemolysins III (e.g. a human PAQR receptor (gi: 38018661) was found in the ninth iteration with an e-value of 1e-6 and a sequence identity of 13%). Similarly, the homologous relationship between alkaline ceramidases and the SID-1 family was supported by statistically significant hits through intermediate protein sequences (e.g. a hypothetical protein from *Nitrosomonas eutropha *(gi: 114331832) was found by human ACER3 with an e-value of 2e-6 in the seventh iteration, and it in turn found a sea urchin SID-1 protein (gi: 115686293) with an e-value of 4e-06 in the third iteration). A PSI-BLAST search starting with a bacterial hemolysin III (gi: 1708219 from *Bacillus cereus*) found a TMEM8 protein (gi: 47227992 from *Tetraodon nigroviridis*) with an e-value of 9e-6 and a sequence identity of 12% at the fourth iteration. Conversely, transitive PSI-BLAST searches starting from TMEM8 proteins also found PAQRs and bacterial hemolysins III with statistically significant e-values (less than 1e-4).

**Figure 1:**
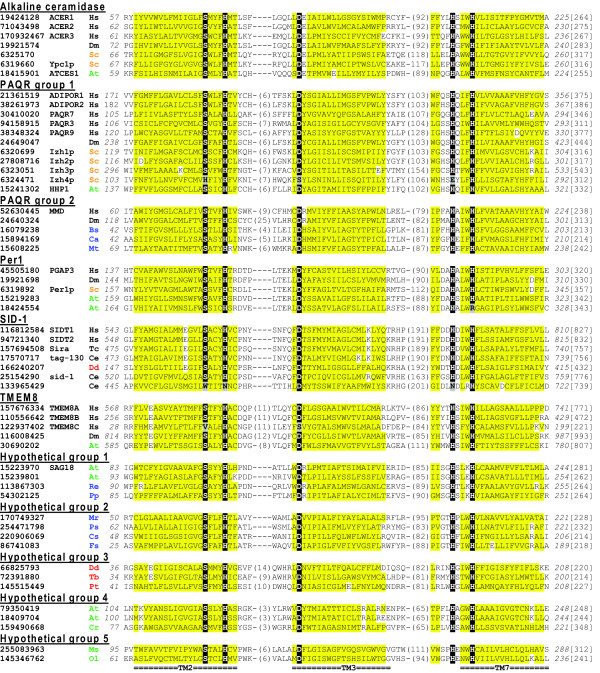
**A multiple sequence alignment of CREST proteins**. Three predicted core transmembrane segments (labeled TM2, TM3 and TM7 respectively below the sequences) with conserved motifs are shown for representative sequences of eleven CREST groups. Putative active site residues are shown on black background, whereas mutations in these positions are on grey background. Non-charged residues in mainly hydrophobic positions are on yellow background. NCBI gene identification numbers, along with common names for some proteins, are shown before the species abbreviations. The numbers of residues in between the three segments are shown in parentheses. Starting/ending residue numbers and sequence lengths are shown in italic font and in brackets, respectively. Species abbreviations are as follows: At, *Arabidopsis thaliana*; Bs, *Bacillus subtilis*; Ca, *Clostridium acetobutylicum*; Ce, *Caenorhabditis elegans*; Cr, *Chlamydomonas reinhardtii*; Cs, *Cyanothece *sp.; Dd, *Dictyostelium discoideum*; Dm, *Drosophila melanogaster*; Fs, *Frankia *sp.; Hs, *Homo sapiens*; Mp, *Micromonas *sp.; Mr, *Methylobacterium radiotolerans*; Mt, *Mycobacterium tuberculosis*; Ol, *Ostreococcus lucimarinus*; Pp, *Photobacterium profundum*; Ps, *Pseudovibrio *sp.; Pt, *Paramecium tetraurelia*; Re, *Ralstonia eutropha*; Sc, *Saccharomyces cerevisiae*; Tb, *Trypanosoma brucei*; Tc, *Tribolium castaneum*. They are colored as follows: metazoans, black; fungi, brown; plants, green; protists, red; and bacteria, blue.

Using the sensitive profile-profile-based similarity search method HHpred [17], we also identified the Per1 family to be distantly related to alkaline ceramidases. For example, an HHpred search using the profile built from the human alkaline ceramidase ACER3 (gi: 296439452) against the Pfam profile database identified the Per1 family (Pfam: PF04080, alignment coverage: 54 to 265 out of 267 profile positions of PF04080, alignment sequence identity: 17%) with a probability score of 98.3 and an e-value of 1e-4. HHpred searches also reinforced other findings in PSI-BLAST searches. For example, the HHpred search using the same alkaline ceramidase query against the profile database of human proteome found a human SID-1 protein (gi: 8923171, alignment coverage: residues 472 to 812, sequence identity: 18%) with a probability score of 97.4 and an e-value of 0.028. Examination of the PSI-BLAST and HHpred alignments revealed several motifs harboring conserved residues aligned between members of these families (Figure 1, with motifs described below), further supporting the proposed homologous relationships among them. Transitive PSI-BLAST searches starting from members of the SID-1 family and the Per1 family converged to closely related sequences respectively, suggesting that each of them is distantly related to the other families.

Additional support of homology among these families was obtained by online CSI-BLAST [18] searches (e-value inclusion cutoff 1e-4, against nr database). For example, a CSI-BLAST search starting from the human alkaline ceramidase ACER3 found PAQR, TMEM8, SID-1 and Per1 family members with statistically significant e-values (less than 1e-4).

### Sequence groups of the CREST superfamily

**Figure 2:**
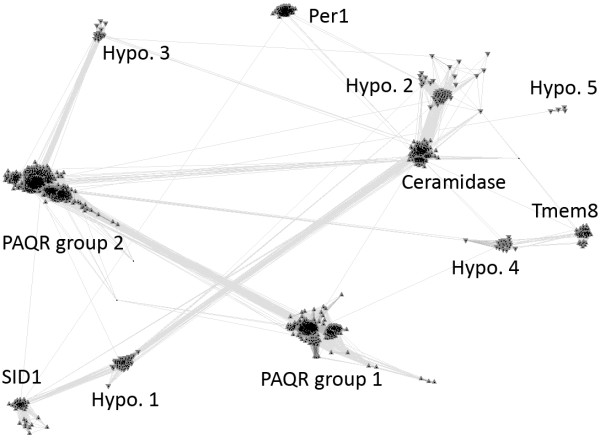
**A sequence cluster diagram of CREST proteins**. This diagram is generated by the CLANS program on a non-redundant set of CREST domains (see Methods). Protein domains are represented as dots and the connections between them suggest BLAST hits with p-values less than 1e-6. Eleven groups of proteins are labeled. 'Hypo.' is the abbreviation for a hypothetical protein group. Members from known families and hypothetical groups have upper and lower triangle contours, respectively.

We identified nearly 3000 CREST members from the NCBI non-redundant database using transitive PSI-BLAST searches starting from queries in alkaline ceramidase, PAQR, Per1, SID-1 and TMEM8 families (see Additional file 1 for the list of proteins). Clustering of the transmembrane domains in these proteins by CLANS [19] revealed eleven major sequence groups (Figure 2) described below. A multiple sequence alignment of representative members of these groups is shown in Figure 1. It covers three predicted core transmembrane segments with putative active site residues (see Additional file 2 for an alignment of all seven predicted core transmembrane segments).

#### Alkaline ceramidases

Three types of ceramidases exist in mammalian genomes and are classified according the the pH optima of their catalytic activity [5]. Acid ceramidase resides in the lysosome and its catalytic domain exhibits similarity to members of the Ntn (N-terminal nucleophile)-hydrolases [20]. Neutral ceramidase is a metal-dependent hydrolase with a different structural fold [21]. The three copies of alkaline ceramidases in mammalian genomes are transmembrane proteins located in the ER and Golgi that have been indicated in intracellular sphingolipid regulation [5,22]. Close homologs of mammalian alkaline ceramidases form a tight group in the CLANS clustering result (Figure 2). Alkaline ceramidases are populated in other eukaryotic organisms, such as insects, fungi and plants. Interestingly, alkaline ceramidases from both fungi [23] and insects [24] have been shown to have a negative effect on life span, suggesting that they are potential targets in ageing studies.

#### Two distinct groups of PAQR receptors

Several members of the PAQR receptor family [25] have been identified as putative receptors for progestin [26,27] and the adipose-derived protein adiponectin (also called adipoQ) [28]. Adiponectin and its receptors are associated with a number of pathophysiological conditions including obesity and type 2 diabetes [29,30]. The CLANS sequence clustering revealed two distinct groups in the large PAQR family (Figure 2) comprising nearly 2,000 eukaryotic and bacterial proteins.

The first PAQR group consists of only eukaryotic proteins and includes the originally identified adiponectin receptors in mammals [28] and the putative membrane-bound progestin receptors in fish [26] and their mammalian orthologs [31] (their subcellular localization and function as progestin receptors have been controversial and under debate [27]). This group also includes members from fungi [32], plants [33] and various protists. An expansion of this group occurs in many eukaryotic species, e.g., the human genome has nine members and the budding yeast *Saccharomyces cerevisiae *has four members (Izh1-4p, Figure 1), suggesting that these putative receptors can potentially accept a variety of ligands. The four *S. cerevisiae *members have been shown to play a role in zinc metabolism [32]. One of them (Izh2p) was also shown to be involved in lipid and phosphate metabolism [34] and to inhibit the expression of a gene involved in iron uptake [35].

The second PAQR group consists of bacterial and eukaryotic proteins. The bacterial homologs are frequently annotated as 'hemolysin III', suggesting the role of a cytotoxin. A bacterial member from *Bacillus cereus *was shown to be a pore-forming hemolysin [9]. The same activity was demonstrated for a homolog from the Gram-negative pathogen *Vibrio vulnificus *[36]. Members of this group are widely distributed in various Gram-positive and Gram-negative bacterial phyla (see Additional file 1). The STRING protein-protein interaction server [37] revealed that some of the bacterial hemolysin III genes are neighbors to a gene encoding a hypothetical protein that contains a DegV domain (Pfam entry: PF02645) known to bind fatty acids (e.g., gi: 81428574 from *Lactobacillus sakei *found a DegV domain-containing protein LSA0964 with a score of 0.752). For some other bacterial members, the STRING server reported the gene neighborhood association with a DUF1836 domain-containing protein (e.g. gi: 157151576 from *Streptococcus gordonii *found a DUF1836 domain-containing hypothetical protein SGO_1308 with a score of 0.877). DUF1836 is classified in the helix-turn-helix (HTH) clan in the Pfam database and could serve as a transcriptional regulator for some of the bacterial hemolysins III. The two human proteins from the second group (PAQR10 and PAQR11) are annotated as 'monocyte to macrophage differentiation factors' [38] with limited experimental studies [39]. Compared to the first PAQR group, the second group has a more restricted distribution in eukaryotes. This group has no plant members and the only two species from fungi in this group belong to the parasitic *Microsporidia *phylum.

#### The Per1 family

The Per1p protein in *S. cerevisiae *was originally identified as a suppressor of the *cdc1 *mutant [40] and was suggested to play a role in manganese homeostasis [41]. Later studies showed that Per1p and its mammalian ortholog PGAP3 are involved in the lipid remodeling of GPI-anchored proteins [10,11]. This remodeling process involves the detachment of lipid chains from the intermediate GPI-anchored proteins and the reattachment of new, usually saturated lipid chains, thus facilitating the localization of GPI-anchored proteins to specialized microdomains ('lipid rafts'). Both the yeast Per1p and the mammalian PGAP3 were shown to be required for the hydrolysis of lipid moieties from GPI-anchored proteins. Direct evidence of hydrolase activity in Per1p or PGAP3 is lacking. However, their homology relationship to alkaline ceramidase (which catalyzes a similar hydrolysis reaction) and the conservation of similar residues (discussed below) together suggest that Per1p and PGAP3 are hydrolases. The Per1 family (Figure 2) comprises about 150 closely related eukaryotic proteins mainly from fungi, plants and metazoans.

#### The SID-1 family

The SID-1 protein from *Caenorhabditis elegans *was characterized as a transmembrane protein that facilitates double-stranded RNA (dsRNA) uptake and thus plays a key role in the systematic RNAi response [12,13]. Its mammalian homologs were also implicated in the RNAi pathways [42,43]. SID-1 members have a restricted distribution in eukaryotes, as they are only found in metazoan species and a few lower organisms such as *Dictyostelium discoideum *and *Monosiga brevicollis*. Mammalian genomes contain two closely related SID-1 homologs, while copy number varies in insects (e.g., no copies in *Drosophila melanogaster *but up to three copies in *Tribolium castaneum*) [44,45]. SID-1 proteins are usually long with more than 800 amino acids and have four additional predicted membrane segments in addition to the seven core membrane segments (discussed below).

Lineage-specific expansion of the SID-1 family is observed in *C. elegans *and *Caenorhabditis briggsae*, with several divergent copies of SID-1-like proteins in both genomes. In *C. elegans*, only the originally identified SID-1 protein and another SID-1-like protein tag-130 possess the complete set of conserved residues (Figure 1), while the other SID-1-like proteins could have lost the proposed hydrolase activity. The SID-1-like protein tag-130 in *C. elegans *exhibits higher sequence similarity to SID-1-like proteins in mammals and insects than the originally identified *C. elegans *SID-1 protein. Experimental studies have shown that unlike SID-1, tag-130 does not contribute to systematic RNAi response in *C. elegans *[44] and its function remains to be investigated.

#### The TMEM8 family

The TMEM8 family proteins share a Pfam domain of unknown function (DUF3522) corresponding to the transmembrane region that is homologous to other CREST members. They are present in metazoans and plants. Vertebrate genomes have three copies of TMEM8 proteins (TMEM8a-c) while most insects only have one copy. TMEM8a and TMEM8b in vertebrates, as well as the TMEM8 family members in other metazoan species and plants, are multi-domain proteins usually with more than 400 amino acids. The transmembrane domains are located at their C-termini. They also possess a divergent EGF domain with six conserved cysteines (detected by HHpred) and an unannotated N-terminal region. TMEM8c proteins in vertebrates are single-domain proteins of about 220 amino acids. The conserved serine and aspartic acid are absent in TMEM8c proteins (Figure 1), possibly resulting in the loss of hydrolase activity. Recently, several studies have indicated that human TMEM8b (also named NGX6 for nasopharyngeal carcinoma associated gene 6) was down-regulated in several cancer cell lines and may be a tumor suppressor [14,15].

#### Five groups of proteins with unknown function

**Figure 3:**
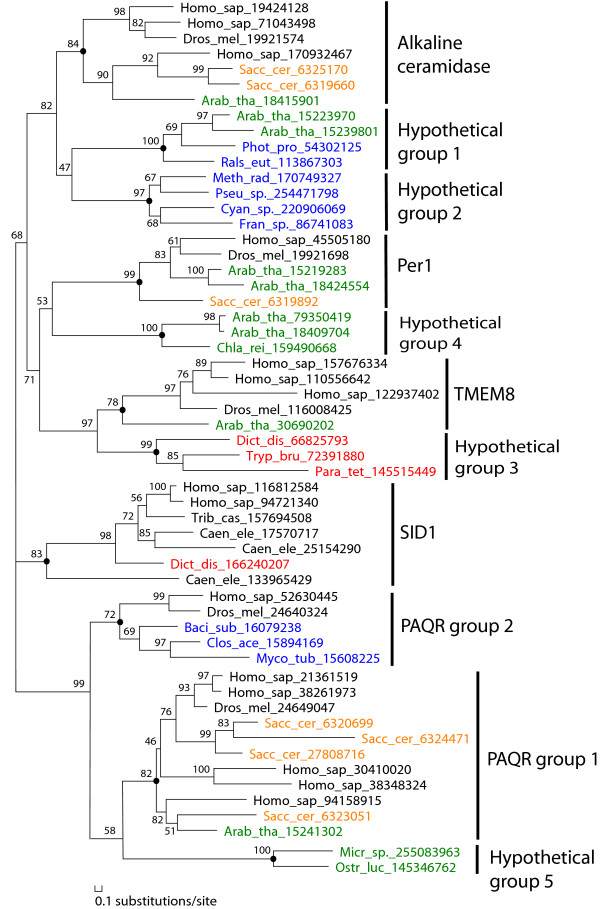
**Phylogenetic reconstruction of CREST proteins by MOLPHY**. This phylogenetic tree is made by MOLPHY (see Methods) using the CREST proteins shown in Figure 1. Each protein is denoted by its species name abbreviation and its gi number. Bootstrap percentages are shown for the internal nodes. The eleven CREST groups are labeled to the right of the tree. The root of each group is marked by a black circle. Species abbreviations are as follows: Arab_tha, *Arabidopsis thaliana*; Baci_sub, *Bacillus subtilis*; Caen_ele, *Caenorhabditis elegans*; Chla_rei, *Chlamydomonas reinhardtii*; Clos_ace, *Clostridium acetobutylicum*; Cyan_sp., *Cyanothece *sp.; Dict_dis, *Dictyostelium discoideum*; Dros_mel, *Drosophila melanogaster*; Fran_sp., *Frankia *sp.; Homo_sap, *Homo sapiens*; Meth_rad, *Methylobacterium radiotolerans*; Micr_sp., *Micromonas *sp.; Myco_tub, *Mycobacterium tuberculosis*; Ostr_luc, *Ostreococcus lucimarinus*; Para_tet, *Paramecium tetraurelia*; Phot_pro, *Photobacterium profundum*; Pseu_sp., *Pseudovibrio *sp.; Rals_eut, *Ralstonia eutropha*; Sacc_cer, *Saccharomyces cerevisiae*; Trib_cas, *Tribolium castaneum*; and Tryp_bru, *Trypanosoma brucei*. Sequence names are colored as follows: metazoans, black; fungi, brown; plants, green; protists, red; and bacteria, blue.

In addition to the known protein groups, CLANS clustering revealed five groups of proteins of unknown function often annotated as hypothetical proteins (Figures 1 and 2, named hypothetical groups 1-5). Their relationships to the known CREST groups were investigated by the phylogenetic reconstruction of the CREST sequences in Figure 1 using MOLPHY (see Methods). Consistent with the CLANS grouping, each of the eleven groups in Figure 1 is monophyletic in the MOLPHY phylogenetic tree (Figure 3), generally with good statistical support (nine out of the eleven groups with bootstrap percentage values above 80%). A similar tree generated by PhyML also supports the separation of these groups (see Additional file 3).

Hypothetical group 1 consists of proteins from bacteria and plants. One member in this group is SAG18 (gi: 15223970) from *Arabidopsis thaliana*, which was found in a screen of senescence-associated genes [46]. The bacterial members of this group are mainly from Proteobacteria (see Additional file 1). Hypothetical group 2 consists of only bacterial proteins. They are mainly from the Alphaproteobacteria class (see Additional file 1). These two hypothetical groups appear to be more closely related to alkaline ceramidases (with a modest bootstrap percentage value of 82% for grouping them with alkaline ceramidases) than to the other known groups according to the phylogenetic tree (Figure 3). Therefore, it would be interesting to experimentally test the ceramidase activity for these hypothetical proteins. Proteins of hypothetical group 3 are from various protists of genera such as *Trypanosoma, Toxoplasma *and *Paramecium*, as well as from the Choanoflagellate *Monosiga brevicollis*. They form a well-supported clade with the TMEM8 group (Figure 3). Hypothetical groups 4 and 5 consist of proteins from green plants (Viridiplantae) and green algae (Chlorophyta) respectively. Hypothetical group 5 and the two PAQR groups form a well-supported clade, whereas the relationship of hypothetical group 4 to the other groups remains unclear (it clusters with the Per1 group with a low bootstrap support value) (Figure 3).

The phylogenetic tree revealed two separate locations of bacterial sequences (blue names in Figure 3). One cluster of bacterial sequences (from hypothetical groups 1 and 2) are clustered with alkaline ceramidases from eukaryotes, and the other cluster (bacterial hemolysins III) is grouped with PAQRs from eukaryotes. Such a distribution suggests that at least two copies of CREST members are present in the last common ancestor of bacteria and eukaryotes. No CREST members were found from archaeal organisms, likely reflecting the difference of membrane lipid composition of archaea from bacteria and eukaryotes [47]. Alkaline ceramidase and Per1 are hydrolases acting on the amide or ester bond between the hydrophobic acyl group and the hydrophilic head group (sphingosine or glycerol). However, ether bond exists between the acyl group and the head group of archaeal membrane lipids, in contrast to the ester or amide bond in bacterial and eukaryotic membrane lipids.

### Domain architecture and sequence motifs of the CREST superfamily

**Figure 4:**
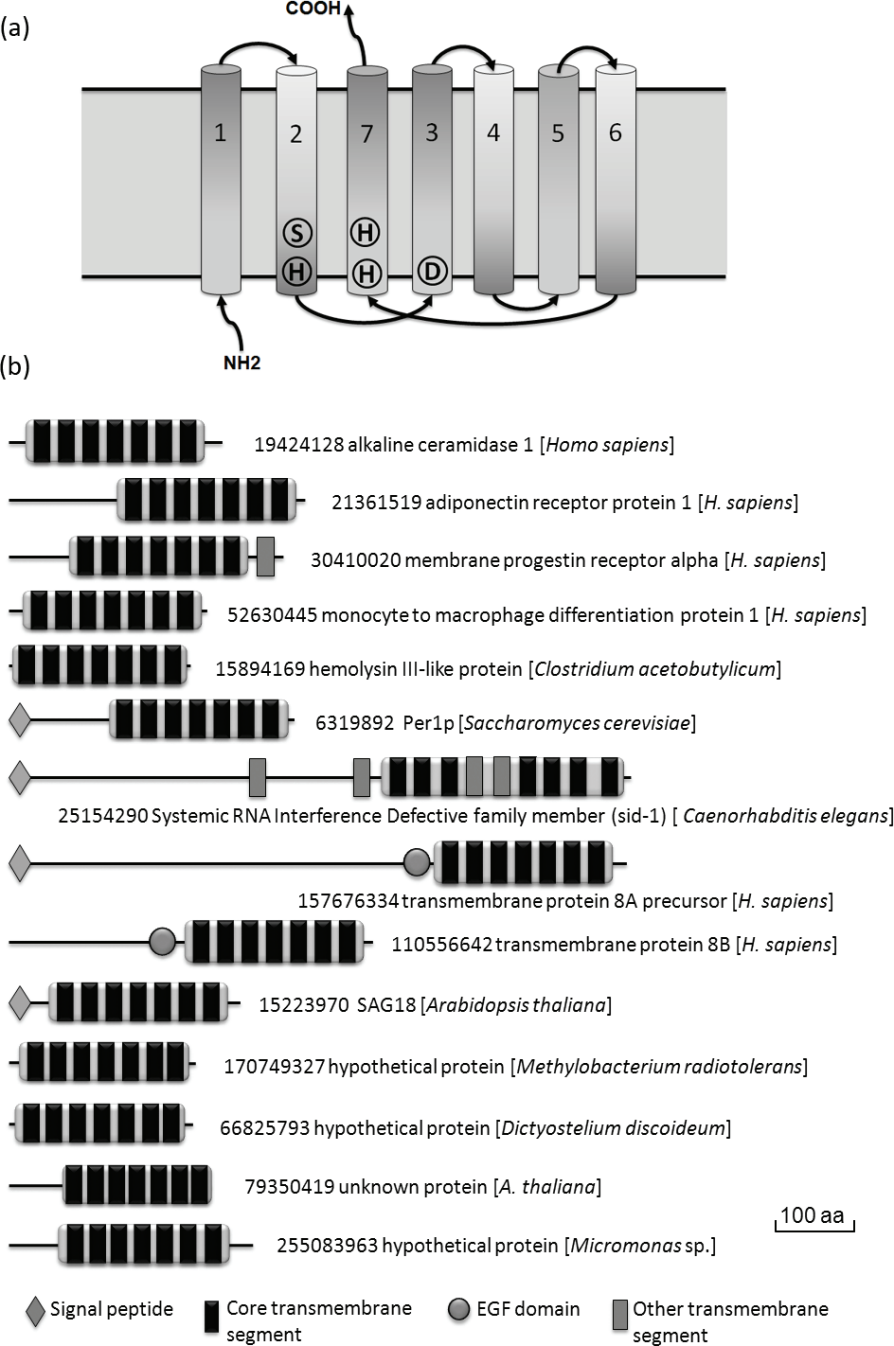
**A diagram of predicted core transmembrane segments and domain diagrams of CREST proteins**. (a) A cartoon diagram showing the seven predicted core transmembrane segments of CREST proteins. The N- and C-termini are labeled. The predicted core transmembrane segments (TM) are labeled 1-7 from the N-terminus to the C-terminus. TM7 is placed as neighboring to TM2 and TM3 to reflect possible spatial closeness among TM2, TM3 and TM7 that harbor motifs with conserved putative active site residues (shown inside them). (b) Domain diagrams of representative CREST members. NCBI gene identification numbers and annotations are shown beside the domain diagrams. Species names are shown in brackets. The last five proteins are from hypothetical groups 1-5, respectively.

Members of the CREST superfamily share seven predicted core transmembrane segments (Figure 4a). Some members of the PAQRs, such as human progestin receptor α, have an additional eighth predicted membrane segment at the C-terminus (Figure 4b). The N- and C-terminal soluble segments of most CREST members are generally short, e.g., the N-terminus of the Per1 family proteins contains an uncharacterized short sequence segment with several conserved cysteines. The N-terminal segment in alkaline ceramidase 2 (ACER2) were found to be indispensible for its enzymatic activity [48]. A unique feature of the SID-1 family proteins is the presence of four more predicted transmembrane segments in addition to the seven core membrane segments. Two of the four additional predicted transmembrane segments are located N-terminally to the CREST domain. As SID-1 was proposed to be an RNA transporter, these two additional predicted transmembrane segments could contribute to pore forming in the membrane similar to ion-channel-linked receptors. The other two additional predicted transmembrane segments are inserted within the core CREST domain (Figure 4b). They probably would not affect the topology of the core structure and the active site placement if they form an up-and-down helical hairpin commonly found in transmembrane proteins. Alternatively, they could be embedded hydrophobic segments that do not span the lipid bilayer, as indicated by the gene truncation experiment aimed at determining the topology of SID-1 [13] (it should be noted that truncation of a transmembrane protein could cause structural changes or misfolding and lead to inconsistent results, which were observed in some of the SID-1 truncations [13]). The additional membrane segments in SID-1 may contribute to structural stability or substrate gating that is also found in intramembrane proteases [3]. Except for some long bacterial hemolysin III proteins and the EGF domain-containing TMEM8 members (Figure 4b), no other known domains were detected in CREST members. Signal peptides were predicted in Per1, SID-1 and some TMEM8 family members.

Three conserved sequence motifs with semi-invariant residues were identified in all CREST groups (Figure 1). The first motif SxxxH ('x' is any amino acid) is located at the end of the second predicted core transmembrane segment. The second motif with a conserved aspartic acid is located at the beginning of the third predicted core transmembrane segment. The third motif with two conserved histidines (HxxxH) resides at the beginning of the seventh predicted core transmembrane segment. We also observed a conserved position comprising mainly small residues in the sixth predicted core transmembrane segment (see Additional file 2). Topologically, the three motifs are predicted to be located near the same side of the membrane and thus may be proximal to each other and form the putative active site (Figure 4a). The three predicted core transmembrane segments (TM2, TM3 and TM7) harboring the motifs could also be spatially close to each other (shown in Figure 4a). Coincidently, in the seven-transmembrane GPCR structures [49], transmembrane segments 2, 3 and 7 are also spatially close and interact with each other. We did not detect statistically significant sequence similarities between CREST members and GPCRs. The set of CREST-specific sequence motifs was not found in GPCRs, and GPCRs have a different topology than PAQRs in the plasma membrane (discussed below). Structure determination of CREST members could help elucidate whether CREST members exhibit a similar structural fold as GPCRs and whether they are evolutionarily related to GPCRs.

## Discussion

Cataloguing distantly related protein families into superfamilies helps understanding their evolution and deducing useful information about their structure and function. Such a level of classification is used in protein structure classification databases such as SCOP [50] and CATH [51] (the superfamily level), the Pfam protein families database [52] (the clan level), the MEROPS peptidase database [53] (the clan level), and in membrane transporter classification [54] (the superfamily level). Distantly related protein families can result from gene duplication events followed by sequence and functional divergence, or from accelerated evolution in certain evolutionary lineages. Although they can have different cellular functions and/or phylogenetic distributions, distantly related protein families often share similar biochemical activities, exemplified by enzymes performing similar reactions. The identities and placements of active site residues responsible for their common biochemical activities are usually conserved as well. Cross-referencing information of distantly related protein families can shed light on their function and help experimental design.

In this study, we unified five known protein families as well as several groups of proteins with unknown function into a large and diverse superfamily of putative transmembrane hydrolases comprising nearly 3000 sequences in the current sequence database. The statistically significant sequence similarities detected by multiple similarity search methods, together with shared sequence motifs, support the homologous relationships among members of the CREST superfamily. The most striking feature of the CREST superfamily of transmembrane proteins is the large functional capacity revealed by independent experimental studies of its members, with diverse and seemingly unrelated functions of hormone receptors (PAQRs), bacterial hemolysins, dsRNA transporters (SID-1) and putative tumor suppressers (TMEM8). It also remains unclear how the putative hydrolase activity contributes to these functions. The hydrolase prediction could open new directions for the future research of CREST members.

**Figure 5:**
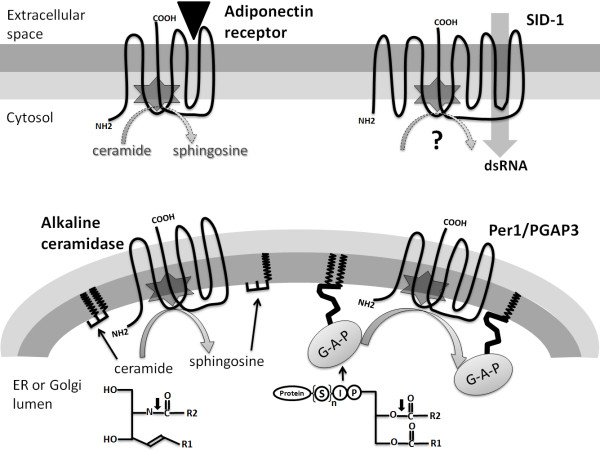
**The topology and subcellular localization of four CREST proteins**. Like in Figure 4a, TM7 is set as neighboring to TM2 and TM3 to reflect possible spatial closeness between TM2, TM3 and TM7. Putative active site locations are marked by six-point stars. Alkaline ceramidases and Per1/PGAP3 are located in the ER or the Golgi membrane with their C-termini in the cytosol. Alkaline ceramidases catalyze the hydrolysis of ceramide to sphingosine. Per1/PGAP3 is required for phospholipase A2 activity that removes one of the two fatty acid chains (shown in zigzagged shapes) from the GPI-anchored protein (labeled 'G-A-P'). The chemical structures of ceramide and the GPI-anchored protein are shown (R1 and R2 are two aliphatic chains; P, I and S in circles represent phosphate group, inositol group, and suger or other groups connecting to the protein, respectively). The amide bond in ceramide hydrolyzed by alkaline ceramidase and the ester bond in the GPI-anchored protein hydrolyzed by Per1/PGAP3 are indicated by downward arrows. Adiponectin receptors and SID-1 are located in the plasma membrane with their N-termini in the cytosol. Adiponectin receptors interact with the extracellular hormone adiponectin (shown as a triangle) and may possess ceramidase or other hydrolase activity (marked by a dotted arrow). The putative dsRNA transporter SID-1 may possess an unknown hydrolase activity (marked by a question mark).

The hydrolase activity has been independently assigned to the alkaline ceramidase family [5] and the Per1 family [10,11]. They catalyze similar reactions on different substrates (Figure 5). Alkaline ceramidase hydrolyzes the amide bond in ceramide to produce sphingosine and a fatty acid. Per1 is responsible for the phospholipase A2 activity that removes fatty acids in GPI-anchored proteins. Human alkaline ceramidase 2 (ACER2), an enzyme residing in the membrane of the Golgi apparatus, was shown to have its C-terminus in the cytosol [48]. Such a topology positions its putative active site residues (Figure 4a) near the lumen side of the Golgi membrane (Figure 5). Therefore, the substrates and products of the catalysis would be within or near the Golgi inner lipid layer (Figure 5). Likewise, the ER membrane-located human alkaline ceramidase 1 (ACER1) [55] is predicted to have the same topology as ACER2 [38]. Per1 is also located in the ER membrane and has the same predicted topology with its C-terminus in the cytosol (Figure 5). Such a topology is consistent with the ER localization of Per1's substrates, GPI-anchored proteins (before they are delivered to the plasma membrane), which are anchored to the inner lipid layer of the ER membrane using fatty acids [56] (Figure 5). The substrates of alkaline ceramidase and Per1 have similar chemical structures in their lipid moieties with two aliphatic chains (Figure 5), suggesting similar modes of substrate binding and catalytic mechanisms.

Based on the inferred homologous relationship between PAQRs and alkaline ceramidases, it has been speculated that PAQRs could function as ceramidases and generate sphingosine as a second messenger for downstream signaling [7]. Although no direct evidence of these receptors being hydrolases currently exist, experimental results showed that sphingolipids function as downstream effectors of a PAQR member in yeast [7], and that a ceramidase inhibitor can antagonize human PAQRs [57]. Furthermore, a latest study showed that adiponectin can induce increased ceramidase activity and this effect is associated with adiponectin receptors [8]. As two of the CREST groups, alkaline ceramidase and Per1, perform similar yet distinct hydrolysis reactions on different substrates, it is also possible that the substrates and reaction products of the other CREST groups differ, which could contribute to their functional diversity. Therefore, the future experimental studies of PAQRs should not be restricted to just the potential effects of ceramidase activity. Other hydrolase activities on various lipid molecules, especially the phospholipase A2 activity (suggested for Per1), and their cellular effects can be tested for PAQR members. The products of the phospholipase A2 activity on a phospholipid substrate are a lysophospholipid molecule and a free fatty acid [58], both of which are potential precursors of second messengers that could regulate downstream signaling events [59,60].

Classic seven-transmembrane GPCR receptors transduce signals across the plasma membrane and rely on a separate effector protein (such as adenylate cyclase or phospholipase C) to produce second messengers. Well-studied enzyme-linked receptors, such as receptor tyrosine kinases, receptor guanylate cyclases and histidine kinases, are multi-domain proteins that have soluble domains in the cytosol with enzymatic activities. If PAQRs do prove to be receptors with hydrolase activity, they would represent a unique type of receptors that uses the enzymatic activity in the transmembrane domain to generate second messengers (such as sphingosine resulted from ceramidase activity) or their precursors (such as lysophospholipid molecules resulted from phospholipase A2 activity). Further studies are required to determine if PAQRs possess intrinsic ceramidase or other hydrolase activity. A recent genetic linkage analysis revealed that one single nucleotide polymorphism (rs10920533) in human *ADIPOR1 *(the gene encoding adiponectin receptor 1) interacts with plasma saturated fatty acids [61], indirectly supporting its role in lipid modification. Another interesting question is if and how the hydrolase activity can be modulated by the binding of extracellular ligands such as adiponectin. The second group of PAQRs includes bacterial hemolysins. Besides pore forming [9], these toxins could exert their virulence through the putative hydrolase activity, e.g., by changing membrane lipid composition or generating second messengers to modify signaling events in host cells.

In contrast to GPCR receptors, human adiponectin receptors have an internal N-terminus and an extracellular C-terminus [28], thus positioning the conserved, putative active site residues near the cytosol side of the plasma membrane (Figure 5). The other human PAQR members were also predicted to have their N-termini residing in the cytosol (predictions made by Phobius [38]). The *C. elegans *SID-1, also located in the plasma membrane, adopts a topology similar to adiponectin receptors with its N-terminus in the cytosol and its putative active site near the inner lipid layer of the plasma membrane [13] (Figure 5). SID-1 was proposed to be a dsRNA transporter as its transfection to *Drosophila *(lacking an endogenic SID-1 ortholog) cells is sufficient to enable dsRNA uptake [13,62]. In this case, SID-1 has the pore-forming ability similar to bacterial hemolysins III. In a less likely scenario, SID-1 itself is not a dsRNA transporter, but indirectly causes dsRNA uptake by regulating the activity of a separate dsRNA transporter [13]. The proposed hydrolase activity of SID-1 could be involved in regulating its dsRNA transport ability, whether such a transport process is directly or indirectly mediated by SID-1. It would also be interesting to test the hydrolase activity and its roles in the TMEM8 family of putative tumor suppressors.

The specific roles of the conserved residues in CREST proteins have not been reported, and the catalytic mechanisms of alkaline ceramidases and Per1 are yet to be determined. Mutational studies performed on yeast Per1p [10] with the proposed phospholipase A2 activity offer valuable information about the relative importance of some conserved residues. Alanine mutation of either the conserved histidine in the first motif ('SxxxH') or the first conserved histidine in the third motif ('HxxxH') abolished Per1p activity, suggesting that these histidines are candidates of active site residues that directly contribute to catalysis. On the other hand, alanine mutation of the conserved serine residue in the first motif (the only serine conserved among all CREST groups), as well as alanine mutations of several other residues that are not conserved among all CREST groups, did not affect the activity of Per1p. Therefore, the proposed phospholipase A2 activity of Per1p is unlikely to have a serine esterase-type mechanism that is utilized in certain groups of soluble phospholipase A2 enzymes with an α/β hydrolase fold [58]. Although the conserved serine in the first motif may not directly involve in catalysis, it could participate in hydrogen-bonding interactions with an active site residue to help maintain its optimal orientation for metal-binding or catalysis. This serine appears to be important for the activity of SID-1, as one of the RNA interference-defective SID-1 mutants has a single mutation of this serine to an isoleucine (see the supplemental Figure S3 in reference [12]).

Three histidines and one aspartate are conserved in CREST proteins, reminiscent of the active site composition of several well-studied metal-dependent hydrolases such as thermolysin [63], bovine carboxypeptidase A [64], D-Ala-D-Ala carboxypeptidase [65] and the intramembrane S2P-like proteases [66]. These enzymes adopt different structural folds, yet they all utilize histidines and negatively charged residues for their catalytic activity (three residues used for metal binding and one residue used for activating a metal-bound water molecule as the nucleophile to attack the scissile bond). The presence of such a set of conserved residues in the CREST superfamily and the mutational results of yeast Per1p support the hypothesis that most CREST members are metal-dependent hydrolases. A large group of secreted enzymes with phospholipase A2 activity (proposed for Per1p) are also metal-dependent hydrolases with histidines in their active sites [58].

## Conclusions

We inferred distant homologous relationships among five transmembrane protein families (alkaline ceramidase, PAQR, Per1, SID-1 and TMEM8) and several groups of proteins with unknown function. Members of this superfamily share seven predicted core transmembrane segments and a set of conserved histidine and aspartate residues. Such a conservation pattern coupled with experimental evidence suggests that they are putative metal-dependent hydrolases acting on molecules with fatty acid moieties. It remains unclear how such putative hydrolase activity contributes to the diverse and seemingly unrelated functions of hormone receptors, bacterial hemolysins, the SID-1 dsRNA transporters and the TMEM8 putative tumor suppressers. Further experimental investigations could reveal new aspects of lipid biology in the actions of these transmembrane proteins.

## Methods

### Sequence similarity searches

PSI-BLAST [16] was used to search for homologs of the alkaline ceramidase family starting with the human alkaline ceramidase protein ACER3 (NCBI gene identification (gi) number: 296439452) against the non-redundant (nr) protein database (e-value inclusion cutoff: 1e-4, more stringent than the default value in NCBI PSI-BLAST web server (0.005)). To perform transitive searches, the protein hits found by PSI-BLAST were grouped by BLASTCLUST (with the score coverage threshold (-S, defined as the bit score divided by alignment length) set to 1, length coverage threshold (-L) set to 0.5, and no requirement of length coverage on both sequences (-b F)) and a representative sequence from each group was used to initiate new PSI-BLAST searches. The HHpred web server [17] was used for profile-against-profile-based similarity searches using several members found by PSI-BLAST against the Pfam database [52] and the human proteome database with default parameter settings. Newly identified human homologs were again subject to transitive PSI-BLAST searches. Several CREST members were also submitted to the CSI-BLAST server [18] (database: nr; e-value inclusion cutoff: 1e-4; maximum iteration number: 20) to further verify the findings of PSI-BLAST and HHpred.

### Domain architecture analysis

HMMER3 [67] and HHpred were used to detect known Pfam domains (Pfam version: 24.0) in CREST members with default parameter settings. Phobius [38] was used to predict transmembrane segments and membrane topology. Phobius and SignalP 3.0 [68] were used to predict signal peptides.

### Sequence clustering, alignment and phylogenetic reconstruction

For sequence clustering, highly similar sequences were removed at the 95% identity level by CD-HIT [69]. Sequence fragments with less than 100 residues were removed. CLANS [19] was used to cluster the reduced non-redundant set of domains based on pairwise BLAST p-values. The clustering was run to equilibrium in a 2D representation under default settings. Connections between points (sequences) in the final diagram were set by a BLAST P-value cutoff of 1e-6. For sequence alignment, representative CREST members in each group were manually selected to sample sequences from diverse organisms with emphasis on those that have been experimentally studied and described in this article. The multiple sequence alignment of these representatives was made by PROMALS that uses information of predicted secondary structures and database homologs [70]. The alignment was then improved by manual curation. The MOLPHY package [71] was used for phylogenetic reconstruction based on this alignment (positions with gap fractions larger than 0.1 were removed). The JTT amino acid substitution model [72] was used in MOLPHY. The local estimates of bootstrap percentages were obtained by the RELL method [73] (-R option in the ProtML program of MOLPHY). We also used PhyML (version 3.0) [74] for phylogenetic reconstruction with default parameter settings (the LG amino acid substitution model [75], four rate categories of a discrete gamma model to take into account rate variability among sites, and an approximate likelihood-ratio test for branch support [76]).

## Competing interests

The authors declare that they have no competing interests.

## Authors' contributions

JP carried out the sequence analysis and drafted the manuscript. JP, DM, EO, NG participated in the design of this study. All authors read and approved the final manuscript.

## Reviewers' comments

### Reviewer's report 1

#### Kira S. Markarova, National Center for Biotechnology Information

This paper describes new superfamily of membrane proteins predicted to be metal-dependent hydrolases. Among this superfamily there are several important eukaryotic families that before were not recognized as being homologous. Generally this is a good paper with all conclusions well-supported and justified. In my opinion, however, the important message of the paper could be fit in a shorter format, like, for example, the "Discovery note" in Biology Direct. For instance, the Results section of the paper describing the support for unification of these families is an "overkill". It would be enough to briefly describe the results of either PSI-BLAST or HHpred and mention that other programs support them. The description of the others work related to these families also could be briefer, since not everything what was shown is related to this work. There are other areas for omitting in family description and discussion, just because that they reiterate the same information that is already present in the text somewhere.

#### Authors' response

*We considered other article types when preparing the manuscript, but decided that the length (less than 1500 words) and figure number (no more than one figure or table) limits of the discovery note format could not accommodate the complete contents of this work. In this revised version, one more figure is added to make a total of five figures. We feel that the new figure showing the phylogenetic tree is necessary since it provided additional information about the relationships among the CREST groups and helped address comments raised by all three reviewers. Researchers can have different opinions on the necessity of detailed description of similarity search results. For example, the third reviewer asked for more support from other similarity search methods. We feel that providing details of the similarity search results makes the descriptions scientifically more rigorous and would be appealing to audiences who are interested in these details. We agree that some of the cited works appeared to be not closely related to this work. However, we still considered them worthwhile since not much is known for many CREST groups and the limited existing literature could potentially be useful for cross reference when new experimental data are available in the future. We checked the manuscript and deleted some texts that were reiterated*.

I have also several suggestions and questions.

1. For people who are interested in these proteins it would be helpful to provide a supplementary material with all ~ 3000 identified protein IDs and other related information like organism where it was found and assignment to a family.

#### Authors' response

*We added a supplementary data file listing CREST proteins found by transitive PSI-BLAST searches. They are grouped according to the CLANS clustering results and have species information, definitions and html links to their NCBI records*.

2. There are a lot of bacterial proteins in CREST superfamily. For most of them no functional information is available. Neighborhood analysis can provide some insights (same "guilt by association approach" as the analysis of domain fusions) into their function and point to the potential functionally related gene families. Have you tried to look into it? If not, it is worth checking and reporting.

#### Authors' response

*We previously did try to find functional linkages of bacterial CREST members to other proteins using the STRING functional association server, but did not include the results since we did not obtain substantial information regarding the functions of these proteins. One frequent top hit of the gene neighborhood analysis reported by STRING for bacterial hemolysins III is a hypothetical protein containing a domain of unknown function (DUF1836). Pfam classified DUF1836 in the HTH (helix-turn-helix) clan, which is also supported by HHpred searches of DUF1836 proteins. This linkage suggests that some bacterial hemolysins III are regulated by putative transcription factors with DUF1836 domains. Another hit for some bacterial hemolysins III is a DegV domain-containing protein known to bind fatty acids. Bacterial hemolysin III genes were also frequently linked to proteins with various enzymatic activities, such as methylases, glycosyltransferases and phosphotransferases, mostly with marginal to medium scores (most of the scores examined were below 0.7, which is considered to be a cutoff for high confidence associations in the STRING server). While possible associations with proteins having enzyme activities may suggest that bacterial hemolysins III are also enzymes performing concerted reactions along with other enzymes, we did not obtain further definitive cellular function information based on these associations. For the two groups of hypothetical proteins with bacterial members, we also did not find substantial functional information by the gene association analysis using the STRING server. We added the STRING gene association results linking bacterial hemolysins III to DegV and DUF1836 in the revised manuscript*.

3. The CREST superfamily has two nice motifs: SxxxH and HxxxH. Have you tried to apply motif search in order to identify families with the same motifs but not found by sequence similarity approach? Surprisingly this approach still can be useful (as we recently showed for FtsZ superfamily - PMID: 20459678). In any event it would be interesting to know the outcome of this search.

#### Authors' response

*As the reviewer suggested, we searched the pattern 'SxxxHx(100,10000)HxxxH' (the length in between the two motifs was set to be 100 or above since they are separated by five predicted core transmembrane segments) against the nr database. Over 55000 sequences were found and the majority of the hits appear to be not homologous to CREST members according to our manual inspection (in contrast, a search of the GGGTG(S/T)G motif characteristic of the FtsZ superfamily only retrieved about 7500 hits from the nr database and the majority of them appeared to be FtsZ homologs according to their annotations). Thus we conclude that these two motifs (SxxxH and HxxxH) themselves are not enough for characterization of the CREST members. As these motifs lie within or near the end of the predicted transmembrane segments (TMs, see Figure 1), we devised a new search strategy that takes into account their relative positioning in predicted TMs. The following steps were conducted: (1). Predict the TMs using Phobius for a target protein set. (2). For each protein in the set, analyze the motif patterns for its predicted TMs in the following way. For each predicted TM defined by the starting position i and the ending position j, search for the pattern SxxxH in the range [(i+j)/2, j+5] (corresponding to the C-terminal half of the TM plus 5 residues C-terminal to the TM). Search for the pattern HxxxH in the range [i-5, (i+j)/2] (corresponding to the N-terminal half of the predicted TM plus 5 residues N-terminal to the predicted TM). (3). A positive motif match is reported if SxxxH and HxxxH are found in two separate predicted TMs under the conditions that SxxxH is N-terminal to HxxxH and they are at least 3 predicted TMs apart from each other (SxxxH is in core TM2 and HxxxH is in core TM7 in Figure 1, so they should be at least 5 TMs apart (SID-1 has an additional pair of TMs in between), but to correct for potential missing TMs by the prediction program, this number is relaxed to 3 predicted TMs apart). We applied this strategy to a number of eukaryote proteomes (human, fruit fly, budding yeast and Arabidopsis thaliana) and about 1100 prokaryotic proteomes downloaded from the NCBI ftp site. The results were clustered by BLASTCLUST and each cluster was manually checked. We did not find new candidates of CREST members*.

4. What is your opinion on the origin of CREST family? Could the ancestor be placed in LUCA and if yes, are there any ideas, on its ancestral function. Have you noticed any representatives of CREST family in archaea?

#### Authors' response

*We built a phylogenetic tree using MOLPHY for the eleven groups of representative sequences shown in Figure 1. According to this tree, there are two separate groups of bacterial proteins associated with eukaryotic alkaline ceramidases and PAQR receptors, respectively. We thus proposed in the revised manuscript that there might be at least two copies of CREST members in the last common ancestor of eukaryotes and bacteria. We did not find members of the CREST superfamily in archaea. We proposed in the revised manuscript that the lack of CREST members in archaea is likely related to the difference of archaeal membrane lipid composition from those of the bacteria and eukaryotes. CREST members such as alkaline ceramidase and Per1 are proposed to hydrolyze the amide or ester bond between the hydrophobic acyl group and the hydrophilic head group (sphingosine or glycerol). However, in archaea, such amide and ester bonds are replaced by the ether bonds in membrane lipids. Therefore, the reactions catalyzed by CREST members may not be useful in archaea*.

### Reviewer's report 2

#### Igor B. Zhulin, University of Tennessee at Knoxville

The paper by Pei et al. describes a superfamily of putative transmembrane hydrolases, which includes more than 3000 proteins from eukaryotes and prokaryotes. From the technical point of view, this is a very well executed study. All relationships between protein sequences were established using careful analysis. I have two problems with this paper:

1. The novelty is rather marginal. A relationship between eukaryotic PAQR proteins and bacterial hemolysin-type proteins has been captured in the Pfam HlyIII family (PF03006), which includes more than 2000 transmembrane proteins. Current work does expand this family into a superfamily by essentially combining it with other Pfam families, such as Ceramidase (PF05875), and DUF3522 (PF12036) and also identifies several novel families within this superfamily. This is definitely a finding, but it does not sound too exciting. Authors postulate that "Cataloguing distantly related protein families into superfamilies helps understanding their evolution and deducing useful information about their structure and function". I agree, but this work does not suggest too much about the evolution of this superfamily and useful information about function is rather limited. It appears to me that the most useful information is the identification of conserved residues that suggest the hydrolase activity - something, which is testable experimentally.

#### Authors' response

*We agree that limited functional information was inferred for CREST groups, which is characterized by a diverse functional capacity. To obtain more evolutionary information, we used phylogenetic reconstruction to investigate the relationships between the eleven CREST groups. The phylogenetic trees built by MOLPHY and PhyML were added in the revised manuscript*.

2. More serious concern is that no actual data is provided other than some examples. I understand that there are more than 3000 protein sequences, but there must be some way of presenting it. Figure 1 shows a representative alignment of less than 60 sequences. As for the rest, we have to take authors' word for it. Providing the actual data (as a supplementary file, I suppose) is especially important in this case, because (i) cataloguing is the whole purpose of this work (but "the full catalog" is missing, we've got only "the advertisement with 'best buy' items"), and (ii) in the absence of a model deposited to a public resource (e.g. Pfam) this is the only way for biologists to obtain this information. I think both of these concerns can be easily addressed by placing more emphasis on novelty and potential impact and by providing data.

#### Authors' response

*In the revised manuscript, we provided supplementary data listing the eleven groups of CREST proteins found by transitive PSI-BLAST searches. Besides the linking of various known protein families and hypothetical groups in the CREST superfamily and the identification of the conserved and potential active site residues, we feel that one novel aspect of this work lies in the discovery of a diverse array of cellular functions that this superfamily encompasses. While the alkaline ceramidase and Per1 were proposed to possess hydrolase activities in ceramide or GPI-anchored protein biosynthesis pathways, it is quite interesting how such putative hydrolase activity contributes to the diverse and seemingly unrelated functions of hormone receptors, bacterial hemolysins, the SID1 dsRNA transporters and the TMEM8 putative tumor suppressers. The hydrolase prediction can potentially provide new directions in the future experimental studies of these proteins. We proposed that PAQRs could represent a novel type of receptors that use their transmembrane domains to generate second messengers. We also suggested that the experimental work on these receptors should not be restricted to testing the ceramidase activity, as CREST members can catalyze different reactions exemplified by alkaline ceramidases and Per1. We emphasized these points in the discussion and conclusions of the revised manuscript*.

### Reviewer's report 3

#### Rob Knight, University of Colorado at Boulder

In this manuscript, the authors unite several families of putative transmembrane hydrolases into one superfamily using similarity search methods, specifically iterated PSI-BLAST. The superfamily contains several protein families of unknown function, providing a clear hypothesis concerning the function of these new members (although no experimental data are provided for validation). Overall, the paper is competently written and executed, though not especially groundbreaking. One puzzle is that the authors use iterated PSI-BLAST rather than profile-profile techniques such as COMPASS, which might be worth commenting on as the same group has previously argued that profile-profile methods provide substantially better results than sequence-profile matching, especially for detecting similarities in highly diverged protein families. The technique is also similar to the Shotgun algorithm from Patsy Babbitt's lab and implemented in some of our own software, which the authors might consider citing (although the approach is straightforward and has likely been independently invented many times).

#### Authors' response

*For similarity searches, we used HHpred, a profile-profile-based method similar to COMPASS, but with the predicted secondary structure in its scoring to obtain better sensitivity. Transitive PSI-BLAST searches were still used since (1) they helped linking most of the groups with statistical support; (2) they enabled exhaustive search for members of the CREST superfamily in the NCBI nonredundant sequence database (nr). No profile-profile based searches on the nr database currently exist as far as we know, possibly due to the time-consuming nature of such searches. We submitted some CREST members to the COMPASS web server http://prodata.swmed.edu/compass. Using the alkaline ceramidase ACER3 from human as a query against the Pfam 23.0 database, COMPASS did identify Hemolysin III (e-value: 8.05e-13) as the third best hit and Per1 (e-value: 2.94e-06) among the top 20 hits. However, most of the top 20 hits with statistically significant e-values (less than 1e-4), such as Herpes_LMP1 and Macoilin, appear to be false positives as they do not possess the conserved motifs unique to the CREST superfamily. A COMPASS search using yeast Per1p as the query also retrieved many false positives as significant hits. These results reflect possible issues with the profiles of membrane proteins on which COMPASS has not been extensively tested. We thus did not include the COMPASS results in the manuscript*.

It would be interesting to compare an explicit phylogenetic tree with the clans output shown in Figure 1. Doug Theobald, now at Brandeis, developed a method for converting COMPASS profile-profile significance scores into a phylogenetic tree (PMID 16266719) which it might be interesting to use here.

#### Authors' response

As the COMPASS results of several CREST members frequently included false positives among the top hits, we chose not to use the method suggested by the reviewer. We instead added the phylogenetic analysis of CREST members by MOLPHY, a frequently used tree reconstruction method based on maximum likelihood. The tree reported by MOLPHY is consistent with the CLANS grouping and provided more information about the positioning of the hypothetical groups relative to the known groups. We added the phylogenetic analysis results in the revised manuscript. We also provided the phylogenetic tree reconstructed by another program PhyML in the supplementary data.

The discussion of the relationships among protein families and conclusions generally seem appropriate, and the manuscript should be a useful contribution to the literature, especially if specific experimental tests could be proposed that would confirm the function of the unknown protein families and if the results could be confirmed with the additional methods suggested above.

#### Authors' response

*In the revised manuscript, we proposed to test the ceramidase activity for hypothetical groups 1 and 2 since they are more closely related to alkaline ceramidases than to the other known groups. Since two of the CREST groups, alkaline ceramidase and Per1, catalyze similar yet distinct reactions on different substrates, we reason that the substrates for the other CREST groups, such as PAQRs, could also differ. We thus proposed in the revised manuscript that experimental studies of PAQRs should not be restricted to just the ceramidase activity. Other potential lipid hydrolase activities should also be considered. Specifically, we proposed in the revised manuscript that the phospholipase A2 activity (previously suggested for Per1) could be tested. The reaction products (a lysophospholipid molecule and a free fatty acid) of the phospholipase A2 activity on a phospholipid substrate can be precursors of second messengers that could function in PAQR signaling*.

## Supplementary Material

Additional file 1 **A list of proteins belonging to the CREST superfamily**. This file contains a list of CREST proteins found in transitive PSI-BLAST searches (sequence fragments with less than 100 amino acids are excluded). They are grouped according to the CLANS clustering results. Click here for file

Additional file 2 **A multiple sequence alignment of CREST domains**. This file contains the alignment of CREST domains covering seven predicted core transmembrane segments for sequences shown in Figure 1. Click here for file

Additional file 3 **Phylogenetic reconstruction by PhyML for representative CREST domains**. This file contains the phylogenetic tree generated by PhyML (version 3.0) (see Methods) for sequences shown in Figure 1. The eleven CREST groups are labeled to the right of the tree. The root of each group is marked by a black circle. Species abbreviations and coloring schemes are the same as those described in the legend to Figure 3. Click here for file
