# Supplementary material for: CREST - a large and diverse superfamily of putative transmembrane hydrolases
Source: Biol Direct. 2011 Jul 6;6:37. doi: 10.1186/1745-6150-6-37 (PMC3146951; doi:10.1186/1745-6150-6-37)
Supplement: Additional file 1 — A list of proteins belonging to the CREST superfamily. This file contains a list of CREST proteins found in transitive PSI-BLAST searches (sequence fragments with less than 100 amino acids are excluded). They are grouped according to the CLANS clustering results. [file 1745-6150-6-37-S1.HTML]

```
## Additional data file 1

Below are CREST superfamily proteins found in transitive PSI-BLAST searches (sequence fragments with PSI-BLAST hits less than 100aa were ignored).
The eleven groups are manually defined using the CLANS clustering results.
The proteins are listed below in the following format:
gi domain_range |organism name|domain|phylum class|definition line   (domain - E: eukaryote; B: bacteria)
```

```
Alkaline ceramidase:
115389580 20..302    |Aspergillus terreus NIH2624                   |E|Fungi Dikarya                      | >gi|115389580|ref|XP_001212295.1| predicted protein [Aspergillus terreus NIH2624]
119184585 18..284    |Coccidioides immitis RS                       |E|Fungi Dikarya                      | >gi|119184585|ref|XP_001243177.1| conserved hypothetical protein [Coccidioides immitis RS]
240109908 18..284    |Coccidioides posadasii C735 delta SOWgp       |E|Fungi Dikarya                      | >gi|240109908|gb|EER28077.1| Alkaline phytoceramidase family protein [Coccidioides posadasii C735 delta SOWgp]
119500094 24..289    |Neosartorya fischeri NRRL 181                 |E|Fungi Dikarya                      | >gi|119500094|ref|XP_001266804.1| Alkaline phytoceramidase, putative [Neosartorya fischeri NRRL 181]
70993502  24..280    |Aspergillus fumigatus Af293                   |E|Fungi Dikarya                      | >gi|70993502|ref|XP_751598.1| alkaline dihydroceramidase Ydc1 [Aspergillus fumigatus Af293]
121708291 24..287    |Aspergillus clavatus NRRL 1                   |E|Fungi Dikarya                      | >gi|121708291|ref|XP_001272086.1| Alkaline phytoceramidase, putative [Aspergillus clavatus NRRL 1]
212542461 25..288    |Penicillium marneffei ATCC 18224              |E|Fungi Dikarya                      | >gi|212542461|ref|XP_002151385.1| alkaline dihydroceramidase Ydc1, putative [Penicillium marneffei ATCC 18224]
242768958 25..288    |Talaromyces stipitatus ATCC 10500             |E|Fungi Dikarya                      | >gi|242768958|ref|XP_002341672.1| alkaline dihydroceramidase Ydc1, putative [Talaromyces stipitatus ATCC 10500]
242768967 25..268    |Talaromyces stipitatus ATCC 10500             |E|Fungi Dikarya                      | >gi|242768967|ref|XP_002341674.1| alkaline dihydroceramidase Ydc1, putative [Talaromyces stipitatus ATCC 10500]
258568756 18..281    |Uncinocarpus reesii 1704                      |E|Fungi Dikarya                      | >gi|258568756|ref|XP_002585122.1| predicted protein [Uncinocarpus reesii 1704]
259481803 22..285    |Aspergillus nidulans FGSC A4                  |E|Fungi Dikarya                      | >gi|259481803|tpe|CBF75665.1| TPA: conserved hypothetical protein similar to alkaline phytoceramidase (Eurofung) [Aspergillus nidulans FGSC A4]
67526409  479..742   |Aspergillus nidulans FGSC A4                  |E|Fungi Dikarya                      | >gi|67526409|ref|XP_661266.1| hypothetical protein AN3662.2 [Aspergillus nidulans FGSC A4]
296816349 48..310    |Arthroderma otae CBS 113480                   |E|Fungi Dikarya                      | >gi|296816349|ref|XP_002848511.1| alkaline ceramidase [Arthroderma otae CBS 113480]
154303985 24..284    |Botryotinia fuckeliana B05.10                 |E|Fungi Dikarya                      | >gi|154303985|ref|XP_001552398.1| alkaline ceramidase [Botryotinia fuckeliana B05.10]
156030629 2..244     |Sclerotinia sclerotiorum 1980 UF-70           |E|Fungi Dikarya                      | >gi|156030629|ref|XP_001584641.1| hypothetical protein SS1G_14410 [Sclerotinia sclerotiorum 1980]
189189684 25..284    |Pyrenophora tritici-repentis Pt-1C-BFP        |E|Fungi Dikarya                      | >gi|189189684|ref|XP_001931181.1| dihydroceramidase [Pyrenophora tritici-repentis Pt-1C-BFP]
295668020 21..278    |Paracoccidioides brasiliensis Pb01            |E|Fungi Dikarya                      | >gi|295668020|ref|XP_002794559.1| alkaline phytoceramidase [Paracoccidioides brasiliensis Pb01]
225679710 21..274    |Paracoccidioides brasiliensis Pb03            |E|Fungi Dikarya                      | >gi|225679710|gb|EEH17994.1| dihydroceramidase [Paracoccidioides brasiliensis Pb03]
226291454 1..181     |Paracoccidioides brasiliensis Pb18            |E|Fungi Dikarya                      | >gi|226291454|gb|EEH46882.1| alkaline phytoceramidase [Paracoccidioides brasiliensis Pb18]
225558905 21..274    |Ajellomyces capsulatus G186AR                 |E|Fungi Dikarya                      | >gi|225558905|gb|EEH07188.1| alkaline ceramidase [Ajellomyces capsulatus G186AR]
154275338 31..233    |Ajellomyces capsulatus NAm1                   |E|Fungi Dikarya                      | >gi|154275338|ref|XP_001538520.1| conserved hypothetical protein [Ajellomyces capsulatus NAm1]
239611110 21..274    |Ajellomyces dermatitidis ER-3                 |E|Fungi Dikarya                      | >gi|239611110|gb|EEQ88097.1| alkaline dihydroceramidase Ydc1 [Ajellomyces dermatitidis ER-3]
261205868 21..274    |Ajellomyces dermatitidis SLH14081             |E|Fungi Dikarya                      | >gi|261205868|ref|XP_002627671.1| alkaline dihydroceramidase Ydc1 [Ajellomyces dermatitidis SLH14081]
255715531 22..275    |Lachancea thermotolerans CBS 6340             |E|Fungi Dikarya                      | >gi|255715531|ref|XP_002554047.1| KLTH0E13112p [Lachancea thermotolerans]
50294424  18..268    |Candida glabrata CBS 138                      |E|Fungi Dikarya                      | >gi|50294424|ref|XP_449623.1| hypothetical protein [Candida glabrata CBS 138]
281206199 33..282    |Polysphondylium pallidum PN500                |E|Amoebozoa Mycetozoa                | >gi|281206199|gb|EFA80388.1| alkaline dihydroceramidase [Polysphondylium pallidum PN500]
66806077  14..260    |Dictyostelium discoideum AX4                  |E|Amoebozoa Mycetozoa                | >gi|66806077|ref|XP_636760.1| alkaline dihydroceramidase [Dictyostelium discoideum AX4]
254577913 21..265    |Zygosaccharomyces rouxii CBS 732              |E|Fungi Dikarya                      | >gi|254577913|ref|XP_002494943.1| ZYRO0A13442p [Zygosaccharomyces rouxii]
50289217  21..264    |Candida glabrata CBS 138                      |E|Fungi Dikarya                      | >gi|50289217|ref|XP_447039.1| hypothetical protein [Candida glabrata CBS 138]
6325170   20..263    |Saccharomyces cerevisiae S288c                |E|Fungi Dikarya                      | >gi|6325170|ref|NP_015238.1| Ydc1p [Saccharomyces cerevisiae S288c]
66825453  13..256    |Dictyostelium discoideum AX4                  |E|Amoebozoa Mycetozoa                | >gi|66825453|ref|XP_646081.1| alkaline dihydroceramidase [Dictyostelium discoideum AX4]
151946571 21..263    |Saccharomyces cerevisiae YJM789               |E|Fungi Dikarya                      | >gi|151946571|gb|EDN64793.1| alkaline ceramidase [Saccharomyces cerevisiae YJM789]
6319660   21..263    |Saccharomyces cerevisiae S288c                |E|Fungi Dikarya                      | >gi|6319660|ref|NP_009742.1| Ypc1p [Saccharomyces cerevisiae S288c]
207347586 1..176     |Saccharomyces cerevisiae AWRI1631             |E|Fungi Dikarya                      | >gi|207347586|gb|EDZ73709.1| YBR183Wp-like protein [Saccharomyces cerevisiae AWRI1631]
156847184 21..263    |Vanderwaltozyma polyspora DSM 70294           |E|Fungi Dikarya                      | >gi|156847184|ref|XP_001646477.1| hypothetical protein Kpol_1048p50 [Vanderwaltozyma polyspora DSM 70294]
50310037  25..265    |Kluyveromyces lactis NRRL Y-1140              |E|Fungi Dikarya                      | >gi|50310037|ref|XP_455032.1| hypothetical protein [Kluyveromyces lactis NRRL Y-1140]
164427589 20..258    |Neurospora crassa OR74A                       |E|Fungi Dikarya                      | >gi|164427589|ref|XP_965356.2| hypothetical protein NCU02969 [Neurospora crassa OR74A]
289621507 1..166     |Sordaria macrospora                           |E|Fungi Dikarya                      | >gi|289621507|emb|CBI52290.1| unnamed protein product [Sordaria macrospora]
170084403 21..259    |Laccaria bicolor S238N-H82                    |E|Fungi Dikarya                      | >gi|170084403|ref|XP_001873425.1| predicted protein [Laccaria bicolor S238N-H82]
39941416  29..267    |Magnaporthe oryzae 70-15                      |E|Fungi Dikarya                      | >gi|39941416|ref|XP_360245.1| hypothetical protein MGG_05619 [Magnaporthe oryzae 70-15]
255947658 22..259    |Penicillium chrysogenum Wisconsin 54-1255     |E|Fungi Dikarya                      | >gi|255947658|ref|XP_002564596.1| Pc22g05630 [Penicillium chrysogenum Wisconsin 54-1255]
156843326 21..257    |Vanderwaltozyma polyspora DSM 70294           |E|Fungi Dikarya                      | >gi|156843326|ref|XP_001644731.1| hypothetical protein Kpol_1024p27 [Vanderwaltozyma polyspora DSM 70294]
171684743 23..259    |Podospora anserina S mat+                     |E|Fungi Dikarya                      | >gi|171684743|ref|XP_001907313.1| hypothetical protein [Podospora anserina S mat+]
145229759 24..259    |Aspergillus niger CBS 513.88                  |E|Fungi Dikarya                      | >gi|145229759|ref|XP_001389188.1| hypothetical protein An01g07640 [Aspergillus niger]
159128415 37..270    |Aspergillus fumigatus A1163                   |E|Fungi Dikarya                      | >gi|159128415|gb|EDP53530.1| conserved hypothetical protein [Aspergillus fumigatus A1163]
146323151 18..242    |Aspergillus fumigatus Af293                   |E|Fungi Dikarya                      | >gi|146323151|ref|XP_748448.2| conserved hypothetical protein [Aspergillus fumigatus Af293]
296414950 19..251    |Tuber melanosporum Mel28                      |E|Fungi Dikarya                      | >gi|296414950|ref|XP_002837158.1| hypothetical protein [Tuber melanosporum Mel28]
67523755  12..243    |Aspergillus nidulans FGSC A4                  |E|Fungi Dikarya                      | >gi|67523755|ref|XP_659937.1| hypothetical protein AN2333.2 [Aspergillus nidulans FGSC A4]
119193666 21..251    |Coccidioides immitis RS                       |E|Fungi Dikarya                      | >gi|119193666|ref|XP_001247439.1| hypothetical protein CIMG_01210 [Coccidioides immitis RS]
240105615 21..251    |Coccidioides posadasii C735 delta SOWgp       |E|Fungi Dikarya                      | >gi|240105615|gb|EER23808.1| Alkaline phytoceramidase family protein [Coccidioides posadasii C735 delta SOWgp]
254569664 19..249    |Pichia pastoris GS115                         |E|Fungi Dikarya                      | >gi|254569664|ref|XP_002491942.1| Alkaline dihydroceramidase, involved in sphingolipid metabolism [Pichia pastoris GS115]
291173371 1..231     |Arthroderma benhamiae CBS 112371              |E|Fungi Dikarya                      | >gi|291173371|gb|EFE29211.1| conserved hypothetical protein [Arthroderma benhamiae CBS 112371]
146417218 21..250    |Meyerozyma guilliermondii ATCC 6260           |E|Fungi Dikarya                      | >gi|146417218|ref|XP_001484578.1| hypothetical protein PGUG_02307 [Meyerozyma guilliermondii ATCC 6260]
190346185 21..250    |Meyerozyma guilliermondii ATCC 6260           |E|Fungi Dikarya                      | >gi|190346185|gb|EDK38209.2| hypothetical protein PGUG_02307 [Meyerozyma guilliermondii ATCC 6260]
149234547 22..251    |Lodderomyces elongisporus NRRL YB-4239        |E|Fungi Dikarya                      | >gi|149234547|ref|XP_001523153.1| hypothetical protein LELG_05699 [Lodderomyces elongisporus NRRL YB-4239]
241955142 21..250    |Candida dubliniensis CD36                     |E|Fungi Dikarya                      | >gi|241955142|ref|XP_002420292.1| alkaline ceramidase, putative [Candida dubliniensis CD36]
68481136  21..250    |Candida albicans SC5314                       |E|Fungi Dikarya                      | >gi|68481136|ref|XP_715483.1| hypothetical protein CaO19.10616 [Candida albicans SC5314]
255729078 21..250    |Candida tropicalis MYA-3404                   |E|Fungi Dikarya                      | >gi|255729078|ref|XP_002549464.1| hypothetical protein CTRG_03761 [Candida tropicalis MYA-3404]
50417188  21..250    |Debaryomyces hansenii CBS767                  |E|Fungi Dikarya                      | >gi|50417188|ref|XP_457637.1| DEHA2B15796p [Debaryomyces hansenii CBS767]
58266212  21..250    |Cryptococcus neoformans var. neoformans JEC21 |E|Fungi Dikarya                      | >gi|58266212|ref|XP_570262.1| ceramidase [Cryptococcus neoformans var. neoformans JEC21]
116192375 23..251    |Chaetomium globosum CBS 148.51                |E|Fungi Dikarya                      | >gi|116192375|ref|XP_001222000.1| hypothetical protein CHGG_05905 [Chaetomium globosum CBS 148.51]
121703325 20..248    |Aspergillus clavatus NRRL 1                   |E|Fungi Dikarya                      | >gi|121703325|ref|XP_001269927.1| alkaline ceramidase family protein [Aspergillus clavatus NRRL 1]
238506705 14..242    |Aspergillus flavus NRRL3357                   |E|Fungi Dikarya                      | >gi|238506705|ref|XP_002384554.1| alkaline phytoceramidase, putative [Aspergillus flavus NRRL3357]
169785807 1..142     |Aspergillus oryzae RIB40                      |E|Fungi Dikarya                      | >gi|169785807|ref|XP_001827364.1| hypothetical protein [Aspergillus oryzae RIB40]
256721952 28..256    |Nectria haematococca mpVI 77-13-4             |E|Fungi Dikarya                      | >gi|256721952|gb|EEU35343.1| hypothetical protein NECHADRAFT_100775 [Nectria haematococca mpVI 77-13-4]
260945919 55..283    |Clavispora lusitaniae ATCC 42720              |E|Fungi Dikarya                      | >gi|260945919|ref|XP_002617257.1| hypothetical protein CLUG_02701 [Clavispora lusitaniae ATCC 42720]
45201295  16..244    |Ashbya gossypii ATCC 10895                    |E|Fungi Dikarya                      | >gi|45201295|ref|NP_986865.1| AGR199Wp [Ashbya gossypii ATCC 10895]
50551185  21..249    |Yarrowia lipolytica CLIB122                   |E|Fungi Dikarya                      | >gi|50551185|ref|XP_503066.1| YALI0D20262p [Yarrowia lipolytica]
115386554 20..247    |Aspergillus terreus NIH2624                   |E|Fungi Dikarya                      | >gi|115386554|ref|XP_001209818.1| predicted protein [Aspergillus terreus NIH2624]
189188988 25..252    |Pyrenophora tritici-repentis Pt-1C-BFP        |E|Fungi Dikarya                      | >gi|189188988|ref|XP_001930833.1| alkaline ceramidase family protein [Pyrenophora tritici-repentis Pt-1C-BFP]
196016027 20..247    |Trichoplax adhaerens                          |E|Metazoa Placozoa                   | >gi|196016027|ref|XP_002117868.1| hypothetical protein TRIADDRAFT_33194 [Trichoplax adhaerens]
256725331 10..237    |Nectria haematococca mpVI 77-13-4             |E|Fungi Dikarya                      | >gi|256725331|gb|EEU38695.1| hypothetical protein NECHADRAFT_62486 [Nectria haematococca mpVI 77-13-4]
46116490  28..255    |Gibberella zeae PH-1                          |E|Fungi Dikarya                      | >gi|46116490|ref|XP_384263.1| hypothetical protein FG04087.1 [Gibberella zeae PH-1]
238034201 22..248    |Pichia pastoris                               |E|Fungi Dikarya                      | >gi|238034201|emb|CAY67042.1| Alkaline ceramidase [Pichia pastoris]
255955659 20..246    |Penicillium chrysogenum Wisconsin 54-1255     |E|Fungi Dikarya                      | >gi|255955659|ref|XP_002568582.1| Pc21g15720 [Penicillium chrysogenum Wisconsin 54-1255]
167525892 15..240    |Monosiga brevicollis MX1                      |E|Choanoflagellida Codonosigidae     | >gi|167525892|ref|XP_001747280.1| hypothetical protein [Monosiga brevicollis MX1]
46127807  10..235    |Gibberella zeae PH-1                          |E|Fungi Dikarya                      | >gi|46127807|ref|XP_388457.1| hypothetical protein FG08281.1 [Gibberella zeae PH-1]
109108005 15..239    |Macaca mulatta                                |E|Metazoa Chordata                   | >gi|109108005|ref|XP_001088478.1| PREDICTED: alkaline ceramidase 3 [Macaca mulatta]
114639456 15..239    |Pan troglodytes                               |E|Metazoa Chordata                   | >gi|114639456|ref|XP_001175032.1| PREDICTED: alkaline ceramidase 3 isoform 3 [Pan troglodytes]
170932467 15..239    |Homo sapiens                                  |E|Metazoa Chordata                   | >gi|170932467|ref|NP_060837.3| alkaline ceramidase 3 [Homo sapiens]
291384224 15..239    |Oryctolagus cuniculus                         |E|Metazoa Chordata                   | >gi|291384224|ref|XP_002708541.1| PREDICTED: phytoceramidase, alkaline [Oryctolagus cuniculus]
296217004 15..239    |Callithrix jacchus                            |E|Metazoa Chordata                   | >gi|296217004|ref|XP_002754847.1| PREDICTED: alkaline ceramidase 3-like isoform 1 [Callithrix jacchus]
73987895  15..239    |Canis lupus familiaris                        |E|Metazoa Chordata                   | >gi|73987895|ref|XP_849760.1| PREDICTED: similar to Alkaline phytoceramidase (aPHC) (Alkaline ceramidase) (Alkaline dihydroceramidase SB89) isoform 2 [Canis familiaris]
194213412 83..283    |Equus caballus                                |E|Metazoa Chordata                   | >gi|194213412|ref|XP_001494798.2| PREDICTED: similar to Alkaline phytoceramidase (aPHC) (Alkaline ceramidase) (Alkaline dihydroceramidase SB89) [Equus caballus]
114639458 1..197     |Pan troglodytes                               |E|Metazoa Chordata                   | >gi|114639458|ref|XP_001175028.1| PREDICTED: similar to alkaline phytoceramidase isoform 1 [Pan troglodytes]
221040710 1..158     |Homo sapiens                                  |E|Metazoa Chordata                   | >gi|221040710|dbj|BAH12032.1| unnamed protein product [Homo sapiens]
297689777 15..140    |Pongo abelii                                  |E|Metazoa Chordata                   | >gi|297689777|ref|XP_002822297.1| PREDICTED: alkaline ceramidase 3-like [Pongo abelii]
126327807 16..240    |Monodelphis domestica                         |E|Metazoa Chordata                   | >gi|126327807|ref|XP_001378015.1| PREDICTED: alkaline ceramidase 3-like [Monodelphis domestica]
12835417  15..239    |Mus musculus                                  |E|Metazoa Chordata                   | >gi|12835417|dbj|BAB23250.1| unnamed protein product [Mus musculus]
293344237 15..239    |Rattus norvegicus                             |E|Metazoa Chordata                   | >gi|293344237|ref|XP_001065019.2| PREDICTED: brain washing-like [Rattus norvegicus]
84794581  15..239    |Mus musculus                                  |E|Metazoa Chordata                   | >gi|84794581|ref|NP_079684.2| alkaline ceramidase 3 [Mus musculus]
12856561  1..197     |Mus musculus                                  |E|Metazoa Chordata                   | >gi|12856561|dbj|BAB30708.1| unnamed protein product [Mus musculus]
148684393 1..197     |Mus musculus                                  |E|Metazoa Chordata                   | >gi|148684393|gb|EDL16340.1| phytoceramidase, alkaline, isoform CRA_e [Mus musculus]
148684391 1..144     |Mus musculus                                  |E|Metazoa Chordata                   | >gi|148684391|gb|EDL16338.1| phytoceramidase, alkaline, isoform CRA_c [Mus musculus]
148231273 15..239    |Xenopus laevis                                |E|Metazoa Chordata                   | >gi|148231273|ref|NP_001088415.1| alkaline ceramidase 3 [Xenopus laevis]
156121215 15..239    |Bos taurus                                    |E|Metazoa Chordata                   | >gi|156121215|ref|NP_001095755.1| alkaline ceramidase 3 [Bos taurus]
73987893  15..165    |Canis lupus familiaris                        |E|Metazoa Chordata                   | >gi|73987893|ref|XP_859655.1| PREDICTED: similar to Alkaline phytoceramidase (aPHC) (Alkaline ceramidase) (Alkaline dihydroceramidase SB89) isoform 3 [Canis familiaris]
163914871 15..239    |Xenopus (Silurana) tropicalis                 |E|Metazoa Chordata                   | >gi|163914871|ref|NP_001106437.1| alkaline ceramidase 3 [Xenopus (Silurana) tropicalis]
169607523 32..256    |Phaeosphaeria nodorum SN15                    |E|Fungi Dikarya                      | >gi|169607523|ref|XP_001797181.1| hypothetical protein SNOG_06820 [Phaeosphaeria nodorum SN15]
221106266 18..242    |Hydra magnipapillata                          |E|Metazoa Cnidaria                   | >gi|221106266|ref|XP_002168363.1| PREDICTED: similar to Alkaline phytoceramidase [Hydra magnipapillata]
260800620 15..239    |Branchiostoma floridae                        |E|Metazoa Chordata                   | >gi|260800620|ref|XP_002595196.1| hypothetical protein BRAFLDRAFT_238691 [Branchiostoma floridae]
292618470 15..239    |Danio rerio                                   |E|Metazoa Chordata                   | >gi|292618470|ref|XP_683002.4| PREDICTED: brain washing-like, partial [Danio rerio]
50731474  15..239    |Gallus gallus                                 |E|Metazoa Chordata                   | >gi|50731474|ref|XP_417279.1| PREDICTED: similar to alkaline phytoceramidase [Gallus gallus]
119180066 33..255    |Coccidioides immitis RS                       |E|Fungi Dikarya                      | >gi|119180066|ref|XP_001241540.1| hypothetical protein CIMG_08703 [Coccidioides immitis RS]
240110259 1..148     |Coccidioides posadasii C735 delta SOWgp       |E|Fungi Dikarya                      | >gi|240110259|gb|EER28418.1| hypothetical protein CPC735_062910 [Coccidioides posadasii C735 delta SOWgp]
262098540 25..247    |Phytophthora infestans T30-4                  |E|stramenopiles Oomycetes            | >gi|262098540|gb|EEY56592.1| alkaline phytoceramidase (aPHC), putative [Phytophthora infestans T30-4]
156402872 9..230     |Nematostella vectensis                        |E|Metazoa Cnidaria                   | >gi|156402872|ref|XP_001639814.1| predicted protein [Nematostella vectensis]
169154956 30..251    |Danio rerio                                   |E|Metazoa Chordata                   | >gi|169154956|emb|CAQ14946.1| novel protein similar to H.sapiens ASAH3, N-acylsphingosine amidohydrolase (alkaline ceramidase) 3 (ASAH3, zgc:110285) [Danio rerio]
62955175  9..230     |Danio rerio                                   |E|Metazoa Chordata                   | >gi|62955175|ref|NP_001017603.1| alkaline ceramidase 1 [Danio rerio]
171695672 28..249    |Podospora anserina S mat+                     |E|Fungi Dikarya                      | >gi|171695672|ref|XP_001912760.1| hypothetical protein [Podospora anserina S mat+]
198438148 13..234    |Ciona intestinalis                            |E|Metazoa Chordata                   | >gi|198438148|ref|XP_002124873.1| PREDICTED: similar to GJ18341 [Ciona intestinalis]
242008228 16..237    |Pediculus humanus corporis                    |E|Metazoa Arthropoda                 | >gi|242008228|ref|XP_002424912.1| Alkaline ceramidase, putative [Pediculus humanus corporis]
109123094 9..229     |Macaca mulatta                                |E|Metazoa Chordata                   | >gi|109123094|ref|XP_001087211.1| PREDICTED: alkaline ceramidase 1 [Macaca mulatta]
114674889 9..229     |Pan troglodytes                               |E|Metazoa Chordata                   | >gi|114674889|ref|XP_524068.2| PREDICTED: alkaline ceramidase 1 [Pan troglodytes]
119589511 9..229     |Homo sapiens                                  |E|Metazoa Chordata                   | >gi|119589511|gb|EAW69105.1| N-acylsphingosine amidohydrolase (alkaline ceramidase) 3, isoform CRA_b [Homo sapiens]
19424128  9..229     |Homo sapiens                                  |E|Metazoa Chordata                   | >gi|19424128|ref|NP_597999.1| alkaline ceramidase 1 [Homo sapiens]
118104043 14..234    |Gallus gallus                                 |E|Metazoa Chordata                   | >gi|118104043|ref|XP_424820.2| PREDICTED: similar to cancer related gene-liver 1 [Gallus gallus]
119579038 14..234    |Homo sapiens                                  |E|Metazoa Chordata                   | >gi|119579038|gb|EAW58634.1| N-acylsphingosine amidohydrolase 3-like [Homo sapiens]
149737015 14..234    |Equus caballus                                |E|Metazoa Chordata                   | >gi|149737015|ref|XP_001496499.1| PREDICTED: similar to N-acylsphingosine amidohydrolase 3-like [Equus caballus]
193784764 14..234    |Homo sapiens                                  |E|Metazoa Chordata                   | >gi|193784764|dbj|BAG53917.1| unnamed protein product [Homo sapiens]
297684360 14..234    |Pongo abelii                                  |E|Metazoa Chordata                   | >gi|297684360|ref|XP_002819810.1| PREDICTED: alkaline ceramidase 2-like [Pongo abelii]
36304156  14..234    |Homo sapiens                                  |E|Metazoa Chordata                   | >gi|36304156|gb|AAQ85132.1| alkaline ceramidase 2 [Homo sapiens]
71043498  14..234    |Homo sapiens                                  |E|Metazoa Chordata                   | >gi|71043498|ref|NP_001010887.2| alkaline ceramidase 2 [Homo sapiens]
114623872 1..185     |Pan troglodytes                               |E|Metazoa Chordata                   | >gi|114623872|ref|XP_001150335.1| PREDICTED: N-acylsphingosine amidohydrolase 3-like isoform 1 [Pan troglodytes]
126333877 107..327   |Monodelphis domestica                         |E|Metazoa Chordata                   | >gi|126333877|ref|XP_001363180.1| PREDICTED: similar to cancer related gene-liver 1 [Monodelphis domestica]
157817955 14..234    |Rattus norvegicus                             |E|Metazoa Chordata                   | >gi|157817955|ref|NP_001101413.1| alkaline ceramidase 2 [Rattus norvegicus]
147905003 14..234    |Xenopus laevis                                |E|Metazoa Chordata                   | >gi|147905003|ref|NP_001090322.1| alkaline ceramidase 2 [Xenopus laevis]
197246691 14..234    |Xenopus (Silurana) tropicalis                 |E|Metazoa Chordata                   | >gi|197246691|gb|AAI68538.1| acer2 protein [Xenopus (Silurana) tropicalis]
62859327  14..234    |Xenopus (Silurana) tropicalis                 |E|Metazoa Chordata                   | >gi|62859327|ref|NP_001017116.1| alkaline ceramidase 2 [Xenopus (Silurana) tropicalis]
134025399 14..168    |Xenopus (Silurana) tropicalis                 |E|Metazoa Chordata                   | >gi|134025399|gb|AAI35346.1| acer2 protein [Xenopus (Silurana) tropicalis]
148706267 19..239    |Mus musculus                                  |E|Metazoa Chordata                   | >gi|148706267|gb|EDL38214.1| N-acylsphingosine amidohydrolase (alkaline ceramidase) 3 [Mus musculus]
28376625  18..238    |Mus musculus                                  |E|Metazoa Chordata                   | >gi|28376625|ref|NP_783858.1| alkaline ceramidase 1 [Mus musculus]
157822473 18..238    |Rattus norvegicus                             |E|Metazoa Chordata                   | >gi|157822473|ref|NP_001100345.1| alkaline ceramidase 1 [Rattus norvegicus]
196002413 8..228     |Trichoplax adhaerens                          |E|Metazoa Placozoa                   | >gi|196002413|ref|XP_002111074.1| hypothetical protein TRIADDRAFT_23096 [Trichoplax adhaerens]
21314858  14..234    |Mus musculus                                  |E|Metazoa Chordata                   | >gi|21314858|ref|NP_647467.1| alkaline ceramidase 2 [Mus musculus]
26351559  14..214    |Mus musculus                                  |E|Metazoa Chordata                   | >gi|26351559|dbj|BAC39416.1| unnamed protein product [Mus musculus]
224043740 64..284    |Taeniopygia guttata                           |E|Metazoa Chordata                   | >gi|224043740|ref|XP_002189935.1| PREDICTED: hypothetical protein [Taeniopygia guttata]
281339764 9..229     |Ailuropoda melanoleuca                        |E|Metazoa Chordata                   | >gi|281339764|gb|EFB15348.1| hypothetical protein PANDA_017638 [Ailuropoda melanoleuca]
281352358 14..234    |Ailuropoda melanoleuca                        |E|Metazoa Chordata                   | >gi|281352358|gb|EFB27942.1| hypothetical protein PANDA_007047 [Ailuropoda melanoleuca]
291383205 14..234    |Oryctolagus cuniculus                         |E|Metazoa Chordata                   | >gi|291383205|ref|XP_002708124.1| PREDICTED: alkaline ceramidase 2 [Oryctolagus cuniculus]
296189987 14..234    |Callithrix jacchus                            |E|Metazoa Chordata                   | >gi|296189987|ref|XP_002743003.1| PREDICTED: alkaline ceramidase 2-like [Callithrix jacchus]
61817829  14..234    |Bos taurus                                    |E|Metazoa Chordata                   | >gi|61817829|ref|XP_612871.1| PREDICTED: alkaline ceramidase 2-like [Bos taurus]
73971696  14..234    |Canis lupus familiaris                        |E|Metazoa Chordata                   | >gi|73971696|ref|XP_538674.2| PREDICTED: similar to alkaline ceramidase 2 [Canis familiaris]
194034352 38..235    |Sus scrofa                                    |E|Metazoa Chordata                   | >gi|194034352|ref|XP_001926946.1| PREDICTED: similar to N-acylsphingosine amidohydrolase 3-like [Sus scrofa]
291233831 11..231    |Saccoglossus kowalevskii                      |E|Metazoa Hemichordata               | >gi|291233831|ref|XP_002736855.1| PREDICTED: alkaline ceramidase 2-like [Saccoglossus kowalevskii]
292616556 13..233    |Danio rerio                                   |E|Metazoa Chordata                   | >gi|292616556|ref|XP_002663077.1| PREDICTED: alkaline ceramidase 2-like [Danio rerio]
296232648 9..229     |Callithrix jacchus                            |E|Metazoa Chordata                   | >gi|296232648|ref|XP_002761667.1| PREDICTED: alkaline ceramidase 1-like [Callithrix jacchus]
73987258  9..229     |Canis lupus familiaris                        |E|Metazoa Chordata                   | >gi|73987258|ref|XP_854540.1| PREDICTED: similar to N-acylsphingosine amidohydrolase 3 [Canis familiaris]
118103137 9..228     |Gallus gallus                                 |E|Metazoa Chordata                   | >gi|118103137|ref|XP_418208.2| PREDICTED: similar to alkaline ceramidase [Gallus gallus]
119473787 1..220     |Neosartorya fischeri NRRL 181                 |E|Fungi Dikarya                      | >gi|119473787|ref|XP_001258769.1| hypothetical protein NFIA_002220 [Neosartorya fischeri NRRL 181]
163914855 10..229    |Xenopus (Silurana) tropicalis                 |E|Metazoa Chordata                   | >gi|163914855|ref|NP_001106432.1| alkaline ceramidase 1 [Xenopus (Silurana) tropicalis]
198430035 16..235    |Ciona intestinalis                            |E|Metazoa Chordata                   | >gi|198430035|ref|XP_002121302.1| PREDICTED: similar to brain washing CG13969-PA [Ciona intestinalis]
198430031 16..195    |Ciona intestinalis                            |E|Metazoa Chordata                   | >gi|198430031|ref|XP_002121151.1| PREDICTED: similar to N-acylsphingosine amidohydrolase 3-like [Ciona intestinalis]
72070145  40..259    |Strongylocentrotus purpuratus                 |E|Metazoa Echinodermata              | >gi|72070145|ref|XP_799207.1| PREDICTED: similar to alkaline ceramidase 2 [Strongylocentrotus purpuratus]
119474313 39..257    |Neosartorya fischeri NRRL 181                 |E|Fungi Dikarya                      | >gi|119474313|ref|XP_001259032.1| alkaline phytoceramidase, putative [Neosartorya fischeri NRRL 181]
159128224 12..209    |Aspergillus fumigatus A1163                   |E|Fungi Dikarya                      | >gi|159128224|gb|EDP53339.1| alkaline ceramidase, putative [Aspergillus fumigatus A1163]
126139379 21..239    |Scheffersomyces stipitis CBS 6054             |E|Fungi Dikarya                      | >gi|126139379|ref|XP_001386212.1| hypothetical protein PICST_63351 [Scheffersomyces stipitis CBS 6054]
156545442 9..227     |Nasonia vitripennis                           |E|Metazoa Arthropoda                 | >gi|156545442|ref|XP_001606736.1| PREDICTED: similar to GA12666-PA [Nasonia vitripennis]
157121017 10..228    |Aedes aegypti                                 |E|Metazoa Arthropoda                 | >gi|157121017|ref|XP_001653733.1| alkaline ceramidase [Aedes aegypti]
170029208 10..228    |Culex quinquefasciatus                        |E|Metazoa Arthropoda                 | >gi|170029208|ref|XP_001842485.1| alkaline ceramidase [Culex quinquefasciatus]
18028135  24..242    |Drosophila melanogaster                       |E|Metazoa Arthropoda                 | >gi|18028135|gb|AAL55991.1|AF323976_1 brain washing [Drosophila melanogaster]
194760394 25..243    |Drosophila ananassae                          |E|Metazoa Arthropoda                 | >gi|194760394|ref|XP_001962426.1| GF14446 [Drosophila ananassae]
194879152 24..242    |Drosophila erecta                             |E|Metazoa Arthropoda                 | >gi|194879152|ref|XP_001974185.1| GG21218 [Drosophila erecta]
195345260 24..242    |Drosophila sechellia                          |E|Metazoa Arthropoda                 | >gi|195345260|ref|XP_002039188.1| GM17388 [Drosophila sechellia]
198472900 32..250    |Drosophila pseudoobscura pseudoobscura        |E|Metazoa Arthropoda                 | >gi|198472900|ref|XP_001356108.2| GA12666 [Drosophila pseudoobscura pseudoobscura]
19921574  24..242    |Drosophila melanogaster                       |E|Metazoa Arthropoda                 | >gi|19921574|ref|NP_610020.1| brain washing [Drosophila melanogaster]
195580322 24..178    |Drosophila simulans                           |E|Metazoa Arthropoda                 | >gi|195580322|ref|XP_002079999.1| GD24242 [Drosophila simulans]
195161779 32..164    |Drosophila persimilis                         |E|Metazoa Arthropoda                 | >gi|195161779|ref|XP_002021739.1| GL26347 [Drosophila persimilis]
195051660 19..237    |Drosophila grimshawi                          |E|Metazoa Arthropoda                 | >gi|195051660|ref|XP_001993144.1| GH13657 [Drosophila grimshawi]
195115872 19..237    |Drosophila mojavensis                         |E|Metazoa Arthropoda                 | >gi|195115872|ref|XP_002002480.1| GI17409 [Drosophila mojavensis]
195398419 19..237    |Drosophila virilis                            |E|Metazoa Arthropoda                 | >gi|195398419|ref|XP_002057819.1| GJ18341 [Drosophila virilis]
195443378 31..249    |Drosophila willistoni                         |E|Metazoa Arthropoda                 | >gi|195443378|ref|XP_002069394.1| GK18730 [Drosophila willistoni]
289724777 14..232    |Glossina morsitans morsitans                  |E|Metazoa Arthropoda                 | >gi|289724777|gb|ADD18339.1| alkaline ceramidase [Glossina morsitans morsitans]
58386547  10..228    |Anopheles gambiae str. PEST                   |E|Metazoa Arthropoda                 | >gi|58386547|ref|XP_314841.2| AGAP008729-PA [Anopheles gambiae str. PEST]
66501023  9..227     |Apis mellifera                                |E|Metazoa Arthropoda                 | >gi|66501023|ref|XP_623814.1| PREDICTED: similar to brain washing CG13969-PA [Apis mellifera]
115454765 14..231    |Oryza sativa Japonica Group                   |E|Viridiplantae Streptophyta         | >gi|115454765|ref|NP_001050983.1| Os03g0698900 [Oryza sativa Japonica Group]
218193581 14..231    |Oryza sativa Indica Group                     |E|Viridiplantae Streptophyta         | >gi|218193581|gb|EEC76008.1| hypothetical protein OsI_13149 [Oryza sativa Indica Group]
126323276 9..226     |Monodelphis domestica                         |E|Metazoa Chordata                   | >gi|126323276|ref|XP_001376492.1| PREDICTED: alkaline ceramidase 1-like [Monodelphis domestica]
18415901  13..230    |Arabidopsis thaliana                          |E|Viridiplantae Streptophyta         | >gi|18415901|ref|NP_567660.1| dihydroceramidase [Arabidopsis thaliana]
297803888 13..230    |Arabidopsis lyrata subsp. lyrata              |E|Viridiplantae Streptophyta         | >gi|297803888|ref|XP_002869828.1| ATCES1 [Arabidopsis lyrata subsp. lyrata]
2832683   150..361   |Arabidopsis thaliana                          |E|Viridiplantae Streptophyta         | >gi|2832683|emb|CAA16783.1| putative protein [Arabidopsis thaliana]
198414485 13..230    |Ciona intestinalis                            |E|Metazoa Chordata                   | >gi|198414485|ref|XP_002121592.1| PREDICTED: similar to N-acylsphingosine amidohydrolase 3-like [Ciona intestinalis]
217073384 13..230    |Medicago truncatula                           |E|Viridiplantae Streptophyta         | >gi|217073384|gb|ACJ85051.1| unknown [Medicago truncatula]
87162688  13..230    |Medicago truncatula                           |E|Viridiplantae Streptophyta         | >gi|87162688|gb|ABD28483.1| Alkaline phytoceramidase [Medicago truncatula]
217070982 4..152     |Medicago truncatula                           |E|Viridiplantae Streptophyta         | >gi|217070982|gb|ACJ83851.1| unknown [Medicago truncatula]
87241163  1..145     |Medicago truncatula                           |E|Viridiplantae Streptophyta         | >gi|87241163|gb|ABD33021.1| Alkaline phytoceramidase [Medicago truncatula]
219115157 104..321   |Phaeodactylum tricornutum CCAP 1055/1         |E|stramenopiles Bacillariophyta      | >gi|219115157|ref|XP_002178374.1| predicted protein [Phaeodactylum tricornutum CCAP 1055/1]
224031831 14..231    |Zea mays                                      |E|Viridiplantae Streptophyta         | >gi|224031831|gb|ACN34991.1| unknown [Zea mays]
224031893 14..231    |Zea mays                                      |E|Viridiplantae Streptophyta         | >gi|224031893|gb|ACN35022.1| unknown [Zea mays]
226497810 14..231    |Zea mays                                      |E|Viridiplantae Streptophyta         | >gi|226497810|ref|NP_001141321.1| hypothetical protein LOC100273412 [Zea mays]
226507238 14..231    |Zea mays                                      |E|Viridiplantae Streptophyta         | >gi|226507238|ref|NP_001140999.1| hypothetical protein LOC100273078 [Zea mays]
242033295 14..231    |Sorghum bicolor                               |E|Viridiplantae Streptophyta         | >gi|242033295|ref|XP_002464042.1| hypothetical protein SORBIDRAFT_01g011140 [Sorghum bicolor]
224028703 1..137     |Zea mays                                      |E|Viridiplantae Streptophyta         | >gi|224028703|gb|ACN33427.1| unknown [Zea mays]
224113865 14..231    |Populus trichocarpa                           |E|Viridiplantae Streptophyta         | >gi|224113865|ref|XP_002316596.1| predicted protein [Populus trichocarpa]
224147238 14..231    |Populus trichocarpa                           |E|Viridiplantae Streptophyta         | >gi|224147238|ref|XP_002336433.1| predicted protein [Populus trichocarpa]
224118004 14..231    |Populus trichocarpa                           |E|Viridiplantae Streptophyta         | >gi|224118004|ref|XP_002331534.1| predicted protein [Populus trichocarpa]
255554723 13..230    |Ricinus communis                              |E|Viridiplantae Streptophyta         | >gi|255554723|ref|XP_002518399.1| alkaline phytoceramidase, putative [Ricinus communis]
260829743 13..229    |Branchiostoma floridae                        |E|Metazoa Chordata                   | >gi|260829743|ref|XP_002609821.1| hypothetical protein BRAFLDRAFT_280354 [Branchiostoma floridae]
110832730 18..233    |Caenorhabditis briggsae                       |E|Metazoa Nematoda                   | >gi|110832730|sp|Q60WT2.2|ACASE_CAEBR RecName: Full=Alkaline ceramidase; Short=AlkCDase; AltName: Full=Alkaline N-acylsphingosine amidohydrolase; AltName: Full=Alkaline acylsphingosine deacylase
268558634 18..233    |Caenorhabditis briggsae                       |E|Metazoa Nematoda                   | >gi|268558634|ref|XP_002637308.1| Hypothetical protein CBG18997 [Caenorhabditis briggsae]
32567035  18..233    |Caenorhabditis elegans                        |E|Metazoa Nematoda                   | >gi|32567035|ref|NP_504697.2| hypothetical protein W02F12.2 [Caenorhabditis elegans]
47217371  13..228    |Tetraodon nigroviridis                        |E|Metazoa Chordata                   | >gi|47217371|emb|CAG00731.1| unnamed protein product [Tetraodon nigroviridis]
169845026 22..236    |Coprinopsis cinerea okayama7#130              |E|Fungi Dikarya                      | >gi|169845026|ref|XP_001829233.1| hypothetical protein CC1G_06570 [Coprinopsis cinerea okayama7#130]
298411613 22..221    |Coprinopsis cinerea okayama7#130              |E|Fungi Dikarya                      | >gi|298411613|gb|EAU92559.2| phytoceramidase [Coprinopsis cinerea okayama7#130]
11359534  20..233    |Neurospora crassa                             |E|Fungi Dikarya                      | >gi|11359534|pir||T50986 hypothetical protein B7F18.50 [imported] - Neurospora crassa
168020061 22..235    |Physcomitrella patens subsp. patens           |E|Viridiplantae Streptophyta         | >gi|168020061|ref|XP_001762562.1| predicted protein [Physcomitrella patens subsp. patens]
168024408 23..236    |Physcomitrella patens subsp. patens           |E|Viridiplantae Streptophyta         | >gi|168024408|ref|XP_001764728.1| predicted protein [Physcomitrella patens subsp. patens]
168045476 23..236    |Physcomitrella patens subsp. patens           |E|Viridiplantae Streptophyta         | >gi|168045476|ref|XP_001775203.1| predicted protein [Physcomitrella patens subsp. patens]
168066594 22..235    |Physcomitrella patens subsp. patens           |E|Viridiplantae Streptophyta         | >gi|168066594|ref|XP_001785220.1| predicted protein [Physcomitrella patens subsp. patens]
224055853 16..229    |Populus trichocarpa                           |E|Viridiplantae Streptophyta         | >gi|224055853|ref|XP_002298686.1| predicted protein [Populus trichocarpa]
225467755 17..230    |Vitis vinifera                                |E|Viridiplantae Streptophyta         | >gi|225467755|ref|XP_002265752.1| PREDICTED: hypothetical protein [Vitis vinifera]
255638051 19..230    |Glycine max                                   |E|Viridiplantae Streptophyta         | >gi|255638051|gb|ACU19340.1| unknown [Glycine max]
255639818 19..230    |Glycine max                                   |E|Viridiplantae Streptophyta         | >gi|255639818|gb|ACU20202.1| unknown [Glycine max]
193690524 16..225    |Acyrthosiphon pisum                           |E|Metazoa Arthropoda                 | >gi|193690524|ref|XP_001952860.1| PREDICTED: similar to LOC495272 protein [Acyrthosiphon pisum]
145229589 101..309   |Aspergillus niger CBS 513.88                  |E|Fungi Dikarya                      | >gi|145229589|ref|XP_001389103.1| hypothetical protein An01g06800 [Aspergillus niger]
240281825 253..461   |Ajellomyces capsulatus H143                   |E|Fungi Dikarya                      | >gi|240281825|gb|EER45328.1| alkaline ceramidase [Ajellomyces capsulatus H143]
115384426 31..234    |Aspergillus terreus NIH2624                   |E|Fungi Dikarya                      | >gi|115384426|ref|XP_001208760.1| conserved hypothetical protein [Aspergillus terreus NIH2624]
47205329  1..204     |Tetraodon nigroviridis                        |E|Metazoa Chordata                   | >gi|47205329|emb|CAF91013.1| unnamed protein product [Tetraodon nigroviridis]
164657181 1..199     |Malassezia globosa CBS 7966                   |E|Fungi Dikarya                      | >gi|164657181|ref|XP_001729717.1| hypothetical protein MGL_3261 [Malassezia globosa CBS 7966]
225433590 7..205     |Vitis vinifera                                |E|Viridiplantae Streptophyta         | >gi|225433590|ref|XP_002269882.1| PREDICTED: hypothetical protein [Vitis vinifera]
47214752  3..201     |Tetraodon nigroviridis                        |E|Metazoa Chordata                   | >gi|47214752|emb|CAG01287.1| unnamed protein product [Tetraodon nigroviridis]
156055426 11..208    |Sclerotinia sclerotiorum 1980 UF-70           |E|Fungi Dikarya                      | >gi|156055426|ref|XP_001593637.1| hypothetical protein SS1G_05065 [Sclerotinia sclerotiorum 1980]
255554034 17..209    |Ricinus communis                              |E|Viridiplantae Streptophyta         | >gi|255554034|ref|XP_002518057.1| alkaline phytoceramidase, putative [Ricinus communis]
116205359 28..219    |Chaetomium globosum CBS 148.51                |E|Fungi Dikarya                      | >gi|116205359|ref|XP_001228490.1| hypothetical protein CHGG_10563 [Chaetomium globosum CBS 148.51]
238503878 1..191     |Aspergillus flavus NRRL3357                   |E|Fungi Dikarya                      | >gi|238503878|ref|XP_002383171.1| alkaline dihydroceramidase Ydc1, putative [Aspergillus flavus NRRL3357]
221040462 15..202    |Homo sapiens                                  |E|Metazoa Chordata                   | >gi|221040462|dbj|BAH11938.1| unnamed protein product [Homo sapiens]
296217006 15..202    |Callithrix jacchus                            |E|Metazoa Chordata                   | >gi|296217006|ref|XP_002754848.1| PREDICTED: alkaline ceramidase 3-like isoform 2 [Callithrix jacchus]
119595418 1..165     |Homo sapiens                                  |E|Metazoa Chordata                   | >gi|119595418|gb|EAW75012.1| phytoceramidase, alkaline, isoform CRA_c [Homo sapiens]
221040622 1..144     |Homo sapiens                                  |E|Metazoa Chordata                   | >gi|221040622|dbj|BAH11988.1| unnamed protein product [Homo sapiens]
221119897 18..204    |Hydra magnipapillata                          |E|Metazoa Cnidaria                   | >gi|221119897|ref|XP_002157790.1| PREDICTED: similar to Alkaline phytoceramidase [Hydra magnipapillata]
262098539 14..199    |Phytophthora infestans T30-4                  |E|stramenopiles Oomycetes            | >gi|262098539|gb|EEY56591.1| alkaline phytoceramidase (aPHC), putative [Phytophthora infestans T30-4]
169764855 1..185     |Aspergillus oryzae RIB40                      |E|Fungi Dikarya                      | >gi|169764855|ref|XP_001816899.1| hypothetical protein [Aspergillus oryzae RIB40]
115377515 2..183     |Stigmatella aurantiaca DW4/3-1                |B|Proteobacteria Deltaproteobacteria | >gi|115377515|ref|ZP_01464715.1| alkaline phytoceramidase [Stigmatella aurantiaca DW4/3-1]
261361668 3..182     |Verticillium albo-atrum VaMs.102              |E|Fungi Dikarya                      | >gi|261361668|gb|EEY24096.1| dihydroceramidase [Verticillium albo-atrum VaMs.102]
156039245 1..179     |Sclerotinia sclerotiorum 1980 UF-70           |E|Fungi Dikarya                      | >gi|156039245|ref|XP_001586730.1| hypothetical protein SS1G_11759 [Sclerotinia sclerotiorum 1980]
46134105  335..510   |Gibberella zeae PH-1                          |E|Fungi Dikarya                      | >gi|46134105|ref|XP_389368.1| hypothetical protein FG09192.1 [Gibberella zeae PH-1]
149016885 14..188    |Rattus norvegicus                             |E|Metazoa Chordata                   | >gi|149016885|gb|EDL76007.1| N-acylsphingosine amidohydrolase 3-like (predicted), isoform CRA_b [Rattus norvegicus]
37590520  14..188    |Mus musculus                                  |E|Metazoa Chordata                   | >gi|37590520|gb|AAH59819.1| Acer2 protein [Mus musculus]
224087519 21..195    |Taeniopygia guttata                           |E|Metazoa Chordata                   | >gi|224087519|ref|XP_002192116.1| PREDICTED: similar to N-acylsphingosine amidohydrolase (alkaline ceramidase) 3 [Taeniopygia guttata]
198430033 16..187    |Ciona intestinalis                            |E|Metazoa Chordata                   | >gi|198430033|ref|XP_002121227.1| PREDICTED: similar to Alkaline ceramidase 2 (AlkCDase 2) (N-acylsphingosine amidohydrolase 3-like) (Acylsphingosine deacylase 3-like) [Ciona intestinalis]
242813112 1..167     |Talaromyces stipitatus ATCC 10500             |E|Fungi Dikarya                      | >gi|242813112|ref|XP_002486100.1| conserved hypothetical protein [Talaromyces stipitatus ATCC 10500]
297703253 4..165     |Pongo abelii                                  |E|Metazoa Chordata                   | >gi|297703253|ref|XP_002828562.1| PREDICTED: alkaline ceramidase 1-like [Pongo abelii]
149068894 15..169    |Rattus norvegicus                             |E|Metazoa Chordata                   | >gi|149068894|gb|EDM18446.1| similar to Alkaline phytoceramidase (aPHC) (Alkaline ceramidase) (predicted), isoform CRA_a [Rattus norvegicus]
23274052  15..169    |Mus musculus                                  |E|Metazoa Chordata                   | >gi|23274052|gb|AAH23924.1| Acer3 protein [Mus musculus]
154302591 40..194    |Botryotinia fuckeliana B05.10                 |E|Fungi Dikarya                      | >gi|154302591|ref|XP_001551705.1| hypothetical protein BC1G_09872 [Botryotinia fuckeliana B05.10]
258574895 21..175    |Uncinocarpus reesii 1704                      |E|Fungi Dikarya                      | >gi|258574895|ref|XP_002541629.1| predicted protein [Uncinocarpus reesii 1704]
281210857 21..173    |Polysphondylium pallidum PN500                |E|Amoebozoa Mycetozoa                | >gi|281210857|gb|EFA85023.1| alkaline dihydroceramidase [Polysphondylium pallidum PN500]
145251059 24..175    |Aspergillus niger CBS 513.88                  |E|Fungi Dikarya                      | >gi|145251059|ref|XP_001397043.1| hypothetical protein An15g05120 [Aspergillus niger]
198434230 12..159    |Ciona intestinalis                            |E|Metazoa Chordata                   | >gi|198434230|ref|XP_002131503.1| PREDICTED: similar to GF14446 [Ciona intestinalis]
149068896 1..144     |Rattus norvegicus                             |E|Metazoa Chordata                   | >gi|149068896|gb|EDM18448.1| similar to Alkaline phytoceramidase (aPHC) (Alkaline ceramidase) (predicted), isoform CRA_c [Rattus norvegicus]
149609280 20..156    |Ornithorhynchus anatinus                      |E|Metazoa Chordata                   | >gi|149609280|ref|XP_001519806.1| PREDICTED: similar to N-acylsphingosine amidohydrolase 3-like [Ornithorhynchus anatinus]
169607469 8..144     |Phaeosphaeria nodorum SN15                    |E|Fungi Dikarya                      | >gi|169607469|ref|XP_001797154.1| hypothetical protein SNOG_06792 [Phaeosphaeria nodorum SN15]
224129128 1..133     |Populus trichocarpa                           |E|Viridiplantae Streptophyta         | >gi|224129128|ref|XP_002328897.1| predicted protein [Populus trichocarpa]
258577975 1..129     |Uncinocarpus reesii 1704                      |E|Fungi Dikarya                      | >gi|258577975|ref|XP_002543169.1| predicted protein [Uncinocarpus reesii 1704]
281347682 1..127     |Ailuropoda melanoleuca                        |E|Metazoa Chordata                   | >gi|281347682|gb|EFB23266.1| hypothetical protein PANDA_020026 [Ailuropoda melanoleuca]
33341740  1..127     |Homo sapiens                                  |E|Metazoa Chordata                   | >gi|33341740|gb|AAQ15241.1|AF370405_1 PP11646 [Homo sapiens]
154309961 1..124     |Botryotinia fuckeliana B05.10                 |E|Fungi Dikarya                      | >gi|154309961|ref|XP_001554313.1| hypothetical protein BC1G_06901 [Botryotinia fuckeliana B05.10]
170590256 42..155    |Brugia malayi                                 |E|Metazoa Nematoda                   | >gi|170590256|ref|XP_001899888.1| cancer related gene-liver 1 [Brugia malayi]
157074026 9..119     |Bos taurus                                    |E|Metazoa Chordata                   | >gi|157074026|ref|NP_001096724.1| alkaline ceramidase 1 [Bos taurus]
194212520 9..118     |Equus caballus                                |E|Metazoa Chordata                   | >gi|194212520|ref|XP_001916884.1| PREDICTED: similar to N-acylsphingosine amidohydrolase 3 [Equus caballus]
291001573 29..262    |Naegleria gruberi strain NEG-M                |E|Heterolobosea Schizopyrenida       | >gi|291001573|ref|XP_002683353.1| predicted protein [Naegleria gruberi]

```
PAQR group 1:
18377749  66..345    |Arabidopsis thaliana                          |E|Viridiplantae Streptophyta         | >gi|18377749|gb|AAL67024.1| unknown protein [Arabidopsis thaliana]
297798884 68..347    |Arabidopsis lyrata subsp. lyrata              |E|Viridiplantae Streptophyta         | >gi|297798884|ref|XP_002867326.1| hypothetical protein ARALYDRAFT_491666 [Arabidopsis lyrata subsp. lyrata]
30688829  66..345    |Arabidopsis thaliana                          |E|Viridiplantae Streptophyta         | >gi|30688829|ref|NP_194814.2| heptahelical transmembrane protein2 [Arabidopsis thaliana]
50311505  335..612   |Kluyveromyces lactis NRRL Y-1140              |E|Fungi Dikarya                      | >gi|50311505|ref|XP_455777.1| hypothetical protein [Kluyveromyces lactis NRRL Y-1140]
226458629 40..316    |Micromonas pusilla CCMP1545                   |E|Viridiplantae Chlorophyta          | >gi|226458629|gb|EEH55926.1| predicted protein [Micromonas pusilla CCMP1545]
156847996 287..562   |Vanderwaltozyma polyspora DSM 70294           |E|Fungi Dikarya                      | >gi|156847996|ref|XP_001646881.1| hypothetical protein Kpol_2002p94 [Vanderwaltozyma polyspora DSM 70294]
224142341 56..331    |Populus trichocarpa                           |E|Viridiplantae Streptophyta         | >gi|224142341|ref|XP_002324517.1| predicted protein [Populus trichocarpa]
50286265  250..524   |Candida glabrata CBS 138                      |E|Fungi Dikarya                      | >gi|50286265|ref|XP_445561.1| hypothetical protein [Candida glabrata CBS 138]
212275953 91..363    |Zea mays                                      |E|Viridiplantae Streptophyta         | >gi|212275953|ref|NP_001130583.1| hypothetical protein LOC100191682 [Zea mays]
195641232 89..358    |Zea mays                                      |E|Viridiplantae Streptophyta         | >gi|195641232|gb|ACG40084.1| hemolysin-III related family protein [Zea mays]
68442915  35..307    |Danio rerio                                   |E|Metazoa Chordata                   | >gi|68442915|ref|XP_685691.1| PREDICTED: progestin and adipoQ receptor family member VIII-like [Danio rerio]
108706691 95..366    |Oryza sativa Japonica Group                   |E|Viridiplantae Streptophyta         | >gi|108706691|gb|ABF94486.1| Haemolysin-III related family protein, expressed [Oryza sativa Japonica Group]
242096494 93..363    |Sorghum bicolor                               |E|Viridiplantae Streptophyta         | >gi|242096494|ref|XP_002438737.1| hypothetical protein SORBIDRAFT_10g025230 [Sorghum bicolor]
254568404 212..482   |Pichia pastoris GS115                         |E|Fungi Dikarya                      | >gi|254568404|ref|XP_002491312.1| Membrane protein involved in zinc metabolism, member of the four-protein IZH family [Pichia pastoris GS115]
144925078 83..351    |Medicago truncatula                           |E|Viridiplantae Streptophyta         | >gi|144925078|gb|ABP03876.1| Hly-III related proteins [Medicago truncatula]
217073582 83..265    |Medicago truncatula                           |E|Viridiplantae Streptophyta         | >gi|217073582|gb|ACJ85151.1| unknown [Medicago truncatula]
255634953 53..321    |Glycine max                                   |E|Viridiplantae Streptophyta         | >gi|255634953|gb|ACU17835.1| unknown [Glycine max]
290975978 90..358    |Naegleria gruberi strain NEG-M                |E|Heterolobosea Schizopyrenida       | >gi|290975978|ref|XP_002670718.1| predicted protein [Naegleria gruberi]
218198668 99..365    |Oryza sativa Indica Group                     |E|Viridiplantae Streptophyta         | >gi|218198668|gb|EEC81095.1| hypothetical protein OsI_23936 [Oryza sativa Indica Group]
297606284 1..173     |Oryza sativa Japonica Group                   |E|Viridiplantae Streptophyta         | >gi|297606284|ref|NP_001058233.2| Os06g0652200 [Oryza sativa Japonica Group]
125538331 87..352    |Oryza sativa Indica Group                     |E|Viridiplantae Streptophyta         | >gi|125538331|gb|EAY84726.1| hypothetical protein OsI_06094 [Oryza sativa Indica Group]
125581034 76..311    |Oryza sativa Japonica Group                   |E|Viridiplantae Streptophyta         | >gi|125581034|gb|EAZ21965.1| hypothetical protein OsJ_05618 [Oryza sativa Japonica Group]
115444609 22..211    |Oryza sativa Japonica Group                   |E|Viridiplantae Streptophyta         | >gi|115444609|ref|NP_001046084.1| Os02g0179500 [Oryza sativa Japonica Group]
45184671  142..405   |Ashbya gossypii ATCC 10895                    |E|Fungi Dikarya                      | >gi|45184671|ref|NP_982389.1| AAL153Cp [Ashbya gossypii ATCC 10895]
13272399  57..319    |Arabidopsis thaliana                          |E|Viridiplantae Streptophyta         | >gi|13272399|gb|AAK17138.1|AF325070_1 hypothetical protein [Arabidopsis thaliana]
18400468  69..331    |Arabidopsis thaliana                          |E|Viridiplantae Streptophyta         | >gi|18400468|ref|NP_565564.1| heptahelical protein 3 [Arabidopsis thaliana]
297825335 69..331    |Arabidopsis lyrata subsp. lyrata              |E|Viridiplantae Streptophyta         | >gi|297825335|ref|XP_002880550.1| hypothetical protein ARALYDRAFT_481271 [Arabidopsis lyrata subsp. lyrata]
147805376 27..285    |Vitis vinifera                                |E|Viridiplantae Streptophyta         | >gi|147805376|emb|CAN76362.1| hypothetical protein VITISV_035439 [Vitis vinifera]
225446239 180..374   |Vitis vinifera                                |E|Viridiplantae Streptophyta         | >gi|225446239|ref|XP_002263728.1| PREDICTED: hypothetical protein [Vitis vinifera]
296084517 180..374   |Vitis vinifera                                |E|Viridiplantae Streptophyta         | >gi|296084517|emb|CBI25538.3| unnamed protein product [Vitis vinifera]
195630112 76..333    |Zea mays                                      |E|Viridiplantae Streptophyta         | >gi|195630112|gb|ACG36618.1| hypothetical protein [Zea mays]
212275726 76..333    |Zea mays                                      |E|Viridiplantae Streptophyta         | >gi|212275726|ref|NP_001130249.1| hypothetical protein LOC100191343 [Zea mays]
242060738 70..263    |Sorghum bicolor                               |E|Viridiplantae Streptophyta         | >gi|242060738|ref|XP_002451658.1| hypothetical protein SORBIDRAFT_04g005400 [Sorghum bicolor]
2980781   66..322    |Arabidopsis thaliana                          |E|Viridiplantae Streptophyta         | >gi|2980781|emb|CAA18208.1| putative protein [Arabidopsis thaliana]
47211444  857..1112  |Tetraodon nigroviridis                        |E|Metazoa Chordata                   | >gi|47211444|emb|CAF93696.1| unnamed protein product [Tetraodon nigroviridis]
226509848 72..326    |Zea mays                                      |E|Viridiplantae Streptophyta         | >gi|226509848|ref|NP_001143779.1| hypothetical protein LOC100276544 [Zea mays]
126338262 78..328    |Monodelphis domestica                         |E|Metazoa Chordata                   | >gi|126338262|ref|XP_001372276.1| PREDICTED: progestin and adipoQ receptor family member 9-like [Monodelphis domestica]
116089314 78..327    |Mus musculus                                  |E|Metazoa Chordata                   | >gi|116089314|ref|NP_940806.2| progestin and adipoQ receptor family member 9 [Mus musculus]
293349376 38..287    |Rattus norvegicus                             |E|Metazoa Chordata                   | >gi|293349376|ref|XP_001068629.2| PREDICTED: progestin and adipoQ receptor family member IX, partial [Rattus norvegicus]
293361283 55..304    |Rattus norvegicus                             |E|Metazoa Chordata                   | >gi|293361283|ref|XP_236503.5| PREDICTED: progestin and adipoQ receptor family member IX, partial [Rattus norvegicus]
297672165 80..329    |Pongo abelii                                  |E|Metazoa Chordata                   | >gi|297672165|ref|XP_002814179.1| PREDICTED: progestin and adipoQ receptor family member 9-like isoform 1 [Pongo abelii]
297672167 80..329    |Pongo abelii                                  |E|Metazoa Chordata                   | >gi|297672167|ref|XP_002814180.1| PREDICTED: progestin and adipoQ receptor family member 9-like isoform 2 [Pongo abelii]
38348324  80..329    |Homo sapiens                                  |E|Metazoa Chordata                   | >gi|38348324|ref|NP_940906.1| progestin and adipoQ receptor family member 9 [Homo sapiens]
51701770  78..327    |Mus musculus                                  |E|Metazoa Chordata                   | >gi|51701770|sp|Q6TCG2.1|PAQR9_MOUSE RecName: Full=Progestin and adipoQ receptor family member 9; AltName: Full=Progestin and adipoQ receptor family member IX
291399917 142..392   |Oryctolagus cuniculus                         |E|Metazoa Chordata                   | >gi|291399917|ref|XP_002716638.1| PREDICTED: progestin and adipoQ receptor family member IX-like [Oryctolagus cuniculus]
109048956 80..329    |Macaca mulatta                                |E|Metazoa Chordata                   | >gi|109048956|ref|XP_001111860.1| PREDICTED: progestin and adipoQ receptor family member 9-like [Macaca mulatta]
118150996 80..329    |Bos taurus                                    |E|Metazoa Chordata                   | >gi|118150996|ref|NP_001071419.1| progestin and adipoQ receptor family member 9 [Bos taurus]
296227906 332..581   |Callithrix jacchus                            |E|Metazoa Chordata                   | >gi|296227906|ref|XP_002807709.1| PREDICTED: LOW QUALITY PROTEIN: progestin and adipoQ receptor family member 9-like [Callithrix jacchus]
73990669  80..329    |Canis lupus familiaris                        |E|Metazoa Chordata                   | >gi|73990669|ref|XP_542820.2| PREDICTED: similar to progestin and adipoQ receptor family member IX [Canis familiaris]
154346510 105..354   |Leishmania braziliensis MHOM/BR/75/M2904      |E|Euglenozoa Kinetoplastida          | >gi|154346510|ref|XP_001569192.1| hypothetical protein [Leishmania braziliensis MHOM/BR/75/M2904]
154346502 105..236   |Leishmania braziliensis MHOM/BR/75/M2904      |E|Euglenozoa Kinetoplastida          | >gi|154346502|ref|XP_001569188.1| hypothetical protein [Leishmania braziliensis MHOM/BR/75/M2904]
146104884 105..352   |Leishmania infantum JPCM5                     |E|Euglenozoa Kinetoplastida          | >gi|146104884|ref|XP_001469933.1| hypothetical protein [Leishmania infantum]
157877645 105..350   |Leishmania major strain Friedlin              |E|Euglenozoa Kinetoplastida          | >gi|157877645|ref|XP_001687137.1| hypothetical protein [Leishmania major strain Friedlin]
124075960 44..289    |Danio rerio                                   |E|Metazoa Chordata                   | >gi|124075960|sp|Q7ZVH1.2|MPRGA_DANRE RecName: Full=Membrane progestin receptor gamma-A; Short=mPR gamma-A; AltName: Full=Progestin and adipoQ receptor family member V-A
41055698  44..289    |Danio rerio                                   |E|Metazoa Chordata                   | >gi|41055698|ref|NP_956481.1| membrane progestin receptor gamma-A [Danio rerio]
260950145 63..308    |Clavispora lusitaniae ATCC 42720              |E|Fungi Dikarya                      | >gi|260950145|ref|XP_002619369.1| hypothetical protein CLUG_00528 [Clavispora lusitaniae ATCC 42720]
57525819  44..289    |Danio rerio                                   |E|Metazoa Chordata                   | >gi|57525819|ref|NP_001003573.1| membrane progestin receptor gamma-B [Danio rerio]
166158104 44..274    |Xenopus (Silurana) tropicalis                 |E|Metazoa Chordata                   | >gi|166158104|ref|NP_001107460.1| hypothetical protein LOC100135309 [Xenopus (Silurana) tropicalis]
261333050 89..333    |Trypanosoma brucei gambiense DAL972           |E|Euglenozoa Kinetoplastida          | >gi|261333050|emb|CBH16045.1| hypothetical protein, conserved [Trypanosoma brucei gambiense DAL972]
71748200  89..333    |Trypanosoma brucei TREU927                    |E|Euglenozoa Kinetoplastida          | >gi|71748200|ref|XP_823155.1| hypothetical protein [Trypanosoma brucei TREU927]
150951535 40..283    |Scheffersomyces stipitis CBS 6054             |E|Fungi Dikarya                      | >gi|150951535|ref|XP_001387872.2| hemolysin III domain membrane protein [Scheffersomyces stipitis CBS 6054]
118404428 43..285    |Xenopus (Silurana) tropicalis                 |E|Metazoa Chordata                   | >gi|118404428|ref|NP_001072708.1| progestin and adipoQ receptor family member V [Xenopus (Silurana) tropicalis]
146421742 63..305    |Meyerozyma guilliermondii ATCC 6260           |E|Fungi Dikarya                      | >gi|146421742|ref|XP_001486815.1| hypothetical protein PGUG_00192 [Meyerozyma guilliermondii ATCC 6260]
222636004 101..343   |Oryza sativa Japonica Group                   |E|Viridiplantae Streptophyta         | >gi|222636004|gb|EEE66136.1| hypothetical protein OsJ_22189 [Oryza sativa Japonica Group]
224142557 89..331    |Populus trichocarpa                           |E|Viridiplantae Streptophyta         | >gi|224142557|ref|XP_002324622.1| predicted protein [Populus trichocarpa]
294654468 84..326    |Debaryomyces hansenii CBS767                  |E|Fungi Dikarya                      | >gi|294654468|ref|XP_456527.2| DEHA2A04730p [Debaryomyces hansenii CBS767]
238879037 68..309    |Candida albicans WO-1                         |E|Fungi Dikarya                      | >gi|238879037|gb|EEQ42675.1| conserved hypothetical protein [Candida albicans WO-1]
241948841 68..309    |Candida dubliniensis CD36                     |E|Fungi Dikarya                      | >gi|241948841|ref|XP_002417143.1| haemolysis-ralated integral membrane protein, putative [Candida dubliniensis CD36]
68490638  68..309    |Candida albicans SC5314                       |E|Fungi Dikarya                      | >gi|68490638|ref|XP_710866.1| potential haemolysin-related integral membrane protein [Candida albicans SC5314]
68490663  68..309    |Candida albicans SC5314                       |E|Fungi Dikarya                      | >gi|68490663|ref|XP_710854.1| potential haemolysin-related integral membrane protein [Candida albicans SC5314]
255730613 71..312    |Candida tropicalis MYA-3404                   |E|Fungi Dikarya                      | >gi|255730613|ref|XP_002550231.1| conserved hypothetical protein [Candida tropicalis MYA-3404]
124088404 63..303    |Paramecium tetraurelia strain d4-2            |E|Alveolata Ciliophora               | >gi|124088404|ref|XP_001347088.1| Progestin and adipoQ receptor family [Paramecium tetraurelia strain d4-2]
290979798 115..355   |Naegleria gruberi strain NEG-M                |E|Heterolobosea Schizopyrenida       | >gi|290979798|ref|XP_002672620.1| predicted protein [Naegleria gruberi]
50290285  43..282    |Candida glabrata CBS 138                      |E|Fungi Dikarya                      | >gi|50290285|ref|XP_447574.1| hypothetical protein [Candida glabrata CBS 138]
149244798 99..337    |Lodderomyces elongisporus NRRL YB-4239        |E|Fungi Dikarya                      | >gi|149244798|ref|XP_001526942.1| hypothetical protein LELG_01771 [Lodderomyces elongisporus NRRL YB-4239]
71650344  87..324    |Trypanosoma cruzi strain CL Brener            |E|Euglenozoa Kinetoplastida          | >gi|71650344|ref|XP_813872.1| hypothetical protein [Trypanosoma cruzi strain CL Brener]
71651026  87..324    |Trypanosoma cruzi strain CL Brener            |E|Euglenozoa Kinetoplastida          | >gi|71651026|ref|XP_814199.1| hypothetical protein [Trypanosoma cruzi strain CL Brener]
153792423 65..301    |Danio rerio                                   |E|Metazoa Chordata                   | >gi|153792423|ref|NP_001093530.1| similar to G protein coupled progestin receptor gamma [Danio rerio]
242096558 130..366   |Sorghum bicolor                               |E|Viridiplantae Streptophyta         | >gi|242096558|ref|XP_002438769.1| hypothetical protein SORBIDRAFT_10g025870 [Sorghum bicolor]
196015209 66..301    |Trichoplax adhaerens                          |E|Metazoa Placozoa                   | >gi|196015209|ref|XP_002117462.1| hypothetical protein TRIADDRAFT_32617 [Trichoplax adhaerens]
145356647 30..264    |Ostreococcus lucimarinus CCE9901              |E|Viridiplantae Chlorophyta          | >gi|145356647|ref|XP_001422539.1| predicted protein [Ostreococcus lucimarinus CCE9901]
109017337 43..276    |Macaca mulatta                                |E|Metazoa Chordata                   | >gi|109017337|ref|XP_001116489.1| PREDICTED: progestin and adipoQ receptor family member 6-like isoform 1 [Macaca mulatta]
114560236 43..276    |Pan troglodytes                               |E|Metazoa Chordata                   | >gi|114560236|ref|XP_001164771.1| PREDICTED: progestin and adipoQ receptor family member VI isoform 6 [Pan troglodytes]
114560240 19..252    |Pan troglodytes                               |E|Metazoa Chordata                   | >gi|114560240|ref|XP_001164692.1| PREDICTED: similar to Progestin and adipoQ receptor family member VI isoform 5 [Pan troglodytes]
119573367 40..273    |Homo sapiens                                  |E|Metazoa Chordata                   | >gi|119573367|gb|EAW52982.1| progestin and adipoQ receptor family member VI, isoform CRA_g [Homo sapiens]
296229162 43..276    |Callithrix jacchus                            |E|Metazoa Chordata                   | >gi|296229162|ref|XP_002760142.1| PREDICTED: progestin and adipoQ receptor family member 6 isoform 1 [Callithrix jacchus]
296229164 19..252    |Callithrix jacchus                            |E|Metazoa Chordata                   | >gi|296229164|ref|XP_002760143.1| PREDICTED: progestin and adipoQ receptor family member 6 isoform 2 [Callithrix jacchus]
297280299 19..252    |Macaca mulatta                                |E|Metazoa Chordata                   | >gi|297280299|ref|XP_002801902.1| PREDICTED: progestin and adipoQ receptor family member 6-like isoform 4 [Macaca mulatta]
297663228 43..276    |Pongo abelii                                  |E|Metazoa Chordata                   | >gi|297663228|ref|XP_002810077.1| PREDICTED: progestin and adipoQ receptor family member 6-like isoform 1 [Pongo abelii]
297663234 19..252    |Pongo abelii                                  |E|Metazoa Chordata                   | >gi|297663234|ref|XP_002810080.1| PREDICTED: progestin and adipoQ receptor family member 6-like isoform 4 [Pongo abelii]
38018653  43..276    |Homo sapiens                                  |E|Metazoa Chordata                   | >gi|38018653|gb|AAR08372.1| progestin and adipoQ receptor family member VI [Homo sapiens]
41282031  43..276    |Homo sapiens                                  |E|Metazoa Chordata                   | >gi|41282031|ref|NP_940798.1| progestin and adipoQ receptor family member 6 isoform 2 [Homo sapiens]
55957519  19..252    |Homo sapiens                                  |E|Metazoa Chordata                   | >gi|55957519|emb|CAI15543.1| progestin and adipoQ receptor family member VI [Homo sapiens]
59006763  43..276    |Homo sapiens                                  |E|Metazoa Chordata                   | >gi|59006763|emb|CAD38905.2| hypothetical protein [Homo sapiens]
114560228 43..254    |Pan troglodytes                               |E|Metazoa Chordata                   | >gi|114560228|ref|XP_001164805.1| PREDICTED: progestin and adipoQ receptor family member VI isoform 7 [Pan troglodytes]
33187697  19..204    |Homo sapiens                                  |E|Metazoa Chordata                   | >gi|33187697|gb|AAP97703.1|AF455047_1 hypothetical protein variant 2 [Homo sapiens]
134117570 80..313    |Cryptococcus neoformans var. neoformans B-3501A|E|Fungi Dikarya                      | >gi|134117570|ref|XP_772556.1| hypothetical protein CNBL0360 [Cryptococcus neoformans var. neoformans B-3501A]
58270148  19..247    |Cryptococcus neoformans var. neoformans JEC21 |E|Fungi Dikarya                      | >gi|58270148|ref|XP_572230.1| integral to membrane protein [Cryptococcus neoformans var. neoformans JEC21]
149414655 104..337   |Ornithorhynchus anatinus                      |E|Metazoa Chordata                   | >gi|149414655|ref|XP_001515913.1| PREDICTED: similar to Paqr5 protein [Ornithorhynchus anatinus]
164448618 43..276    |Bos taurus                                    |E|Metazoa Chordata                   | >gi|164448618|ref|NP_001039690.2| progestin and adipoQ receptor family member VI [Bos taurus]
194036014 43..276    |Sus scrofa                                    |E|Metazoa Chordata                   | >gi|194036014|ref|XP_001927888.1| PREDICTED: similar to progestin and adipoQ receptor family member VI [Sus scrofa]
194210676 43..276    |Equus caballus                                |E|Metazoa Chordata                   | >gi|194210676|ref|XP_001495132.2| PREDICTED: similar to progestin and adipoQ receptor family member VI [Equus caballus]
296489682 43..276    |Bos taurus                                    |E|Metazoa Chordata                   | >gi|296489682|gb|DAA31795.1| progestin and adipoQ receptor family member VI [Bos taurus]
254572489 65..298    |Pichia pastoris GS115                         |E|Fungi Dikarya                      | >gi|254572489|ref|XP_002493354.1| Plasma membrane protein involved in zinc metabolism and osmotin-induced apoptosis [Pichia pastoris GS115]
109071487 70..302    |Macaca mulatta                                |E|Metazoa Chordata                   | >gi|109071487|ref|XP_001107033.1| PREDICTED: membrane progestin receptor beta-like isoform 1 [Macaca mulatta]
126310130 70..302    |Monodelphis domestica                         |E|Metazoa Chordata                   | >gi|126310130|ref|XP_001364107.1| PREDICTED: membrane progestin receptor beta-like [Monodelphis domestica]
149732270 70..302    |Equus caballus                                |E|Metazoa Chordata                   | >gi|149732270|ref|XP_001503004.1| PREDICTED: progestin and adipoQ receptor family member VIII [Equus caballus]
19115960  70..302    |Homo sapiens                                  |E|Metazoa Chordata                   | >gi|19115960|ref|NP_588608.1| membrane progestin receptor beta [Homo sapiens]
193785570 75..307    |Homo sapiens                                  |E|Metazoa Chordata                   | >gi|193785570|dbj|BAG54628.1| unnamed protein product [Homo sapiens]
26327777  70..302    |Mus musculus                                  |E|Metazoa Chordata                   | >gi|26327777|dbj|BAC27629.1| unnamed protein product [Mus musculus]
26339100  70..302    |Mus musculus                                  |E|Metazoa Chordata                   | >gi|26339100|dbj|BAC33221.1| unnamed protein product [Mus musculus]
281339639 72..304    |Ailuropoda melanoleuca                        |E|Metazoa Chordata                   | >gi|281339639|gb|EFB15223.1| hypothetical protein PANDA_003448 [Ailuropoda melanoleuca]
28569053  70..302    |Mus musculus                                  |E|Metazoa Chordata                   | >gi|28569053|gb|AAO47230.1|AF313617_1 putative membrane steroid receptor [Mus musculus]
291396372 70..302    |Oryctolagus cuniculus                         |E|Metazoa Chordata                   | >gi|291396372|ref|XP_002714547.1| PREDICTED: progestin and adipoQ receptor family member VIII [Oryctolagus cuniculus]
296198399 87..319    |Callithrix jacchus                            |E|Metazoa Chordata                   | >gi|296198399|ref|XP_002746731.1| PREDICTED: membrane progestin receptor beta-like [Callithrix jacchus]
34190825  70..302    |Homo sapiens                                  |E|Metazoa Chordata                   | >gi|34190825|gb|AAH30664.2| Progestin and adipoQ receptor family member VIII [Homo sapiens]
40254346  70..302    |Mus musculus                                  |E|Metazoa Chordata                   | >gi|40254346|ref|NP_083105.3| membrane progestin receptor beta [Mus musculus]
55626870  75..307    |Pan troglodytes                               |E|Metazoa Chordata                   | >gi|55626870|ref|XP_527410.1| PREDICTED: membrane progestin receptor beta isoform 3 [Pan troglodytes]
62078931  70..302    |Rattus norvegicus                             |E|Metazoa Chordata                   | >gi|62078931|ref|NP_001014121.1| membrane progestin receptor beta [Rattus norvegicus]
73973314  70..302    |Canis lupus familiaris                        |E|Metazoa Chordata                   | >gi|73973314|ref|XP_538961.2| PREDICTED: similar to progestin and adipoQ receptor family member VIII [Canis familiaris]
109465054 43..275    |Rattus norvegicus                             |E|Metazoa Chordata                   | >gi|109465054|ref|XP_001059976.1| PREDICTED: progestin and adipoQ receptor family member VI [Rattus norvegicus]
38259190  43..275    |Mus musculus                                  |E|Metazoa Chordata                   | >gi|38259190|ref|NP_940802.1| progestin and adipoQ receptor family member 6 [Mus musculus]
149048143 43..171    |Rattus norvegicus                             |E|Metazoa Chordata                   | >gi|149048143|gb|EDM00719.1| rCG62446, isoform CRA_a [Rattus norvegicus]
149412367 70..302    |Ornithorhynchus anatinus                      |E|Metazoa Chordata                   | >gi|149412367|ref|XP_001509948.1| PREDICTED: similar to transmembrane protein [Ornithorhynchus anatinus]
15241302  90..322    |Arabidopsis thaliana                          |E|Viridiplantae Streptophyta         | >gi|15241302|ref|NP_197527.1| heptahelical transmembrane protein1 [Arabidopsis thaliana]
297812207 25..257    |Arabidopsis lyrata subsp. lyrata              |E|Viridiplantae Streptophyta         | >gi|297812207|ref|XP_002873987.1| hypothetical protein ARALYDRAFT_326421 [Arabidopsis lyrata subsp. lyrata]
155371997 70..302    |Bos taurus                                    |E|Metazoa Chordata                   | >gi|155371997|ref|NP_001094605.1| membrane progestin receptor beta [Bos taurus]
241570051 73..305    |Ixodes scapularis                             |E|Metazoa Arthropoda                 | >gi|241570051|ref|XP_002402648.1| adiponectin receptor, putative [Ixodes scapularis]
256087096 68..300    |Schistosoma mansoni                           |E|Metazoa Platyhelminthes            | >gi|256087096|ref|XP_002579713.1| progestin and adipoq receptor family member VI [Schistosoma mansoni]
291242644 44..276    |Saccoglossus kowalevskii                      |E|Metazoa Hemichordata               | >gi|291242644|ref|XP_002741216.1| PREDICTED: progestin and adipoQ receptor family member VIII-like [Saccoglossus kowalevskii]
47523352  70..302    |Sus scrofa                                    |E|Metazoa Chordata                   | >gi|47523352|ref|NP_998905.1| membrane progestin receptor beta [Sus scrofa]
28569057  2..170     |Homo sapiens                                  |E|Metazoa Chordata                   | >gi|28569057|gb|AAO47232.1|AF313619_1 putative membrane steroid receptor [Homo sapiens]
50553322  82..314    |Yarrowia lipolytica CLIB122                   |E|Fungi Dikarya                      | >gi|50553322|ref|XP_504072.1| YALI0E17677p [Yarrowia lipolytica]
56755809  71..303    |Schistosoma japonicum                         |E|Metazoa Platyhelminthes            | >gi|56755809|gb|AAW26083.1| SJCHGC00917 protein [Schistosoma japonicum]
56755898  71..303    |Schistosoma japonicum                         |E|Metazoa Platyhelminthes            | >gi|56755898|gb|AAW26127.1| unknown [Schistosoma japonicum]
109081693 43..274    |Macaca mulatta                                |E|Metazoa Chordata                   | >gi|109081693|ref|XP_001085851.1| PREDICTED: membrane progestin receptor gamma isoform 1 [Macaca mulatta]
114657835 43..274    |Pan troglodytes                               |E|Metazoa Chordata                   | >gi|114657835|ref|XP_001174881.1| PREDICTED: hypothetical protein isoform 2 [Pan troglodytes]
114657839 43..274    |Pan troglodytes                               |E|Metazoa Chordata                   | >gi|114657839|ref|XP_001174879.1| PREDICTED: hypothetical protein isoform 1 [Pan troglodytes]
157389021 43..274    |Homo sapiens                                  |E|Metazoa Chordata                   | >gi|157389021|ref|NP_001098024.1| membrane progestin receptor gamma [Homo sapiens]
24657741  43..274    |Homo sapiens                                  |E|Metazoa Chordata                   | >gi|24657741|gb|AAH39234.1| Progestin and adipoQ receptor family member V [Homo sapiens]
197100449 1..230     |Pongo abelii                                  |E|Metazoa Chordata                   | >gi|197100449|ref|NP_001127441.1| membrane progestin receptor gamma [Pongo abelii]
67971370  25..128    |Macaca fascicularis                           |E|Metazoa Chordata                   | >gi|67971370|dbj|BAE02027.1| unnamed protein product [Macaca fascicularis]
118197121 19..250    |Carassius auratus                             |E|Metazoa Chordata                   | >gi|118197121|dbj|BAF37036.1| membrane progestin receptor gamma-2 [Carassius auratus]
118404208 70..301    |Xenopus (Silurana) tropicalis                 |E|Metazoa Chordata                   | >gi|118404208|ref|NP_001072419.1| progestin and adipoQ receptor family member VIII [Xenopus (Silurana) tropicalis]
126277579 293..524   |Monodelphis domestica                         |E|Metazoa Chordata                   | >gi|126277579|ref|XP_001376974.1| PREDICTED: membrane progestin receptor gamma-like [Monodelphis domestica]
147906649 70..301    |Xenopus laevis                                |E|Metazoa Chordata                   | >gi|147906649|ref|NP_001079330.1| progestin and adipoQ receptor family member VIII [Xenopus laevis]
213625127 70..301    |Xenopus laevis                                |E|Metazoa Chordata                   | >gi|213625127|gb|AAI69886.1| Membrane progesterone receptor-like [Xenopus laevis]
213626685 70..301    |Xenopus laevis                                |E|Metazoa Chordata                   | >gi|213626685|gb|AAI69888.1| Mpr-a protein [Xenopus laevis]
148228527 43..274    |Xenopus laevis                                |E|Metazoa Chordata                   | >gi|148228527|ref|NP_001087406.1| progestin and adipoQ receptor family member VI [Xenopus laevis]
148232234 43..274    |Xenopus laevis                                |E|Metazoa Chordata                   | >gi|148232234|ref|NP_001089391.1| hypothetical protein LOC734441 [Xenopus laevis]
149692287 191..422   |Equus caballus                                |E|Metazoa Chordata                   | >gi|149692287|ref|XP_001495614.1| PREDICTED: similar to Membrane progestin receptor gamma (mPR gamma) (Progestin and adipoQ receptor family member V) [Equus caballus]
156370264 63..294    |Nematostella vectensis                        |E|Metazoa Cnidaria                   | >gi|156370264|ref|XP_001628391.1| predicted protein [Nematostella vectensis]
156385236 75..306    |Nematostella vectensis                        |E|Metazoa Cnidaria                   | >gi|156385236|ref|XP_001633537.1| predicted protein [Nematostella vectensis]
189530236 43..274    |Danio rerio                                   |E|Metazoa Chordata                   | >gi|189530236|ref|XP_699176.3| PREDICTED: progestin and adipoQ receptor family member 6 [Danio rerio]
194034367 29..260    |Sus scrofa                                    |E|Metazoa Chordata                   | >gi|194034367|ref|XP_001928087.1| PREDICTED: similar to Membrane progestin receptor gamma (mPR gamma) (Progestin and adipoQ receptor family member V) [Sus scrofa]
224048908 69..300    |Taeniopygia guttata                           |E|Metazoa Chordata                   | >gi|224048908|ref|XP_002192018.1| PREDICTED: progestin and adipoQ receptor family member VIII [Taeniopygia guttata]
56605924  70..299    |Gallus gallus                                 |E|Metazoa Chordata                   | >gi|56605924|ref|NP_001008462.1| membrane progestin receptor beta [Gallus gallus]
254720803 64..295    |Gallus gallus                                 |E|Metazoa Chordata                   | >gi|254720803|ref|NP_001157123.1| progestin and adipoQ receptor family member VII [Gallus gallus]
260784105 51..282    |Branchiostoma floridae                        |E|Metazoa Chordata                   | >gi|260784105|ref|XP_002587109.1| hypothetical protein BRAFLDRAFT_241959 [Branchiostoma floridae]
281343957 28..259    |Ailuropoda melanoleuca                        |E|Metazoa Chordata                   | >gi|281343957|gb|EFB19541.1| hypothetical protein PANDA_009438 [Ailuropoda melanoleuca]
291412309 58..289    |Oryctolagus cuniculus                         |E|Metazoa Chordata                   | >gi|291412309|ref|XP_002722429.1| PREDICTED: progestin and adipoQ receptor family member V [Oryctolagus cuniculus]
296213573 112..343   |Callithrix jacchus                            |E|Metazoa Chordata                   | >gi|296213573|ref|XP_002753334.1| PREDICTED: membrane progestin receptor gamma-like [Callithrix jacchus]
7020125   43..274    |Homo sapiens                                  |E|Metazoa Chordata                   | >gi|7020125|dbj|BAA91004.1| unnamed protein product [Homo sapiens]
297459418 43..274    |Bos taurus                                    |E|Metazoa Chordata                   | >gi|297459418|ref|XP_605853.5| PREDICTED: progestin and adipoQ receptor family member V [Bos taurus]
32766270  43..274    |Mus musculus                                  |E|Metazoa Chordata                   | >gi|32766270|gb|AAH54855.1| Paqr5 protein [Mus musculus]
58037337  43..274    |Mus musculus                                  |E|Metazoa Chordata                   | >gi|58037337|ref|NP_083024.1| membrane progestin receptor gamma [Mus musculus]
62078917  43..274    |Rattus norvegicus                             |E|Metazoa Chordata                   | >gi|62078917|ref|NP_001014114.1| membrane progestin receptor gamma [Rattus norvegicus]
68271306  43..274    |Rattus norvegicus                             |E|Metazoa Chordata                   | >gi|68271306|gb|AAY89126.1| progestin membrane receptor gamma [Rattus norvegicus]
71680034  29..260    |Mus musculus                                  |E|Metazoa Chordata                   | >gi|71680034|gb|AAI00517.1| Paqr5 protein [Mus musculus]
34330174  70..301    |Danio rerio                                   |E|Metazoa Chordata                   | >gi|34330174|ref|NP_899188.1| membrane progestin receptor alpha-B [Danio rerio]
40714507  70..301    |Carassius auratus                             |E|Metazoa Chordata                   | >gi|40714507|dbj|BAD06917.1| membrane progestin receptor alpha [Carassius auratus]
42767641  70..301    |Ictalurus punctatus                           |E|Metazoa Chordata                   | >gi|42767641|gb|AAS45554.1| putative membrane progestin receptor alpha [Ictalurus punctatus]
70984745  73..304    |Aspergillus fumigatus Af293                   |E|Fungi Dikarya                      | >gi|70984745|ref|XP_747879.1| haemolysin-III family protein [Aspergillus fumigatus Af293]
74000931  88..319    |Canis lupus familiaris                        |E|Metazoa Chordata                   | >gi|74000931|ref|XP_544748.2| PREDICTED: similar to Membrane progestin receptor gamma (mPR gamma) (Progestin and adipoQ receptor family member V) [Canis familiaris]
118090154 64..294    |Gallus gallus                                 |E|Metazoa Chordata                   | >gi|118090154|ref|XP_001233721.1| PREDICTED: similar to progestin and adipoQ receptor family member III [Gallus gallus]
118197119 44..274    |Carassius auratus                             |E|Metazoa Chordata                   | >gi|118197119|dbj|BAF37035.1| membrane progestin receptor gamma-1 [Carassius auratus]
149639450 77..307    |Ornithorhynchus anatinus                      |E|Metazoa Chordata                   | >gi|149639450|ref|XP_001506452.1| PREDICTED: similar to Progestin and adipoQ receptor family member 3 (Progestin and adipoQ receptor family member III) [Ornithorhynchus anatinus]
156408540 53..283    |Nematostella vectensis                        |E|Metazoa Cnidaria                   | >gi|156408540|ref|XP_001641914.1| predicted protein [Nematostella vectensis]
196007350 36..266    |Trichoplax adhaerens                          |E|Metazoa Placozoa                   | >gi|196007350|ref|XP_002113541.1| hypothetical protein TRIADDRAFT_15474 [Trichoplax adhaerens]
239614995 73..303    |Ajellomyces dermatitidis ER-3                 |E|Fungi Dikarya                      | >gi|239614995|gb|EEQ91982.1| hemolysin-III channel protein Izh2 [Ajellomyces dermatitidis ER-3]
261187962 73..303    |Ajellomyces dermatitidis SLH14081             |E|Fungi Dikarya                      | >gi|261187962|ref|XP_002620398.1| hemolysin-III channel protein Izh2 [Ajellomyces dermatitidis SLH14081]
260817148 41..271    |Branchiostoma floridae                        |E|Metazoa Chordata                   | >gi|260817148|ref|XP_002603449.1| hypothetical protein BRAFLDRAFT_280976 [Branchiostoma floridae]
260817152 41..271    |Branchiostoma floridae                        |E|Metazoa Chordata                   | >gi|260817152|ref|XP_002603451.1| hypothetical protein BRAFLDRAFT_80425 [Branchiostoma floridae]
42767645  44..274    |Ictalurus punctatus                           |E|Metazoa Chordata                   | >gi|42767645|gb|AAS45556.1| putative membrane progestin receptor gamma [Ictalurus punctatus]
47214510  113..343   |Tetraodon nigroviridis                        |E|Metazoa Chordata                   | >gi|47214510|emb|CAG00934.1| unnamed protein product [Tetraodon nigroviridis]
47230691  18..248    |Tetraodon nigroviridis                        |E|Metazoa Chordata                   | >gi|47230691|emb|CAF99884.1| unnamed protein product [Tetraodon nigroviridis]
71834444  64..294    |Danio rerio                                   |E|Metazoa Chordata                   | >gi|71834444|ref|NP_001025319.1| progestin and adipoQ receptor family member IIIb [Danio rerio]
108999660 66..295    |Macaca mulatta                                |E|Metazoa Chordata                   | >gi|108999660|ref|XP_001107865.1| PREDICTED: membrane progestin receptor alpha-like isoform 1 [Macaca mulatta]
114554799 66..295    |Pan troglodytes                               |E|Metazoa Chordata                   | >gi|114554799|ref|XP_001136360.1| PREDICTED: membrane progestin receptor alpha isoform 1 [Pan troglodytes]
296207103 66..295    |Callithrix jacchus                            |E|Metazoa Chordata                   | >gi|296207103|ref|XP_002750501.1| PREDICTED: membrane progestin receptor alpha-like [Callithrix jacchus]
297665955 66..295    |Pongo abelii                                  |E|Metazoa Chordata                   | >gi|297665955|ref|XP_002811302.1| PREDICTED: membrane progestin receptor alpha-like [Pongo abelii]
30410020  66..295    |Homo sapiens                                  |E|Metazoa Chordata                   | >gi|30410020|ref|NP_848509.1| membrane progestin receptor alpha [Homo sapiens]
54035159  66..295    |Homo sapiens                                  |E|Metazoa Chordata                   | >gi|54035159|gb|AAH34015.1| Progestin and adipoQ receptor family member VII [Homo sapiens]
121934115 18..221    |Homo sapiens                                  |E|Metazoa Chordata                   | >gi|121934115|gb|AAI27801.1| PAQR7 protein [Homo sapiens]
109627672 70..299    |Takifugu rubripes                             |E|Metazoa Chordata                   | >gi|109627672|ref|NP_001035912.1| progestin and adipoQ receptor family member VII [Takifugu rubripes]
47220843  69..298    |Tetraodon nigroviridis                        |E|Metazoa Chordata                   | >gi|47220843|emb|CAG00050.1| unnamed protein product [Tetraodon nigroviridis]
118197117 72..301    |Carassius auratus                             |E|Metazoa Chordata                   | >gi|118197117|dbj|BAF37034.1| membrane progestin receptor beta [Carassius auratus]
126328639 71..300    |Monodelphis domestica                         |E|Metazoa Chordata                   | >gi|126328639|ref|XP_001369036.1| PREDICTED: membrane progestin receptor alpha-like [Monodelphis domestica]
154324234 159..388   |Botryotinia fuckeliana B05.10                 |E|Fungi Dikarya                      | >gi|154324234|ref|XP_001561431.1| hypothetical protein BC1G_00516 [Botryotinia fuckeliana B05.10]
156481282 70..299    |Micropogonias undulatus                       |E|Metazoa Chordata                   | >gi|156481282|gb|ABU68407.1| membrane progestin receptor alpha [Micropogonias undulatus]
51316387  70..299    |Cynoscion nebulosus                           |E|Metazoa Chordata                   | >gi|51316387|sp|Q801D8.1|MPRA_CYNNE RecName: Full=Membrane progestin receptor alpha; Short=mPR alpha; AltName: Full=Membrane progesterone receptor
215511355 1..154     |Micropterus salmoides                         |E|Metazoa Chordata                   | >gi|215511355|gb|ACJ67879.1| membrane progestin receptor A [Micropterus salmoides]
224120350 75..304    |Populus trichocarpa                           |E|Viridiplantae Streptophyta         | >gi|224120350|ref|XP_002331026.1| predicted protein [Populus trichocarpa]
254578502 67..296    |Zygosaccharomyces rouxii CBS 732              |E|Fungi Dikarya                      | >gi|254578502|ref|XP_002495237.1| ZYRO0B06556p [Zygosaccharomyces rouxii]
259880224 70..299    |Paralichthys lethostigma                      |E|Metazoa Chordata                   | >gi|259880224|gb|ACW83621.1| membrane progestin receptor alpha [Paralichthys lethostigma]
260802702 41..270    |Branchiostoma floridae                        |E|Metazoa Chordata                   | >gi|260802702|ref|XP_002596231.1| hypothetical protein BRAFLDRAFT_203007 [Branchiostoma floridae]
260803346 46..275    |Branchiostoma floridae                        |E|Metazoa Chordata                   | >gi|260803346|ref|XP_002596551.1| hypothetical protein BRAFLDRAFT_96405 [Branchiostoma floridae]
260805312 38..267    |Branchiostoma floridae                        |E|Metazoa Chordata                   | >gi|260805312|ref|XP_002597531.1| hypothetical protein BRAFLDRAFT_78914 [Branchiostoma floridae]
260805314 38..267    |Branchiostoma floridae                        |E|Metazoa Chordata                   | >gi|260805314|ref|XP_002597532.1| hypothetical protein BRAFLDRAFT_78913 [Branchiostoma floridae]
260817146 41..270    |Branchiostoma floridae                        |E|Metazoa Chordata                   | >gi|260817146|ref|XP_002603448.1| hypothetical protein BRAFLDRAFT_222672 [Branchiostoma floridae]
260817150 41..270    |Branchiostoma floridae                        |E|Metazoa Chordata                   | >gi|260817150|ref|XP_002603450.1| hypothetical protein BRAFLDRAFT_80424 [Branchiostoma floridae]
260817154 41..270    |Branchiostoma floridae                        |E|Metazoa Chordata                   | >gi|260817154|ref|XP_002603452.1| hypothetical protein BRAFLDRAFT_80426 [Branchiostoma floridae]
281351583 64..293    |Ailuropoda melanoleuca                        |E|Metazoa Chordata                   | >gi|281351583|gb|EFB27167.1| hypothetical protein PANDA_001133 [Ailuropoda melanoleuca]
294489344 70..299    |Oryzias latipes                               |E|Metazoa Chordata                   | >gi|294489344|ref|NP_001170947.1| G protein coupled progestin receptor alpha [Oryzias latipes]
34330180  72..301    |Danio rerio                                   |E|Metazoa Chordata                   | >gi|34330180|ref|NP_899187.1| membrane progestin receptor beta [Danio rerio]
62204252  72..301    |Danio rerio                                   |E|Metazoa Chordata                   | >gi|62204252|gb|AAH92682.1| Progestin and adipoQ receptor family member VIII [Danio rerio]
41152309  65..294    |Danio rerio                                   |E|Metazoa Chordata                   | >gi|41152309|ref|NP_957004.1| progestin and adipoQ receptor family member 3 [Danio rerio]
47217771  69..298    |Tetraodon nigroviridis                        |E|Metazoa Chordata                   | >gi|47217771|emb|CAG05993.1| unnamed protein product [Tetraodon nigroviridis]
47523358  68..297    |Sus scrofa                                    |E|Metazoa Chordata                   | >gi|47523358|ref|NP_998904.1| membrane progestin receptor alpha [Sus scrofa]
84039870  68..297    |Ovis aries                                    |E|Metazoa Chordata                   | >gi|84039870|gb|ABC49848.1| membrane progesterone receptor [Ovis aries]
84370139  68..297    |Bos taurus                                    |E|Metazoa Chordata                   | >gi|84370139|ref|NP_001033642.1| progestin and adipoQ receptor family member VII [Bos taurus]
50305585  67..296    |Kluyveromyces lactis NRRL Y-1140              |E|Fungi Dikarya                      | >gi|50305585|ref|XP_452753.1| hypothetical protein [Kluyveromyces lactis NRRL Y-1140]
66800017  131..360   |Dictyostelium discoideum AX4                  |E|Amoebozoa Mycetozoa                | >gi|66800017|ref|XP_628934.1| Hly-III related family protein [Dictyostelium discoideum AX4]
78675497  73..302    |Oryzias latipes                               |E|Metazoa Chordata                   | >gi|78675497|dbj|BAE47504.1| G protein coupled progestin receptor beta [Oryzias latipes]
149695088 68..296    |Equus caballus                                |E|Metazoa Chordata                   | >gi|149695088|ref|XP_001504165.1| PREDICTED: similar to putative membrane steroid receptor [Equus caballus]
151942455 75..303    |Saccharomyces cerevisiae YJM789               |E|Fungi Dikarya                      | >gi|151942455|gb|EDN60811.1| membrane protein involved in zinc metabolism [Saccharomyces cerevisiae YJM789]
190404587 75..303    |Saccharomyces cerevisiae RM11-1a              |E|Fungi Dikarya                      | >gi|190404587|gb|EDV07854.1| hypothetical protein SCRG_00050 [Saccharomyces cerevisiae RM11-1a]
45270876  75..303    |Saccharomyces cerevisiae                      |E|Fungi Dikarya                      | >gi|45270876|gb|AAS56819.1| YDR492W [Saccharomyces cerevisiae]
6320699   75..303    |Saccharomyces cerevisiae S288c                |E|Fungi Dikarya                      | >gi|6320699|ref|NP_010780.1| Izh1p [Saccharomyces cerevisiae S288c]
171676938 83..311    |Podospora anserina S mat+                     |E|Fungi Dikarya                      | >gi|171676938|ref|XP_001903421.1| hypothetical protein [Podospora anserina S mat+]
221121397 268..496   |Hydra magnipapillata                          |E|Metazoa Cnidaria                   | >gi|221121397|ref|XP_002166013.1| PREDICTED: similar to predicted protein [Hydra magnipapillata]
241154913 82..310    |Ixodes scapularis                             |E|Metazoa Arthropoda                 | >gi|241154913|ref|XP_002407410.1| adiponectin receptor, putative [Ixodes scapularis]
255712703 70..298    |Lachancea thermotolerans CBS 6340             |E|Fungi Dikarya                      | >gi|255712703|ref|XP_002552634.1| KLTH0C09504p [Lachancea thermotolerans]
296812597 75..303    |Arthroderma otae CBS 113480                   |E|Fungi Dikarya                      | >gi|296812597|ref|XP_002846636.1| hemolysin-III family protein [Arthroderma otae CBS 113480]
298405163 143..371   |Coprinopsis cinerea okayama7#130              |E|Fungi Dikarya                      | >gi|298405163|gb|EFI27064.1| HlyIII channel protein [Coprinopsis cinerea okayama7#130]
169851489 1..113     |Coprinopsis cinerea okayama7#130              |E|Fungi Dikarya                      | >gi|169851489|ref|XP_001832434.1| hypothetical protein CC1G_11059 [Coprinopsis cinerea okayama7#130]
34392790  70..298    |Oryzias latipes                               |E|Metazoa Chordata                   | >gi|34392790|dbj|BAC82706.1| G protein coupled progestin receptor gamma [Oryzias latipes]
42767643  72..300    |Ictalurus punctatus                           |E|Metazoa Chordata                   | >gi|42767643|gb|AAS45555.1| putative membrane progestin receptor beta [Ictalurus punctatus]
47216963  64..292    |Tetraodon nigroviridis                        |E|Metazoa Chordata                   | >gi|47216963|emb|CAG04905.1| unnamed protein product [Tetraodon nigroviridis]
50306311  68..296    |Kluyveromyces lactis NRRL Y-1140              |E|Fungi Dikarya                      | >gi|50306311|ref|XP_453128.1| hypothetical protein [Kluyveromyces lactis NRRL Y-1140]
72058996  63..291    |Strongylocentrotus purpuratus                 |E|Metazoa Echinodermata              | >gi|72058996|ref|XP_784040.1| PREDICTED: hypothetical protein [Strongylocentrotus purpuratus]
1150994   84..311    |Saccharomyces cerevisiae                      |E|Fungi Dikarya                      | >gi|1150994|gb|AAC49478.1| hypothetical protein UND327 [Saccharomyces cerevisiae]
27808716  74..301    |Saccharomyces cerevisiae S288c                |E|Fungi Dikarya                      | >gi|27808716|ref|NP_014641.2| Izh2p [Saccharomyces cerevisiae S288c]
115396250 77..304    |Aspergillus terreus NIH2624                   |E|Fungi Dikarya                      | >gi|115396250|ref|XP_001213764.1| conserved hypothetical protein [Aspergillus terreus NIH2624]
115534482 57..284    |Caenorhabditis elegans                        |E|Metazoa Nematoda                   | >gi|115534482|ref|NP_502745.2| hypothetical protein Y67A10A.8 [Caenorhabditis elegans]
268535124 57..284    |Caenorhabditis briggsae                       |E|Metazoa Nematoda                   | >gi|268535124|ref|XP_002632695.1| Hypothetical protein CBG21626 [Caenorhabditis briggsae]
119187101 81..308    |Coccidioides immitis RS                       |E|Fungi Dikarya                      | >gi|119187101|ref|XP_001244157.1| hypothetical protein CIMG_03598 [Coccidioides immitis RS]
240108320 77..304    |Coccidioides posadasii C735 delta SOWgp       |E|Fungi Dikarya                      | >gi|240108320|gb|EER26494.1| Hemolysin-III related family protein [Coccidioides posadasii C735 delta SOWgp]
119190241 85..312    |Coccidioides immitis RS                       |E|Fungi Dikarya                      | >gi|119190241|ref|XP_001245727.1| hypothetical protein CIMG_05168 [Coccidioides immitis RS]
240107174 85..312    |Coccidioides posadasii C735 delta SOWgp       |E|Fungi Dikarya                      | >gi|240107174|gb|EER25359.1| Hemolysin-III related family protein [Coccidioides posadasii C735 delta SOWgp]
119491815 75..302    |Neosartorya fischeri NRRL 181                 |E|Fungi Dikarya                      | >gi|119491815|ref|XP_001263402.1| haemolysin-III channel protein Izh2, putative [Neosartorya fischeri NRRL 181]
70999690  75..302    |Aspergillus fumigatus Af293                   |E|Fungi Dikarya                      | >gi|70999690|ref|XP_754562.1| haemolysin-III channel protein Izh2 [Aspergillus fumigatus Af293]
121705596 75..302    |Aspergillus clavatus NRRL 1                   |E|Fungi Dikarya                      | >gi|121705596|ref|XP_001271061.1| haemolysin-III channel protein Izh2, putative [Aspergillus clavatus NRRL 1]
145228359 83..310    |Aspergillus niger CBS 513.88                  |E|Fungi Dikarya                      | >gi|145228359|ref|XP_001388488.1| hemolysin-III channel protein Izh2 [Aspergillus niger CBS 513.88]
149024231 65..292    |Rattus norvegicus                             |E|Metazoa Chordata                   | >gi|149024231|gb|EDL80728.1| rCG31200 [Rattus norvegicus]
21313652  65..292    |Mus musculus                                  |E|Metazoa Chordata                   | >gi|21313652|ref|NP_082271.1| membrane progestin receptor alpha [Mus musculus]
28569055  65..292    |Mus musculus                                  |E|Metazoa Chordata                   | >gi|28569055|gb|AAO47231.1|AF313618_1 putative membrane steroid receptor [Mus musculus]
77539436  65..292    |Rattus norvegicus                             |E|Metazoa Chordata                   | >gi|77539436|ref|NP_001029253.1| progestin and adipoQ receptor family member VII [Rattus norvegicus]
154284552 9..236     |Ajellomyces capsulatus NAm1                   |E|Fungi Dikarya                      | >gi|154284552|ref|XP_001543071.1| conserved hypothetical protein [Ajellomyces capsulatus NAm1]
225556629 83..310    |Ajellomyces capsulatus G186AR                 |E|Fungi Dikarya                      | >gi|225556629|gb|EEH04917.1| hemolysin-III family protein [Ajellomyces capsulatus G186AR]
240281489 1..171     |Ajellomyces capsulatus H143                   |E|Fungi Dikarya                      | >gi|240281489|gb|EER44992.1| adipor-like receptor [Ajellomyces capsulatus H143]
154314660 69..296    |Botryotinia fuckeliana B05.10                 |E|Fungi Dikarya                      | >gi|154314660|ref|XP_001556654.1| hypothetical protein BC1G_04039 [Botryotinia fuckeliana B05.10]
156053275 69..296    |Sclerotinia sclerotiorum 1980 UF-70           |E|Fungi Dikarya                      | >gi|156053275|ref|XP_001592564.1| hypothetical protein SS1G_06805 [Sclerotinia sclerotiorum 1980]
156841549 78..305    |Vanderwaltozyma polyspora DSM 70294           |E|Fungi Dikarya                      | >gi|156841549|ref|XP_001644147.1| hypothetical protein Kpol_1053p26 [Vanderwaltozyma polyspora DSM 70294]
169609615 67..294    |Phaeosphaeria nodorum SN15                    |E|Fungi Dikarya                      | >gi|169609615|ref|XP_001798226.1| hypothetical protein SNOG_07900 [Phaeosphaeria nodorum SN15]
169624748 70..297    |Phaeosphaeria nodorum SN15                    |E|Fungi Dikarya                      | >gi|169624748|ref|XP_001805779.1| hypothetical protein SNOG_15635 [Phaeosphaeria nodorum SN15]
169770593 74..301    |Aspergillus oryzae RIB40                      |E|Fungi Dikarya                      | >gi|169770593|ref|XP_001819766.1| hypothetical protein [Aspergillus oryzae RIB40]
238486970 51..265    |Aspergillus flavus NRRL3357                   |E|Fungi Dikarya                      | >gi|238486970|ref|XP_002374723.1| hemolysin-III channel protein Izh2, putative [Aspergillus flavus NRRL3357]
170099766 71..298    |Laccaria bicolor S238N-H82                    |E|Fungi Dikarya                      | >gi|170099766|ref|XP_001881101.1| predicted protein [Laccaria bicolor S238N-H82]
189204808 63..290    |Pyrenophora tritici-repentis Pt-1C-BFP        |E|Fungi Dikarya                      | >gi|189204808|ref|XP_001938739.1| adiponectin receptor protein 1 [Pyrenophora tritici-repentis Pt-1C-BFP]
212527054 79..306    |Penicillium marneffei ATCC 18224              |E|Fungi Dikarya                      | >gi|212527054|ref|XP_002143684.1| hemolysin-III channel protein Izh2, putative [Penicillium marneffei ATCC 18224]
212527056 141..368   |Penicillium marneffei ATCC 18224              |E|Fungi Dikarya                      | >gi|212527056|ref|XP_002143685.1| hemolysin-III channel protein Izh2, putative [Penicillium marneffei ATCC 18224]
239606869 83..310    |Ajellomyces dermatitidis ER-3                 |E|Fungi Dikarya                      | >gi|239606869|gb|EEQ83856.1| hemolysin-III channel protein Izh2 [Ajellomyces dermatitidis ER-3]
261191248 83..310    |Ajellomyces dermatitidis SLH14081             |E|Fungi Dikarya                      | >gi|261191248|ref|XP_002622032.1| hemolysin-III channel protein Izh2 [Ajellomyces dermatitidis SLH14081]
240275467 62..289    |Ajellomyces capsulatus H143                   |E|Fungi Dikarya                      | >gi|240275467|gb|EER38981.1| hemolysin-III family protein [Ajellomyces capsulatus H143]
225561859 63..289    |Ajellomyces capsulatus G186AR                 |E|Fungi Dikarya                      | >gi|225561859|gb|EEH10139.1| hemolysin-III family protein [Ajellomyces capsulatus G186AR]
242782435 65..292    |Talaromyces stipitatus ATCC 10500             |E|Fungi Dikarya                      | >gi|242782435|ref|XP_002479998.1| hemolysin-III channel protein Izh2, putative [Talaromyces stipitatus ATCC 10500]
242782440 79..306    |Talaromyces stipitatus ATCC 10500             |E|Fungi Dikarya                      | >gi|242782440|ref|XP_002479999.1| hemolysin-III channel protein Izh2, putative [Talaromyces stipitatus ATCC 10500]
255931867 79..306    |Penicillium chrysogenum Wisconsin 54-1255     |E|Fungi Dikarya                      | >gi|255931867|ref|XP_002557490.1| Pc12g06480 [Penicillium chrysogenum Wisconsin 54-1255]
255936851 72..299    |Penicillium chrysogenum Wisconsin 54-1255     |E|Fungi Dikarya                      | >gi|255936851|ref|XP_002559452.1| Pc13g10300 [Penicillium chrysogenum Wisconsin 54-1255]
255945465 71..298    |Penicillium chrysogenum Wisconsin 54-1255     |E|Fungi Dikarya                      | >gi|255945465|ref|XP_002563500.1| Pc20g10060 [Penicillium chrysogenum Wisconsin 54-1255]
256271421 62..289    |Saccharomyces cerevisiae JAY291               |E|Fungi Dikarya                      | >gi|256271421|gb|EEU06482.1| Izh4p [Saccharomyces cerevisiae JAY291]
259149384 62..289    |Saccharomyces cerevisiae EC1118               |E|Fungi Dikarya                      | >gi|259149384|emb|CAY86188.1| Izh4p [Saccharomyces cerevisiae EC1118]
51012627  62..289    |Saccharomyces cerevisiae                      |E|Fungi Dikarya                      | >gi|51012627|gb|AAT92607.1| YOL101C [Saccharomyces cerevisiae]
6324471   62..289    |Saccharomyces cerevisiae S288c                |E|Fungi Dikarya                      | >gi|6324471|ref|NP_014540.1| Izh4p [Saccharomyces cerevisiae S288c]
258563712 71..298    |Uncinocarpus reesii 1704                      |E|Fungi Dikarya                      | >gi|258563712|ref|XP_002582601.1| conserved hypothetical protein [Uncinocarpus reesii 1704]
260798725 36..263    |Branchiostoma floridae                        |E|Metazoa Chordata                   | >gi|260798725|ref|XP_002594350.1| hypothetical protein BRAFLDRAFT_208926 [Branchiostoma floridae]
261352298 106..333   |Verticillium albo-atrum VaMs.102              |E|Fungi Dikarya                      | >gi|261352298|gb|EEY14726.1| adiponectin receptor protein [Verticillium albo-atrum VaMs.102]
289619302 92..319    |Sordaria macrospora                           |E|Fungi Dikarya                      | >gi|289619302|emb|CBI54179.1| unnamed protein product [Sordaria macrospora]
291179658 86..313    |Arthroderma benhamiae CBS 112371              |E|Fungi Dikarya                      | >gi|291179658|gb|EFE35444.1| hypothetical protein ARB_05486 [Arthroderma benhamiae CBS 112371]
291185213 85..312    |Trichophyton verrucosum HKI 0517              |E|Fungi Dikarya                      | >gi|291185213|gb|EFE40701.1| hypothetical protein TRV_04563 [Trichophyton verrucosum HKI 0517]
291399552 65..292    |Oryctolagus cuniculus                         |E|Metazoa Chordata                   | >gi|291399552|ref|XP_002716154.1| PREDICTED: progestin and adipoQ receptor family member VII [Oryctolagus cuniculus]
296824424 75..302    |Arthroderma otae CBS 113480                   |E|Fungi Dikarya                      | >gi|296824424|ref|XP_002850655.1| adiponectin receptor protein 1 [Arthroderma otae CBS 113480]
39940336  79..306    |Magnaporthe oryzae 70-15                      |E|Fungi Dikarya                      | >gi|39940336|ref|XP_359705.1| hypothetical protein MGG_05072 [Magnaporthe oryzae 70-15]
45185686  87..314    |Ashbya gossypii ATCC 10895                    |E|Fungi Dikarya                      | >gi|45185686|ref|NP_983402.1| ACL002Cp [Ashbya gossypii ATCC 10895]
45198855  70..297    |Ashbya gossypii ATCC 10895                    |E|Fungi Dikarya                      | >gi|45198855|ref|NP_985884.1| AFR337Wp [Ashbya gossypii ATCC 10895]
50286739  73..300    |Candida glabrata CBS 138                      |E|Fungi Dikarya                      | >gi|50286739|ref|XP_445799.1| hypothetical protein [Candida glabrata CBS 138]
50288265  74..301    |Candida glabrata CBS 138                      |E|Fungi Dikarya                      | >gi|50288265|ref|XP_446561.1| hypothetical protein [Candida glabrata CBS 138]
66800759  324..551   |Dictyostelium discoideum AX4                  |E|Amoebozoa Mycetozoa                | >gi|66800759|ref|XP_629305.1| hypothetical protein DDB_G0293072 [Dictyostelium discoideum AX4]
67537524  75..302    |Aspergillus nidulans FGSC A4                  |E|Fungi Dikarya                      | >gi|67537524|ref|XP_662536.1| hypothetical protein AN4932.2 [Aspergillus nidulans FGSC A4]
72167680  84..311    |Strongylocentrotus purpuratus                 |E|Metazoa Echinodermata              | >gi|72167680|ref|XP_788437.1| PREDICTED: similar to putative membrane progestin receptor gamma [Strongylocentrotus purpuratus]
74149667  65..292    |Mus musculus                                  |E|Metazoa Chordata                   | >gi|74149667|dbj|BAE36452.1| unnamed protein product [Mus musculus]
10437529  12..238    |Homo sapiens                                  |E|Metazoa Chordata                   | >gi|10437529|dbj|BAB15062.1| unnamed protein product [Homo sapiens]
109095059 140..366   |Macaca mulatta                                |E|Metazoa Chordata                   | >gi|109095059|ref|XP_001096853.1| PREDICTED: adiponectin receptor protein 2-like isoform 3 [Macaca mulatta]
126340241 141..367   |Monodelphis domestica                         |E|Metazoa Chordata                   | >gi|126340241|ref|XP_001373467.1| PREDICTED: adiponectin receptor protein 2 [Monodelphis domestica]
149049603 140..366   |Rattus norvegicus                             |E|Metazoa Chordata                   | >gi|149049603|gb|EDM02057.1| rCG30257 [Rattus norvegicus]
149566040 210..436   |Ornithorhynchus anatinus                      |E|Metazoa Chordata                   | >gi|149566040|ref|XP_001520804.1| PREDICTED: similar to Adiponectin receptor 2 [Ornithorhynchus anatinus]
172052588 140..366   |Macaca fuscata                                |E|Metazoa Chordata                   | >gi|172052588|dbj|BAG16754.1| adiponectin receptor 2 [Macaca fuscata]
255308920 140..366   |Equus caballus                                |E|Metazoa Chordata                   | >gi|255308920|ref|NP_001157302.1| adiponectin receptor protein 2 [Equus caballus]
281349607 140..366   |Ailuropoda melanoleuca                        |E|Metazoa Chordata                   | >gi|281349607|gb|EFB25191.1| hypothetical protein PANDA_001997 [Ailuropoda melanoleuca]
291392867 140..366   |Oryctolagus cuniculus                         |E|Metazoa Chordata                   | >gi|291392867|ref|XP_002712820.1| PREDICTED: adiponectin receptor 1 [Oryctolagus cuniculus]
296211088 140..366   |Callithrix jacchus                            |E|Metazoa Chordata                   | >gi|296211088|ref|XP_002807121.1| PREDICTED: LOW QUALITY PROTEIN: adiponectin receptor protein 2-like [Callithrix jacchus]
297690786 140..366   |Pongo abelii                                  |E|Metazoa Chordata                   | >gi|297690786|ref|XP_002822789.1| PREDICTED: adiponectin receptor protein 2-like [Pongo abelii]
38261973  140..366   |Homo sapiens                                  |E|Metazoa Chordata                   | >gi|38261973|ref|NP_078827.2| adiponectin receptor protein 2 [Homo sapiens]
39841016  140..366   |Mus musculus                                  |E|Metazoa Chordata                   | >gi|39841016|ref|NP_932102.2| adiponectin receptor protein 2 [Mus musculus]
55742866  140..366   |Sus scrofa                                    |E|Metazoa Chordata                   | >gi|55742866|ref|NP_001007193.1| adiponectin receptor protein 2 [Sus scrofa]
73997558  140..366   |Canis lupus familiaris                        |E|Metazoa Chordata                   | >gi|73997558|ref|XP_854029.1| PREDICTED: similar to adiponectin receptor 2 isoform 2 [Canis familiaris]
83816891  140..366   |Rattus norvegicus                             |E|Metazoa Chordata                   | >gi|83816891|ref|NP_001033068.1| adiponectin receptor protein 2 [Rattus norvegicus]
94966807  140..366   |Bos taurus                                    |E|Metazoa Chordata                   | >gi|94966807|ref|NP_001035589.1| adiponectin receptor protein 2 [Bos taurus]
95768601  24..250    |Bos taurus                                    |E|Metazoa Chordata                   | >gi|95768601|gb|ABF57367.1| adiponectin receptor 2 [Bos taurus]
40805094  1..205     |Sus scrofa                                    |E|Metazoa Chordata                   | >gi|40805094|gb|AAR91795.1| adiponectin receptor 2 [Sus scrofa]
40850655  1..119     |Bos taurus                                    |E|Metazoa Chordata                   | >gi|40850655|gb|AAR96049.1| adiponectin receptor-2 [Bos taurus]
109018829 129..355   |Macaca mulatta                                |E|Metazoa Chordata                   | >gi|109018829|ref|XP_001105805.1| PREDICTED: adiponectin receptor protein 1-like isoform 5 [Macaca mulatta]
119611855 53..279    |Homo sapiens                                  |E|Metazoa Chordata                   | >gi|119611855|gb|EAW91449.1| adiponectin receptor 1, isoform CRA_a [Homo sapiens]
124111425 129..355   |Anas platyrhynchos                            |E|Metazoa Chordata                   | >gi|124111425|gb|ABI49513.2| adiponectin receptor 1 [Anas platyrhynchos]
126306680 129..355   |Monodelphis domestica                         |E|Metazoa Chordata                   | >gi|126306680|ref|XP_001364335.1| PREDICTED: adiponectin receptor protein 1 [Monodelphis domestica]
147899025 138..364   |Xenopus laevis                                |E|Metazoa Chordata                   | >gi|147899025|ref|NP_001089438.1| adiponectin receptor 1 [Xenopus laevis]
149058570 129..355   |Rattus norvegicus                             |E|Metazoa Chordata                   | >gi|149058570|gb|EDM09727.1| adiponectin receptor 1, isoform CRA_a [Rattus norvegicus]
149743860 129..355   |Equus caballus                                |E|Metazoa Chordata                   | >gi|149743860|ref|XP_001496089.1| PREDICTED: similar to adiponectin receptor 1 [Equus caballus]
193785556 129..355   |Homo sapiens                                  |E|Metazoa Chordata                   | >gi|193785556|dbj|BAG50922.1| unnamed protein product [Homo sapiens]
197927374 129..355   |Felis catus                                   |E|Metazoa Chordata                   | >gi|197927374|ref|NP_001128153.1| adiponectin receptor protein 1 [Felis catus]
21361519  129..355   |Homo sapiens                                  |E|Metazoa Chordata                   | >gi|21361519|ref|NP_057083.2| adiponectin receptor protein 1 [Homo sapiens]
224085029 132..358   |Taeniopygia guttata                           |E|Metazoa Chordata                   | >gi|224085029|ref|XP_002198547.1| PREDICTED: adiponectin receptor 1 [Taeniopygia guttata]
281346582 129..355   |Ailuropoda melanoleuca                        |E|Metazoa Chordata                   | >gi|281346582|gb|EFB22166.1| hypothetical protein PANDA_014997 [Ailuropoda melanoleuca]
291402613 129..355   |Oryctolagus cuniculus                         |E|Metazoa Chordata                   | >gi|291402613|ref|XP_002717631.1| PREDICTED: adiponectin receptor 1 [Oryctolagus cuniculus]
296230446 134..360   |Callithrix jacchus                            |E|Metazoa Chordata                   | >gi|296230446|ref|XP_002760703.1| PREDICTED: adiponectin receptor protein 1-like [Callithrix jacchus]
38259186  129..355   |Mus musculus                                  |E|Metazoa Chordata                   | >gi|38259186|ref|NP_082596.2| adiponectin receptor protein 1 [Mus musculus]
40805092  103..329   |Sus scrofa                                    |E|Metazoa Chordata                   | >gi|40805092|gb|AAR91794.1| adiponectin receptor 1 [Sus scrofa]
46485456  129..355   |Rattus norvegicus                             |E|Metazoa Chordata                   | >gi|46485456|ref|NP_997470.1| adiponectin receptor protein 1 [Rattus norvegicus]
4929559   129..355   |Homo sapiens                                  |E|Metazoa Chordata                   | >gi|4929559|gb|AAD34040.1|AF151803_1 CGI-45 protein [Homo sapiens]
55742864  129..355   |Sus scrofa                                    |E|Metazoa Chordata                   | >gi|55742864|ref|NP_001007194.1| adiponectin receptor protein 1 [Sus scrofa]
62896565  129..355   |Homo sapiens                                  |E|Metazoa Chordata                   | >gi|62896565|dbj|BAD96223.1| adiponectin receptor 1 variant [Homo sapiens]
71895887  129..355   |Gallus gallus                                 |E|Metazoa Chordata                   | >gi|71895887|ref|NP_001026198.1| adiponectin receptor protein 1 [Gallus gallus]
73487233  129..355   |Rattus norvegicus                             |E|Metazoa Chordata                   | >gi|73487233|gb|AAZ76713.1| adiponectin receptor 1 [Rattus norvegicus]
73960225  129..355   |Canis lupus familiaris                        |E|Metazoa Chordata                   | >gi|73960225|ref|XP_848356.1| PREDICTED: similar to adiponectin receptor 1 isoform 2 [Canis familiaris]
74150719  129..355   |Mus musculus                                  |E|Metazoa Chordata                   | >gi|74150719|dbj|BAE25493.1| unnamed protein product [Mus musculus]
74207594  129..355   |Mus musculus                                  |E|Metazoa Chordata                   | >gi|74207594|dbj|BAE40044.1| unnamed protein product [Mus musculus]
77404219  129..355   |Bos taurus                                    |E|Metazoa Chordata                   | >gi|77404219|ref|NP_001029227.1| adiponectin receptor protein 1 [Bos taurus]
90085214  12..238    |Macaca fascicularis                           |E|Metazoa Chordata                   | >gi|90085214|dbj|BAE91348.1| unnamed protein product [Macaca fascicularis]
12849860  129..352   |Mus musculus                                  |E|Metazoa Chordata                   | >gi|12849860|dbj|BAB28509.1| unnamed protein product [Mus musculus]
56609208  2..203     |Sus scrofa                                    |E|Metazoa Chordata                   | >gi|56609208|gb|AAW03193.1| adiponectin receptor 1 [Sus scrofa]
115752476 78..304    |Strongylocentrotus purpuratus                 |E|Metazoa Echinodermata              | >gi|115752476|ref|XP_781396.2| PREDICTED: similar to Paqr5 protein, partial [Strongylocentrotus purpuratus]
119467143 73..299    |Neosartorya fischeri NRRL 181                 |E|Fungi Dikarya                      | >gi|119467143|ref|XP_001257378.1| Haemolysin-III related protein [Neosartorya fischeri NRRL 181]
147902276 135..361   |Xenopus laevis                                |E|Metazoa Chordata                   | >gi|147902276|ref|NP_001080721.1| adiponectin receptor 1 [Xenopus laevis]
149606405 129..355   |Ornithorhynchus anatinus                      |E|Metazoa Chordata                   | >gi|149606405|ref|XP_001515926.1| PREDICTED: similar to Adiponectin receptor 1 [Ornithorhynchus anatinus]
56118268  133..359   |Xenopus (Silurana) tropicalis                 |E|Metazoa Chordata                   | >gi|56118268|ref|NP_001007928.1| adiponectin receptor 1 [Xenopus (Silurana) tropicalis]
147905939 139..365   |Xenopus laevis                                |E|Metazoa Chordata                   | >gi|147905939|ref|NP_001087336.1| MGC85478 protein [Xenopus laevis]
148231558 141..367   |Xenopus laevis                                |E|Metazoa Chordata                   | >gi|148231558|ref|NP_001087571.1| adiponectin receptor 2 [Xenopus laevis]
158253874 114..340   |Danio rerio                                   |E|Metazoa Chordata                   | >gi|158253874|gb|AAI54262.1| Adipor2 protein [Danio rerio]
47209855  118..344   |Tetraodon nigroviridis                        |E|Metazoa Chordata                   | >gi|47209855|emb|CAG12238.1| unnamed protein product [Tetraodon nigroviridis]
70887627  113..339   |Danio rerio                                   |E|Metazoa Chordata                   | >gi|70887627|ref|NP_001020677.1| adiponectin receptor protein 2 [Danio rerio]
166034431 54..211    |Danio rerio                                   |E|Metazoa Chordata                   | >gi|166034431|gb|ABY78992.1| adiponectin receptor 2 [Danio rerio]
166034433 125..351   |Danio rerio                                   |E|Metazoa Chordata                   | >gi|166034433|gb|ABY78993.1| adiponectin receptor 1b [Danio rerio]
169154357 131..357   |Danio rerio                                   |E|Metazoa Chordata                   | >gi|169154357|emb|CAQ14260.1| adiponectin receptor 1b [Danio rerio]
47087283  131..357   |Danio rerio                                   |E|Metazoa Chordata                   | >gi|47087283|ref|NP_998665.1| adiponectin receptor protein 1 [Danio rerio]
167516160 29..255    |Monosiga brevicollis MX1                      |E|Choanoflagellida Codonosigidae     | >gi|167516160|ref|XP_001742421.1| hypothetical protein [Monosiga brevicollis MX1]
198427082 148..374   |Ciona intestinalis                            |E|Metazoa Chordata                   | >gi|198427082|ref|XP_002129916.1| PREDICTED: similar to adiponectin receptor 2 [Ciona intestinalis]
213514328 125..351   |Salmo salar                                   |E|Metazoa Chordata                   | >gi|213514328|ref|NP_001133596.1| Adiponectin receptor protein 1 [Salmo salar]
223648194 125..351   |Salmo salar                                   |E|Metazoa Chordata                   | >gi|223648194|gb|ACN10855.1| Adiponectin receptor protein 1 [Salmo salar]
47222862  72..298    |Tetraodon nigroviridis                        |E|Metazoa Chordata                   | >gi|47222862|emb|CAF96529.1| unnamed protein product [Tetraodon nigroviridis]
224096384 141..367   |Taeniopygia guttata                           |E|Metazoa Chordata                   | >gi|224096384|ref|XP_002197931.1| PREDICTED: adiponectin receptor 2 [Taeniopygia guttata]
56119026  139..365   |Gallus gallus                                 |E|Metazoa Chordata                   | >gi|56119026|ref|NP_001007855.1| adiponectin receptor protein 2 [Gallus gallus]
67782279  139..365   |Gallus gallus                                 |E|Metazoa Chordata                   | >gi|67782279|gb|AAY81970.1| adiponectin receptor 2 [Gallus gallus]
85719404  139..365   |Anas platyrhynchos                            |E|Metazoa Chordata                   | >gi|85719404|gb|ABC75392.1| adiponectin receptor 2 [Anas platyrhynchos]
148667194 146..286   |Mus musculus                                  |E|Metazoa Chordata                   | >gi|148667194|gb|EDK99610.1| adiponectin receptor 2, isoform CRA_b [Mus musculus]
26333389  140..280   |Mus musculus                                  |E|Metazoa Chordata                   | >gi|26333389|dbj|BAC30412.1| unnamed protein product [Mus musculus]
26338227  140..280   |Mus musculus                                  |E|Metazoa Chordata                   | >gi|26338227|dbj|BAC32799.1| unnamed protein product [Mus musculus]
224154828 1..117     |Taeniopygia guttata                           |E|Metazoa Chordata                   | >gi|224154828|ref|XP_002199024.1| PREDICTED: similar to adiponectin receptor 2, partial [Taeniopygia guttata]
225681099 71..297    |Paracoccidioides brasiliensis Pb03            |E|Fungi Dikarya                      | >gi|225681099|gb|EEH19383.1| adiponectin receptor protein [Paracoccidioides brasiliensis Pb03]
295673448 76..264    |Paracoccidioides brasiliensis Pb01            |E|Fungi Dikarya                      | >gi|295673448|ref|XP_002797270.1| conserved hypothetical protein [Paracoccidioides brasiliensis Pb01]
226292200 129..303   |Paracoccidioides brasiliensis Pb18            |E|Fungi Dikarya                      | >gi|226292200|gb|EEH47620.1| adiponectin receptor protein [Paracoccidioides brasiliensis Pb18]
254579985 78..304    |Zygosaccharomyces rouxii CBS 732              |E|Fungi Dikarya                      | >gi|254579985|ref|XP_002495978.1| ZYRO0C07612p [Zygosaccharomyces rouxii]
255712829 77..303    |Lachancea thermotolerans CBS 6340             |E|Fungi Dikarya                      | >gi|255712829|ref|XP_002552697.1| KLTH0C11044p [Lachancea thermotolerans]
259482024 38..264    |Aspergillus nidulans FGSC A4                  |E|Fungi Dikarya                      | >gi|259482024|tpe|CBF76105.1| TPA: hypothetical protein ANIA_10630 [Aspergillus nidulans FGSC A4]
260816356 59..285    |Branchiostoma floridae                        |E|Metazoa Chordata                   | >gi|260816356|ref|XP_002602937.1| hypothetical protein BRAFLDRAFT_62482 [Branchiostoma floridae]
260816354 73..222    |Branchiostoma floridae                        |E|Metazoa Chordata                   | >gi|260816354|ref|XP_002602936.1| hypothetical protein BRAFLDRAFT_107807 [Branchiostoma floridae]
261357754 78..304    |Verticillium albo-atrum VaMs.102              |E|Fungi Dikarya                      | >gi|261357754|gb|EEY20182.1| adiponectin receptor protein [Verticillium albo-atrum VaMs.102]
109074375 64..289    |Macaca mulatta                                |E|Metazoa Chordata                   | >gi|109074375|ref|XP_001091757.1| PREDICTED: progestin and adipoQ receptor family member 3 isoform 2 [Macaca mulatta]
119894075 64..289    |Bos taurus                                    |E|Metazoa Chordata                   | >gi|119894075|ref|XP_614798.3| PREDICTED: CG7530-like [Bos taurus]
158255900 64..289    |Homo sapiens                                  |E|Metazoa Chordata                   | >gi|158255900|dbj|BAF83921.1| unnamed protein product [Homo sapiens]
194209038 179..404   |Equus caballus                                |E|Metazoa Chordata                   | >gi|194209038|ref|XP_001492120.2| PREDICTED: similar to Progestin and adipoQ receptor family member 3 (Progestin and adipoQ receptor family member III) [Equus caballus]
281346822 64..289    |Ailuropoda melanoleuca                        |E|Metazoa Chordata                   | >gi|281346822|gb|EFB22406.1| hypothetical protein PANDA_000282 [Ailuropoda melanoleuca]
297673840 64..289    |Pongo abelii                                  |E|Metazoa Chordata                   | >gi|297673840|ref|XP_002814956.1| PREDICTED: progestin and adipoQ receptor family member 3-like [Pongo abelii]
38018647  64..289    |Homo sapiens                                  |E|Metazoa Chordata                   | >gi|38018647|gb|AAR08369.1| progestin and adipoQ receptor family member III [Homo sapiens]
38259216  64..289    |Mus musculus                                  |E|Metazoa Chordata                   | >gi|38259216|ref|NP_940814.1| progestin and adipoQ receptor family member 3 [Mus musculus]
58865642  64..289    |Rattus norvegicus                             |E|Metazoa Chordata                   | >gi|58865642|ref|NP_001012033.1| progestin and adipoQ receptor family member 3 [Rattus norvegicus]
66272333  64..289    |Mus musculus                                  |E|Metazoa Chordata                   | >gi|66272333|gb|AAH96380.1| Paqr3 protein [Mus musculus]
67969607  64..289    |Macaca fascicularis                           |E|Metazoa Chordata                   | >gi|67969607|dbj|BAE01152.1| unnamed protein product [Macaca fascicularis]
74002011  129..354   |Canis lupus familiaris                        |E|Metazoa Chordata                   | >gi|74002011|ref|XP_544942.2| PREDICTED: similar to Progestin and adipoQ receptor family member III [Canis familiaris]
74226044  64..289    |Mus musculus                                  |E|Metazoa Chordata                   | >gi|74226044|dbj|BAE28767.1| unnamed protein product [Mus musculus]
94158915  64..289    |Homo sapiens                                  |E|Metazoa Chordata                   | >gi|94158915|ref|NP_001035292.1| progestin and adipoQ receptor family member 3 [Homo sapiens]
114594063 64..267    |Pan troglodytes                               |E|Metazoa Chordata                   | >gi|114594063|ref|XP_526575.2| PREDICTED: progestin and adipoQ receptor family member III isoform 3 [Pan troglodytes]
28703706  64..264    |Homo sapiens                                  |E|Metazoa Chordata                   | >gi|28703706|gb|AAH47510.1| PAQR3 protein [Homo sapiens]
45708699  64..264    |Homo sapiens                                  |E|Metazoa Chordata                   | >gi|45708699|gb|AAH31256.1| PAQR3 protein [Homo sapiens]
193786288 2..199     |Homo sapiens                                  |E|Metazoa Chordata                   | >gi|193786288|dbj|BAG51571.1| unnamed protein product [Homo sapiens]
119626243 1..171     |Homo sapiens                                  |E|Metazoa Chordata                   | >gi|119626243|gb|EAX05838.1| progestin and adipoQ receptor family member III, isoform CRA_c [Homo sapiens]
114594061 64..233    |Pan troglodytes                               |E|Metazoa Chordata                   | >gi|114594061|ref|XP_001145409.1| PREDICTED: hypothetical protein isoform 1 [Pan troglodytes]
119626242 64..233    |Homo sapiens                                  |E|Metazoa Chordata                   | >gi|119626242|gb|EAX05837.1| progestin and adipoQ receptor family member III, isoform CRA_b [Homo sapiens]
115390777 253..478   |Aspergillus terreus NIH2624                   |E|Fungi Dikarya                      | >gi|115390777|ref|XP_001212893.1| conserved hypothetical protein [Aspergillus terreus NIH2624]
115752474 51..276    |Strongylocentrotus purpuratus                 |E|Metazoa Echinodermata              | >gi|115752474|ref|XP_001200934.1| PREDICTED: similar to putative membrane progestin receptor gamma [Strongylocentrotus purpuratus]
116182504 251..476   |Chaetomium globosum CBS 148.51                |E|Fungi Dikarya                      | >gi|116182504|ref|XP_001221101.1| hypothetical protein CHGG_01880 [Chaetomium globosum CBS 148.51]
119178282 255..480   |Coccidioides immitis RS                       |E|Fungi Dikarya                      | >gi|119178282|ref|XP_001240828.1| hypothetical protein CIMG_07991 [Coccidioides immitis RS]
240104811 244..469   |Coccidioides posadasii C735 delta SOWgp       |E|Fungi Dikarya                      | >gi|240104811|gb|EER23006.1| Hemolysin-III related family protein [Coccidioides posadasii C735 delta SOWgp]
258577155 248..473   |Uncinocarpus reesii 1704                      |E|Fungi Dikarya                      | >gi|258577155|ref|XP_002542759.1| conserved hypothetical protein [Uncinocarpus reesii 1704]
119468052 255..480   |Neosartorya fischeri NRRL 181                 |E|Fungi Dikarya                      | >gi|119468052|ref|XP_001257832.1| IZH family channel protein (Izh3), putative [Neosartorya fischeri NRRL 181]
121710030 252..477   |Aspergillus clavatus NRRL 1                   |E|Fungi Dikarya                      | >gi|121710030|ref|XP_001272631.1| IZH family channel protein (Izh3), putative [Aspergillus clavatus NRRL 1]
159124165 255..480   |Aspergillus fumigatus A1163                   |E|Fungi Dikarya                      | >gi|159124165|gb|EDP49283.1| IZH family channel protein (Izh3), putative [Aspergillus fumigatus A1163]
169768042 257..482   |Aspergillus oryzae RIB40                      |E|Fungi Dikarya                      | >gi|169768042|ref|XP_001818492.1| hypothetical protein [Aspergillus oryzae RIB40]
238484987 257..482   |Aspergillus flavus NRRL3357                   |E|Fungi Dikarya                      | >gi|238484987|ref|XP_002373732.1| IZH family channel protein (Izh3), putative [Aspergillus flavus NRRL3357]
70991521  255..480   |Aspergillus fumigatus Af293                   |E|Fungi Dikarya                      | >gi|70991521|ref|XP_750609.1| IZH family channel protein (Izh3) [Aspergillus fumigatus Af293]
126331047 64..289    |Monodelphis domestica                         |E|Metazoa Chordata                   | >gi|126331047|ref|XP_001365148.1| PREDICTED: progestin and adipoQ receptor family member 3-like [Monodelphis domestica]
134117518 488..713   |Cryptococcus neoformans var. neoformans B-3501A|E|Fungi Dikarya                      | >gi|134117518|ref|XP_772530.1| hypothetical protein CNBL0100 [Cryptococcus neoformans var. neoformans B-3501A]
58270096  410..631   |Cryptococcus neoformans var. neoformans JEC21 |E|Fungi Dikarya                      | >gi|58270096|ref|XP_572204.1| endoplasmic reticulum protein [Cryptococcus neoformans var. neoformans JEC21]
145238710 255..480   |Aspergillus niger CBS 513.88                  |E|Fungi Dikarya                      | >gi|145238710|ref|XP_001392002.1| IZH family channel protein (Izh3) [Aspergillus niger CBS 513.88]
148227402 64..289    |Xenopus laevis                                |E|Metazoa Chordata                   | >gi|148227402|ref|NP_001084789.1| progestin and adipoQ receptor family member III [Xenopus laevis]
213625769 64..289    |Xenopus (Silurana) tropicalis                 |E|Metazoa Chordata                   | >gi|213625769|gb|AAI71300.1| progestin and adipoQ receptor family member III [Xenopus (Silurana) tropicalis]
62859303  64..289    |Xenopus (Silurana) tropicalis                 |E|Metazoa Chordata                   | >gi|62859303|ref|NP_001016135.1| progestin and adipoQ receptor family member III [Xenopus (Silurana) tropicalis]
154273310 255..480   |Ajellomyces capsulatus NAm1                   |E|Fungi Dikarya                      | >gi|154273310|ref|XP_001537507.1| conserved hypothetical protein [Ajellomyces capsulatus NAm1]
225555782 255..480   |Ajellomyces capsulatus G186AR                 |E|Fungi Dikarya                      | >gi|225555782|gb|EEH04073.1| hemolysin-III family protein [Ajellomyces capsulatus G186AR]
240279104 255..480   |Ajellomyces capsulatus H143                   |E|Fungi Dikarya                      | >gi|240279104|gb|EER42609.1| hemolysin-III family protein [Ajellomyces capsulatus H143]
154299083 256..481   |Botryotinia fuckeliana B05.10                 |E|Fungi Dikarya                      | >gi|154299083|ref|XP_001549962.1| hypothetical protein BC1G_11854 [Botryotinia fuckeliana B05.10]
156058149 256..481   |Sclerotinia sclerotiorum 1980 UF-70           |E|Fungi Dikarya                      | >gi|156058149|ref|XP_001594998.1| hypothetical protein SS1G_04806 [Sclerotinia sclerotiorum 1980]
154346504 269..494   |Leishmania braziliensis MHOM/BR/75/M2904      |E|Euglenozoa Kinetoplastida          | >gi|154346504|ref|XP_001569189.1| hypothetical protein [Leishmania braziliensis MHOM/BR/75/M2904]
156846778 78..303    |Vanderwaltozyma polyspora DSM 70294           |E|Fungi Dikarya                      | >gi|156846778|ref|XP_001646275.1| hypothetical protein Kpol_1032p9 [Vanderwaltozyma polyspora DSM 70294]
169117900 271..496   |Sporothrix schenckii                          |E|Fungi Dikarya                      | >gi|169117900|gb|ACA43006.1| SSGPRC1 [Sporothrix schenckii]
169601584 201..426   |Phaeosphaeria nodorum SN15                    |E|Fungi Dikarya                      | >gi|169601584|ref|XP_001794214.1| hypothetical protein SNOG_03660 [Phaeosphaeria nodorum SN15]
170093239 342..567   |Laccaria bicolor S238N-H82                    |E|Fungi Dikarya                      | >gi|170093239|ref|XP_001877841.1| predicted protein [Laccaria bicolor S238N-H82]
171695138 253..478   |Podospora anserina S mat+                     |E|Fungi Dikarya                      | >gi|171695138|ref|XP_001912493.1| hypothetical protein [Podospora anserina S mat+]
185134471 76..301    |Oncorhynchus mykiss                           |E|Metazoa Chordata                   | >gi|185134471|ref|NP_001118123.1| membrane progestin receptor beta [Oncorhynchus mykiss]
189193783 248..473   |Pyrenophora tritici-repentis Pt-1C-BFP        |E|Fungi Dikarya                      | >gi|189193783|ref|XP_001933230.1| adiponectin receptor protein 1 [Pyrenophora tritici-repentis Pt-1C-BFP]
19112805  83..308    |Schizosaccharomyces pombe 972h-               |E|Fungi Dikarya                      | >gi|19112805|ref|NP_596013.1| Haemolysin-III family protein [Schizosaccharomyces pombe 972h-]
196015211 75..300    |Trichoplax adhaerens                          |E|Metazoa Placozoa                   | >gi|196015211|ref|XP_002117463.1| hypothetical protein TRIADDRAFT_61447 [Trichoplax adhaerens]
212540958 258..483   |Penicillium marneffei ATCC 18224              |E|Fungi Dikarya                      | >gi|212540958|ref|XP_002150634.1| IZH family channel protein (Izh3), putative [Penicillium marneffei ATCC 18224]
242800082 259..484   |Talaromyces stipitatus ATCC 10500             |E|Fungi Dikarya                      | >gi|242800082|ref|XP_002483514.1| IZH family channel protein (Izh3), putative [Talaromyces stipitatus ATCC 10500]
224049041 64..289    |Taeniopygia guttata                           |E|Metazoa Chordata                   | >gi|224049041|ref|XP_002191733.1| PREDICTED: similar to Progestin and adipoQ receptor family member III [Taeniopygia guttata]
225682249 197..422   |Paracoccidioides brasiliensis Pb03            |E|Fungi Dikarya                      | >gi|225682249|gb|EEH20533.1| conserved hypothetical protein [Paracoccidioides brasiliensis Pb03]
226289623 266..491   |Paracoccidioides brasiliensis Pb18            |E|Fungi Dikarya                      | >gi|226289623|gb|EEH45107.1| adiponectin receptor protein [Paracoccidioides brasiliensis Pb18]
295662130 266..491   |Paracoccidioides brasiliensis Pb01            |E|Fungi Dikarya                      | >gi|295662130|ref|XP_002791619.1| adiponectin receptor protein [Paracoccidioides brasiliensis Pb01]
225718778 187..412   |Caligus clemensi                              |E|Metazoa Arthropoda                 | >gi|225718778|gb|ACO15235.1| ADIPOR-like receptor CG5315 [Caligus clemensi]
255943243 255..480   |Penicillium chrysogenum Wisconsin 54-1255     |E|Fungi Dikarya                      | >gi|255943243|ref|XP_002562390.1| Pc18g05630 [Penicillium chrysogenum Wisconsin 54-1255]
256081505 222..447   |Schistosoma mansoni                           |E|Metazoa Platyhelminthes            | >gi|256081505|ref|XP_002577010.1| adiponectin receptor [Schistosoma mansoni]
256735148 254..479   |Nectria haematococca mpVI 77-13-4             |E|Fungi Dikarya                      | >gi|256735148|gb|EEU48494.1| predicted protein [Nectria haematococca mpVI 77-13-4]
261192023 255..480   |Ajellomyces dermatitidis SLH14081             |E|Fungi Dikarya                      | >gi|261192023|ref|XP_002622419.1| IZH family channel protein [Ajellomyces dermatitidis SLH14081]
261357475 258..483   |Verticillium albo-atrum VaMs.102              |E|Fungi Dikarya                      | >gi|261357475|gb|EEY19903.1| hemolysin-III family protein [Verticillium albo-atrum VaMs.102]
289618014 247..472   |Sordaria macrospora                           |E|Fungi Dikarya                      | >gi|289618014|emb|CBI55591.1| unnamed protein product [Sordaria macrospora]
85117833  247..472   |Neurospora crassa OR74A                       |E|Fungi Dikarya                      | >gi|85117833|ref|XP_965338.1| hypothetical protein NCU03238 [Neurospora crassa OR74A]
291175997 145..370   |Arthroderma benhamiae CBS 112371              |E|Fungi Dikarya                      | >gi|291175997|gb|EFE31799.1| hypothetical protein ARB_01398 [Arthroderma benhamiae CBS 112371]
291187648 380..605   |Trichophyton verrucosum HKI 0517              |E|Fungi Dikarya                      | >gi|291187648|gb|EFE43025.1| hypothetical protein TRV_02218 [Trichophyton verrucosum HKI 0517]
296817753 258..483   |Arthroderma otae CBS 113480                   |E|Fungi Dikarya                      | >gi|296817753|ref|XP_002849213.1| adiponectin receptor protein 1 [Arthroderma otae CBS 113480]
39945394  276..501   |Magnaporthe oryzae 70-15                      |E|Fungi Dikarya                      | >gi|39945394|ref|XP_362234.1| hypothetical protein MGG_04679 [Magnaporthe oryzae 70-15]
46108364  254..479   |Gibberella zeae PH-1                          |E|Fungi Dikarya                      | >gi|46108364|ref|XP_381240.1| hypothetical protein FG01064.1 [Gibberella zeae PH-1]
47206882  119..344   |Tetraodon nigroviridis                        |E|Metazoa Chordata                   | >gi|47206882|emb|CAF96021.1| unnamed protein product [Tetraodon nigroviridis]
67537962  253..478   |Aspergillus nidulans FGSC A4                  |E|Fungi Dikarya                      | >gi|67537962|ref|XP_662755.1| hypothetical protein AN5151.2 [Aspergillus nidulans FGSC A4]
110755949 110..334   |Apis mellifera                                |E|Metazoa Arthropoda                 | >gi|110755949|ref|XP_392336.3| PREDICTED: similar to CG7530-PA, isoform A [Apis mellifera]
153792512 125..349   |Bombyx mori                                   |E|Metazoa Arthropoda                 | >gi|153792512|ref|NP_001093316.1| adiponectin receptor [Bombyx mori]
298399909 106..330   |Heliconius cydno cordula                      |E|Metazoa Arthropoda                 | >gi|298399909|gb|ADI81274.1| adiponectin receptor 2 [Heliconius cydno cordula]
298399929 106..330   |Heliconius cydno cordula                      |E|Metazoa Arthropoda                 | >gi|298399929|gb|ADI81284.1| adiponectin receptor 2 [Heliconius cydno cordula]
298399991 106..330   |Heliconius melpomene melpomene                |E|Metazoa Arthropoda                 | >gi|298399991|gb|ADI81315.1| adiponectin receptor 2 [Heliconius melpomene melpomene]
156360570 34..258    |Nematostella vectensis                        |E|Metazoa Cnidaria                   | >gi|156360570|ref|XP_001625100.1| predicted protein [Nematostella vectensis]
157106662 171..395   |Aedes aegypti                                 |E|Metazoa Arthropoda                 | >gi|157106662|ref|XP_001649426.1| adiponectin receptor [Aedes aegypti]
158292239 143..367   |Anopheles gambiae str. PEST                   |E|Metazoa Arthropoda                 | >gi|158292239|ref|XP_313784.4| AGAP004486-PA [Anopheles gambiae str. PEST]
170032989 183..407   |Culex quinquefasciatus                        |E|Metazoa Arthropoda                 | >gi|170032989|ref|XP_001844362.1| adiponectin receptor protein 2 [Culex quinquefasciatus]
164656250 320..544   |Malassezia globosa CBS 7966                   |E|Fungi Dikarya                      | >gi|164656250|ref|XP_001729253.1| hypothetical protein MGL_3720 [Malassezia globosa CBS 7966]
170580441 143..367   |Brugia malayi                                 |E|Metazoa Nematoda                   | >gi|170580441|ref|XP_001895266.1| ADIPOR-like receptor C43G2.1 [Brugia malayi]
189238157 146..370   |Tribolium castaneum                           |E|Metazoa Arthropoda                 | >gi|189238157|ref|XP_976123.2| PREDICTED: similar to AGAP004486-PA isoform 2 [Tribolium castaneum]
270008824 148..372   |Tribolium castaneum                           |E|Metazoa Arthropoda                 | >gi|270008824|gb|EFA05272.1| hypothetical protein TcasGA2_TC015429 [Tribolium castaneum]
19114118  203..427   |Schizosaccharomyces pombe 972h-               |E|Fungi Dikarya                      | >gi|19114118|ref|NP_593206.1| Haemolysin-III family protein [Schizosaccharomyces pombe 972h-]
193605822 139..363   |Acyrthosiphon pisum                           |E|Metazoa Arthropoda                 | >gi|193605822|ref|XP_001951712.1| PREDICTED: similar to CG5315 CG5315-PA [Acyrthosiphon pisum]
239790795 63..287    |Acyrthosiphon pisum                           |E|Metazoa Arthropoda                 | >gi|239790795|dbj|BAH71934.1| ACYPI001921 [Acyrthosiphon pisum]
193722547 1..122     |Acyrthosiphon pisum                           |E|Metazoa Arthropoda                 | >gi|193722547|ref|XP_001952878.1| PREDICTED: similar to adiponectin receptor protein 2, partial [Acyrthosiphon pisum]
239790797 1..120     |Acyrthosiphon pisum                           |E|Metazoa Arthropoda                 | >gi|239790797|dbj|BAH71935.1| ACYPI001921 [Acyrthosiphon pisum]
193624930 94..318    |Acyrthosiphon pisum                           |E|Metazoa Arthropoda                 | >gi|193624930|ref|XP_001951714.1| PREDICTED: similar to CG7530 CG7530-PA [Acyrthosiphon pisum]
194746319 257..481   |Drosophila ananassae                          |E|Metazoa Arthropoda                 | >gi|194746319|ref|XP_001955628.1| GF16147 [Drosophila ananassae]
194910971 237..461   |Drosophila erecta                             |E|Metazoa Arthropoda                 | >gi|194910971|ref|XP_001982260.1| GG12506 [Drosophila erecta]
195158465 125..349   |Drosophila persimilis                         |E|Metazoa Arthropoda                 | >gi|195158465|ref|XP_002020106.1| GL13682 [Drosophila persimilis]
195331059 238..462   |Drosophila sechellia                          |E|Metazoa Arthropoda                 | >gi|195331059|ref|XP_002032220.1| GM23638 [Drosophila sechellia]
195502628 234..458   |Drosophila yakuba                             |E|Metazoa Arthropoda                 | >gi|195502628|ref|XP_002098307.1| GE24027 [Drosophila yakuba]
195572916 237..461   |Drosophila simulans                           |E|Metazoa Arthropoda                 | >gi|195572916|ref|XP_002104441.1| GD18448 [Drosophila simulans]
198450120 238..462   |Drosophila pseudoobscura pseudoobscura        |E|Metazoa Arthropoda                 | >gi|198450120|ref|XP_001357860.2| GA18798 [Drosophila pseudoobscura pseudoobscura]
24649047  198..422   |Drosophila melanogaster                       |E|Metazoa Arthropoda                 | >gi|24649047|ref|NP_651061.1| CG5315, isoform A [Drosophila melanogaster]
45551946  116..340   |Drosophila melanogaster                       |E|Metazoa Arthropoda                 | >gi|45551946|ref|NP_732759.2| CG5315, isoform B [Drosophila melanogaster]
195055662 194..418   |Drosophila grimshawi                          |E|Metazoa Arthropoda                 | >gi|195055662|ref|XP_001994732.1| GH17396 [Drosophila grimshawi]
195111044 194..418   |Drosophila mojavensis                         |E|Metazoa Arthropoda                 | >gi|195111044|ref|XP_002000089.1| GI22724 [Drosophila mojavensis]
195392391 183..407   |Drosophila virilis                            |E|Metazoa Arthropoda                 | >gi|195392391|ref|XP_002054841.1| GJ24663 [Drosophila virilis]
195454026 192..416   |Drosophila willistoni                         |E|Metazoa Arthropoda                 | >gi|195454026|ref|XP_002074052.1| GK14433 [Drosophila willistoni]
195997689 100..324   |Trichoplax adhaerens                          |E|Metazoa Placozoa                   | >gi|195997689|ref|XP_002108713.1| hypothetical protein TRIADDRAFT_51941 [Trichoplax adhaerens]
213405054 208..432   |Schizosaccharomyces japonicus yFS275          |E|Fungi Dikarya                      | >gi|213405054|ref|XP_002173299.1| hemolysin-III family protein [Schizosaccharomyces japonicus yFS275]
213406529 84..308    |Schizosaccharomyces japonicus yFS275          |E|Fungi Dikarya                      | >gi|213406529|ref|XP_002174036.1| adipor-like receptor izh2 [Schizosaccharomyces japonicus yFS275]
226467458 102..326   |Schistosoma japonicum                         |E|Metazoa Platyhelminthes            | >gi|226467458|emb|CAX69605.1| adiponectin receptor 2 [Schistosoma japonicum]
76154206  102..223   |Schistosoma japonicum                         |E|Metazoa Platyhelminthes            | >gi|76154206|gb|AAX25699.2| SJCHGC05641 protein [Schistosoma japonicum]
229577054 158..382   |Nasonia vitripennis                           |E|Metazoa Arthropoda                 | >gi|229577054|ref|NP_001153422.1| adiponectin receptor 1 [Nasonia vitripennis]
256079167 102..326   |Schistosoma mansoni                           |E|Metazoa Platyhelminthes            | >gi|256079167|ref|XP_002575861.1| adiponectin receptor [Schistosoma mansoni]
256079169 110..334   |Schistosoma mansoni                           |E|Metazoa Platyhelminthes            | >gi|256079169|ref|XP_002575862.1| adiponectin receptor [Schistosoma mansoni]
256727336 69..293    |Nectria haematococca mpVI 77-13-4             |E|Fungi Dikarya                      | >gi|256727336|gb|EEU40696.1| hypothetical protein NECHADRAFT_76108 [Nectria haematococca mpVI 77-13-4]
257206070 112..336   |Schistosoma japonicum                         |E|Metazoa Platyhelminthes            | >gi|257206070|emb|CAX82686.1| adiponectin receptor 2 [Schistosoma japonicum]
268572183 310..534   |Caenorhabditis briggsae                       |E|Metazoa Nematoda                   | >gi|268572183|ref|XP_002641256.1| Hypothetical protein CBG05167 [Caenorhabditis briggsae]
71992838  305..529   |Caenorhabditis elegans                        |E|Metazoa Nematoda                   | >gi|71992838|ref|NP_498148.2| hypothetical protein Y32H12A.5 [Caenorhabditis elegans]
289739833 187..411   |Glossina morsitans morsitans                  |E|Metazoa Arthropoda                 | >gi|289739833|gb|ADD18664.1| putative membrane protein [Glossina morsitans morsitans]
50546563  233..457   |Yarrowia lipolytica CLIB122                   |E|Fungi Dikarya                      | >gi|50546563|ref|XP_500751.1| YALI0B11242p [Yarrowia lipolytica]
66525845  158..382   |Apis mellifera                                |E|Metazoa Arthropoda                 | >gi|66525845|ref|XP_623057.1| PREDICTED: similar to CG5315-PA, isoform A [Apis mellifera]
146104889 268..491   |Leishmania infantum JPCM5                     |E|Euglenozoa Kinetoplastida          | >gi|146104889|ref|XP_001469934.1| hypothetical protein [Leishmania infantum]
157877649 267..490   |Leishmania major strain Friedlin              |E|Euglenozoa Kinetoplastida          | >gi|157877649|ref|XP_001687138.1| hypothetical protein [Leishmania major strain Friedlin]
154320263 52..275    |Botryotinia fuckeliana B05.10                 |E|Fungi Dikarya                      | >gi|154320263|ref|XP_001559448.1| hypothetical protein BC1G_02112 [Botryotinia fuckeliana B05.10]
156337064 89..312    |Nematostella vectensis                        |E|Metazoa Cnidaria                   | >gi|156337064|ref|XP_001619789.1| hypothetical protein NEMVEDRAFT_v1g223823 [Nematostella vectensis]
156408544 89..285    |Nematostella vectensis                        |E|Metazoa Cnidaria                   | >gi|156408544|ref|XP_001641916.1| predicted protein [Nematostella vectensis]
156370240 29..252    |Nematostella vectensis                        |E|Metazoa Cnidaria                   | >gi|156370240|ref|XP_001628379.1| predicted protein [Nematostella vectensis]
156408830 39..262    |Nematostella vectensis                        |E|Metazoa Cnidaria                   | >gi|156408830|ref|XP_001642059.1| predicted protein [Nematostella vectensis]
157137817 125..348   |Aedes aegypti                                 |E|Metazoa Arthropoda                 | >gi|157137817|ref|XP_001664048.1| hypothetical protein AaeL_AAEL013864 [Aedes aegypti]
170054054 113..328   |Culex quinquefasciatus                        |E|Metazoa Arthropoda                 | >gi|170054054|ref|XP_001862953.1| progestin and adipoQ receptor family member III [Culex quinquefasciatus]
158289244 37..260    |Anopheles gambiae str. PEST                   |E|Metazoa Arthropoda                 | >gi|158289244|ref|XP_311000.4| AGAP000144-PA [Anopheles gambiae str. PEST]
168043183 49..272    |Physcomitrella patens subsp. patens           |E|Viridiplantae Streptophyta         | >gi|168043183|ref|XP_001774065.1| predicted protein [Physcomitrella patens subsp. patens]
17539162  196..419   |Caenorhabditis elegans                        |E|Metazoa Nematoda                   | >gi|17539162|ref|NP_500998.1| hypothetical protein C43G2.1 [Caenorhabditis elegans]
268536834 196..418   |Caenorhabditis briggsae                       |E|Metazoa Nematoda                   | >gi|268536834|ref|XP_002633552.1| Hypothetical protein CBG05420 [Caenorhabditis briggsae]
195061215 115..338   |Drosophila grimshawi                          |E|Metazoa Arthropoda                 | >gi|195061215|ref|XP_001995947.1| GH14088 [Drosophila grimshawi]
195113177 122..344   |Drosophila mojavensis                         |E|Metazoa Arthropoda                 | >gi|195113177|ref|XP_002001145.1| GI10620 [Drosophila mojavensis]
195390903 115..337   |Drosophila virilis                            |E|Metazoa Arthropoda                 | >gi|195390903|ref|XP_002054106.1| GJ22972 [Drosophila virilis]
195996175 42..265    |Trichoplax adhaerens                          |E|Metazoa Placozoa                   | >gi|195996175|ref|XP_002107956.1| hypothetical protein TRIADDRAFT_20060 [Trichoplax adhaerens]
198415436 132..355   |Ciona intestinalis                            |E|Metazoa Chordata                   | >gi|198415436|ref|XP_002130860.1| PREDICTED: similar to progestin and adipoQ receptor family member VIII [Ciona intestinalis]
242023406 7..230     |Pediculus humanus corporis                    |E|Metazoa Arthropoda                 | >gi|242023406|ref|XP_002432125.1| conserved hypothetical protein [Pediculus humanus corporis]
242220567 245..468   |Postia placenta Mad-698-R                     |E|Fungi Dikarya                      | >gi|242220567|ref|XP_002476048.1| hypothetical G-protein coupled receptor [Postia placenta Mad-698-R]
260830896 40..263    |Branchiostoma floridae                        |E|Metazoa Chordata                   | >gi|260830896|ref|XP_002610396.1| hypothetical protein BRAFLDRAFT_209279 [Branchiostoma floridae]
270001595 81..304    |Tribolium castaneum                           |E|Metazoa Arthropoda                 | >gi|270001595|gb|EEZ98042.1| hypothetical protein TcasGA2_TC000446 [Tribolium castaneum]
91077522  78..301    |Tribolium castaneum                           |E|Metazoa Arthropoda                 | >gi|91077522|ref|XP_970230.1| PREDICTED: similar to CG7530 CG7530-PA [Tribolium castaneum]
291225602 155..378   |Saccoglossus kowalevskii                      |E|Metazoa Hemichordata               | >gi|291225602|ref|XP_002732790.1| PREDICTED: adiponectin receptor 1-like [Saccoglossus kowalevskii]
296815602 79..302    |Arthroderma otae CBS 113480                   |E|Fungi Dikarya                      | >gi|296815602|ref|XP_002848138.1| hemolysin-III family protein [Arthroderma otae CBS 113480]
70981875  73..296    |Aspergillus fumigatus Af293                   |E|Fungi Dikarya                      | >gi|70981875|ref|XP_746466.1| haemolysin-III family protein [Aspergillus fumigatus Af293]
71002945  429..652   |Ustilago maydis 521                           |E|Fungi Dikarya                      | >gi|71002945|ref|XP_756153.1| hypothetical protein UM00006.1 [Ustilago maydis 521]
71650342  71..294    |Trypanosoma cruzi strain CL Brener            |E|Euglenozoa Kinetoplastida          | >gi|71650342|ref|XP_813871.1| hypothetical protein [Trypanosoma cruzi strain CL Brener]
71651024  71..294    |Trypanosoma cruzi strain CL Brener            |E|Euglenozoa Kinetoplastida          | >gi|71651024|ref|XP_814198.1| hypothetical protein [Trypanosoma cruzi strain CL Brener]
154314909 185..407   |Botryotinia fuckeliana B05.10                 |E|Fungi Dikarya                      | >gi|154314909|ref|XP_001556778.1| predicted protein [Botryotinia fuckeliana B05.10]
194743324 108..330   |Drosophila ananassae                          |E|Metazoa Arthropoda                 | >gi|194743324|ref|XP_001954150.1| GF18132 [Drosophila ananassae]
194900832 111..333   |Drosophila erecta                             |E|Metazoa Arthropoda                 | >gi|194900832|ref|XP_001979959.1| GG21110 [Drosophila erecta]
195328933 111..333   |Drosophila sechellia                          |E|Metazoa Arthropoda                 | >gi|195328933|ref|XP_002031166.1| GM25830 [Drosophila sechellia]
195501531 115..337   |Drosophila yakuba                             |E|Metazoa Arthropoda                 | >gi|195501531|ref|XP_002097835.1| GE26431 [Drosophila yakuba]
195570850 111..333   |Drosophila simulans                           |E|Metazoa Arthropoda                 | >gi|195570850|ref|XP_002103417.1| GD20404 [Drosophila simulans]
195143849 117..339   |Drosophila persimilis                         |E|Metazoa Arthropoda                 | >gi|195143849|ref|XP_002012909.1| GL23669 [Drosophila persimilis]
198451025 117..339   |Drosophila pseudoobscura pseudoobscura        |E|Metazoa Arthropoda                 | >gi|198451025|ref|XP_002137204.1| GA26694 [Drosophila pseudoobscura pseudoobscura]
195444202 119..341   |Drosophila willistoni                         |E|Metazoa Arthropoda                 | >gi|195444202|ref|XP_002069760.1| GK11690 [Drosophila willistoni]
21358277  111..333   |Drosophila melanogaster                       |E|Metazoa Arthropoda                 | >gi|21358277|ref|NP_650387.1| CG7530, isoform A [Drosophila melanogaster]
260828372 49..271    |Branchiostoma floridae                        |E|Metazoa Chordata                   | >gi|260828372|ref|XP_002609137.1| hypothetical protein BRAFLDRAFT_249054 [Branchiostoma floridae]
261333049 70..292    |Trypanosoma brucei gambiense DAL972           |E|Euglenozoa Kinetoplastida          | >gi|261333049|emb|CBH16044.1| adiponectin receptor protein 1 [Trypanosoma brucei gambiense DAL972]
71748198  70..292    |Trypanosoma brucei TREU927                    |E|Euglenozoa Kinetoplastida          | >gi|71748198|ref|XP_823154.1| adiponectin receptor protein 1 [Trypanosoma brucei TREU927]
39956468  18..240    |Magnaporthe oryzae 70-15                      |E|Fungi Dikarya                      | >gi|39956468|ref|XP_364246.1| hypothetical protein MGG_09091 [Magnaporthe oryzae 70-15]
146104893 96..317    |Leishmania infantum JPCM5                     |E|Euglenozoa Kinetoplastida          | >gi|146104893|ref|XP_001469935.1| hypothetical protein [Leishmania infantum]
157877653 96..317    |Leishmania major strain Friedlin              |E|Euglenozoa Kinetoplastida          | >gi|157877653|ref|XP_001687139.1| hypothetical protein [Leishmania major strain Friedlin]
154346512 96..317    |Leishmania braziliensis MHOM/BR/75/M2904      |E|Euglenozoa Kinetoplastida          | >gi|154346512|ref|XP_001569193.1| hypothetical protein [Leishmania braziliensis MHOM/BR/75/M2904]
119493727 54..274    |Neosartorya fischeri NRRL 181                 |E|Fungi Dikarya                      | >gi|119493727|ref|XP_001263950.1| Haemolysin-III related protein [Neosartorya fischeri NRRL 181]
281203559 165..385   |Polysphondylium pallidum PN500                |E|Amoebozoa Mycetozoa                | >gi|281203559|gb|EFA77756.1| hypothetical protein PPL_09254 [Polysphondylium pallidum PN500]
110764275 125..344   |Apis mellifera                                |E|Metazoa Arthropoda                 | >gi|110764275|ref|XP_001122843.1| PREDICTED: similar to CG33203-PC [Apis mellifera]
168066811 108..327   |Physcomitrella patens subsp. patens           |E|Viridiplantae Streptophyta         | >gi|168066811|ref|XP_001785325.1| predicted protein [Physcomitrella patens subsp. patens]
198421340 83..302    |Ciona intestinalis                            |E|Metazoa Chordata                   | >gi|198421340|ref|XP_002122039.1| PREDICTED: similar to progestin and adipoQ receptor family member III [Ciona intestinalis]
198429629 5..207     |Ciona intestinalis                            |E|Metazoa Chordata                   | >gi|198429629|ref|XP_002129020.1| PREDICTED: similar to progestin and adipoQ receptor family member III [Ciona intestinalis]
66803703  150..369   |Dictyostelium discoideum AX4                  |E|Amoebozoa Mycetozoa                | >gi|66803703|ref|XP_635686.1| hypothetical protein DDB_G0290605 [Dictyostelium discoideum AX4]
156398893 120..336   |Nematostella vectensis                        |E|Metazoa Cnidaria                   | >gi|156398893|ref|XP_001638422.1| predicted protein [Nematostella vectensis]
196007352 49..265    |Trichoplax adhaerens                          |E|Metazoa Placozoa                   | >gi|196007352|ref|XP_002113542.1| hypothetical protein TRIADDRAFT_57064 [Trichoplax adhaerens]
221460135 208..424   |Drosophila melanogaster                       |E|Metazoa Arthropoda                 | >gi|221460135|ref|NP_788756.2| CG33203, isoform C [Drosophila melanogaster]
221460137 208..421   |Drosophila melanogaster                       |E|Metazoa Arthropoda                 | >gi|221460137|ref|NP_001138119.1| CG33203, isoform B [Drosophila melanogaster]
195112316 6..207     |Drosophila mojavensis                         |E|Metazoa Arthropoda                 | >gi|195112316|ref|XP_002000720.1| GI10382 [Drosophila mojavensis]
198452375 8..208     |Drosophila pseudoobscura pseudoobscura        |E|Metazoa Arthropoda                 | >gi|198452375|ref|XP_001358741.2| GA17365 [Drosophila pseudoobscura pseudoobscura]
194742774 1..187     |Drosophila ananassae                          |E|Metazoa Arthropoda                 | >gi|194742774|ref|XP_001953876.1| GF17984 [Drosophila ananassae]
195145110 1..187     |Drosophila persimilis                         |E|Metazoa Arthropoda                 | >gi|195145110|ref|XP_002013539.1| GL23352 [Drosophila persimilis]
195449323 1..187     |Drosophila willistoni                         |E|Metazoa Arthropoda                 | >gi|195449323|ref|XP_002072024.1| GK22627 [Drosophila willistoni]
194906733 206..372   |Drosophila erecta                             |E|Metazoa Arthropoda                 | >gi|194906733|ref|XP_001981420.1| GG12049 [Drosophila erecta]
195056099 237..403   |Drosophila grimshawi                          |E|Metazoa Arthropoda                 | >gi|195056099|ref|XP_001994950.1| GH17513 [Drosophila grimshawi]
195341045 208..374   |Drosophila sechellia                          |E|Metazoa Arthropoda                 | >gi|195341045|ref|XP_002037122.1| GM12278 [Drosophila sechellia]
21064539  162..326   |Drosophila melanogaster                       |E|Metazoa Arthropoda                 | >gi|21064539|gb|AAM29499.1| RE52005p [Drosophila melanogaster]
296412010 74..290    |Tuber melanosporum Mel28                      |E|Fungi Dikarya                      | >gi|296412010|ref|XP_002835721.1| hypothetical protein [Tuber melanosporum Mel28]
91092254  66..282    |Tribolium castaneum                           |E|Metazoa Arthropoda                 | >gi|91092254|ref|XP_966929.1| PREDICTED: similar to HL06052p [Tribolium castaneum]
126342579 43..258    |Monodelphis domestica                         |E|Metazoa Chordata                   | >gi|126342579|ref|XP_001371476.1| PREDICTED: progestin and adipoQ receptor family member 4-like [Monodelphis domestica]
189525916 43..258    |Danio rerio                                   |E|Metazoa Chordata                   | >gi|189525916|ref|XP_001920113.1| PREDICTED: progestin and adipoQ receptor family member 4-like [Danio rerio]
47217797  79..294    |Tetraodon nigroviridis                        |E|Metazoa Chordata                   | >gi|47217797|emb|CAG07211.1| unnamed protein product [Tetraodon nigroviridis]
68357724  43..258    |Danio rerio                                   |E|Metazoa Chordata                   | >gi|68357724|ref|XP_693997.1| PREDICTED: progestin and adipoQ receptor family member 4-like isoform 1 [Danio rerio]
148224299 43..257    |Xenopus laevis                                |E|Metazoa Chordata                   | >gi|148224299|ref|NP_001088198.1| progestin and adipoQ receptor family member IV [Xenopus laevis]
156369701 60..274    |Nematostella vectensis                        |E|Metazoa Cnidaria                   | >gi|156369701|ref|XP_001628113.1| predicted protein [Nematostella vectensis]
242023757 86..300    |Pediculus humanus corporis                    |E|Metazoa Arthropoda                 | >gi|242023757|ref|XP_002432297.1| conserved hypothetical protein [Pediculus humanus corporis]
58700649  52..266    |Acanthopagrus schlegelii                      |E|Metazoa Chordata                   | >gi|58700649|gb|AAW81033.1| membrane progestin receptor [Acanthopagrus schlegelii]
109127319 43..256    |Macaca mulatta                                |E|Metazoa Chordata                   | >gi|109127319|ref|XP_001088502.1| PREDICTED: progestin and adipoQ receptor family member 4 isoform 3 [Macaca mulatta]
126335331 43..256    |Monodelphis domestica                         |E|Metazoa Chordata                   | >gi|126335331|ref|XP_001371332.1| PREDICTED: progestin and adipoQ receptor family member 4-like [Monodelphis domestica]
12963841  43..256    |Mus musculus                                  |E|Metazoa Chordata                   | >gi|12963841|ref|NP_076313.1| progestin and adipoQ receptor family member 4 [Mus musculus]
281343468 38..251    |Ailuropoda melanoleuca                        |E|Metazoa Chordata                   | >gi|281343468|gb|EFB19052.1| hypothetical protein PANDA_021642 [Ailuropoda melanoleuca]
296219399 43..256    |Callithrix jacchus                            |E|Metazoa Chordata                   | >gi|296219399|ref|XP_002755864.1| PREDICTED: progestin and adipoQ receptor family member 4-like isoform 1 [Callithrix jacchus]
31542756  43..256    |Homo sapiens                                  |E|Metazoa Chordata                   | >gi|31542756|ref|NP_689554.2| progestin and adipoQ receptor family member 4 [Homo sapiens]
62821768  43..256    |Rattus norvegicus                             |E|Metazoa Chordata                   | >gi|62821768|ref|NP_001017377.1| progestin and adipoQ receptor family member 4 [Rattus norvegicus]
194379928 11..189    |Homo sapiens                                  |E|Metazoa Chordata                   | >gi|194379928|dbj|BAG58316.1| unnamed protein product [Homo sapiens]
16549125  50..217    |Homo sapiens                                  |E|Metazoa Chordata                   | >gi|16549125|dbj|BAB70758.1| unnamed protein product [Homo sapiens]
149642563 43..256    |Bos taurus                                    |E|Metazoa Chordata                   | >gi|149642563|ref|NP_001092367.1| progestin and adipoQ receptor family member 4 [Bos taurus]
57088535  43..256    |Canis lupus familiaris                        |E|Metazoa Chordata                   | >gi|57088535|ref|XP_547171.1| PREDICTED: similar to progestin and adipoQ receptor family member IV isoform 1 [Canis familiaris]
109127321 56..217    |Macaca mulatta                                |E|Metazoa Chordata                   | >gi|109127321|ref|XP_001088385.1| PREDICTED: progestin and adipoQ receptor family member 4 isoform 2 [Macaca mulatta]
296219401 56..217    |Callithrix jacchus                            |E|Metazoa Chordata                   | >gi|296219401|ref|XP_002755865.1| PREDICTED: progestin and adipoQ receptor family member 4-like isoform 2 [Callithrix jacchus]
297697900 56..217    |Pongo abelii                                  |E|Metazoa Chordata                   | >gi|297697900|ref|XP_002826076.1| PREDICTED: progestin and adipoQ receptor family member 4-like isoform 2 [Pongo abelii]
109127323 56..184    |Macaca mulatta                                |E|Metazoa Chordata                   | >gi|109127323|ref|XP_001088066.1| PREDICTED: progestin and adipoQ receptor family member 4 isoform 1 [Macaca mulatta]
21750701  56..184    |Homo sapiens                                  |E|Metazoa Chordata                   | >gi|21750701|dbj|BAC03821.1| unnamed protein product [Homo sapiens]
296219403 56..184    |Callithrix jacchus                            |E|Metazoa Chordata                   | >gi|296219403|ref|XP_002755866.1| PREDICTED: progestin and adipoQ receptor family member 4-like isoform 3 [Callithrix jacchus]
73959068  56..184    |Canis lupus familiaris                        |E|Metazoa Chordata                   | >gi|73959068|ref|XP_864556.1| PREDICTED: similar to progestin and adipoQ receptor family member IV isoform 2 [Canis familiaris]
260803390 39..252    |Branchiostoma floridae                        |E|Metazoa Chordata                   | >gi|260803390|ref|XP_002596573.1| hypothetical protein BRAFLDRAFT_234956 [Branchiostoma floridae]
291184693 63..276    |Trichophyton verrucosum HKI 0517              |E|Fungi Dikarya                      | >gi|291184693|gb|EFE40209.1| hemolysin-III family protein [Trichophyton verrucosum HKI 0517]
291175020 64..271    |Arthroderma benhamiae CBS 112371              |E|Fungi Dikarya                      | >gi|291175020|gb|EFE30829.1| hemolysin-III family protein [Arthroderma benhamiae CBS 112371]
281206261 259..470   |Polysphondylium pallidum PN500                |E|Amoebozoa Mycetozoa                | >gi|281206261|gb|EFA80450.1| Hly-III related family protein [Polysphondylium pallidum PN500]
133941682 36..245    |Caenorhabditis elegans                        |E|Metazoa Nematoda                   | >gi|133941682|ref|NP_504750.2| hypothetical protein K11C4.2 [Caenorhabditis elegans]
268558704 36..245    |Caenorhabditis briggsae                       |E|Metazoa Nematoda                   | >gi|268558704|ref|XP_002637343.1| Hypothetical protein CBG19039 [Caenorhabditis briggsae]
291237884 1..210     |Saccoglossus kowalevskii                      |E|Metazoa Hemichordata               | >gi|291237884|ref|XP_002738862.1| PREDICTED: progestin and adipoQ receptor family member VI-like, partial [Saccoglossus kowalevskii]
255087466 288..496   |Micromonas sp. RCC299                         |E|Viridiplantae Chlorophyta          | >gi|255087466|ref|XP_002505656.1| haemolysin-iii related expressed protein [Micromonas sp. RCC299]
119487620 3..210     |Neosartorya fischeri NRRL 181                 |E|Fungi Dikarya                      | >gi|119487620|ref|XP_001262542.1| Haemolysin-III related protein [Neosartorya fischeri NRRL 181]
291222803 42..249    |Saccoglossus kowalevskii                      |E|Metazoa Hemichordata               | >gi|291222803|ref|XP_002731404.1| PREDICTED: progestin and adipoQ receptor family member IV-like [Saccoglossus kowalevskii]
85089578  93..300    |Neurospora crassa OR74A                       |E|Fungi Dikarya                      | >gi|85089578|ref|XP_958013.1| hypothetical protein NCU04987 [Neurospora crassa OR74A]
170586014 51..257    |Brugia malayi                                 |E|Metazoa Nematoda                   | >gi|170586014|ref|XP_001897776.1| Haemolysin-III related family protein [Brugia malayi]
73950579  65..270    |Canis lupus familiaris                        |E|Metazoa Chordata                   | >gi|73950579|ref|XP_544490.2| PREDICTED: similar to progestin and adipoQ receptor family member VII [Canis familiaris]
149046854 64..268    |Rattus norvegicus                             |E|Metazoa Chordata                   | >gi|149046854|gb|EDL99628.1| rCG37954, isoform CRA_a [Rattus norvegicus]
148688400 64..212    |Mus musculus                                  |E|Metazoa Chordata                   | >gi|148688400|gb|EDL20347.1| progestin and adipoQ receptor family member III, isoform CRA_b [Mus musculus]
255711316 251..455   |Lachancea thermotolerans CBS 6340             |E|Fungi Dikarya                      | >gi|255711316|ref|XP_002551941.1| KLTH0B03498p [Lachancea thermotolerans]
168057079 162..365   |Physcomitrella patens subsp. patens           |E|Viridiplantae Streptophyta         | >gi|168057079|ref|XP_001780544.1| predicted protein [Physcomitrella patens subsp. patens]
170588681 215..418   |Brugia malayi                                 |E|Metazoa Nematoda                   | >gi|170588681|ref|XP_001899102.1| ADIPOR-like receptor C43G2.1 [Brugia malayi]
242826274 50..252    |Talaromyces stipitatus ATCC 10500             |E|Fungi Dikarya                      | >gi|242826274|ref|XP_002488607.1| adiponectin receptor, putative [Talaromyces stipitatus ATCC 10500]
224097246 190..390   |Populus trichocarpa                           |E|Viridiplantae Streptophyta         | >gi|224097246|ref|XP_002310891.1| predicted protein [Populus trichocarpa]
151941191 252..451   |Saccharomyces cerevisiae YJM789               |E|Fungi Dikarya                      | >gi|151941191|gb|EDN59569.1| membrane protein involved in zinc metabolism [Saccharomyces cerevisiae YJM789]
207343193 252..451   |Saccharomyces cerevisiae AWRI1631             |E|Fungi Dikarya                      | >gi|207343193|gb|EDZ70731.1| YLR023Cp-like protein [Saccharomyces cerevisiae AWRI1631]
259148012 252..451   |Saccharomyces cerevisiae EC1118               |E|Fungi Dikarya                      | >gi|259148012|emb|CAY81261.1| Izh3p [Saccharomyces cerevisiae EC1118]
6323051   252..451   |Saccharomyces cerevisiae S288c                |E|Fungi Dikarya                      | >gi|6323051|ref|NP_013123.1| Izh3p [Saccharomyces cerevisiae S288c]
170048664 15..214    |Culex quinquefasciatus                        |E|Metazoa Arthropoda                 | >gi|170048664|ref|XP_001870725.1| conserved hypothetical protein [Culex quinquefasciatus]
212275215 169..368   |Zea mays                                      |E|Viridiplantae Streptophyta         | >gi|212275215|ref|NP_001130559.1| hypothetical protein LOC100191658 [Zea mays]
238014870 182..381   |Zea mays                                      |E|Viridiplantae Streptophyta         | >gi|238014870|gb|ACR38470.1| unknown [Zea mays]
242083586 192..381   |Sorghum bicolor                               |E|Viridiplantae Streptophyta         | >gi|242083586|ref|XP_002442218.1| hypothetical protein SORBIDRAFT_08g016470 [Sorghum bicolor]
254578842 241..440   |Zygosaccharomyces rouxii CBS 732              |E|Fungi Dikarya                      | >gi|254578842|ref|XP_002495407.1| ZYRO0B10538p [Zygosaccharomyces rouxii]
118398850 268..466   |Tetrahymena thermophila                       |E|Alveolata Ciliophora               | >gi|118398850|ref|XP_001031752.1| hemolysin-III related protein [Tetrahymena thermophila]
149236599 292..490   |Lodderomyces elongisporus NRRL YB-4239        |E|Fungi Dikarya                      | >gi|149236599|ref|XP_001524177.1| conserved hypothetical protein [Lodderomyces elongisporus NRRL YB-4239]
198433746 7..205     |Ciona intestinalis                            |E|Metazoa Chordata                   | >gi|198433746|ref|XP_002125627.1| PREDICTED: similar to predicted protein [Ciona intestinalis]
225458870 206..404   |Vitis vinifera                                |E|Viridiplantae Streptophyta         | >gi|225458870|ref|XP_002285384.1| PREDICTED: hypothetical protein [Vitis vinifera]
71006436  396..594   |Ustilago maydis 521                           |E|Fungi Dikarya                      | >gi|71006436|ref|XP_757884.1| hypothetical protein UM01737.1 [Ustilago maydis 521]
116782209 148..345   |Picea sitchensis                              |E|Viridiplantae Streptophyta         | >gi|116782209|gb|ABK22411.1| unknown [Picea sitchensis]
169615685 51..248    |Phaeosphaeria nodorum SN15                    |E|Fungi Dikarya                      | >gi|169615685|ref|XP_001801258.1| hypothetical protein SNOG_11005 [Phaeosphaeria nodorum SN15]
262112030 285..482   |Phytophthora infestans T30-4                  |E|stramenopiles Oomycetes            | >gi|262112030|gb|EEY70082.1| adiponectin receptor protein, putative [Phytophthora infestans T30-4]
15233813  178..374   |Arabidopsis thaliana                          |E|Viridiplantae Streptophyta         | >gi|15233813|ref|NP_195545.1| heptahelical protein 5 [Arabidopsis thaliana]
15235657  178..374   |Arabidopsis thaliana                          |E|Viridiplantae Streptophyta         | >gi|15235657|ref|NP_195483.1| heptahelical protein 4 [Arabidopsis thaliana]
51970506  178..374   |Arabidopsis thaliana                          |E|Viridiplantae Streptophyta         | >gi|51970506|dbj|BAD43945.1| putative protein [Arabidopsis thaliana]
297802184 184..375   |Arabidopsis lyrata subsp. lyrata              |E|Viridiplantae Streptophyta         | >gi|297802184|ref|XP_002868976.1| hypothetical protein ARALYDRAFT_490848 [Arabidopsis lyrata subsp. lyrata]
164659416 61..257    |Malassezia globosa CBS 7966                   |E|Fungi Dikarya                      | >gi|164659416|ref|XP_001730832.1| hypothetical protein MGL_1831 [Malassezia globosa CBS 7966]
255568631 192..388   |Ricinus communis                              |E|Viridiplantae Streptophyta         | >gi|255568631|ref|XP_002525289.1| conserved hypothetical protein [Ricinus communis]
255635835 167..363   |Glycine max                                   |E|Viridiplantae Streptophyta         | >gi|255635835|gb|ACU18265.1| unknown [Glycine max]
255644752 173..369   |Glycine max                                   |E|Viridiplantae Streptophyta         | >gi|255644752|gb|ACU22878.1| unknown [Glycine max]
255634469 1..176     |Glycine max                                   |E|Viridiplantae Streptophyta         | >gi|255634469|gb|ACU17599.1| unknown [Glycine max]
118374093 250..445   |Tetrahymena thermophila                       |E|Alveolata Ciliophora               | >gi|118374093|ref|XP_001020238.1| hemolysin-III related protein [Tetrahymena thermophila]
122937709 193..388   |Lilium longiflorum                            |E|Viridiplantae Streptophyta         | >gi|122937709|gb|ABM68566.1| hemolysin III-related family protein [Lilium longiflorum]
146414616 248..443   |Meyerozyma guilliermondii ATCC 6260           |E|Fungi Dikarya                      | >gi|146414616|ref|XP_001483278.1| hypothetical protein PGUG_04007 [Meyerozyma guilliermondii ATCC 6260]
190347604 248..443   |Meyerozyma guilliermondii ATCC 6260           |E|Fungi Dikarya                      | >gi|190347604|gb|EDK39909.2| hypothetical protein PGUG_04007 [Meyerozyma guilliermondii ATCC 6260]
150864799 255..450   |Scheffersomyces stipitis CBS 6054             |E|Fungi Dikarya                      | >gi|150864799|ref|XP_001383775.2| hypothetical protein PICST_88627 [Scheffersomyces stipitis CBS 6054]
224127168 174..369   |Populus trichocarpa                           |E|Viridiplantae Streptophyta         | >gi|224127168|ref|XP_002320004.1| predicted protein [Populus trichocarpa]
225467604 287..482   |Vitis vinifera                                |E|Viridiplantae Streptophyta         | >gi|225467604|ref|XP_002264538.1| PREDICTED: hypothetical protein [Vitis vinifera]
225438241 19..213    |Vitis vinifera                                |E|Viridiplantae Streptophyta         | >gi|225438241|ref|XP_002264612.1| PREDICTED: hypothetical protein [Vitis vinifera]
296084692 185..379   |Vitis vinifera                                |E|Viridiplantae Streptophyta         | >gi|296084692|emb|CBI25834.3| unnamed protein product [Vitis vinifera]
238882995 287..482   |Candida albicans WO-1                         |E|Fungi Dikarya                      | >gi|238882995|gb|EEQ46633.1| conserved hypothetical protein [Candida albicans WO-1]
68475767  287..482   |Candida albicans SC5314                       |E|Fungi Dikarya                      | >gi|68475767|ref|XP_718090.1| potential haemolysin-related integral membrane protein [Candida albicans SC5314]
68475902  287..482   |Candida albicans SC5314                       |E|Fungi Dikarya                      | >gi|68475902|ref|XP_718024.1| potential haemolysin-related integral membrane protein [Candida albicans SC5314]
241956846 306..501   |Candida dubliniensis CD36                     |E|Fungi Dikarya                      | >gi|241956846|ref|XP_002421143.1| ADIPOR-like receptor IZH3 orthologue, putative; implicated in zinc homeostasis protein, putative [Candida dubliniensis CD36]
255538030 210..405   |Ricinus communis                              |E|Viridiplantae Streptophyta         | >gi|255538030|ref|XP_002510080.1| conserved hypothetical protein [Ricinus communis]
255574958 59..254    |Ricinus communis                              |E|Viridiplantae Streptophyta         | >gi|255574958|ref|XP_002528385.1| conserved hypothetical protein [Ricinus communis]
255732413 286..481   |Candida tropicalis MYA-3404                   |E|Fungi Dikarya                      | >gi|255732413|ref|XP_002551130.1| conserved hypothetical protein [Candida tropicalis MYA-3404]
294657072 247..442   |Debaryomyces hansenii CBS767                  |E|Fungi Dikarya                      | >gi|294657072|ref|XP_459388.2| DEHA2E01408p [Debaryomyces hansenii CBS767]
115451741 198..392   |Oryza sativa Japonica Group                   |E|Viridiplantae Streptophyta         | >gi|115451741|ref|NP_001049471.1| Os03g0232900 [Oryza sativa Japonica Group]
218192393 198..392   |Oryza sativa Indica Group                     |E|Viridiplantae Streptophyta         | >gi|218192393|gb|EEC74820.1| hypothetical protein OsI_10642 [Oryza sativa Indica Group]
222624521 156..350   |Oryza sativa Japonica Group                   |E|Viridiplantae Streptophyta         | >gi|222624521|gb|EEE58653.1| hypothetical protein OsJ_10038 [Oryza sativa Japonica Group]
242041679 197..391   |Sorghum bicolor                               |E|Viridiplantae Streptophyta         | >gi|242041679|ref|XP_002468234.1| hypothetical protein SORBIDRAFT_01g042200 [Sorghum bicolor]
20330770  198..352   |Oryza sativa Japonica Group                   |E|Viridiplantae Streptophyta         | >gi|20330770|gb|AAM19133.1|AC103891_13 Hypothetical protein [Oryza sativa Japonica Group]
56753559  197..391   |Schistosoma japonicum                         |E|Metazoa Platyhelminthes            | >gi|56753559|gb|AAW24982.1| SJCHGC03479 protein [Schistosoma japonicum]
296812575 73..266    |Arthroderma otae CBS 113480                   |E|Fungi Dikarya                      | >gi|296812575|ref|XP_002846625.1| hemolysin-III family protein [Arthroderma otae CBS 113480]
255582305 157..348   |Ricinus communis                              |E|Viridiplantae Streptophyta         | >gi|255582305|ref|XP_002531943.1| conserved hypothetical protein [Ricinus communis]
46116418  72..263    |Gibberella zeae PH-1                          |E|Fungi Dikarya                      | >gi|46116418|ref|XP_384227.1| hypothetical protein FG04051.1 [Gibberella zeae PH-1]
145519836 237..427   |Paramecium tetraurelia strain d4-2            |E|Alveolata Ciliophora               | >gi|145519836|ref|XP_001445779.1| hypothetical protein [Paramecium tetraurelia strain d4-2]
147816024 341..531   |Vitis vinifera                                |E|Viridiplantae Streptophyta         | >gi|147816024|emb|CAN61543.1| hypothetical protein VITISV_008489 [Vitis vinifera]
225445767 160..350   |Vitis vinifera                                |E|Viridiplantae Streptophyta         | >gi|225445767|ref|XP_002274323.1| PREDICTED: hypothetical protein [Vitis vinifera]
148907642 232..422   |Picea sitchensis                              |E|Viridiplantae Streptophyta         | >gi|148907642|gb|ABR16950.1| unknown [Picea sitchensis]
242041865 182..372   |Sorghum bicolor                               |E|Viridiplantae Streptophyta         | >gi|242041865|ref|XP_002468327.1| hypothetical protein SORBIDRAFT_01g043860 [Sorghum bicolor]
115488760 218..407   |Oryza sativa Japonica Group                   |E|Viridiplantae Streptophyta         | >gi|115488760|ref|NP_001066867.1| Os12g0511200 [Oryza sativa Japonica Group]
125579495 194..383   |Oryza sativa Japonica Group                   |E|Viridiplantae Streptophyta         | >gi|125579495|gb|EAZ20641.1| hypothetical protein OsJ_36257 [Oryza sativa Japonica Group]
145542805 225..414   |Paramecium tetraurelia strain d4-2            |E|Alveolata Ciliophora               | >gi|145542805|ref|XP_001457089.1| hypothetical protein [Paramecium tetraurelia strain d4-2]
159466110 195..384   |Chlamydomonas reinhardtii                     |E|Viridiplantae Chlorophyta          | >gi|159466110|ref|XP_001691252.1| hypothetical protein CHLREDRAFT_128250 [Chlamydomonas reinhardtii]
168010135 227..415   |Physcomitrella patens subsp. patens           |E|Viridiplantae Streptophyta         | >gi|168010135|ref|XP_001757760.1| predicted protein [Physcomitrella patens subsp. patens]
168053080 228..416   |Physcomitrella patens subsp. patens           |E|Viridiplantae Streptophyta         | >gi|168053080|ref|XP_001778966.1| predicted protein [Physcomitrella patens subsp. patens]
169848062 563..751   |Coprinopsis cinerea okayama7#130              |E|Fungi Dikarya                      | >gi|169848062|ref|XP_001830739.1| hypothetical protein CC1G_03276 [Coprinopsis cinerea okayama7#130]
189207196 76..264    |Pyrenophora tritici-repentis Pt-1C-BFP        |E|Fungi Dikarya                      | >gi|189207196|ref|XP_001939932.1| adiponectin receptor protein 1 [Pyrenophora tritici-repentis Pt-1C-BFP]
224060100 1..189     |Taeniopygia guttata                           |E|Metazoa Chordata                   | >gi|224060100|ref|XP_002195610.1| PREDICTED: similar to progestin and adipoQ receptor family member IX, partial [Taeniopygia guttata]
290999333 1..189     |Naegleria gruberi strain NEG-M                |E|Heterolobosea Schizopyrenida       | >gi|290999333|ref|XP_002682234.1| predicted protein [Naegleria gruberi]
145530491 242..429   |Paramecium tetraurelia strain d4-2            |E|Alveolata Ciliophora               | >gi|145530491|ref|XP_001451023.1| hypothetical protein [Paramecium tetraurelia strain d4-2]
145538009 227..414   |Paramecium tetraurelia strain d4-2            |E|Alveolata Ciliophora               | >gi|145538009|ref|XP_001454710.1| hypothetical protein [Paramecium tetraurelia strain d4-2]
260807617 100..286   |Branchiostoma floridae                        |E|Metazoa Chordata                   | >gi|260807617|ref|XP_002598605.1| hypothetical protein BRAFLDRAFT_66999 [Branchiostoma floridae]
114560242 47..231    |Pan troglodytes                               |E|Metazoa Chordata                   | >gi|114560242|ref|XP_001164575.1| PREDICTED: similar to Progestin and adipoQ receptor family member VI isoform 3 [Pan troglodytes]
119573361 1..170     |Homo sapiens                                  |E|Metazoa Chordata                   | >gi|119573361|gb|EAW52976.1| progestin and adipoQ receptor family member VI, isoform CRA_b [Homo sapiens]
12698174  1..170     |Macaca fascicularis                           |E|Metazoa Chordata                   | >gi|12698174|dbj|BAB21914.1| hypothetical protein [Macaca fascicularis]
33187693  1..170     |Homo sapiens                                  |E|Metazoa Chordata                   | >gi|33187693|gb|AAP97701.1|AF455045_1 hypothetical protein [Homo sapiens]
90077208  1..170     |Macaca fascicularis                           |E|Metazoa Chordata                   | >gi|90077208|dbj|BAE88284.1| unnamed protein product [Macaca fascicularis]
114560230 1..148     |Pan troglodytes                               |E|Metazoa Chordata                   | >gi|114560230|ref|XP_001164497.1| PREDICTED: progestin and adipoQ receptor family member VI isoform 1 [Pan troglodytes]
24432011  1..148     |Homo sapiens                                  |E|Metazoa Chordata                   | >gi|24432011|ref|NP_079173.2| progestin and adipoQ receptor family member 6 isoform 1 [Homo sapiens]
10439153  1..138     |Homo sapiens                                  |E|Metazoa Chordata                   | >gi|10439153|dbj|BAB15446.1| unnamed protein product [Homo sapiens]
116059892 261..441   |Ostreococcus tauri                            |E|Viridiplantae Chlorophyta          | >gi|116059892|emb|CAL55599.1| Predicted membrane proteins, contain hemolysin III domain (ISS) [Ostreococcus tauri]
189205110 63..243    |Pyrenophora tritici-repentis Pt-1C-BFP        |E|Fungi Dikarya                      | >gi|189205110|ref|XP_001938890.1| adiponectin receptor protein 1 [Pyrenophora tritici-repentis Pt-1C-BFP]
125598016 171..345   |Oryza sativa Japonica Group                   |E|Viridiplantae Streptophyta         | >gi|125598016|gb|EAZ37796.1| hypothetical protein OsJ_22132 [Oryza sativa Japonica Group]
297606256 177..351   |Oryza sativa Japonica Group                   |E|Viridiplantae Streptophyta         | >gi|297606256|ref|NP_001058180.2| Os06g0643700 [Oryza sativa Japonica Group]
51535474  171..345   |Oryza sativa Japonica Group                   |E|Viridiplantae Streptophyta         | >gi|51535474|dbj|BAD37371.1| putative adiponectin receptor 1 [Oryza sativa Japonica Group]
145503590 1..173     |Paramecium tetraurelia strain d4-2            |E|Alveolata Ciliophora               | >gi|145503590|ref|XP_001437770.1| hypothetical protein [Paramecium tetraurelia strain d4-2]
115709073 1..172     |Strongylocentrotus purpuratus                 |E|Metazoa Echinodermata              | >gi|115709073|ref|XP_001201842.1| PREDICTED: hypothetical protein, partial [Strongylocentrotus purpuratus]
124784357 3..174     |Taenia asiatica                               |E|Metazoa Platyhelminthes            | >gi|124784357|gb|ABN14974.1| progestin and adipoQ receptor family member V [Taenia asiatica]
241834479 32..201    |Ixodes scapularis                             |E|Metazoa Arthropoda                 | >gi|241834479|ref|XP_002414998.1| progestin and adipoQ receptor, putative [Ixodes scapularis]
157133319 164..330   |Aedes aegypti                                 |E|Metazoa Arthropoda                 | >gi|157133319|ref|XP_001662832.1| hypothetical protein AaeL_AAEL012718 [Aedes aegypti]
157126874 53..209    |Aedes aegypti                                 |E|Metazoa Arthropoda                 | >gi|157126874|ref|XP_001660987.1| hypothetical protein AaeL_AAEL010672 [Aedes aegypti]
158296394 99..265    |Anopheles gambiae str. PEST                   |E|Metazoa Arthropoda                 | >gi|158296394|ref|XP_316813.4| AGAP000844-PA [Anopheles gambiae str. PEST]
195391420 252..418   |Drosophila virilis                            |E|Metazoa Arthropoda                 | >gi|195391420|ref|XP_002054358.1| GJ22843 [Drosophila virilis]
195503462 209..375   |Drosophila yakuba                             |E|Metazoa Arthropoda                 | >gi|195503462|ref|XP_002098662.1| GE10488 [Drosophila yakuba]
242825744 51..215    |Talaromyces stipitatus ATCC 10500             |E|Fungi Dikarya                      | >gi|242825744|ref|XP_002488501.1| adiponectin receptor, putative [Talaromyces stipitatus ATCC 10500]
148230782 64..221    |Xenopus laevis                                |E|Metazoa Chordata                   | >gi|148230782|ref|NP_001090458.1| hypothetical protein LOC779371 [Xenopus laevis]
169775389 71..227    |Aspergillus oryzae RIB40                      |E|Fungi Dikarya                      | >gi|169775389|ref|XP_001822162.1| hypothetical protein [Aspergillus oryzae RIB40]
238495965 21..161    |Aspergillus flavus NRRL3357                   |E|Fungi Dikarya                      | >gi|238495965|ref|XP_002379218.1| hemolysin-III family protein [Aspergillus flavus NRRL3357]
115738298 191..342   |Strongylocentrotus purpuratus                 |E|Metazoa Echinodermata              | >gi|115738298|ref|XP_790490.2| PREDICTED: similar to adiponectin receptor 2 [Strongylocentrotus purpuratus]
296808143 63..214    |Arthroderma otae CBS 113480                   |E|Fungi Dikarya                      | >gi|296808143|ref|XP_002844410.1| hemolysin-III family protein [Arthroderma otae CBS 113480]
145252504 164..314   |Aspergillus niger CBS 513.88                  |E|Fungi Dikarya                      | >gi|145252504|ref|XP_001397765.1| hypothetical protein An16g04540 [Aspergillus niger]
47200149  10..157    |Tetraodon nigroviridis                        |E|Metazoa Chordata                   | >gi|47200149|emb|CAF88140.1| unnamed protein product [Tetraodon nigroviridis]
297743702 160..300   |Vitis vinifera                                |E|Viridiplantae Streptophyta         | >gi|297743702|emb|CBI36585.3| unnamed protein product [Vitis vinifera]
148683352 26..165    |Mus musculus                                  |E|Metazoa Chordata                   | >gi|148683352|gb|EDL15299.1| mCG8849, isoform CRA_b [Mus musculus]
148683344 2..105     |Mus musculus                                  |E|Metazoa Chordata                   | >gi|148683344|gb|EDL15291.1| mCG133422 [Mus musculus]
242002196 95..233    |Ixodes scapularis                             |E|Metazoa Arthropoda                 | >gi|242002196|ref|XP_002435741.1| adiponectin receptor, putative [Ixodes scapularis]
156536981 1..131     |Nasonia vitripennis                           |E|Metazoa Arthropoda                 | >gi|156536981|ref|XP_001608278.1| PREDICTED: similar to HL06052p [Nasonia vitripennis]
226531932 80..208    |Zea mays                                      |E|Viridiplantae Streptophyta         | >gi|226531932|ref|NP_001147245.1| hemolysin-III related family protein [Zea mays]
260951131 251..377   |Clavispora lusitaniae ATCC 42720              |E|Fungi Dikarya                      | >gi|260951131|ref|XP_002619862.1| hypothetical protein CLUG_01021 [Clavispora lusitaniae ATCC 42720]
297802056 922..1048  |Arabidopsis lyrata subsp. lyrata              |E|Viridiplantae Streptophyta         | >gi|297802056|ref|XP_002868912.1| hypothetical protein ARALYDRAFT_352947 [Arabidopsis lyrata subsp. lyrata]
119573363 40..165    |Homo sapiens                                  |E|Metazoa Chordata                   | >gi|119573363|gb|EAW52978.1| progestin and adipoQ receptor family member VI, isoform CRA_c [Homo sapiens]
55957521  191..295   |Homo sapiens                                  |E|Metazoa Chordata                   | >gi|55957521|emb|CAI15545.1| progestin and adipoQ receptor family member VI [Homo sapiens]
218186937 205..327   |Oryza sativa Indica Group                     |E|Viridiplantae Streptophyta         | >gi|218186937|gb|EEC69364.1| hypothetical protein OsI_38488 [Oryza sativa Indica Group]
291186037 75..192    |Trichophyton verrucosum HKI 0517              |E|Fungi Dikarya                      | >gi|291186037|gb|EFE41490.1| hemolysin-III family protein [Trichophyton verrucosum HKI 0517]
291180133 75..191    |Arthroderma benhamiae CBS 112371              |E|Fungi Dikarya                      | >gi|291180133|gb|EFE35918.1| hemolysin-III family protein [Arthroderma benhamiae CBS 112371]
148683342 47..160    |Mus musculus                                  |E|Metazoa Chordata                   | >gi|148683342|gb|EDL15289.1| mCG133432 [Mus musculus]
149251550 9..120     |Mus musculus                                  |E|Metazoa Chordata                   | >gi|149251550|ref|XP_914776.2| PREDICTED: similar to progestin and adipoQ receptor family member VI [Mus musculus]
82890264  28..139    |Mus musculus                                  |E|Metazoa Chordata                   | >gi|82890264|ref|XP_897804.1| PREDICTED: similar to progestin and adipoQ receptor family member VI [Mus musculus]
156033033 1..113     |Sclerotinia sclerotiorum 1980 UF-70           |E|Fungi Dikarya                      | >gi|156033033|ref|XP_001585353.1| hypothetical protein SS1G_13592 [Sclerotinia sclerotiorum 1980]
260788990 22..131    |Branchiostoma floridae                        |E|Metazoa Chordata                   | >gi|260788990|ref|XP_002589531.1| hypothetical protein BRAFLDRAFT_97030 [Branchiostoma floridae]
295670872 64..172    |Paracoccidioides brasiliensis Pb01            |E|Fungi Dikarya                      | >gi|295670872|ref|XP_002795983.1| conserved hypothetical protein [Paracoccidioides brasiliensis Pb01]
71419420  34..329    |Trypanosoma cruzi strain CL Brener            |E|Euglenozoa Kinetoplastida          | >gi|71419420|ref|XP_811164.1| hypothetical protein [Trypanosoma cruzi strain CL Brener]
71665594  34..329    |Trypanosoma cruzi strain CL Brener            |E|Euglenozoa Kinetoplastida          | >gi|71665594|ref|XP_819765.1| hypothetical protein [Trypanosoma cruzi strain CL Brener]
261329742 34..313    |Trypanosoma brucei gambiense DAL972           |E|Euglenozoa Kinetoplastida          | >gi|261329742|emb|CBH12724.1| hypothetical protein, conserved [Trypanosoma brucei gambiense DAL972]
72391730  34..313    |Trypanosoma brucei TREU927                    |E|Euglenozoa Kinetoplastida          | >gi|72391730|ref|XP_846159.1| hypothetical protein [Trypanosoma brucei TREU927]
154331137 222..366   |Leishmania braziliensis MHOM/BR/75/M2904      |E|Euglenozoa Kinetoplastida          | >gi|154331137|ref|XP_001562008.1| hypothetical protein [Leishmania braziliensis MHOM/BR/75/M2904]
146071614 237..365   |Leishmania infantum JPCM5                     |E|Euglenozoa Kinetoplastida          | >gi|146071614|ref|XP_001463158.1| hypothetical protein [Leishmania infantum]
157864271 235..361   |Leishmania major strain Friedlin              |E|Euglenozoa Kinetoplastida          | >gi|157864271|ref|XP_001680846.1| hypothetical protein [Leishmania major strain Friedlin]
123428294 59..264    |Trichomonas vaginalis G3                      |E|Parabasalia Trichomonadida         | >gi|123428294|ref|XP_001307450.1| Haemolysin-III related family protein [Trichomonas vaginalis G3]
123494441 59..264    |Trichomonas vaginalis G3                      |E|Parabasalia Trichomonadida         | >gi|123494441|ref|XP_001326509.1| hypothetical protein [Trichomonas vaginalis G3]
159466320 84..284    |Chlamydomonas reinhardtii                     |E|Viridiplantae Chlorophyta          | >gi|159466320|ref|XP_001691357.1| predicted protein [Chlamydomonas reinhardtii]
159481311 115..278   |Chlamydomonas reinhardtii                     |E|Viridiplantae Chlorophyta          | >gi|159481311|ref|XP_001698725.1| hypothetical protein CHLREDRAFT_151803 [Chlamydomonas reinhardtii]
198413063 42..274    |Ciona intestinalis                            |E|Metazoa Chordata                   | >gi|198413063|ref|XP_002124832.1| PREDICTED: similar to predicted protein, partial [Ciona intestinalis]

```
PAQR group 2:
198435153 50..301    |Ciona intestinalis                            |E|Metazoa Chordata                   | >gi|198435153|ref|XP_002127427.1| PREDICTED: similar to monocyte to macrophage differentiation-associated [Ciona intestinalis]
66820859  26..269    |Dictyostelium discoideum AX4                  |E|Amoebozoa Mycetozoa                | >gi|66820859|ref|XP_643985.1| hypothetical protein DDB_G0274283 [Dictyostelium discoideum AX4]
195353433 129..354   |Drosophila sechellia                          |E|Metazoa Arthropoda                 | >gi|195353433|ref|XP_002043209.1| GM17479 [Drosophila sechellia]
194896669 210..427   |Drosophila erecta                             |E|Metazoa Arthropoda                 | >gi|194896669|ref|XP_001978519.1| GG17636 [Drosophila erecta]
195480531 208..425   |Drosophila yakuba                             |E|Metazoa Arthropoda                 | >gi|195480531|ref|XP_002101293.1| GE17542 [Drosophila yakuba]
195565546 85..302    |Drosophila simulans                           |E|Metazoa Arthropoda                 | >gi|195565546|ref|XP_002106360.1| GD16164 [Drosophila simulans]
24640324  85..302    |Drosophila melanogaster                       |E|Metazoa Arthropoda                 | >gi|24640324|ref|NP_572382.1| CG4615 [Drosophila melanogaster]
114611999 43..265    |Pan troglodytes                               |E|Metazoa Chordata                   | >gi|114611999|ref|XP_527649.2| PREDICTED: monocyte-to-macrophage differentiation factor 2 [Pan troglodytes]
154759277 34..256    |Homo sapiens                                  |E|Metazoa Chordata                   | >gi|154759277|ref|NP_001094070.1| monocyte to macrophage differentiation factor 2 isoform 1 [Homo sapiens]
71052022  34..256    |Homo sapiens                                  |E|Metazoa Chordata                   | >gi|71052022|gb|AAH37881.2| MMD2 protein [Homo sapiens]
194246712 12..232    |Candidatus Phytoplasma mali                   |B|Tenericutes Mollicutes             | >gi|194246712|ref|YP_002004351.1| Channel protein, hemolysin III family [Candidatus Phytoplasma mali]
194768437 77..294    |Drosophila ananassae                          |E|Metazoa Arthropoda                 | >gi|194768437|ref|XP_001966318.1| GF22105 [Drosophila ananassae]
195040229 59..276    |Drosophila grimshawi                          |E|Metazoa Arthropoda                 | >gi|195040229|ref|XP_001991029.1| GH12297 [Drosophila grimshawi]
195131535 59..276    |Drosophila mojavensis                         |E|Metazoa Arthropoda                 | >gi|195131535|ref|XP_002010206.1| GI15804 [Drosophila mojavensis]
195394075 79..296    |Drosophila virilis                            |E|Metazoa Arthropoda                 | >gi|195394075|ref|XP_002055671.1| GJ18658 [Drosophila virilis]
195162395 73..290    |Drosophila persimilis                         |E|Metazoa Arthropoda                 | >gi|195162395|ref|XP_002022041.1| GL14200 [Drosophila persimilis]
195432216 61..278    |Drosophila willistoni                         |E|Metazoa Arthropoda                 | >gi|195432216|ref|XP_002064122.1| GK19878 [Drosophila willistoni]
197294257 14..227    |Candidatus Phytoplasma australiense           |B|Tenericutes Mollicutes             | >gi|197294257|ref|YP_001798798.1| Hemolysin III related protein [Candidatus Phytoplasma australiense]
290984063 73..283    |Naegleria gruberi strain NEG-M                |E|Heterolobosea Schizopyrenida       | >gi|290984063|ref|XP_002674747.1| predicted protein [Naegleria gruberi]
192337537 21..230    |Wheat blue dwarf phytoplasma                  |B|Tenericutes Mollicutes             | >gi|192337537|gb|ACF04190.1| hemolysin III-like protein [Wheat blue dwarf phytoplasma]
192337542 21..230    |Paulownia witches'-broom phytoplasma          |B|Tenericutes Mollicutes             | >gi|192337542|gb|ACF04192.1| hemolysin III-like protein [Paulownia witches'-broom phytoplasma]
229918268 3..212     |Exiguobacterium sp. AT1b                      |B|Firmicutes Bacillales              | >gi|229918268|ref|YP_002886914.1| channel protein, hemolysin III family [Exiguobacterium sp. AT1b]
260578663 6..215     |Corynebacterium jeikeium ATCC 43734           |B|Actinobacteria Actinobacteridae    | >gi|260578663|ref|ZP_05846571.1| membrane protein [Corynebacterium jeikeium ATCC 43734]
68535957  6..215     |Corynebacterium jeikeium K411                 |B|Actinobacteria Actinobacteridae    | >gi|68535957|ref|YP_250662.1| hypothetical protein jk0880 [Corynebacterium jeikeium K411]
290987311 87..296    |Naegleria gruberi strain NEG-M                |E|Heterolobosea Schizopyrenida       | >gi|290987311|ref|XP_002676366.1| predicted protein [Naegleria gruberi]
39938644  14..223    |Onion yellows phytoplasma OY-M                |B|Tenericutes Mollicutes             | >gi|39938644|ref|NP_950410.1| hemolysin III-like protein [Onion yellows phytoplasma OY-M]
1923237   1..146     |Chlorante-Aster yellows phytoplasma           |B|Tenericutes Mollicutes             | >gi|1923237|gb|AAB51344.1| unknown [Chlorante-Aster yellows phytoplasma]
85057841  62..271    |Aster yellows witches'-broom phytoplasma AYWB |B|Tenericutes Mollicutes             | >gi|85057841|ref|YP_456757.1| hemolysin III [Aster yellows witches'-broom phytoplasma AYWB]
15894169  3..211     |Clostridium acetobutylicum ATCC 824           |B|Firmicutes Clostridia              | >gi|15894169|ref|NP_347518.1| hemolysin III-like protein [Clostridium acetobutylicum ATCC 824]
162447357 13..221    |Acholeplasma laidlawii PG-8A                  |B|Tenericutes Mollicutes             | >gi|162447357|ref|YP_001620489.1| integral membrane protein [Acholeplasma laidlawii PG-8A]
172040766 50..258    |Corynebacterium urealyticum DSM 7109          |B|Actinobacteria Actinobacteridae    | >gi|172040766|ref|YP_001800480.1| transporter of the Hly III family [Corynebacterium urealyticum DSM 7109]
239617011 18..226    |Kosmotoga olearia TBF 19.5.1                  |B|Thermotogae Thermotogales          | >gi|239617011|ref|YP_002940333.1| channel protein, hemolysin III family [Kosmotoga olearia TBF 19.5.1]
294874392 263..471   |Perkinsus marinus ATCC 50983                  |E|Alveolata Perkinsea                | >gi|294874392|ref|XP_002766933.1| hemolysin iii, putative [Perkinsus marinus ATCC 50983]
297582785 4..212     |Bacillus selenitireducens MLS10               |B|Firmicutes Bacillales              | >gi|297582785|ref|YP_003698565.1| hemolysin III family channel protein [Bacillus selenitireducens MLS10]
116747679 4..211     |Syntrophobacter fumaroxidans MPOB             |B|Proteobacteria Deltaproteobacteria | >gi|116747679|ref|YP_844366.1| hemolysin III family channel protein [Syntrophobacter fumaroxidans MPOB]
119715295 40..247    |Nocardioides sp. JS614                        |B|Actinobacteria Actinobacteridae    | >gi|119715295|ref|YP_922260.1| Hly-III family protein [Nocardioides sp. JS614]
119952919 17..224    |Borrelia turicatae 91E135                     |B|Spirochaetes Spirochaetales        | >gi|119952919|ref|YP_945128.1| hypothetical protein BT0117 [Borrelia turicatae 91E135]
126699150 7..214     |Clostridium difficile 630                     |B|Firmicutes Clostridia              | >gi|126699150|ref|YP_001088047.1| putative hemolysin-like membrane protein [Clostridium difficile 630]
255655567 7..214     |Clostridium difficile QCD-23m63               |B|Firmicutes Clostridia              | >gi|255655567|ref|ZP_05400976.1| putative hemolysin-like membrane protein [Clostridium difficile QCD-23m63]
145955324 8..118     |Clostridium difficile QCD-32g58               |B|Firmicutes Clostridia              | >gi|145955324|ref|ZP_01804328.1| hypothetical protein CdifQ_04001745 [Clostridium difficile QCD-32g58]
153813686 7..214     |Ruminococcus obeum ATCC 29174                 |B|Firmicutes Clostridia              | >gi|153813686|ref|ZP_01966354.1| hypothetical protein RUMOBE_04110 [Ruminococcus obeum ATCC 29174]
154492249 8..215     |Parabacteroides merdae ATCC 43184             |B|Bacteroidetes Bacteroidia          | >gi|154492249|ref|ZP_02031875.1| hypothetical protein PARMER_01883 [Parabacteroides merdae ATCC 43184]
156537818 47..254    |Nasonia vitripennis                           |E|Metazoa Arthropoda                 | >gi|156537818|ref|XP_001608073.1| PREDICTED: similar to CG4615-PA [Nasonia vitripennis]
15806880  9..216     |Deinococcus radiodurans R1                    |B|Deinococcus-Thermus Deinococci     | >gi|15806880|ref|NP_295603.1| hemolysin [Deinococcus radiodurans R1]
167379965 26..233    |Entamoeba dispar SAW760                       |E|Amoebozoa Archamoebae              | >gi|167379965|ref|XP_001735342.1| hemolysin-3 [Entamoeba dispar SAW760]
183234844 26..233    |Entamoeba histolytica HM-1:IMSS               |E|Amoebozoa Archamoebae              | >gi|183234844|ref|XP_654314.2| hemolysin-3 [Entamoeba histolytica HM-1:IMSS]
167757201 15..222    |Clostridium ramosum DSM 1402                  |B|Firmicutes Erysipelotrichi         | >gi|167757201|ref|ZP_02429328.1| hypothetical protein CLORAM_02751 [Clostridium ramosum DSM 1402]
167769394 7..214     |Anaerotruncus colihominis DSM 17241           |B|Firmicutes Clostridia              | >gi|167769394|ref|ZP_02441447.1| hypothetical protein ANACOL_00724 [Anaerotruncus colihominis DSM 17241]
187917995 17..224    |Borrelia hermsii DAH                          |B|Spirochaetes Spirochaetales        | >gi|187917995|ref|YP_001883558.1| hypothetical protein BH0117 [Borrelia hermsii DAH]
203284042 17..224    |Borrelia duttonii Ly                          |B|Spirochaetes Spirochaetales        | >gi|203284042|ref|YP_002221782.1| hemolysin III [Borrelia duttonii Ly]
218261955 8..215     |Parabacteroides johnsonii DSM 18315           |B|Bacteroidetes Bacteroidia          | >gi|218261955|ref|ZP_03476616.1| hypothetical protein PRABACTJOHN_02287 [Parabacteroides johnsonii DSM 18315]
227500228 12..219    |Anaerococcus tetradius ATCC 35098             |B|Firmicutes Clostridia              | >gi|227500228|ref|ZP_03930297.1| Hly III family protein [Anaerococcus tetradius ATCC 35098]
239624698 13..220    |Clostridiales bacterium 1_7_47FAA             |B|Firmicutes Clostridia              | >gi|239624698|ref|ZP_04667729.1| conserved hypothetical protein [Clostridiales bacterium 1_7_47_FAA]
253566074 7..214     |Bacteroides sp. 3_2_5                         |B|Bacteroidetes Bacteroidia          | >gi|253566074|ref|ZP_04843528.1| hemolysin III [Bacteroides sp. 3_2_5]
265766823 7..214     |Bacteroides sp. 2_1_16                        |B|Bacteroidetes Bacteroidia          | >gi|265766823|ref|ZP_06094652.1| hemolysin III [Bacteroides sp. 2_1_16]
53714976  7..214     |Bacteroides fragilis YCH46                    |B|Bacteroidetes Bacteroidia          | >gi|53714976|ref|YP_100968.1| hemolysin III [Bacteroides fragilis YCH46]
255011118 7..214     |Bacteroides fragilis 3_1_12                   |B|Bacteroidetes Bacteroidia          | >gi|255011118|ref|ZP_05283244.1| putative hemolysin [Bacteroides fragilis 3_1_12]
255524071 7..214     |Clostridium carboxidivorans P7                |B|Firmicutes Clostridia              | >gi|255524071|ref|ZP_05391032.1| channel protein, hemolysin III family [Clostridium carboxidivorans P7]
260642213 21..228    |Bacteroides finegoldii DSM 17565              |B|Bacteroidetes Bacteroidia          | >gi|260642213|ref|ZP_05414895.2| hemolysin III [Bacteroides finegoldii DSM 17565]
280958171 4..211     |Desulfovibrio aespoeensis Aspo-2              |B|Proteobacteria Deltaproteobacteria | >gi|280958171|ref|ZP_06232815.1| channel protein, hemolysin III family [Desulfovibrio aespoeensis Aspo-2]
291087834 10..217    |Clostridium sp. M62/1                         |B|Firmicutes Clostridia              | >gi|291087834|ref|ZP_06347632.2| hemolysin III [Clostridium sp. M62/1]
295090743 8..215     |Clostridium cf. saccharolyticum K10           |B|Firmicutes Clostridia              | >gi|295090743|emb|CBK76850.1| channel protein, hemolysin III family [Clostridium cf. saccharolyticum K10]
295115002 10..217    |butyrate-producing bacterium SM4/1            |B|Firmicutes Clostridia              | >gi|295115002|emb|CBL35849.1| channel protein, hemolysin III family [butyrate-producing bacterium SM4/1]
291172492 33..240    |Filifactor alocis ATCC 35896                  |B|Firmicutes Clostridia              | >gi|291172492|ref|ZP_06573665.1| hemolysin III [Filifactor alocis ATCC 35896]
291522810 27..234    |Coprococcus catus GD/7                        |B|Firmicutes Clostridia              | >gi|291522810|emb|CBK81103.1| channel protein, hemolysin III family [Coprococcus catus GD/7]
295110713 15..222    |Ruminococcus obeum A2-162                     |B|Firmicutes Clostridia              | >gi|295110713|emb|CBL24666.1| channel protein, hemolysin III family [Ruminococcus obeum A2-162]
84702385  16..223    |Parvularcula bermudensis HTCC2503             |B|Proteobacteria Alphaproteobacteria | >gi|84702385|ref|ZP_01016960.1| hemolysin, putative [Parvularcula bermudensis HTCC2503]
111114939 22..228    |Borrelia afzelii PKo                          |B|Spirochaetes Spirochaetales        | >gi|111114939|ref|YP_709557.1| hemolysin III [Borrelia afzelii PKo]
224531536 22..228    |Borrelia valaisiana VS116                     |B|Spirochaetes Spirochaetales        | >gi|224531536|ref|ZP_03672168.1| membrane protein [Borrelia valaisiana VS116]
118444136 19..225    |Clostridium novyi NT                          |B|Firmicutes Clostridia              | >gi|118444136|ref|YP_878378.1| hemolysin III [Clostridium novyi NT]
150007564 7..213     |Parabacteroides distasonis ATCC 8503          |B|Bacteroidetes Bacteroidia          | >gi|150007564|ref|YP_001302307.1| hemolysin III [Parabacteroides distasonis ATCC 8503]
255013913 7..213     |Bacteroides sp. 2_1_7                         |B|Bacteroidetes Bacteroidia          | >gi|255013913|ref|ZP_05286039.1| hemolysin III [Bacteroides sp. 2_1_7]
262381941 7..213     |Bacteroides sp. 2_1_33B                       |B|Bacteroidetes Bacteroidia          | >gi|262381941|ref|ZP_06075079.1| hemolysin III [Bacteroides sp. 2_1_33B]
154482614 16..222    |Eubacterium ventriosum ATCC 27560             |B|Firmicutes Clostridia              | >gi|154482614|ref|ZP_02025062.1| hypothetical protein EUBVEN_00281 [Eubacterium ventriosum ATCC 27560]
154501505 37..243    |Pseudoflavonifractor capillosus ATCC 29799    |B|Firmicutes Clostridia              | >gi|154501505|ref|ZP_02039206.1| hypothetical protein BACCAP_04858 [Bacteroides capillosus ATCC 29799]
154504733 9..215     |Ruminococcus gnavus ATCC 29149                |B|Firmicutes Clostridia              | >gi|154504733|ref|ZP_02041471.1| hypothetical protein RUMGNA_02240 [Ruminococcus gnavus ATCC 29149]
15594463  22..228    |Borrelia burgdorferi B31                      |B|Spirochaetes Spirochaetales        | >gi|15594463|ref|NP_212251.1| hemolysin III (yplQ) [Borrelia burgdorferi B31]
195941855 22..228    |Borrelia burgdorferi 80a                      |B|Spirochaetes Spirochaetales        | >gi|195941855|ref|ZP_03087237.1| hemolysin III (yplQ) [Borrelia burgdorferi 80a]
216264662 22..228    |Borrelia burgdorferi 156a                     |B|Spirochaetes Spirochaetales        | >gi|216264662|ref|ZP_03436654.1| membrane protein [Borrelia burgdorferi 156a]
218249190 22..228    |Borrelia burgdorferi ZS7                      |B|Spirochaetes Spirochaetales        | >gi|218249190|ref|YP_002374645.1| membrane protein [Borrelia burgdorferi ZS7]
221217393 22..228    |Borrelia burgdorferi 72a                      |B|Spirochaetes Spirochaetales        | >gi|221217393|ref|ZP_03588864.1| membrane protein [Borrelia burgdorferi 72a]
223889142 22..228    |Borrelia burgdorferi 64b                      |B|Spirochaetes Spirochaetales        | >gi|223889142|ref|ZP_03623731.1| membrane protein [Borrelia burgdorferi 64b]
225549269 22..228    |Borrelia burgdorferi 94a                      |B|Spirochaetes Spirochaetales        | >gi|225549269|ref|ZP_03770242.1| membrane protein [Borrelia burgdorferi 94a]
226320691 22..228    |Borrelia burgdorferi 29805                    |B|Spirochaetes Spirochaetales        | >gi|226320691|ref|ZP_03796249.1| membrane protein [Borrelia burgdorferi 29805]
160881894 15..221    |Clostridium phytofermentans ISDg              |B|Firmicutes Clostridia              | >gi|160881894|ref|YP_001560862.1| hemolysin III family channel protein [Clostridium phytofermentans ISDg]
160936865 14..220    |Clostridium bolteae ATCC BAA-613              |B|Firmicutes Clostridia              | >gi|160936865|ref|ZP_02084230.1| hypothetical protein CLOBOL_01754 [Clostridium bolteae ATCC BAA-613]
164686938 6..212     |Clostridium bartlettii DSM 16795              |B|Firmicutes Clostridia              | >gi|164686938|ref|ZP_02210966.1| hypothetical protein CLOBAR_00564 [Clostridium bartlettii DSM 16795]
167748395 18..224    |Anaerostipes caccae DSM 14662                 |B|Firmicutes Clostridia              | >gi|167748395|ref|ZP_02420522.1| hypothetical protein ANACAC_03139 [Anaerostipes caccae DSM 14662]
168186861 6..212     |Clostridium botulinum C str. Eklund           |B|Firmicutes Clostridia              | >gi|168186861|ref|ZP_02621496.1| hemolysin-3 [Clostridium botulinum C str. Eklund]
169351353 20..226    |Clostridium spiroforme DSM 1552               |B|Firmicutes Erysipelotrichi         | >gi|169351353|ref|ZP_02868291.1| hypothetical protein CLOSPI_02133 [Clostridium spiroforme DSM 1552]
210614159 8..214     |Clostridium nexile DSM 1787                   |B|Firmicutes Clostridia              | >gi|210614159|ref|ZP_03290079.1| hypothetical protein CLONEX_02292 [Clostridium nexile DSM 1787]
218134055 17..223    |Bacteroides pectinophilus ATCC 43243          |B|Bacteroidetes Bacteroidia          | >gi|218134055|ref|ZP_03462859.1| hypothetical protein BACPEC_01945 [Bacteroides pectinophilus ATCC 43243]
219684866 22..228    |Borrelia garinii PBr                          |B|Spirochaetes Spirochaetales        | >gi|219684866|ref|ZP_03539808.1| membrane protein [Borrelia garinii PBr]
219685344 22..228    |Borrelia garinii Far04                        |B|Spirochaetes Spirochaetales        | >gi|219685344|ref|ZP_03540163.1| membrane protein [Borrelia garinii Far04]
224534887 22..228    |Borrelia spielmanii A14S                      |B|Spirochaetes Spirochaetales        | >gi|224534887|ref|ZP_03675456.1| membrane protein [Borrelia spielmanii A14S]
51598380  22..228    |Borrelia garinii PBi                          |B|Spirochaetes Spirochaetales        | >gi|51598380|ref|YP_072568.1| hemolysin III [Borrelia garinii PBi]
224001852 4..210     |Thalassiosira pseudonana CCMP1335             |E|stramenopiles Bacillariophyta      | >gi|224001852|ref|XP_002290598.1| hemolysin ii-like protein [Thalassiosira pseudonana CCMP1335]
224541420 32..238    |Catenibacterium mitsuokai DSM 15897           |B|Firmicutes Erysipelotrichi         | >gi|224541420|ref|ZP_03681959.1| hypothetical protein CATMIT_00582 [Catenibacterium mitsuokai DSM 15897]
225028962 10..216    |Eubacterium hallii DSM 3353                   |B|Firmicutes Clostridia              | >gi|225028962|ref|ZP_03718154.1| hypothetical protein EUBHAL_03254 [Eubacterium hallii DSM 3353]
225420030 14..220    |Clostridium asparagiforme DSM 15981           |B|Firmicutes Clostridia              | >gi|225420030|ref|ZP_03762333.1| hypothetical protein CLOSTASPAR_06373 [Clostridium asparagiforme DSM 15981]
225570103 11..217    |Clostridium hylemonae DSM 15053               |B|Firmicutes Clostridia              | >gi|225570103|ref|ZP_03779128.1| hypothetical protein CLOHYLEM_06199 [Clostridium hylemonae DSM 15053]
225572759 7..213     |Blautia hydrogenotrophica DSM 10507           |B|Firmicutes Clostridia              | >gi|225572759|ref|ZP_03781514.1| hypothetical protein RUMHYD_00949 [Blautia hydrogenotrophica DSM 10507]
237784974 26..232    |Corynebacterium kroppenstedtii DSM 44385      |B|Actinobacteria Actinobacteridae    | >gi|237784974|ref|YP_002905679.1| Hly III family transporter [Corynebacterium kroppenstedtii DSM 44385]
240144532 8..214     |Roseburia intestinalis L1-82                  |B|Firmicutes Clostridia              | >gi|240144532|ref|ZP_04743133.1| hemolysin III [Roseburia intestinalis L1-82]
291534319 8..214     |Roseburia intestinalis M50/1                  |B|Firmicutes Clostridia              | >gi|291534319|emb|CBL07431.1| channel protein, hemolysin III family [Roseburia intestinalis M50/1]
253580979 13..219    |Ruminococcus sp. 5_1_39BFAA                   |B|Firmicutes Clostridia              | >gi|253580979|ref|ZP_04858241.1| conserved hypothetical protein [Ruminococcus sp. 5_1_39B_FAA]
253682552 6..212     |Clostridium botulinum D str. 1873             |B|Firmicutes Clostridia              | >gi|253682552|ref|ZP_04863349.1| hemolysin-3 [Clostridium botulinum D str. 1873]
255283049 13..219    |Marvinbryantia formatexigens DSM 14469        |B|Firmicutes Clostridia              | >gi|255283049|ref|ZP_05347604.1| hemolysin III [Bryantella formatexigens DSM 14469]
260588778 7..213     |Blautia hansenii DSM 20583                    |B|Firmicutes Clostridia              | >gi|260588778|ref|ZP_05854691.1| hemolysin III [Blautia hansenii DSM 20583]
283846323 6..212     |Bacillus cellulosilyticus DSM 2522            |B|Firmicutes Bacillales              | >gi|283846323|ref|ZP_06363791.1| channel protein, hemolysin III family [Bacillus cellulosilyticus DSM 2522]
291547797 7..213     |Ruminococcus sp. SR1/5                        |B|Firmicutes Clostridia              | >gi|291547797|emb|CBL20905.1| channel protein, hemolysin III family [Ruminococcus sp. SR1/5]
110799984 7..212     |Clostridium perfringens ATCC 13124            |B|Firmicutes Clostridia              | >gi|110799984|ref|YP_696161.1| hemolysin III [Clostridium perfringens ATCC 13124]
168210062 7..212     |Clostridium perfringens B str. ATCC 3626      |B|Firmicutes Clostridia              | >gi|168210062|ref|ZP_02635687.1| hemolysin III [Clostridium perfringens B str. ATCC 3626]
168213754 7..212     |Clostridium perfringens CPE str. F4969        |B|Firmicutes Clostridia              | >gi|168213754|ref|ZP_02639379.1| hemolysin III [Clostridium perfringens CPE str. F4969]
18310456  7..212     |Clostridium perfringens str. 13               |B|Firmicutes Clostridia              | >gi|18310456|ref|NP_562390.1| hemolysin III [Clostridium perfringens str. 13]
110802029 8..212     |Clostridium perfringens SM101                 |B|Firmicutes Clostridia              | >gi|110802029|ref|YP_698772.1| hemolysin III [Clostridium perfringens SM101]
134298297 6..211     |Desulfotomaculum reducens MI-1                |B|Firmicutes Clostridia              | >gi|134298297|ref|YP_001111793.1| hemolysin III family channel protein [Desulfotomaculum reducens MI-1]
148379555 6..211     |Clostridium botulinum A str. ATCC 3502        |B|Firmicutes Clostridia              | >gi|148379555|ref|YP_001254096.1| hemolysin [Clostridium botulinum A str. ATCC 3502]
168180243 6..211     |Clostridium botulinum NCTC 2916               |B|Firmicutes Clostridia              | >gi|168180243|ref|ZP_02614907.1| hemolysin III [Clostridium botulinum NCTC 2916]
168182522 6..211     |Clostridium botulinum Bf                      |B|Firmicutes Clostridia              | >gi|168182522|ref|ZP_02617186.1| channel protein, hemolysin III family [Clostridium botulinum Bf]
170755546 6..211     |Clostridium botulinum B1 str. Okra            |B|Firmicutes Clostridia              | >gi|170755546|ref|YP_001781225.1| hemolysin III [Clostridium botulinum B1 str. Okra]
170761574 6..211     |Clostridium botulinum A3 str. Loch Maree      |B|Firmicutes Clostridia              | >gi|170761574|ref|YP_001786998.1| hemolysin III [Clostridium botulinum A3 str. Loch Maree]
150014915 7..212     |Clostridium beijerinckii NCIMB 8052           |B|Firmicutes Clostridia              | >gi|150014915|ref|YP_001307169.1| hemolysin III family channel protein [Clostridium beijerinckii NCIMB 8052]
153854356 13..218    |Dorea longicatena DSM 13814                   |B|Firmicutes Clostridia              | >gi|153854356|ref|ZP_01995655.1| hypothetical protein DORLON_01650 [Dorea longicatena DSM 13814]
158319450 15..220    |Alkaliphilus oremlandii OhILAs                |B|Firmicutes Clostridia              | >gi|158319450|ref|YP_001511957.1| hemolysin III family channel protein [Alkaliphilus oremlandii OhILAs]
166033047 11..216    |Dorea formicigenerans ATCC 27755              |B|Firmicutes Clostridia              | >gi|166033047|ref|ZP_02235876.1| hypothetical protein DORFOR_02769 [Dorea formicigenerans ATCC 27755]
167752318 31..236    |Alistipes putredinis DSM 17216                |B|Bacteroidetes Bacteroidia          | >gi|167752318|ref|ZP_02424445.1| hypothetical protein ALIPUT_00562 [Alistipes putredinis DSM 17216]
167755823 9..214     |Clostridium ramosum DSM 1402                  |B|Firmicutes Erysipelotrichi         | >gi|167755823|ref|ZP_02427950.1| hypothetical protein CLORAM_01339 [Clostridium ramosum DSM 1402]
237734792 9..214     |Coprobacillus sp. D7                          |B|Firmicutes Erysipelotrichi         | >gi|237734792|ref|ZP_04565273.1| hemolysin [Mollicutes bacterium D7]
167760265 11..216    |Clostridium scindens ATCC 35704               |B|Firmicutes Clostridia              | >gi|167760265|ref|ZP_02432392.1| hypothetical protein CLOSCI_02638 [Clostridium scindens ATCC 35704]
167768436 13..218    |Clostridium sp. SS2/1                         |B|Firmicutes Clostridia              | >gi|167768436|ref|ZP_02440489.1| hypothetical protein CLOSS21_02995 [Clostridium sp. SS2/1]
291560411 7..212     |butyrate-producing bacterium SSC/2            |B|Firmicutes Clostridia              | >gi|291560411|emb|CBL39211.1| channel protein, hemolysin III family [butyrate-producing bacterium SSC/2]
169350185 9..214     |Clostridium spiroforme DSM 1552               |B|Firmicutes Erysipelotrichi         | >gi|169350185|ref|ZP_02867123.1| hypothetical protein CLOSPI_00929 [Clostridium spiroforme DSM 1552]
172057197 7..212     |Exiguobacterium sibiricum 255-15              |B|Firmicutes Bacillales              | >gi|172057197|ref|YP_001813657.1| hemolysin III family channel protein [Exiguobacterium sibiricum 255-15]
182419992 7..212     |Clostridium butyricum 5521                    |B|Firmicutes Clostridia              | >gi|182419992|ref|ZP_02951226.1| hemolysin-3 [Clostridium butyricum 5521]
187934459 7..212     |Clostridium botulinum B str. Eklund 17B       |B|Firmicutes Clostridia              | >gi|187934459|ref|YP_001884310.1| hemolysin-3 [Clostridium botulinum B str. Eklund 17B]
188589054 7..212     |Clostridium botulinum E3 str. Alaska E43      |B|Firmicutes Clostridia              | >gi|188589054|ref|YP_001919494.1| hemolysin-3 [Clostridium botulinum E3 str. Alaska E43]
251778171 7..212     |Clostridium botulinum E1 str. 'BoNT E Beluga' |B|Firmicutes Clostridia              | >gi|251778171|ref|ZP_04821091.1| hemolysin-3 [Clostridium botulinum E1 str. 'BoNT E Beluga']
196040941 7..212     |Bacillus cereus NVH0597-99                    |B|Firmicutes Bacillales              | >gi|196040941|ref|ZP_03108238.1| channel protein, hemolysin III family [Bacillus cereus NVH0597-99]
229099879 7..211     |Bacillus cereus Rock3-29                      |B|Firmicutes Bacillales              | >gi|229099879|ref|ZP_04230803.1| hypothetical protein bcere0020_50970 [Bacillus cereus Rock3-29]
229106037 7..211     |Bacillus cereus Rock3-28                      |B|Firmicutes Bacillales              | >gi|229106037|ref|ZP_04236658.1| hypothetical protein bcere0019_51610 [Bacillus cereus Rock3-28]
229118941 7..211     |Bacillus cereus Rock1-3                       |B|Firmicutes Bacillales              | >gi|229118941|ref|ZP_04248288.1| hypothetical protein bcere0017_52050 [Bacillus cereus Rock1-3]
229164402 7..211     |Bacillus cereus R309803                       |B|Firmicutes Bacillales              | >gi|229164402|ref|ZP_04292331.1| hypothetical protein bcere0009_51600 [Bacillus cereus R309803]
197301427 9..214     |Ruminococcus lactaris ATCC 29176              |B|Firmicutes Clostridia              | >gi|197301427|ref|ZP_03166507.1| hypothetical protein RUMLAC_00158 [Ruminococcus lactaris ATCC 29176]
205375152 7..212     |Bacillus coahuilensis m4-4                    |B|Firmicutes Bacillales              | >gi|205375152|ref|ZP_03227943.1| hemolysin III family channel protein [Bacillus coahuilensis m4-4]
210624314 8..213     |Clostridium hiranonis DSM 13275               |B|Firmicutes Clostridia              | >gi|210624314|ref|ZP_03294318.1| hypothetical protein CLOHIR_02274 [Clostridium hiranonis DSM 13275]
212635135 8..213     |Shewanella piezotolerans WP3                  |B|Proteobacteria Gammaproteobacteria | >gi|212635135|ref|YP_002311660.1| hemolysin III family channel protein [Shewanella piezotolerans WP3]
224368877 34..239    |Desulfobacterium autotrophicum HRM2           |B|Proteobacteria Deltaproteobacteria | >gi|224368877|ref|YP_002603039.1| channel protein (hemolysin III family protein) [Desulfobacterium autotrophicum HRM2]
226325569 9..214     |Coprococcus comes ATCC 27758                  |B|Firmicutes Clostridia              | >gi|226325569|ref|ZP_03801087.1| hypothetical protein COPCOM_03374 [Coprococcus comes ATCC 27758]
226355480 6..211     |Deinococcus deserti VCD115                    |B|Deinococcus-Thermus Deinococci     | >gi|226355480|ref|YP_002785220.1| hypothetical protein Deide_06220 [Deinococcus deserti VCD115]
228961714 7..212     |Bacillus thuringiensis serovar pakistani str. T13001|B|Firmicutes Bacillales              | >gi|228961714|ref|ZP_04123320.1| hypothetical protein bthur0005_51540 [Bacillus thuringiensis serovar pakistani str. T13001]
49481225  7..212     |Bacillus thuringiensis serovar konkukian str. 97-27|B|Firmicutes Bacillales              | >gi|49481225|ref|YP_039441.1| hemolysin III-like protein [Bacillus thuringiensis serovar konkukian str. 97-27]
163943133 7..211     |Bacillus weihenstephanensis KBAB4             |B|Firmicutes Bacillales              | >gi|163943133|ref|YP_001648017.1| hemolysin III family channel protein [Bacillus weihenstephanensis KBAB4]
218900581 7..211     |Bacillus cereus G9842                         |B|Firmicutes Bacillales              | >gi|218900581|ref|YP_002448992.1| channel protein, hemolysin III family [Bacillus cereus G9842]
228911290 12..216    |Bacillus thuringiensis IBL 200                |B|Firmicutes Bacillales              | >gi|228911290|ref|ZP_04075094.1| hypothetical protein bthur0013_54280 [Bacillus thuringiensis IBL 200]
228942599 7..211     |Bacillus thuringiensis serovar berliner ATCC 10792|B|Firmicutes Bacillales              | >gi|228942599|ref|ZP_04105132.1| hypothetical protein bthur0008_52270 [Bacillus thuringiensis serovar berliner ATCC 10792]
228955700 7..211     |Bacillus thuringiensis serovar kurstaki str. T03a001|B|Firmicutes Bacillales              | >gi|228955700|ref|ZP_04117697.1| hypothetical protein bthur0006_50490 [Bacillus thuringiensis serovar kurstaki str. T03a001]
229014617 7..211     |Bacillus mycoides DSM 2048                    |B|Firmicutes Bacillales              | >gi|229014617|ref|ZP_04171732.1| hypothetical protein bmyco0001_50180 [Bacillus mycoides DSM 2048]
229020925 12..216    |Bacillus cereus AH1273                        |B|Firmicutes Bacillales              | >gi|229020925|ref|ZP_04177616.1| hypothetical protein bcere0030_53760 [Bacillus cereus AH1273]
229136277 12..216    |Bacillus cereus BDRD-ST196                    |B|Firmicutes Bacillales              | >gi|229136277|ref|ZP_04265024.1| hypothetical protein bcere0014_51460 [Bacillus cereus BDRD-ST196]
75762845  12..216    |Bacillus thuringiensis serovar israelensis ATCC 35646|B|Firmicutes Bacillales              | >gi|75762845|ref|ZP_00742663.1| Conserved membrane protein (hemolysin III homolog) [Bacillus thuringiensis serovar israelensis ATCC 35646]
229827308 24..229    |Abiotrophia defectiva ATCC 49176              |B|Firmicutes Lactobacillales         | >gi|229827308|ref|ZP_04453377.1| hypothetical protein GCWU000182_02694 [Abiotrophia defectiva ATCC 49176]
238916013 27..232    |Eubacterium eligens ATCC 27750                |B|Firmicutes Clostridia              | >gi|238916013|ref|YP_002929530.1| hypothetical protein EUBELI_00046 [Eubacterium eligens ATCC 27750]
242262866 6..211     |Clostridium cellulovorans 743B                |B|Firmicutes Clostridia              | >gi|242262866|ref|ZP_04807527.1| channel protein, hemolysin III family [Clostridium cellulovorans 743B]
258513564 6..211     |Desulfotomaculum acetoxidans DSM 771          |B|Firmicutes Clostridia              | >gi|258513564|ref|YP_003189786.1| channel protein, hemolysin III family [Desulfotomaculum acetoxidans DSM 771]
266622466 7..212     |Clostridium hathewayi DSM 13479               |B|Firmicutes Clostridia              | >gi|266622466|ref|ZP_06115401.1| hemolysin III [Clostridium hathewayi DSM 13479]
28563938  7..212     |Oceanobacillus iheyensis HTE831               |B|Firmicutes Bacillales              | >gi|28563938|ref|NP_693331.2| hemolysin III [Oceanobacillus iheyensis HTE831]
22778096  1..204     |Oceanobacillus iheyensis HTE831               |B|Firmicutes Bacillales              | >gi|22778096|dbj|BAC14366.1| hemolysin III [Oceanobacillus iheyensis HTE831]
289422517 8..213     |Peptostreptococcus anaerobius 653-L           |B|Firmicutes Clostridia              | >gi|289422517|ref|ZP_06424360.1| hemolysin-3 [Peptostreptococcus anaerobius 653-L]
291514933 11..216    |Alistipes shahii WAL 8301                     |B|Bacteroidetes Bacteroidia          | >gi|291514933|emb|CBK64143.1| Predicted membrane protein, hemolysin III homolog [Alistipes shahii WAL 8301]
291549125 9..214     |Ruminococcus torques L2-14                    |B|Firmicutes Clostridia              | >gi|291549125|emb|CBL25387.1| channel protein, hemolysin III family [Ruminococcus torques L2-14]
291562537 7..212     |butyrate-producing bacterium SS3/4            |B|Firmicutes Clostridia              | >gi|291562537|emb|CBL41353.1| channel protein, hemolysin III family [butyrate-producing bacterium SS3/4]
293375775 7..212     |Turicibacter sanguinis PC909                  |B|Firmicutes Erysipelotrichi         | >gi|293375775|ref|ZP_06622045.1| channel protein, hemolysin III family protein [Turicibacter sanguinis PC909]
297622807 15..220    |Truepera radiovictrix DSM 17093               |B|Deinococcus-Thermus Deinococci     | >gi|297622807|ref|YP_003704241.1| channel protein, hemolysin III family [Truepera radiovictrix DSM 17093]
89096431  7..212     |Bacillus sp. NRRL B-14911                     |B|Firmicutes Bacillales              | >gi|89096431|ref|ZP_01169324.1| hypothetical protein B14911_27080 [Bacillus sp. NRRL B-14911]
108801102 38..242    |Mycobacterium sp. MCS                         |B|Actinobacteria Actinobacteridae    | >gi|108801102|ref|YP_641299.1| hemolysin III family channel protein [Mycobacterium sp. MCS]
111022818 14..218    |Rhodococcus jostii RHA1                       |B|Actinobacteria Actinobacteridae    | >gi|111022818|ref|YP_705790.1| hemolytic factor [Rhodococcus jostii RHA1]
226365326 14..218    |Rhodococcus opacus B4                         |B|Actinobacteria Actinobacteridae    | >gi|226365326|ref|YP_002783109.1| hemolysin III family protein [Rhodococcus opacus B4]
114567418 23..227    |Syntrophomonas wolfei subsp. wolfei str. Goettingen|B|Firmicutes Clostridia              | >gi|114567418|ref|YP_754572.1| hemolysin III-like protein [Syntrophomonas wolfei subsp. wolfei str. Goettingen]
118464088 39..243    |Mycobacterium avium 104                       |B|Actinobacteria Actinobacteridae    | >gi|118464088|ref|YP_880456.1| channel protein, hemolysin III family protein [Mycobacterium avium 104]
41408802  39..243    |Mycobacterium avium subsp. paratuberculosis K-10|B|Actinobacteria Actinobacteridae    | >gi|41408802|ref|NP_961638.1| hypothetical protein MAP2704 [Mycobacterium avium subsp. paratuberculosis K-10]
118472546 49..253    |Mycobacterium smegmatis str. MC2 155          |B|Actinobacteria Actinobacteridae    | >gi|118472546|ref|YP_889503.1| channel protein, hemolysin III family protein [Mycobacterium smegmatis str. MC2 155]
118480480 12..216    |Bacillus thuringiensis str. Al Hakam          |B|Firmicutes Bacillales              | >gi|118480480|ref|YP_897631.1| hemolysin III [Bacillus thuringiensis str. Al Hakam]
196036080 7..211     |Bacillus cereus W                             |B|Firmicutes Bacillales              | >gi|196036080|ref|ZP_03103480.1| channel protein, hemolysin III family [Bacillus cereus W]
206970097 7..211     |Bacillus cereus AH1134                        |B|Firmicutes Bacillales              | >gi|206970097|ref|ZP_03231050.1| channel protein, hemolysin III family [Bacillus cereus AH1134]
206975808 7..211     |Bacillus cereus H3081.97                      |B|Firmicutes Bacillales              | >gi|206975808|ref|ZP_03236719.1| channel protein, hemolysin III family [Bacillus cereus H3081.97]
218906632 7..211     |Bacillus cereus AH820                         |B|Firmicutes Bacillales              | >gi|218906632|ref|YP_002454466.1| channel protein, hemolysin III family [Bacillus cereus AH820]
228924196 12..216    |Bacillus thuringiensis serovar huazhongensis BGSC 4BD1|B|Firmicutes Bacillales              | >gi|228924196|ref|ZP_04087468.1| hypothetical protein bthur0011_51670 [Bacillus thuringiensis serovar huazhongensis BGSC 4BD1]
228930458 12..216    |Bacillus thuringiensis serovar pondicheriensis BGSC 4BA1|B|Firmicutes Bacillales              | >gi|228930458|ref|ZP_04093458.1| hypothetical protein bthur0010_51360 [Bacillus thuringiensis serovar pondicheriensis BGSC 4BA1]
229142202 12..216    |Bacillus cereus BDRD-ST26                     |B|Firmicutes Bacillales              | >gi|229142202|ref|ZP_04270726.1| hypothetical protein bcere0013_52870 [Bacillus cereus BDRD-ST26]
229159012 12..216    |Bacillus cereus ATCC 4342                     |B|Firmicutes Bacillales              | >gi|229159012|ref|ZP_04287068.1| hypothetical protein bcere0010_51830 [Bacillus cereus ATCC 4342]
229176126 12..216    |Bacillus cereus MM3                           |B|Firmicutes Bacillales              | >gi|229176126|ref|ZP_04303619.1| hypothetical protein bcere0006_51940 [Bacillus cereus MM3]
229181698 12..216    |Bacillus cereus 172560W                       |B|Firmicutes Bacillales              | >gi|229181698|ref|ZP_04309022.1| hypothetical protein bcere0005_50370 [Bacillus cereus 172560W]
229199582 12..216    |Bacillus cereus m1293                         |B|Firmicutes Bacillales              | >gi|229199582|ref|ZP_04326243.1| hypothetical protein bcere0001_50800 [Bacillus cereus m1293]
30023478  7..211     |Bacillus cereus ATCC 14579                    |B|Firmicutes Bacillales              | >gi|30023478|ref|NP_835109.1| hemolysin III [Bacillus cereus ATCC 14579]
30265470  7..211     |Bacillus anthracis str. Ames                  |B|Firmicutes Bacillales              | >gi|30265470|ref|NP_847847.1| hemolysin III family channel protein [Bacillus anthracis str. Ames]
42784635  7..211     |Bacillus cereus ATCC 10987                    |B|Firmicutes Bacillales              | >gi|42784635|ref|NP_981882.1| hemolysin III family channel protein [Bacillus cereus ATCC 10987]
47568572  7..211     |Bacillus cereus G9241                         |B|Firmicutes Bacillales              | >gi|47568572|ref|ZP_00239271.1| hemolysin III [Bacillus cereus G9241]
52145258  7..211     |Bacillus cereus E33L                          |B|Firmicutes Bacillales              | >gi|52145258|ref|YP_086715.1| hemolysin III-like protein [Bacillus cereus E33L]
228918063 3..198     |Bacillus thuringiensis serovar pulsiensis BGSC 4CC1|B|Firmicutes Bacillales              | >gi|228918063|ref|ZP_04081592.1| hypothetical protein bthur0012_52600 [Bacillus thuringiensis serovar pulsiensis BGSC 4CC1]
228988679 3..198     |Bacillus thuringiensis serovar tochigiensis BGSC 4Y1|B|Firmicutes Bacillales              | >gi|228988679|ref|ZP_04148765.1| hypothetical protein bthur0001_53340 [Bacillus thuringiensis serovar tochigiensis BGSC 4Y1]
229035095 3..198     |Bacillus cereus AH1271                        |B|Firmicutes Bacillales              | >gi|229035095|ref|ZP_04189042.1| hypothetical protein bcere0028_51210 [Bacillus cereus AH1271]
229094563 3..198     |Bacillus cereus Rock3-42                      |B|Firmicutes Bacillales              | >gi|229094563|ref|ZP_04225630.1| hypothetical protein bcere0021_52660 [Bacillus cereus Rock3-42]
229187680 3..198     |Bacillus cereus BGSC 6E1                      |B|Firmicutes Bacillales              | >gi|229187680|ref|ZP_04314817.1| hypothetical protein bcere0004_52120 [Bacillus cereus BGSC 6E1]
65317433  3..198     |Bacillus anthracis str. A2012                 |B|Firmicutes Bacillales              | >gi|65317433|ref|ZP_00390392.1| COG1272: Predicted membrane protein, hemolysin III homolog [Bacillus anthracis str. A2012]
254733559 1..152     |Bacillus anthracis str. Western North America USA6153|B|Firmicutes Bacillales              | >gi|254733559|ref|ZP_05191280.1| channel protein, hemolysin III family [Bacillus anthracis str. Western North America USA6153]
254733616 7..117     |Bacillus anthracis str. Western North America USA6153|B|Firmicutes Bacillales              | >gi|254733616|ref|ZP_05191333.1| hemolysin III [Bacillus anthracis str. Western North America USA6153]
118616084 39..243    |Mycobacterium ulcerans Agy99                  |B|Actinobacteria Actinobacteridae    | >gi|118616084|ref|YP_904416.1| hemolysin-like protein [Mycobacterium ulcerans Agy99]
183984352 39..243    |Mycobacterium marinum M                       |B|Actinobacteria Actinobacteridae    | >gi|183984352|ref|YP_001852643.1| hemolysin-like protein [Mycobacterium marinum M]
120405615 36..240    |Mycobacterium vanbaalenii PYR-1               |B|Actinobacteria Actinobacteridae    | >gi|120405615|ref|YP_955444.1| hemolysin III family channel protein [Mycobacterium vanbaalenii PYR-1]
145222642 43..247    |Mycobacterium gilvum PYR-GCK                  |B|Actinobacteria Actinobacteridae    | >gi|145222642|ref|YP_001133320.1| hemolysin III family channel protein [Mycobacterium gilvum PYR-GCK]
134097518 13..217    |Saccharopolyspora erythraea NRRL 2338         |B|Actinobacteria Actinobacteridae    | >gi|134097518|ref|YP_001103179.1| hemolysin III family channel protein [Saccharopolyspora erythraea NRRL 2338]
150391948 12..216    |Alkaliphilus metalliredigens QYMF             |B|Firmicutes Clostridia              | >gi|150391948|ref|YP_001321997.1| hemolysin III family channel protein [Alkaliphilus metalliredigens QYMF]
15608225  35..239    |Mycobacterium tuberculosis H37Rv              |B|Actinobacteria Actinobacteridae    | >gi|15608225|ref|NP_215601.1| hemolysin-like protein [Mycobacterium tuberculosis H37Rv]
289569073 15..219    |Mycobacterium tuberculosis T17                |B|Actinobacteria Actinobacteridae    | >gi|289569073|ref|ZP_06449300.1| LOW QUALITY PROTEIN: hemolysin [Mycobacterium tuberculosis T17]
219556963 4..198     |Mycobacterium tuberculosis T17                |B|Actinobacteria Actinobacteridae    | >gi|219556963|ref|ZP_03536039.1| hemolysin-like protein [Mycobacterium tuberculosis T17]
289555132 15..208    |Mycobacterium tuberculosis KZN 605            |B|Actinobacteria Actinobacteridae    | >gi|289555132|ref|ZP_06444342.1| LOW QUALITY PROTEIN: hemolysin [Mycobacterium tuberculosis KZN 605]
215429951 51..237    |Mycobacterium tuberculosis EAS054             |B|Actinobacteria Actinobacteridae    | >gi|215429951|ref|ZP_03427870.1| hemolysin-like protein [Mycobacterium tuberculosis EAS054]
289753148 35..221    |Mycobacterium tuberculosis EAS054             |B|Actinobacteria Actinobacteridae    | >gi|289753148|ref|ZP_06512526.1| LOW QUALITY PROTEIN: membrane protein [Mycobacterium tuberculosis EAS054]
218752765 51..171    |Mycobacterium tuberculosis GM 1503            |B|Actinobacteria Actinobacteridae    | >gi|218752765|ref|ZP_03531561.1| hemolysin-like protein [Mycobacterium tuberculosis GM 1503]
289761223 35..155    |Mycobacterium tuberculosis GM 1503            |B|Actinobacteria Actinobacteridae    | >gi|289761223|ref|ZP_06520601.1| LOW QUALITY PROTEIN: hypothetical hemolysin-like protein [Mycobacterium tuberculosis GM 1503]
167966577 51..164    |Mycobacterium tuberculosis H37Ra              |B|Actinobacteria Actinobacteridae    | >gi|167966577|ref|ZP_02548854.1| hypothetical hemolysin-like protein [Mycobacterium tuberculosis H37Ra]
15640021  32..236    |Treponema pallidum subsp. pallidum str. Nichols|B|Spirochaetes Spirochaetales        | >gi|15640021|ref|NP_219474.1| hemolysin III (hlyIII) [Treponema pallidum subsp. pallidum str. Nichols]
160882729 10..214    |Bacteroides ovatus ATCC 8483                  |B|Bacteroidetes Bacteroidia          | >gi|160882729|ref|ZP_02063732.1| hypothetical protein BACOVA_00687 [Bacteroides ovatus ATCC 8483]
237714007 10..214    |Bacteroides sp. D1                            |B|Bacteroidetes Bacteroidia          | >gi|237714007|ref|ZP_04544488.1| hemolysin III [Bacteroides sp. D1]
260174003 10..214    |Bacteroides sp. D2                            |B|Bacteroidetes Bacteroidia          | >gi|260174003|ref|ZP_05760415.1| hemolysin III [Bacteroides sp. D2]
298479679 10..214    |Bacteroides sp. D22                           |B|Bacteroidetes Bacteroidia          | >gi|298479679|ref|ZP_06997879.1| hemolysin III [Bacteroides sp. D22]
168703517 6..210     |Gemmata obscuriglobus UQM 2246                |B|Planctomycetes Planctomycetacia    | >gi|168703517|ref|ZP_02735794.1| membrane protein, hemolysin III-like protein [Gemmata obscuriglobus UQM 2246]
210631931 13..217    |Collinsella stercoris DSM 13279               |B|Actinobacteria Coriobacteridae     | >gi|210631931|ref|ZP_03297120.1| hypothetical protein COLSTE_01010 [Collinsella stercoris DSM 13279]
219120285 54..258    |Phaeodactylum tricornutum CCAP 1055/1         |E|stramenopiles Bacillariophyta      | >gi|219120285|ref|XP_002180884.1| predicted protein [Phaeodactylum tricornutum CCAP 1055/1]
226307743 21..225    |Rhodococcus erythropolis PR4                  |B|Actinobacteria Actinobacteridae    | >gi|226307743|ref|YP_002767703.1| hypothetical protein RER_42560 [Rhodococcus erythropolis PR4]
229493942 14..218    |Rhodococcus erythropolis SK121                |B|Actinobacteria Actinobacteridae    | >gi|229493942|ref|ZP_04387714.1| channel protein, hemolysin III family [Rhodococcus erythropolis SK121]
227486285 7..211     |Anaerococcus lactolyticus ATCC 51172          |B|Firmicutes Clostridia              | >gi|227486285|ref|ZP_03916601.1| Hly III family protein [Anaerococcus lactolyticus ATCC 51172]
228994165 3..207     |Bacillus pseudomycoides DSM 12442             |B|Firmicutes Bacillales              | >gi|228994165|ref|ZP_04154065.1| hypothetical protein bpmyx0001_48880 [Bacillus pseudomycoides DSM 12442]
229000234 3..207     |Bacillus mycoides Rock3-17                    |B|Firmicutes Bacillales              | >gi|229000234|ref|ZP_04159803.1| hypothetical protein bmyco0003_47870 [Bacillus mycoides Rock3-17]
229007757 7..211     |Bacillus mycoides Rock1-4                     |B|Firmicutes Bacillales              | >gi|229007757|ref|ZP_04165348.1| hypothetical protein bmyco0002_46350 [Bacillus mycoides Rock1-4]
229087935 7..211     |Bacillus cereus Rock3-44                      |B|Firmicutes Bacillales              | >gi|229087935|ref|ZP_04220046.1| hypothetical protein bcere0022_44900 [Bacillus cereus Rock3-44]
229816091 68..272    |Collinsella intestinalis DSM 13280            |B|Actinobacteria Coriobacteridae     | >gi|229816091|ref|ZP_04446412.1| hypothetical protein COLINT_03147 [Collinsella intestinalis DSM 13280]
240170281 39..243    |Mycobacterium kansasii ATCC 12478             |B|Actinobacteria Actinobacteridae    | >gi|240170281|ref|ZP_04748940.1| hemolysin-like protein [Mycobacterium kansasii ATCC 12478]
242279803 6..210     |Desulfovibrio salexigens DSM 2638             |B|Proteobacteria Deltaproteobacteria | >gi|242279803|ref|YP_002991932.1| channel protein, hemolysin III family [Desulfovibrio salexigens DSM 2638]
253570556 23..227    |Bacteroides sp. 1_1_6                         |B|Bacteroidetes Bacteroidia          | >gi|253570556|ref|ZP_04847964.1| hemolysin III [Bacteroides sp. 1_1_6]
29347393  23..227    |Bacteroides thetaiotaomicron VPI-5482         |B|Bacteroidetes Bacteroidia          | >gi|29347393|ref|NP_810896.1| hemolysin III [Bacteroides thetaiotaomicron VPI-5482]
254520385 7..211     |Clostridium sp. 7_2_43FAA                     |B|Firmicutes Clostridia              | >gi|254520385|ref|ZP_05132441.1| hemolysin III family channel protein [Clostridium sp. 7_2_43FAA]
254819723 39..243    |Mycobacterium intracellulare ATCC 13950       |B|Actinobacteria Actinobacteridae    | >gi|254819723|ref|ZP_05224724.1| channel protein, hemolysin III family protein [Mycobacterium intracellulare ATCC 13950]
256374862 17..221    |Actinosynnema mirum DSM 43827                 |B|Actinobacteria Actinobacteridae    | >gi|256374862|ref|YP_003098522.1| channel protein, hemolysin III family [Actinosynnema mirum DSM 43827]
256824374 19..223    |Kytococcus sedentarius DSM 20547              |B|Actinobacteria Actinobacteridae    | >gi|256824374|ref|YP_003148334.1| hemolysin III [Kytococcus sedentarius DSM 20547]
257456670 10..214    |Treponema vincentii ATCC 35580                |B|Spirochaetes Spirochaetales        | >gi|257456670|ref|ZP_05621863.1| hemolysin-3 [Treponema vincentii ATCC 35580]
258560885 25..229    |Corynebacterium genitalium ATCC 33030         |B|Actinobacteria Actinobacteridae    | >gi|258560885|ref|ZP_05707559.1| Hly-III family protein [Corynebacterium genitalium ATCC 33030]
258654534 15..219    |Nakamurella multipartita DSM 44233            |B|Actinobacteria Actinobacteridae    | >gi|258654534|ref|YP_003203690.1| hemolysin III family channel protein [Nakamurella multipartita DSM 44233]
261338538 49..253    |Bifidobacterium gallicum DSM 20093            |B|Actinobacteria Actinobacteridae    | >gi|261338538|ref|ZP_05966422.1| membrane protein, hemolysin III-like protein [Bifidobacterium gallicum DSM 20093]
268563731 350..554   |Caenorhabditis briggsae                       |E|Metazoa Nematoda                   | >gi|268563731|ref|XP_002638920.1| Hypothetical protein CBG22146 [Caenorhabditis briggsae]
282860163 4..208     |Prevotella bivia JCVIHMP010                   |B|Bacteroidetes Bacteroidia          | >gi|282860163|ref|ZP_06269238.1| channel protein, hemolysin III family protein [Prevotella bivia JCVIHMP010]
291287055 5..209     |Denitrovibrio acetiphilus DSM 12809           |B|Deferribacteres Deferribacterales  | >gi|291287055|ref|YP_003503871.1| hemolysin III family channel protein [Denitrovibrio acetiphilus DSM 12809]
296130697 30..234    |Cellulomonas flavigena DSM 20109              |B|Actinobacteria Actinobacteridae    | >gi|296130697|ref|YP_003637947.1| Hly-III family protein [Cellulomonas flavigena DSM 20109]
296169954 42..246    |Mycobacterium parascrofulaceum ATCC BAA-614   |B|Actinobacteria Actinobacteridae    | >gi|296169954|ref|ZP_06851561.1| hly-III family protein [Mycobacterium parascrofulaceum ATCC BAA-614]
296394379 23..227    |Segniliparus rotundus DSM 44985               |B|Actinobacteria Actinobacteridae    | >gi|296394379|ref|YP_003659263.1| channel protein, hemolysin III family [Segniliparus rotundus DSM 44985]
42527819  11..215    |Treponema denticola ATCC 35405                |B|Spirochaetes Spirochaetales        | >gi|42527819|ref|NP_972917.1| hemolysin III [Treponema denticola ATCC 35405]
71996356  377..581   |Caenorhabditis elegans                        |E|Metazoa Nematoda                   | >gi|71996356|ref|NP_001021821.1| hypothetical protein Y71G12B.23 [Caenorhabditis elegans]
225719929 36..239    |Caenorhabditis elegans                        |E|Metazoa Nematoda                   | >gi|225719929|gb|ACO15793.1| Hypothetical protein Y71G12B.23a [Caenorhabditis elegans]
139439359 21..224    |Collinsella aerofaciens ATCC 25986            |B|Actinobacteria Coriobacteridae     | >gi|139439359|ref|ZP_01772800.1| Hypothetical protein COLAER_01819 [Collinsella aerofaciens ATCC 25986]
145592662 18..221    |Salinispora tropica CNB-440                   |B|Actinobacteria Actinobacteridae    | >gi|145592662|ref|YP_001156959.1| hemolysin III family channel protein [Salinispora tropica CNB-440]
150004214 6..209     |Bacteroides vulgatus ATCC 8482                |B|Bacteroidetes Bacteroidia          | >gi|150004214|ref|YP_001298958.1| hemolysin III [Bacteroides vulgatus ATCC 8482]
212690795 6..209     |Bacteroides dorei DSM 17855                   |B|Bacteroidetes Bacteroidia          | >gi|212690795|ref|ZP_03298923.1| hypothetical protein BACDOR_00282 [Bacteroides dorei DSM 17855]
254884768 6..209     |Bacteroides sp. 4_3_47FAA                     |B|Bacteroidetes Bacteroidia          | >gi|254884768|ref|ZP_05257478.1| hemolysin III [Bacteroides sp. 4_3_47FAA]
159035766 18..221    |Salinispora arenicola CNS-205                 |B|Actinobacteria Actinobacteridae    | >gi|159035766|ref|YP_001535019.1| hemolysin III family channel protein [Salinispora arenicola CNS-205]
167762771 15..218    |Bacteroides stercoris ATCC 43183              |B|Bacteroidetes Bacteroidia          | >gi|167762771|ref|ZP_02434898.1| hypothetical protein BACSTE_01129 [Bacteroides stercoris ATCC 43183]
169628301 39..242    |Mycobacterium abscessus ATCC 19977            |B|Actinobacteria Actinobacteridae    | >gi|169628301|ref|YP_001701950.1| hypothetical protein MAB_1208c [Mycobacterium abscessus ATCC 19977]
183221845 29..232    |Leptospira biflexa serovar Patoc strain 'Patoc 1 (Paris)'|B|Spirochaetes Spirochaetales        | >gi|183221845|ref|YP_001839841.1| putative hemolysin-III related protein [Leptospira biflexa serovar Patoc strain 'Patoc 1 (Paris)']
183602677 52..255    |Bifidobacterium animalis subsp. lactis HN019  |B|Actinobacteria Actinobacteridae    | >gi|183602677|ref|ZP_02964041.1| hypothetical protein BIFLAC_01561 [Bifidobacterium animalis subsp. lactis HN019]
219682508 75..278    |Bifidobacterium animalis subsp. lactis AD011  |B|Actinobacteria Actinobacteridae    | >gi|219682508|ref|YP_002468891.1| hemolysin III [Bifidobacterium animalis subsp. lactis AD011]
289177778 79..282    |Bifidobacterium animalis subsp. lactis BB-12  |B|Actinobacteria Actinobacteridae    | >gi|289177778|gb|ADC85024.1| Conserved membrane protein (hemolysin III-like protein) [Bifidobacterium animalis subsp. lactis BB-12]
187779748 9..212     |Clostridium sporogenes ATCC 15579             |B|Firmicutes Clostridia              | >gi|187779748|ref|ZP_02996221.1| hypothetical protein CLOSPO_03344 [Clostridium sporogenes ATCC 15579]
19705190  8..211     |Fusobacterium nucleatum subsp. nucleatum ATCC 25586|B|Fusobacteria Fusobacteriales       | >gi|19705190|ref|NP_602685.1| hemolysin III [Fusobacterium nucleatum subsp. nucleatum ATCC 25586]
237743399 8..211     |Fusobacterium sp. 7_1                         |B|Fusobacteria Fusobacteriales       | >gi|237743399|ref|ZP_04573880.1| hemolysin III [Fusobacterium sp. 7_1]
209693679 8..211     |Aliivibrio salmonicida LFI1238                |B|Proteobacteria Gammaproteobacteria | >gi|209693679|ref|YP_002261607.1| hemolysin III [Aliivibrio salmonicida LFI1238]
59713155  8..211     |Vibrio fischeri ES114                         |B|Proteobacteria Gammaproteobacteria | >gi|59713155|ref|YP_205931.1| oxidoreductase, inner membrane subunit [Vibrio fischeri ES114]
218887337 7..210     |Desulfovibrio vulgaris str. 'Miyazaki F'      |B|Proteobacteria Deltaproteobacteria | >gi|218887337|ref|YP_002436658.1| channel protein, hemolysin III family [Desulfovibrio vulgaris str. 'Miyazaki F']
219667247 5..208     |Desulfitobacterium hafniense DCB-2            |B|Firmicutes Clostridia              | >gi|219667247|ref|YP_002457682.1| channel protein, hemolysin III family [Desulfitobacterium hafniense DCB-2]
89896899  31..202    |Desulfitobacterium hafniense Y51              |B|Firmicutes Clostridia              | >gi|89896899|ref|YP_520386.1| hypothetical protein DSY4153 [Desulfitobacterium hafniense Y51]
225077143 3..206     |Neisseria flavescens NRL30031/H210            |B|Proteobacteria Betaproteobacteria  | >gi|225077143|ref|ZP_03720342.1| hypothetical protein NEIFLAOT_02198 [Neisseria flavescens NRL30031/H210]
241759105 3..206     |Neisseria flavescens SK114                    |B|Proteobacteria Betaproteobacteria  | >gi|241759105|ref|ZP_04757215.1| hemolysin-3 [Neisseria flavescens SK114]
261379882 3..206     |Neisseria subflava NJ9703                     |B|Proteobacteria Betaproteobacteria  | >gi|261379882|ref|ZP_05984455.1| hemolysin III [Neisseria subflava NJ9703]
227551181 12..215    |Enterococcus faecium TX1330                   |B|Firmicutes Lactobacillales         | >gi|227551181|ref|ZP_03981230.1| hemolysin III [Enterococcus faecium TX1330]
257898833 12..215    |Enterococcus faecium Com15                    |B|Firmicutes Lactobacillales         | >gi|257898833|ref|ZP_05678486.1| hemolysin III [Enterococcus faecium Com15]
69246747  12..215    |Enterococcus faecium DO                       |B|Firmicutes Lactobacillales         | >gi|69246747|ref|ZP_00604095.1| HylII [Enterococcus faecium DO]
238062009 18..221    |Micromonospora sp. ATCC 39149                 |B|Actinobacteria Actinobacteridae    | >gi|238062009|ref|ZP_04606718.1| hemolysin III family channel protein [Micromonospora sp. ATCC 39149]
238856059 12..215    |Enterococcus faecalis TUSoD Ef11              |B|Firmicutes Lactobacillales         | >gi|238856059|ref|ZP_04646338.1| hemolysin-3 [Enterococcus faecalis TUSoD Ef11]
257419412 12..215    |Enterococcus faecalis T11                     |B|Firmicutes Lactobacillales         | >gi|257419412|ref|ZP_05596406.1| hemolysin III hylII [Enterococcus faecalis T11]
29376239  15..218    |Enterococcus faecalis V583                    |B|Firmicutes Lactobacillales         | >gi|29376239|ref|NP_815393.1| hemolysin III [Enterococcus faecalis V583]
227518876 2..203     |Enterococcus faecalis TX0104                  |B|Firmicutes Lactobacillales         | >gi|227518876|ref|ZP_03948925.1| hemolysin III [Enterococcus faecalis TX0104]
242244009 23..226    |Staphylococcus epidermidis W23144             |B|Firmicutes Bacillales              | >gi|242244009|ref|ZP_04798452.1| hemolysin III [Staphylococcus epidermidis W23144]
27468678  23..226    |Staphylococcus epidermidis ATCC 12228         |B|Firmicutes Bacillales              | >gi|27468678|ref|NP_765315.1| hemolysin III [Staphylococcus epidermidis ATCC 12228]
252117674 8..211     |Prevotella melaninogenica ATCC 25845          |B|Bacteroidetes Bacteroidia          | >gi|252117674|ref|ZP_04832659.1| hemolysin-3 [Prevotella melaninogenica ATCC 25845]
288801843 8..211     |Prevotella melaninogenica D18                 |B|Bacteroidetes Bacteroidia          | >gi|288801843|ref|ZP_06407285.1| hemolysin III [Prevotella melaninogenica D18]
254303353 8..211     |Fusobacterium nucleatum subsp. polymorphum ATCC 10953|B|Fusobacteria Fusobacteriales       | >gi|254303353|ref|ZP_04970711.1| possible hemolysin III [Fusobacterium nucleatum subsp. polymorphum ATCC 10953]
256846445 8..211     |Fusobacterium sp. 3_1_36A2                    |B|Fusobacteria Fusobacteriales       | >gi|256846445|ref|ZP_05551902.1| hemolysin III [Fusobacterium sp. 3_1_36A2]
294784486 8..211     |Fusobacterium sp. 3_1_27                      |B|Fusobacteria Fusobacteriales       | >gi|294784486|ref|ZP_06749775.1| hemolysin III [Fusobacterium sp. 3_1_27]
237741307 8..210     |Fusobacterium sp. 4_1_13                      |B|Fusobacteria Fusobacteriales       | >gi|237741307|ref|ZP_04571788.1| hemolysin III [Fusobacterium sp. 4_1_13]
34763557  1..153     |Fusobacterium nucleatum subsp. vincentii ATCC 49256|B|Fusobacteria Fusobacteriales       | >gi|34763557|ref|ZP_00144493.1| Conserved membrane protein (hemolysin III homolog) [Fusobacterium nucleatum subsp. vincentii ATCC 49256]
256822608 26..229    |Kangiella koreensis DSM 16069                 |B|Proteobacteria Gammaproteobacteria | >gi|256822608|ref|YP_003146571.1| hemolysin III family channel protein [Kangiella koreensis DSM 16069]
257057127 22..225    |Saccharomonospora viridis DSM 43017           |B|Actinobacteria Actinobacteridae    | >gi|257057127|ref|YP_003134959.1| channel protein, hemolysin III family [Saccharomonospora viridis DSM 43017]
257867792 12..215    |Enterococcus casseliflavus EC30               |B|Firmicutes Lactobacillales         | >gi|257867792|ref|ZP_05647445.1| hemolysin III [Enterococcus casseliflavus EC30]
257869667 13..216    |Enterococcus gallinarum EG2                   |B|Firmicutes Lactobacillales         | >gi|257869667|ref|ZP_05649320.1| hemolysin III [Enterococcus gallinarum EG2]
260655819 40..243    |Jonquetella anthropi E3_33 E1                 |B|Synergistetes Synergistia          | >gi|260655819|ref|ZP_05861288.1| hemolysin III [Jonquetella anthropi E3_33 E1]
261879081 8..211     |Prevotella bergensis DSM 17361                |B|Bacteroidetes Bacteroidia          | >gi|261879081|ref|ZP_06005508.1| hemolysin III [Prevotella bergensis DSM 17361]
282882572 8..211     |Peptoniphilus lacrimalis 315-B                |B|Firmicutes Clostridia              | >gi|282882572|ref|ZP_06291191.1| inner membrane protein YqfA [Peptoniphilus lacrimalis 315-B]
294339476 23..226    |Thiomonas sp. 3As                             |B|Proteobacteria Betaproteobacteria  | >gi|294339476|emb|CAZ87835.1| Hemolysin-3 (Hemolysin III) (Hly-III) [Thiomonas sp. 3As]
296135370 35..238    |Thiomonas intermedia K12                      |B|Proteobacteria Betaproteobacteria  | >gi|296135370|ref|YP_003642612.1| channel protein, hemolysin III family [Thiomonas intermedia K12]
294675388 5..208     |Prevotella ruminicola 23                      |B|Bacteroidetes Bacteroidia          | >gi|294675388|ref|YP_003576004.1| hemolysin III [Prevotella ruminicola 23]
296443004 7..210     |Clostridium lentocellum DSM 5427              |B|Firmicutes Clostridia              | >gi|296443004|ref|ZP_06885059.1| channel protein, hemolysin III family [Clostridium lentocellum DSM 5427]
297587167 9..212     |Finegoldia magna ATCC 53516                   |B|Firmicutes Clostridia              | >gi|297587167|ref|ZP_06945812.1| hemolysin III [Finegoldia magna ATCC 53516]
46581696  7..210     |Desulfovibrio vulgaris str. Hildenborough     |B|Proteobacteria Deltaproteobacteria | >gi|46581696|ref|YP_012504.1| hemolysin III [Desulfovibrio vulgaris str. Hildenborough]
78356739  5..208     |Desulfovibrio desulfuricans subsp. desulfuricans str. G20|B|Proteobacteria Deltaproteobacteria | >gi|78356739|ref|YP_388188.1| hemolysin III family channel protein [Desulfovibrio desulfuricans subsp. desulfuricans str. G20]
85059976  11..214    |Sodalis glossinidius str. 'morsitans'         |B|Proteobacteria Gammaproteobacteria | >gi|85059976|ref|YP_455678.1| putative hemolysin [Sodalis glossinidius str. 'morsitans']
104774166 17..219    |Lactobacillus delbrueckii subsp. bulgaricus ATCC 11842|B|Firmicutes Lactobacillales         | >gi|104774166|ref|YP_619146.1| hemolysin-like protein [Lactobacillus delbrueckii subsp. bulgaricus ATCC 11842]
116514259 18..219    |Lactobacillus delbrueckii subsp. bulgaricus ATCC BAA-365|B|Firmicutes Lactobacillales         | >gi|116514259|ref|YP_813165.1| hemolysin III-like protein [Lactobacillus delbrueckii subsp. bulgaricus ATCC BAA-365]
110799044 9..211     |Clostridium perfringens ATCC 13124            |B|Firmicutes Clostridia              | >gi|110799044|ref|YP_696595.1| hemolysin III [Clostridium perfringens ATCC 13124]
168204901 9..211     |Clostridium perfringens E str. JGS1987        |B|Firmicutes Clostridia              | >gi|168204901|ref|ZP_02630906.1| hemolysin III [Clostridium perfringens E str. JGS1987]
168214164 9..211     |Clostridium perfringens CPE str. F4969        |B|Firmicutes Clostridia              | >gi|168214164|ref|ZP_02639789.1| hemolysin III [Clostridium perfringens CPE str. F4969]
169343660 9..211     |Clostridium perfringens C str. JGS1495        |B|Firmicutes Clostridia              | >gi|169343660|ref|ZP_02864659.1| hemolysin III [Clostridium perfringens C str. JGS1495]
18310897  9..211     |Clostridium perfringens str. 13               |B|Firmicutes Clostridia              | >gi|18310897|ref|NP_562831.1| hemolysin III [Clostridium perfringens str. 13]
110802509 12..214    |Clostridium perfringens SM101                 |B|Firmicutes Clostridia              | >gi|110802509|ref|YP_699192.1| hemolysin III [Clostridium perfringens SM101]
110806802 12..214    |Shigella flexneri 5 str. 8401                 |B|Proteobacteria Gammaproteobacteria | >gi|110806802|ref|YP_690322.1| putative oxidoreductase [Shigella flexneri 5 str. 8401]
157148437 12..214    |Citrobacter koseri ATCC BAA-895               |B|Proteobacteria Gammaproteobacteria | >gi|157148437|ref|YP_001455756.1| hypothetical protein CKO_04262 [Citrobacter koseri ATCC BAA-895]
15803435  12..214    |Escherichia coli O157:H7 str. EDL933          |B|Proteobacteria Gammaproteobacteria | >gi|15803435|ref|NP_289468.1| putative oxidoreductase [Escherichia coli O157:H7 str. EDL933]
170682502 12..214    |Escherichia coli SMS-3-5                      |B|Proteobacteria Gammaproteobacteria | >gi|170682502|ref|YP_001745052.1| hemolysin III family channel protein [Escherichia coli SMS-3-5]
218550147 12..214    |Escherichia fergusonii ATCC 35469             |B|Proteobacteria Gammaproteobacteria | >gi|218550147|ref|YP_002383938.1| hemolysin, inner membrane subunit [Escherichia fergusonii ATCC 35469]
256024590 12..214    |Escherichia sp. 4_1_40B                       |B|Proteobacteria Gammaproteobacteria | >gi|256024590|ref|ZP_05438455.1| predicted oxidoreductase, inner membrane subunit [Escherichia sp. 4_1_40B]
281179904 12..214    |Escherichia coli SE15                         |B|Proteobacteria Gammaproteobacteria | >gi|281179904|dbj|BAI56234.1| conserved hypothetical protein [Escherichia coli SE15]
82545478  12..214    |Shigella boydii Sb227                         |B|Proteobacteria Gammaproteobacteria | >gi|82545478|ref|YP_409425.1| oxidoreductase [Shigella boydii Sb227]
82778335  25..219    |Shigella dysenteriae Sd197                    |B|Proteobacteria Gammaproteobacteria | >gi|82778335|ref|YP_404684.1| putative oxidoreductase [Shigella dysenteriae Sd197]
297517676 12..172    |Escherichia coli OP50                         |B|Proteobacteria Gammaproteobacteria | >gi|297517676|ref|ZP_06936062.1| putative oxidoreductase [Escherichia coli OP50]
110835495 12..214    |Alcanivorax borkumensis SK2                   |B|Proteobacteria Gammaproteobacteria | >gi|110835495|ref|YP_694354.1| hemolysin III-like protein [Alcanivorax borkumensis SK2]
254428976 12..214    |Alcanivorax sp. DG881                         |B|Proteobacteria Gammaproteobacteria | >gi|254428976|ref|ZP_05042683.1| channel protein, hemolysin III family [Alcanivorax sp. DG881]
114775368 16..218    |Mariprofundus ferrooxydans PV-1               |B|Proteobacteria Zetaproteobacteria  | >gi|114775368|ref|ZP_01450936.1| channel protein, hemolysin III family [Mariprofundus ferrooxydans PV-1]
116333433 10..212    |Lactobacillus brevis ATCC 367                 |B|Firmicutes Lactobacillales         | >gi|116333433|ref|YP_794960.1| hemolysin III-like protein [Lactobacillus brevis ATCC 367]
116491364 13..215    |Oenococcus oeni PSU-1                         |B|Firmicutes Lactobacillales         | >gi|116491364|ref|YP_810908.1| hemolysin III-like protein [Oenococcus oeni PSU-1]
118586610 13..215    |Oenococcus oeni ATCC BAA-1163                 |B|Firmicutes Lactobacillales         | >gi|118586610|ref|ZP_01544050.1| hemolysin like protein [Oenococcus oeni ATCC BAA-1163]
116511323 12..214    |Lactococcus lactis subsp. cremoris SK11       |B|Firmicutes Lactobacillales         | >gi|116511323|ref|YP_808539.1| hemolysin like protein [Lactococcus lactis subsp. cremoris SK11]
125623358 12..214    |Lactococcus lactis subsp. cremoris MG1363     |B|Firmicutes Lactobacillales         | >gi|125623358|ref|YP_001031841.1| hemolysin like protein [Lactococcus lactis subsp. cremoris MG1363]
15672479  12..214    |Lactococcus lactis subsp. lactis Il1403       |B|Firmicutes Lactobacillales         | >gi|15672479|ref|NP_266653.1| hemolysin like protein [Lactococcus lactis subsp. lactis Il1403]
281490991 12..214    |Lactococcus lactis subsp. lactis KF147        |B|Firmicutes Lactobacillales         | >gi|281490991|ref|YP_003352971.1| hypothetical protein LLKF_0501 [Lactococcus lactis subsp. lactis KF147]
134024490 56..258    |Xenopus (Silurana) tropicalis                 |E|Metazoa Chordata                   | >gi|134024490|gb|AAI36009.1| mmd protein [Xenopus (Silurana) tropicalis]
145296582 48..250    |Corynebacterium glutamicum R                  |B|Actinobacteria Actinobacteridae    | >gi|145296582|ref|YP_001139403.1| hypothetical protein cgR_2490 [Corynebacterium glutamicum R]
19553783  48..250    |Corynebacterium glutamicum ATCC 13032         |B|Actinobacteria Actinobacteridae    | >gi|19553783|ref|NP_601785.1| membrane proteins [Corynebacterium glutamicum ATCC 13032]
21325359  41..243    |Corynebacterium glutamicum ATCC 13032         |B|Actinobacteria Actinobacteridae    | >gi|21325359|dbj|BAB99980.1| Predicted membrane proteins, hemolysin III homologs [Corynebacterium glutamicum ATCC 13032]
57157718  48..250    |Corynebacterium glutamicum                    |B|Actinobacteria Actinobacteridae    | >gi|57157718|dbj|BAD83825.1| hypothetical protein [Corynebacterium glutamicum]
146312957 11..213    |Enterobacter sp. 638                          |B|Proteobacteria Gammaproteobacteria | >gi|146312957|ref|YP_001178031.1| hemolysin III family channel protein [Enterobacter sp. 638]
148978302 10..212    |Vibrionales bacterium SWAT-3                  |B|Proteobacteria Gammaproteobacteria | >gi|148978302|ref|ZP_01814807.1| putative hemolysin III [Vibrionales bacterium SWAT-3]
84393435  10..212    |Vibrio splendidus 12B01                       |B|Proteobacteria Gammaproteobacteria | >gi|84393435|ref|ZP_00992192.1| putative hemolysin III [Vibrio splendidus 12B01]
86147135  10..212    |Vibrio sp. MED222                             |B|Proteobacteria Gammaproteobacteria | >gi|86147135|ref|ZP_01065451.1| putative hemolysin III [Vibrio sp. MED222]
152971845 12..214    |Klebsiella pneumoniae subsp. pneumoniae MGH 78578|B|Proteobacteria Gammaproteobacteria | >gi|152971845|ref|YP_001336954.1| putative oxidoreductase [Klebsiella pneumoniae subsp. pneumoniae MGH 78578]
170766013 12..214    |Escherichia albertii TW07627                  |B|Proteobacteria Gammaproteobacteria | >gi|170766013|ref|ZP_02900824.1| channel protein, hemolysin III family [Escherichia albertii TW07627]
206581111 12..214    |Klebsiella pneumoniae 342                     |B|Proteobacteria Gammaproteobacteria | >gi|206581111|ref|YP_002236644.1| channel protein, hemolysin III family [Klebsiella pneumoniae 342]
261342302 12..214    |Enterobacter cancerogenus ATCC 35316          |B|Proteobacteria Gammaproteobacteria | >gi|261342302|ref|ZP_05970160.1| hemolysin III [Enterobacter cancerogenus ATCC 35316]
152997331 14..216    |Marinomonas sp. MWYL1                         |B|Proteobacteria Gammaproteobacteria | >gi|152997331|ref|YP_001342166.1| hemolysin III family channel protein [Marinomonas sp. MWYL1]
153834307 26..228    |Vibrio harveyi HY01                           |B|Proteobacteria Gammaproteobacteria | >gi|153834307|ref|ZP_01986974.1| hemolysin-3 [Vibrio harveyi HY01]
156972732 26..228    |Vibrio harveyi ATCC BAA-1116                  |B|Proteobacteria Gammaproteobacteria | >gi|156972732|ref|YP_001443639.1| hypothetical protein VIBHAR_00397 [Vibrio harveyi ATCC BAA-1116]
163803322 10..212    |Vibrio sp. AND4                               |B|Proteobacteria Gammaproteobacteria | >gi|163803322|ref|ZP_02197200.1| putative hemolysin III [Vibrio sp. AND4]
269962650 26..228    |Vibrio harveyi 1DA3                           |B|Proteobacteria Gammaproteobacteria | >gi|269962650|ref|ZP_06176995.1| hemolysin, putative [Vibrio harveyi 1DA3]
153837694 10..212    |Vibrio parahaemolyticus AQ3810                |B|Proteobacteria Gammaproteobacteria | >gi|153837694|ref|ZP_01990361.1| hemolysin-3 [Vibrio parahaemolyticus AQ3810]
28899822  11..213    |Vibrio parahaemolyticus RIMD 2210633          |B|Proteobacteria Gammaproteobacteria | >gi|28899822|ref|NP_799427.1| putative hemolysin III [Vibrio parahaemolyticus RIMD 2210633]
153955737 9..211     |Clostridium kluyveri DSM 555                  |B|Firmicutes Clostridia              | >gi|153955737|ref|YP_001396502.1| hemolysin III-related protein [Clostridium kluyveri DSM 555]
154686426 3..205     |Bacillus amyloliquefaciens FZB42              |B|Firmicutes Bacillales              | >gi|154686426|ref|YP_001421587.1| YplQ [Bacillus amyloliquefaciens FZB42]
15615428  8..210     |Bacillus halodurans C-125                     |B|Firmicutes Bacillales              | >gi|15615428|ref|NP_243731.1| hemolysin III [Bacillus halodurans C-125]
156375435 23..225    |Nematostella vectensis                        |E|Metazoa Cnidaria                   | >gi|156375435|ref|XP_001630086.1| predicted protein [Nematostella vectensis]
156932649 12..214    |Cronobacter sakazakii ATCC BAA-894            |B|Proteobacteria Gammaproteobacteria | >gi|156932649|ref|YP_001436565.1| hypothetical protein ESA_00432 [Cronobacter sakazakii ATCC BAA-894]
260599239 12..214    |Cronobacter turicensis z3032                  |B|Proteobacteria Gammaproteobacteria | >gi|260599239|ref|YP_003211810.1| hemolysin [Cronobacter turicensis z3032]
157692681 3..205     |Bacillus pumilus SAFR-032                     |B|Firmicutes Bacillales              | >gi|157692681|ref|YP_001487143.1| hemolysin III [Bacillus pumilus SAFR-032]
194016843 3..205     |Bacillus pumilus ATCC 7061                    |B|Firmicutes Bacillales              | >gi|194016843|ref|ZP_03055456.1| hemolysin-3 (Hemolysin III) (Hly-III) [Bacillus pumilus ATCC 7061]
15925160  24..226    |Staphylococcus aureus subsp. aureus Mu50      |B|Firmicutes Bacillales              | >gi|15925160|ref|NP_372694.1| hemolysin III [Staphylococcus aureus subsp. aureus Mu50]
21283825  23..225    |Staphylococcus aureus subsp. aureus MW2       |B|Firmicutes Bacillales              | >gi|21283825|ref|NP_646913.1| hypothetical protein MW2096 [Staphylococcus aureus subsp. aureus MW2]
258423080 24..226    |Staphylococcus aureus A9635                   |B|Firmicutes Bacillales              | >gi|258423080|ref|ZP_05685978.1| hemolysin [Staphylococcus aureus A9635]
283471399 24..226    |Staphylococcus aureus subsp. aureus ST398     |B|Firmicutes Bacillales              | >gi|283471399|emb|CAQ50610.1| hemolysin III [Staphylococcus aureus subsp. aureus ST398]
49484390  23..225    |Staphylococcus aureus subsp. aureus MRSA252   |B|Firmicutes Bacillales              | >gi|49484390|ref|YP_041614.1| hypothetical protein SAR2261 [Staphylococcus aureus subsp. aureus MRSA252]
82751768  24..226    |Staphylococcus aureus RF122                   |B|Firmicutes Bacillales              | >gi|82751768|ref|YP_417509.1| hemolysin [Staphylococcus aureus RF122]
16079238  3..205     |Bacillus subtilis subsp. subtilis str. 168    |B|Firmicutes Bacillales              | >gi|16079238|ref|NP_390062.1| membrane hydrolase [Bacillus subtilis subsp. subtilis str. 168]
291484598 3..205     |Bacillus subtilis subsp. natto BEST195        |B|Firmicutes Bacillales              | >gi|291484598|dbj|BAI85673.1| hypothetical protein BSNT_03249 [Bacillus subtilis subsp. natto BEST195]
296329485 3..205     |Bacillus subtilis subsp. spizizenii ATCC 6633 |B|Firmicutes Bacillales              | >gi|296329485|ref|ZP_06871972.1| putative membrane hydrolase [Bacillus subtilis subsp. spizizenii ATCC 6633]
160889784 11..213    |Bacteroides uniformis ATCC 8492               |B|Bacteroidetes Bacteroidia          | >gi|160889784|ref|ZP_02070787.1| hypothetical protein BACUNI_02215 [Bacteroides uniformis ATCC 8492]
270294061 11..213    |Bacteroides sp. D20                           |B|Bacteroidetes Bacteroidia          | >gi|270294061|ref|ZP_06200263.1| conserved hypothetical protein [Bacteroides sp. D20]
161506403 12..214    |Salmonella enterica subsp. arizonae serovar 62:z4,z23:-- str.|B|Proteobacteria Gammaproteobacteria | >gi|161506403|ref|YP_001573515.1| hypothetical protein SARI_04601 [Salmonella enterica subsp. arizonae serovar 62:z4,z23:-- str. RSK2980]
16761830  12..214    |Salmonella enterica subsp. enterica serovar Typhi str. CT18|B|Proteobacteria Gammaproteobacteria | >gi|16761830|ref|NP_457447.1| hypothetical protein STY3205 [Salmonella enterica subsp. enterica serovar Typhi str. CT18]
168242837 12..214    |Salmonella enterica subsp. enterica serovar Heidelberg str. SL486|B|Proteobacteria Gammaproteobacteria | >gi|168242837|ref|ZP_02667769.1| hemolysin-3 [Salmonella enterica subsp. enterica serovar Heidelberg str. SL486]
168261788 12..214    |Salmonella enterica subsp. enterica serovar Hadar str. RI_05P066|B|Proteobacteria Gammaproteobacteria | >gi|168261788|ref|ZP_02683761.1| hemolysin-3 [Salmonella enterica subsp. enterica serovar Hadar str. RI_05P066]
194468471 12..214    |Salmonella enterica subsp. enterica serovar Kentucky str. CVM29188|B|Proteobacteria Gammaproteobacteria | >gi|194468471|ref|ZP_03074455.1| hemolysin-3 [Salmonella enterica subsp. enterica serovar Kentucky str. CVM29188]
204928392 12..214    |Salmonella enterica subsp. enterica serovar Javiana str.|B|Proteobacteria Gammaproteobacteria | >gi|204928392|ref|ZP_03219592.1| hemolysin-3 [Salmonella enterica subsp. enterica serovar Javiana str. GA_MM04042433]
283788443 12..214    |Citrobacter rodentium ICC168                  |B|Proteobacteria Gammaproteobacteria | >gi|283788443|ref|YP_003368308.1| hypothetical protein ROD_49341 [Citrobacter rodentium ICC168]
213857741 1..182     |Salmonella enterica subsp. enterica serovar Typhi str. M223|B|Proteobacteria Gammaproteobacteria | >gi|213857741|ref|ZP_03384712.1| hypothetical protein SentesT_21680 [Salmonella enterica subsp. enterica serovar Typhi str. M223]
213029484 1..137     |Salmonella enterica subsp. enterica serovar Typhi str. 404ty|B|Proteobacteria Gammaproteobacteria | >gi|213029484|ref|ZP_03343931.1| hypothetical protein Salmonelentericaenterica_47867 [Salmonella enterica subsp. enterica serovar Typhi str. 404ty]
163857542 9..211     |Bordetella petrii DSM 12804                   |B|Proteobacteria Betaproteobacteria  | >gi|163857542|ref|YP_001631840.1| hemolysin III [Bordetella petrii DSM 12804]
167461576 4..206     |Paenibacillus larvae subsp. larvae BRL-230010 |B|Firmicutes Bacillales              | >gi|167461576|ref|ZP_02326665.1| channel protein, hemolysin III family [Paenibacillus larvae subsp. larvae BRL-230010]
167461598 4..206     |Paenibacillus larvae subsp. larvae BRL-230010 |B|Firmicutes Bacillales              | >gi|167461598|ref|ZP_02326687.1| channel protein, hemolysin III family [Paenibacillus larvae subsp. larvae BRL-230010]
167756724 44..246    |Clostridium ramosum DSM 1402                  |B|Firmicutes Erysipelotrichi         | >gi|167756724|ref|ZP_02428851.1| hypothetical protein CLORAM_02265 [Clostridium ramosum DSM 1402]
237734443 25..227    |Coprobacillus sp. D7                          |B|Firmicutes Erysipelotrichi         | >gi|237734443|ref|ZP_04564924.1| hemolysin III [Mollicutes bacterium D7]
170023122 30..232    |Yersinia pseudotuberculosis YPIII             |B|Proteobacteria Gammaproteobacteria | >gi|170023122|ref|YP_001719627.1| hemolysin III family channel protein [Yersinia pseudotuberculosis YPIII]
186896607 30..232    |Yersinia pseudotuberculosis PB1/+             |B|Proteobacteria Gammaproteobacteria | >gi|186896607|ref|YP_001873719.1| hemolysin III family channel protein [Yersinia pseudotuberculosis PB1/+]
22127161  30..232    |Yersinia pestis KIM 10                        |B|Proteobacteria Gammaproteobacteria | >gi|22127161|ref|NP_670584.1| oxidoreductase [Yersinia pestis KIM 10]
238752282 12..214    |Yersinia rohdei ATCC 43380                    |B|Proteobacteria Gammaproteobacteria | >gi|238752282|ref|ZP_04613761.1| hypothetical protein yrohd0001_18720 [Yersinia rohdei ATCC 43380]
238763223 22..224    |Yersinia kristensenii ATCC 33638              |B|Proteobacteria Gammaproteobacteria | >gi|238763223|ref|ZP_04624188.1| hypothetical protein ykris0001_27990 [Yersinia kristensenii ATCC 33638]
238786188 12..214    |Yersinia bercovieri ATCC 43970                |B|Proteobacteria Gammaproteobacteria | >gi|238786188|ref|ZP_04630138.1| hypothetical protein yberc0001_38440 [Yersinia bercovieri ATCC 43970]
170743463 21..223    |Methylobacterium sp. 4-46                     |B|Proteobacteria Alphaproteobacteria | >gi|170743463|ref|YP_001772118.1| Hly-III family protein [Methylobacterium sp. 4-46]
172058365 28..230    |Exiguobacterium sibiricum 255-15              |B|Firmicutes Bacillales              | >gi|172058365|ref|YP_001814825.1| hemolysin III family channel protein [Exiguobacterium sibiricum 255-15]
188534913 12..214    |Erwinia tasmaniensis Et1/99                   |B|Proteobacteria Gammaproteobacteria | >gi|188534913|ref|YP_001908710.1| hypothetical protein ETA_27930 [Erwinia tasmaniensis Et1/99]
189440217 78..280    |Bifidobacterium longum DJO10A                 |B|Actinobacteria Actinobacteridae    | >gi|189440217|ref|YP_001955298.1| putative hemolysin III-like membrane protein [Bifidobacterium longum DJO10A]
227547411 66..268    |Bifidobacterium longum subsp. longum ATCC 55813|B|Actinobacteria Actinobacteridae    | >gi|227547411|ref|ZP_03977460.1| hemolysin III family membrane protein [Bifidobacterium longum subsp. infantis ATCC 55813]
23465145  20..222    |Bifidobacterium longum NCC2705                |B|Actinobacteria Actinobacteridae    | >gi|23465145|ref|NP_695748.1| hypothetical protein BL0560 [Bifidobacterium longum NCC2705]
291517669 66..268    |Bifidobacterium longum subsp. longum F8       |B|Actinobacteria Actinobacteridae    | >gi|291517669|emb|CBK71285.1| Predicted membrane protein, hemolysin III homolog [Bifidobacterium longum subsp. longum F8]
296453260 78..280    |Bifidobacterium longum subsp. longum JDM301   |B|Actinobacteria Actinobacteridae    | >gi|296453260|ref|YP_003660403.1| Hly-III family protein [Bifidobacterium longum subsp. longum JDM301]
46190522  66..268    |Bifidobacterium longum DJO10A                 |B|Actinobacteria Actinobacteridae    | >gi|46190522|ref|ZP_00121423.2| COG1272: Predicted membrane protein, hemolysin III homolog [Bifidobacterium longum DJO10A]
189466166 15..217    |Bacteroides intestinalis DSM 17393            |B|Bacteroidetes Bacteroidia          | >gi|189466166|ref|ZP_03014951.1| hypothetical protein BACINT_02536 [Bacteroides intestinalis DSM 17393]
196229794 16..218    |Chthoniobacter flavus Ellin428                |B|Verrucomicrobia Spartobacteria     | >gi|196229794|ref|ZP_03128658.1| Hly-III family protein [Chthoniobacter flavus Ellin428]
213691016 73..275    |Bifidobacterium longum subsp. infantis ATCC 15697|B|Actinobacteria Actinobacteridae    | >gi|213691016|ref|YP_002321602.1| Hly-III family protein [Bifidobacterium longum subsp. infantis ATCC 15697]
291455773 66..268    |Bifidobacterium breve DSM 20213               |B|Actinobacteria Actinobacteridae    | >gi|291455773|ref|ZP_06595163.1| membrane protein, hemolysin III-like protein [Bifidobacterium breve DSM 20213]
222153080 12..214    |Streptococcus uberis 0140J                    |B|Firmicutes Lactobacillales         | >gi|222153080|ref|YP_002562257.1| haemolysin-III related membrane protein [Streptococcus uberis 0140J]
223042416 23..225    |Staphylococcus capitis SK14                   |B|Firmicutes Bacillales              | >gi|223042416|ref|ZP_03612465.1| hemolysin III [Staphylococcus capitis SK14]
224075317 98..300    |Taeniopygia guttata                           |E|Metazoa Chordata                   | >gi|224075317|ref|XP_002192071.1| PREDICTED: monocyte to macrophage differentiation-associated [Taeniopygia guttata]
224283537 67..269    |Bifidobacterium bifidum NCIMB 41171           |B|Actinobacteria Actinobacteridae    | >gi|224283537|ref|ZP_03646859.1| Putative hemolysin III-like membrane protein [Bifidobacterium bifidum NCIMB 41171]
224477155 23..225    |Staphylococcus carnosus subsp. carnosus TM300 |B|Firmicutes Bacillales              | >gi|224477155|ref|YP_002634761.1| putative hemolysin III [Staphylococcus carnosus subsp. carnosus TM300]
224539422 15..217    |Bacteroides cellulosilyticus DSM 14838        |B|Bacteroidetes Bacteroidia          | >gi|224539422|ref|ZP_03679961.1| hypothetical protein BACCELL_04327 [Bacteroides cellulosilyticus DSM 14838]
225011496 10..212    |Flavobacteria bacterium MS024-2A              |B|Bacteroidetes Flavobacteria.       | >gi|225011496|ref|ZP_03701934.1| channel protein, hemolysin III family [Flavobacteria bacterium MS024-2A]
225018555 15..217    |Clostridium methylpentosum DSM 5476           |B|Firmicutes Clostridia              | >gi|225018555|ref|ZP_03707747.1| hypothetical protein CLOSTMETH_02504 [Clostridium methylpentosum DSM 5476]
225156279 318..520   |Opitutaceae bacterium TAV2                    |B|Verrucomicrobia Opitutae           | >gi|225156279|ref|ZP_03724757.1| channel protein, hemolysin III family [Opitutaceae bacterium TAV2]
225174304 8..210     |Dethiobacter alkaliphilus AHT 1               |B|Firmicutes Clostridia              | >gi|225174304|ref|ZP_03728303.1| channel protein, hemolysin III family [Dethiobacter alkaliphilus AHT 1]
225620779 17..219    |Brachyspira hyodysenteriae WA1                |B|Spirochaetes Spirochaetales        | >gi|225620779|ref|YP_002722037.1| putative channel protein, hemolysin III family protein [Brachyspira hyodysenteriae WA1]
227112624 11..213    |Pectobacterium carotovorum subsp. brasiliensis PBR1692|B|Proteobacteria Gammaproteobacteria | >gi|227112624|ref|ZP_03826280.1| hypothetical protein PcarbP_06657 [Pectobacterium carotovorum subsp. brasiliensis PBR1692]
227328388 11..213    |Pectobacterium carotovorum subsp. carotovorum WPP14|B|Proteobacteria Gammaproteobacteria | >gi|227328388|ref|ZP_03832412.1| hypothetical protein PcarcW_14072 [Pectobacterium carotovorum subsp. carotovorum WPP14]
253687037 11..213    |Pectobacterium carotovorum subsp. carotovorum PC1|B|Proteobacteria Gammaproteobacteria | >gi|253687037|ref|YP_003016227.1| channel protein, hemolysin III family [Pectobacterium carotovorum subsp. carotovorum PC1]
261820170 11..213    |Pectobacterium wasabiae WPP163                |B|Proteobacteria Gammaproteobacteria | >gi|261820170|ref|YP_003258276.1| channel protein, hemolysin III family [Pectobacterium wasabiae WPP163]
50119702  11..213    |Pectobacterium atrosepticum SCRI1043          |B|Proteobacteria Gammaproteobacteria | >gi|50119702|ref|YP_048869.1| hypothetical protein ECA0757 [Pectobacterium atrosepticum SCRI1043]
227497805 40..242    |Actinomyces urogenitalis DSM 15434            |B|Actinobacteria Actinobacteridae    | >gi|227497805|ref|ZP_03927990.1| Hly-III family protein [Actinomyces urogenitalis DSM 15434]
227548109 26..228    |Corynebacterium lipophiloflavum DSM 44291     |B|Actinobacteria Actinobacteridae    | >gi|227548109|ref|ZP_03978158.1| Hly III family protein [Corynebacterium lipophiloflavum DSM 44291]
227833995 63..265    |Corynebacterium aurimucosum ATCC 700975       |B|Actinobacteria Actinobacteridae    | >gi|227833995|ref|YP_002835702.1| transporter of the Hly III family [Corynebacterium aurimucosum ATCC 700975]
262183515 27..229    |Corynebacterium aurimucosum ATCC 700975       |B|Actinobacteria Actinobacteridae    | >gi|262183515|ref|ZP_06042936.1| transporter of the Hly III family protein [Corynebacterium aurimucosum ATCC 700975]
227879119 18..220    |Lactobacillus crispatus JV-V01                |B|Firmicutes Lactobacillales         | >gi|227879119|ref|ZP_03997003.1| hemolysin III [Lactobacillus crispatus JV-V01]
227893498 18..220    |Lactobacillus ultunensis DSM 16047            |B|Firmicutes Lactobacillales         | >gi|227893498|ref|ZP_04011303.1| hemolysin III [Lactobacillus ultunensis DSM 16047]
161507465 27..228    |Lactobacillus helveticus DPC 4571             |B|Firmicutes Lactobacillales         | >gi|161507465|ref|YP_001577419.1| hemolysin III [Lactobacillus helveticus DPC 4571]
260101623 27..228    |Lactobacillus helveticus DSM 20075            |B|Firmicutes Lactobacillales         | >gi|260101623|ref|ZP_05751860.1| hemolysin III [Lactobacillus helveticus DSM 20075]
228474750 23..225    |Staphylococcus hominis SK119                  |B|Firmicutes Bacillales              | >gi|228474750|ref|ZP_04059481.1| hemolysin III [Staphylococcus hominis SK119]
229544324 9..211     |Bacillus coagulans 36D1                       |B|Firmicutes Bacillales              | >gi|229544324|ref|ZP_04433383.1| channel protein, hemolysin III family [Bacillus coagulans 36D1]
229916552 14..216    |Exiguobacterium sp. AT1b                      |B|Firmicutes Bacillales              | >gi|229916552|ref|YP_002885198.1| channel protein, hemolysin III family [Exiguobacterium sp. AT1b]
237729840 12..214    |Citrobacter sp. 30_2                          |B|Proteobacteria Gammaproteobacteria | >gi|237729840|ref|ZP_04560321.1| hemolysin III family channel protein [Citrobacter sp. 30_2]
283835341 12..214    |Citrobacter youngae ATCC 29220                |B|Proteobacteria Gammaproteobacteria | >gi|283835341|ref|ZP_06355082.1| hemolysin III [Citrobacter youngae ATCC 29220]
296104561 12..214    |Enterobacter cloacae subsp. cloacae ATCC 13047|B|Proteobacteria Gammaproteobacteria | >gi|296104561|ref|YP_003614707.1| hemolysin III [Enterobacter cloacae subsp. cloacae ATCC 13047]
237738543 8..210     |Fusobacterium sp. 2_1_31                      |B|Fusobacteria Fusobacteriales       | >gi|237738543|ref|ZP_04569024.1| hemolysin III [Fusobacterium sp. 2_1_31]
262065887 8..210     |Fusobacterium periodonticum ATCC 33693        |B|Fusobacteria Fusobacteriales       | >gi|262065887|ref|ZP_06025499.1| hemolysin III [Fusobacterium periodonticum ATCC 33693]
294783896 8..210     |Fusobacterium sp. 1_1_41FAA                   |B|Fusobacteria Fusobacteriales       | >gi|294783896|ref|ZP_06749218.1| hemolysin III [Fusobacterium sp. 1_1_41FAA]
238754569 18..220    |Yersinia ruckeri ATCC 29473                   |B|Proteobacteria Gammaproteobacteria | >gi|238754569|ref|ZP_04615923.1| hypothetical protein yruck0001_4370 [Yersinia ruckeri ATCC 29473]
238787328 24..226    |Yersinia frederiksenii ATCC 33641             |B|Proteobacteria Gammaproteobacteria | >gi|238787328|ref|ZP_04631127.1| hypothetical protein yfred0001_33160 [Yersinia frederiksenii ATCC 33641]
238796534 24..226    |Yersinia mollaretii ATCC 43969                |B|Proteobacteria Gammaproteobacteria | >gi|238796534|ref|ZP_04640041.1| hypothetical protein ymoll0001_29990 [Yersinia mollaretii ATCC 43969]
238759290 19..220    |Yersinia aldovae ATCC 35236                   |B|Proteobacteria Gammaproteobacteria | >gi|238759290|ref|ZP_04620456.1| hypothetical protein yaldo0001_5170 [Yersinia aldovae ATCC 35236]
238795223 13..214    |Yersinia intermedia ATCC 29909                |B|Proteobacteria Gammaproteobacteria | >gi|238795223|ref|ZP_04638808.1| hypothetical protein yinte0001_4650 [Yersinia intermedia ATCC 29909]
51597487  30..231    |Yersinia pseudotuberculosis IP 32953          |B|Proteobacteria Gammaproteobacteria | >gi|51597487|ref|YP_071678.1| hemolysin III. [Yersinia pseudotuberculosis IP 32953]
123443584 1..188     |Yersinia enterocolitica subsp. enterocolitica 8081|B|Proteobacteria Gammaproteobacteria | >gi|123443584|ref|YP_001007557.1| putative hemolysin III [Yersinia enterocolitica subsp. enterocolitica 8081]
238921207 13..215    |Edwardsiella ictaluri 93-146                  |B|Proteobacteria Gammaproteobacteria | >gi|238921207|ref|YP_002934722.1| channel forming cytolysin, [Edwardsiella ictaluri 93-146]
269140280 13..215    |Edwardsiella tarda EIB202                     |B|Proteobacteria Gammaproteobacteria | >gi|269140280|ref|YP_003296981.1| channel protein, hemolysin III family [Edwardsiella tarda EIB202]
239636167 22..224    |Staphylococcus warneri L37603                 |B|Firmicutes Bacillales              | >gi|239636167|ref|ZP_04677171.1| hemolysin III [Staphylococcus warneri L37603]
242240710 11..213    |Dickeya dadantii Ech703                       |B|Proteobacteria Gammaproteobacteria | >gi|242240710|ref|YP_002988891.1| hemolysin III family channel protein [Dickeya dadantii Ech703]
242371835 23..225    |Staphylococcus epidermidis M23864:W1          |B|Firmicutes Bacillales              | >gi|242371835|ref|ZP_04817409.1| hemolysin III [Staphylococcus epidermidis M23864:W1]
251782426 12..214    |Streptococcus dysgalactiae subsp. equisimilis GGS_124|B|Firmicutes Lactobacillales         | >gi|251782426|ref|YP_002996728.1| hypothetical protein SDEG_1015 [Streptococcus dysgalactiae subsp. equisimilis GGS_124]
251791002 11..213    |Dickeya zeae Ech1591                          |B|Proteobacteria Gammaproteobacteria | >gi|251791002|ref|YP_003005723.1| channel protein, hemolysin III family [Dickeya zeae Ech1591]
251798505 8..210     |Paenibacillus sp. JDR-2                       |B|Firmicutes Bacillales              | >gi|251798505|ref|YP_003013236.1| channel protein, hemolysin III family [Paenibacillus sp. JDR-2]
254229993 10..212    |Vibrio sp. Ex25                               |B|Proteobacteria Gammaproteobacteria | >gi|254229993|ref|ZP_04923394.1| channel protein, hemolysin III family [Vibrio sp. Ex25]
269967005 10..212    |Vibrio alginolyticus 40B                      |B|Proteobacteria Gammaproteobacteria | >gi|269967005|ref|ZP_06181075.1| hemolysin, putative [Vibrio alginolyticus 40B]
91226296  10..212    |Vibrio alginolyticus 12G01                    |B|Proteobacteria Gammaproteobacteria | >gi|91226296|ref|ZP_01261136.1| putative hemolysin III [Vibrio alginolyticus 12G01]
254507324 10..212    |Vibrio parahaemolyticus 16                    |B|Proteobacteria Gammaproteobacteria | >gi|254507324|ref|ZP_05119460.1| hemolysin [Vibrio parahaemolyticus 16]
254520253 14..216    |Clostridium sp. 7_2_43FAA                     |B|Firmicutes Clostridia              | >gi|254520253|ref|ZP_05132309.1| hemolysin III [Clostridium sp. 7_2_43FAA]
255058703 14..216    |Geobacter sp. M18                             |B|Proteobacteria Deltaproteobacteria | >gi|255058703|ref|ZP_05310867.1| channel protein, hemolysin III family [Geobacter sp. M18]
256672081 1..203     |Streptomyces sp. AA4                          |B|Actinobacteria Actinobacteridae    | >gi|256672081|ref|ZP_05483034.1| hemolysin III family channel protein [Streptomyces sp. AA4]
257790021 26..228    |Eggerthella lenta DSM 2243                    |B|Actinobacteria Coriobacteridae     | >gi|257790021|ref|YP_003180627.1| channel protein, hemolysin III family [Eggerthella lenta DSM 2243]
258638716 12..214    |Pantoea sp. At-9b                             |B|Proteobacteria Gammaproteobacteria | >gi|258638716|ref|ZP_05731452.1| channel protein, hemolysin III family [Pantoea sp. At-9b]
259504335 14..216    |Erysipelothrix rhusiopathiae ATCC 19414       |B|Firmicutes Erysipelotrichi         | >gi|259504335|ref|ZP_05747237.1| hemolysin [Erysipelothrix rhusiopathiae ATCC 19414]
259909561 12..214    |Erwinia pyrifoliae Ep1/96                     |B|Proteobacteria Gammaproteobacteria | >gi|259909561|ref|YP_002649917.1| hemolysin [Erwinia pyrifoliae Ep1/96]
283479639 16..218    |Erwinia pyrifoliae DSM 12163                  |B|Proteobacteria Gammaproteobacteria | >gi|283479639|emb|CAY75555.1| putative channel protein, HlyIII family [Erwinia pyrifoliae DSM 12163]
292487134 12..214    |Erwinia amylovora CFBP1430                    |B|Proteobacteria Gammaproteobacteria | >gi|292487134|ref|YP_003530004.1| putative channel protein, HlyIII family [Erwinia amylovora CFBP1430]
260771082 7..209     |Vibrio furnissii CIP 102972                   |B|Proteobacteria Gammaproteobacteria | >gi|260771082|ref|ZP_05880010.1| hypothetical protein VFA_004148 [Vibrio furnissii CIP 102972]
260775009 10..212    |Vibrio coralliilyticus ATCC BAA-450           |B|Proteobacteria Gammaproteobacteria | >gi|260775009|ref|ZP_05883909.1| hypothetical protein VIC_000382 [Vibrio coralliilyticus ATCC BAA-450]
261250610 10..212    |Vibrio orientalis CIP 102891                  |B|Proteobacteria Gammaproteobacteria | >gi|261250610|ref|ZP_05943185.1| hypothetical protein VIA_000629 [Vibrio orientalis CIP 102891]
262273085 11..213    |Grimontia hollisae CIP 101886                 |B|Proteobacteria Gammaproteobacteria | >gi|262273085|ref|ZP_06050902.1| hypothetical protein VHA_000062 [Grimontia hollisae CIP 101886]
269103781 12..214    |Photobacterium damselae subsp. damselae CIP 102761|B|Proteobacteria Gammaproteobacteria | >gi|269103781|ref|ZP_06156478.1| hypothetical protein VDA_003208 [Photobacterium damselae subsp. damselae CIP 102761]
270158305 31..233    |Legionella longbeachae D-4968                 |B|Proteobacteria Gammaproteobacteria | >gi|270158305|ref|ZP_06186962.1| hemolysin-3 [Legionella longbeachae D-4968]
289163445 11..213    |Legionella longbeachae NSW150                 |B|Proteobacteria Gammaproteobacteria | >gi|289163445|ref|YP_003453583.1| hemolysin, inner membrane subunit [Legionella longbeachae NSW150]
270501754 1..203     |Micromonospora aurantiaca ATCC 27029          |B|Actinobacteria Actinobacteridae    | >gi|270501754|ref|ZP_06218671.1| channel protein, hemolysin III family [Micromonospora aurantiaca ATCC 27029]
271499204 17..219    |Dickeya dadantii Ech586                       |B|Proteobacteria Gammaproteobacteria | >gi|271499204|ref|YP_003332229.1| hemolysin III family channel protein [Dickeya dadantii Ech586]
27364475  7..209     |Vibrio vulnificus CMCP6                       |B|Proteobacteria Gammaproteobacteria | >gi|27364475|ref|NP_760003.1| hemolysin [Vibrio vulnificus CMCP6]
37681414  7..209     |Vibrio vulnificus YJ016                       |B|Proteobacteria Gammaproteobacteria | >gi|37681414|ref|NP_936023.1| hemolysin III-like protein [Vibrio vulnificus YJ016]
38489891  7..209     |Vibrio vulnificus                             |B|Proteobacteria Gammaproteobacteria | >gi|38489891|gb|AAP50516.2| hemolysin III [Vibrio vulnificus]
281421615 18..220    |Prevotella copri DSM 18205                    |B|Bacteroidetes Bacteroidia          | >gi|281421615|ref|ZP_06252614.1| hemolysin III [Prevotella copri DSM 18205]
281423746 25..227    |Prevotella oris F0302                         |B|Bacteroidetes Bacteroidia          | >gi|281423746|ref|ZP_06254659.1| hemolysin III [Prevotella oris F0302]
299141198 12..214    |Prevotella oris C735                          |B|Bacteroidetes Bacteroidia          | >gi|299141198|ref|ZP_07034335.1| hemolysin III [Prevotella oris C735]
28210321  8..210     |Clostridium tetani E88                        |B|Firmicutes Clostridia              | >gi|28210321|ref|NP_781265.1| hemolysin III [Clostridium tetani E88]
284989527 60..262    |Geodermatophilus obscurus DSM 43160           |B|Actinobacteria Actinobacteridae    | >gi|284989527|ref|YP_003408081.1| hemolysin III family channel protein [Geodermatophilus obscurus DSM 43160]
288925854 9..211     |Prevotella buccae D17                         |B|Bacteroidetes Bacteroidia          | >gi|288925854|ref|ZP_06419784.1| hemolysin III [Prevotella buccae D17]
289550218 24..226    |Staphylococcus lugdunensis HKU09-01           |B|Firmicutes Bacillales              | >gi|289550218|ref|YP_003471122.1| Hemolysin III [Staphylococcus lugdunensis HKU09-01]
290745615 24..226    |Danio rerio                                   |E|Metazoa Chordata                   | >gi|290745615|gb|ADD51543.1| Paqr11 [Danio rerio]
82658274  24..226    |Danio rerio                                   |E|Metazoa Chordata                   | >gi|82658274|ref|NP_001032496.1| monocyte to macrophage differentiation protein [Danio rerio]
291614450 17..219    |Sideroxydans lithotrophicus ES-1              |B|Proteobacteria Betaproteobacteria  | >gi|291614450|ref|YP_003524607.1| channel protein, hemolysin III family [Sideroxydans lithotrophicus ES-1]
293606381 9..211     |Achromobacter piechaudii ATCC 43553           |B|Proteobacteria Betaproteobacteria  | >gi|293606381|ref|ZP_06688741.1| hemolysin III [Achromobacter piechaudii ATCC 43553]
294497730 8..210     |Bacillus megaterium QM B1551                  |B|Firmicutes Bacillales              | >gi|294497730|ref|YP_003561430.1| hemolysin III [Bacillus megaterium QM B1551]
294634855 12..214    |Edwardsiella tarda ATCC 23685                 |B|Proteobacteria Gammaproteobacteria | >gi|294634855|ref|ZP_06713377.1| hemolysin III [Edwardsiella tarda ATCC 23685]
295134720 11..213    |Zunongwangia profunda SM-A87                  |B|Bacteroidetes Flavobacteria        | >gi|295134720|ref|YP_003585396.1| hemolysin-3 family protein [Zunongwangia profunda SM-A87]
295426283 18..220    |Lactobacillus amylolyticus DSM 11664          |B|Firmicutes Lactobacillales         | >gi|295426283|ref|ZP_06818943.1| hemolysin III [Lactobacillus amylolyticus DSM 11664]
296039349 14..216    |Rhodococcus equi ATCC 33707                   |B|Actinobacteria Actinobacteridae    | >gi|296039349|ref|ZP_06831910.1| hemolysin III family channel protein [Rhodococcus equi ATCC 33707]
296111380 8..210     |Leuconostoc kimchii IMSNU 11154               |B|Firmicutes Lactobacillales         | >gi|296111380|ref|YP_003621762.1| hemolysin III [Leuconostoc kimchii IMSNU 11154]
296118904 50..252    |Corynebacterium ammoniagenes DSM 20306        |B|Actinobacteria Actinobacteridae    | >gi|296118904|ref|ZP_06837477.1| channel protein, hemolysin III family [Corynebacterium ammoniagenes DSM 20306]
296139251 12..214    |Tsukamurella paurometabola DSM 20162          |B|Actinobacteria Actinobacteridae    | >gi|296139251|ref|YP_003646494.1| channel protein, hemolysin III family [Tsukamurella paurometabola DSM 20162]
297538538 13..215    |Methylotenera sp. 301                         |B|Proteobacteria Betaproteobacteria  | >gi|297538538|ref|YP_003674307.1| hemolysin III family channel protein [Methylotenera sp. 301]
298250766 20..222    |Ktedonobacter racemifer DSM 44963             |B|Chloroflexi Ktedonobacteria        | >gi|298250766|ref|ZP_06974570.1| Hly-III family protein [Ktedonobacter racemifer DSM 44963]
299064044 12..214    |Erwinia billingiae Eb661                      |B|Proteobacteria Gammaproteobacteria | >gi|299064044|emb|CAX61164.1| hemolysin III family channel protein [Erwinia billingiae Eb661]
52080688  3..205     |Bacillus licheniformis ATCC 14580             |B|Firmicutes Bacillales              | >gi|52080688|ref|YP_079479.1| putative membrane protein, hemolysin III-like protein [Bacillus licheniformis ATCC 14580]
54310622  12..214    |Photobacterium profundum SS9                  |B|Proteobacteria Gammaproteobacteria | >gi|54310622|ref|YP_131642.1| putative hemolysin [Photobacterium profundum SS9]
90413774  12..214    |Photobacterium profundum 3TCK                 |B|Proteobacteria Gammaproteobacteria | >gi|90413774|ref|ZP_01221762.1| Putative Hemolysin [Photobacterium profundum 3TCK]
56461539  9..211     |Idiomarina loihiensis L2TR                    |B|Proteobacteria Gammaproteobacteria | >gi|56461539|ref|YP_156820.1| hemolysin III-like protein [Idiomarina loihiensis L2TR]
56964297  8..210     |Bacillus clausii KSM-K16                      |B|Firmicutes Bacillales              | >gi|56964297|ref|YP_176028.1| hemolysin [Bacillus clausii KSM-K16]
58337271  18..220    |Lactobacillus acidophilus NCFM                |B|Firmicutes Lactobacillales         | >gi|58337271|ref|YP_193856.1| channel-forming protein [Lactobacillus acidophilus NCFM]
227903856 45..236    |Lactobacillus acidophilus ATCC 4796           |B|Firmicutes Lactobacillales         | >gi|227903856|ref|ZP_04021661.1| hemolysin III [Lactobacillus acidophilus ATCC 4796]
70725872  23..225    |Staphylococcus haemolyticus JCSC1435          |B|Firmicutes Bacillales              | >gi|70725872|ref|YP_252786.1| hypothetical protein SH0871 [Staphylococcus haemolyticus JCSC1435]
73662026  23..225    |Staphylococcus saprophyticus subsp. saprophyticus ATCC 15305|B|Firmicutes Bacillales              | >gi|73662026|ref|YP_300807.1| hypothetical protein SSP0717 [Staphylococcus saprophyticus subsp. saprophyticus ATCC 15305]
82701970  16..218    |Nitrosospira multiformis ATCC 25196           |B|Proteobacteria Betaproteobacteria  | >gi|82701970|ref|YP_411536.1| hemolysin III family channel protein [Nitrosospira multiformis ATCC 25196]
85711504  7..209     |Idiomarina baltica OS145                      |B|Proteobacteria Gammaproteobacteria | >gi|85711504|ref|ZP_01042562.1| hemolysin III-like protein [Idiomarina baltica OS145]
89074755  12..214    |Photobacterium sp. SKA34                      |B|Proteobacteria Gammaproteobacteria | >gi|89074755|ref|ZP_01161213.1| Putative Hemolysin [Photobacterium sp. SKA34]
90581170  12..214    |Photobacterium angustum S14                   |B|Proteobacteria Gammaproteobacteria | >gi|90581170|ref|ZP_01236969.1| Putative Hemolysin [Vibrio angustum S14]
92112249  17..219    |Chromohalobacter salexigens DSM 3043          |B|Proteobacteria Gammaproteobacteria | >gi|92112249|ref|YP_572177.1| hemolysin III family channel protein [Chromohalobacter salexigens DSM 3043]
94500910  8..210     |Bermanella marisrubri                         |B|Proteobacteria Gammaproteobacteria | >gi|94500910|ref|ZP_01307436.1| hemolysin III [Oceanobacter sp. RED65]
1006665   24..225    |Homo sapiens                                  |E|Metazoa Chordata                   | >gi|1006665|emb|CAA59752.1| unnamed protein product [Homo sapiens]
113205824 24..225    |Sus scrofa                                    |E|Metazoa Chordata                   | >gi|113205824|ref|NP_001038060.1| monocyte to macrophage differentiation protein [Sus scrofa]
115497828 24..225    |Bos taurus                                    |E|Metazoa Chordata                   | >gi|115497828|ref|NP_001069069.1| monocyte to macrophage differentiation protein [Bos taurus]
126307468 24..225    |Monodelphis domestica                         |E|Metazoa Chordata                   | >gi|126307468|ref|XP_001363454.1| PREDICTED: monocyte to macrophage differentiation protein-like [Monodelphis domestica]
13385688  24..225    |Mus musculus                                  |E|Metazoa Chordata                   | >gi|13385688|ref|NP_080454.1| monocyte to macrophage differentiation protein [Mus musculus]
149053851 14..215    |Rattus norvegicus                             |E|Metazoa Chordata                   | >gi|149053851|gb|EDM05668.1| monocyte to macrophage differentiation-associated [Rattus norvegicus]
296477099 24..225    |Bos taurus                                    |E|Metazoa Chordata                   | >gi|296477099|gb|DAA19214.1| monocyte to macrophage differentiation-associated [Bos taurus]
57091163  24..225    |Canis lupus familiaris                        |E|Metazoa Chordata                   | >gi|57091163|ref|XP_537684.1| PREDICTED: similar to monocyte to macrophage differentiation-associated precursor [Canis familiaris]
74204095  24..217    |Mus musculus                                  |E|Metazoa Chordata                   | >gi|74204095|dbj|BAE29038.1| unnamed protein product [Mus musculus]
194383584 16..200    |Homo sapiens                                  |E|Metazoa Chordata                   | >gi|194383584|dbj|BAG64763.1| unnamed protein product [Homo sapiens]
296202415 16..200    |Callithrix jacchus                            |E|Metazoa Chordata                   | >gi|296202415|ref|XP_002748450.1| PREDICTED: monocyte to macrophage differentiation protein-like isoform 2 [Callithrix jacchus]
297272620 16..200    |Macaca mulatta                                |E|Metazoa Chordata                   | >gi|297272620|ref|XP_002800468.1| PREDICTED: monocyte to macrophage differentiation protein-like isoform 2 [Macaca mulatta]
56206228  24..176    |Mus musculus                                  |E|Metazoa Chordata                   | >gi|56206228|emb|CAI24626.1| monocyte to macrophage differentiation-associated [Mus musculus]
148683936 11..146    |Mus musculus                                  |E|Metazoa Chordata                   | >gi|148683936|gb|EDL15883.1| monocyte to macrophage differentiation-associated [Mus musculus]
149262352 62..195    |Mus musculus                                  |E|Metazoa Chordata                   | >gi|149262352|ref|XP_001478460.1| PREDICTED: hypothetical protein [Mus musculus]
113970217 23..224    |Shewanella sp. MR-4                           |B|Proteobacteria Gammaproteobacteria | >gi|113970217|ref|YP_734010.1| hemolysin III family channel protein [Shewanella sp. MR-4]
114047594 23..224    |Shewanella sp. MR-7                           |B|Proteobacteria Gammaproteobacteria | >gi|114047594|ref|YP_738144.1| hemolysin III family channel protein [Shewanella sp. MR-7]
117920379 23..224    |Shewanella sp. ANA-3                          |B|Proteobacteria Gammaproteobacteria | >gi|117920379|ref|YP_869571.1| hemolysin III family channel protein [Shewanella sp. ANA-3]
114330506 13..214    |Nitrosomonas eutropha C91                     |B|Proteobacteria Betaproteobacteria  | >gi|114330506|ref|YP_746728.1| channel protein, hemolysin III family protein [Nitrosomonas eutropha C91]
115526623 16..217    |Rhodopseudomonas palustris BisA53             |B|Proteobacteria Alphaproteobacteria | >gi|115526623|ref|YP_783534.1| Hly-III family protein [Rhodopseudomonas palustris BisA53]
116494875 11..212    |Lactobacillus casei ATCC 334                  |B|Firmicutes Lactobacillales         | >gi|116494875|ref|YP_806609.1| hemolysin III-like protein [Lactobacillus casei ATCC 334]
191638382 11..212    |Lactobacillus casei BL23                      |B|Firmicutes Lactobacillales         | >gi|191638382|ref|YP_001987548.1| Hemolysin III [lactobacillus casei BL23]
239631531 11..212    |Lactobacillus paracasei subsp. paracasei 8700:2|B|Firmicutes Lactobacillales         | >gi|239631531|ref|ZP_04674562.1| hemolysin III [Lactobacillus paracasei subsp. paracasei 8700:2]
116617280 9..210     |Leuconostoc mesenteroides subsp. mesenteroides ATCC 8293|B|Firmicutes Lactobacillales         | >gi|116617280|ref|YP_817651.1| hemolysin III-like protein [Leuconostoc mesenteroides subsp. mesenteroides ATCC 8293]
116627463 20..221    |Streptococcus thermophilus LMD-9              |B|Firmicutes Lactobacillales         | >gi|116627463|ref|YP_820082.1| hemolysin III-like protein [Streptococcus thermophilus LMD-9]
228478429 20..221    |Streptococcus salivarius SK126                |B|Firmicutes Lactobacillales         | >gi|228478429|ref|ZP_04063037.1| hemolysin-3 [Streptococcus salivarius SK126]
55820654  20..221    |Streptococcus thermophilus LMG 18311          |B|Firmicutes Lactobacillales         | >gi|55820654|ref|YP_139096.1| hypothetical protein stu0575 [Streptococcus thermophilus LMG 18311]
116629564 19..220    |Lactobacillus gasseri ATCC 33323              |B|Firmicutes Lactobacillales         | >gi|116629564|ref|YP_814736.1| hemolysin III-like protein [Lactobacillus gasseri ATCC 33323]
227520816 19..220    |Lactobacillus gasseri JV-V03                  |B|Firmicutes Lactobacillales         | >gi|227520816|ref|ZP_03950865.1| hemolysin III [Lactobacillus gasseri JV-V03]
227889889 19..220    |Lactobacillus johnsonii ATCC 33200            |B|Firmicutes Lactobacillales         | >gi|227889889|ref|ZP_04007694.1| hemolysin III [Lactobacillus johnsonii ATCC 33200]
238853077 19..220    |Lactobacillus gasseri 202-4                   |B|Firmicutes Lactobacillales         | >gi|238853077|ref|ZP_04643469.1| hemolysin-3 [Lactobacillus gasseri 202-4]
282852057 19..220    |Lactobacillus gasseri 224-1                   |B|Firmicutes Lactobacillales         | >gi|282852057|ref|ZP_06261415.1| channel protein, hemolysin III family protein [Lactobacillus gasseri 224-1]
42519027  19..220    |Lactobacillus johnsonii NCC 533               |B|Firmicutes Lactobacillales         | >gi|42519027|ref|NP_964957.1| hemolysin-like protein [Lactobacillus johnsonii NCC 533]
119025671 87..288    |Bifidobacterium adolescentis ATCC 15703       |B|Actinobacteria Actinobacteridae    | >gi|119025671|ref|YP_909516.1| hypothetical protein BAD_0653 [Bifidobacterium adolescentis ATCC 15703]
120598896 23..224    |Shewanella sp. W3-18-1                        |B|Proteobacteria Gammaproteobacteria | >gi|120598896|ref|YP_963470.1| hemolysin III family channel protein [Shewanella sp. W3-18-1]
124546746 23..224    |Shewanella putrefaciens 200                   |B|Proteobacteria Gammaproteobacteria | >gi|124546746|ref|ZP_01705781.1| channel protein, hemolysin III family [Shewanella putrefaciens 200]
146293018 23..224    |Shewanella putrefaciens CN-32                 |B|Proteobacteria Gammaproteobacteria | >gi|146293018|ref|YP_001183442.1| hemolysin III family channel protein [Shewanella putrefaciens CN-32]
125718213 18..219    |Streptococcus sanguinis SK36                  |B|Firmicutes Lactobacillales         | >gi|125718213|ref|YP_001035346.1| hemolysin III-like [Streptococcus sanguinis SK36]
126174538 23..224    |Shewanella baltica OS155                      |B|Proteobacteria Gammaproteobacteria | >gi|126174538|ref|YP_001050687.1| hemolysin III family channel protein [Shewanella baltica OS155]
153000845 23..224    |Shewanella baltica OS185                      |B|Proteobacteria Gammaproteobacteria | >gi|153000845|ref|YP_001366526.1| hemolysin III family channel protein [Shewanella baltica OS185]
160875553 23..224    |Shewanella baltica OS195                      |B|Proteobacteria Gammaproteobacteria | >gi|160875553|ref|YP_001554869.1| hemolysin III family channel protein [Shewanella baltica OS195]
217973197 23..224    |Shewanella baltica OS223                      |B|Proteobacteria Gammaproteobacteria | >gi|217973197|ref|YP_002357948.1| hemolysin III family channel protein [Shewanella baltica OS223]
126650230 9..210     |Bacillus sp. B14905                           |B|Firmicutes Bacillales              | >gi|126650230|ref|ZP_01722458.1| hemolysin III [Bacillus sp. B14905]
169828237 9..210     |Lysinibacillus sphaericus C3-41               |B|Firmicutes Bacillales              | >gi|169828237|ref|YP_001698395.1| hemolysin-3 [Lysinibacillus sphaericus C3-41]
127513026 32..233    |Shewanella loihica PV-4                       |B|Proteobacteria Gammaproteobacteria | >gi|127513026|ref|YP_001094223.1| hemolysin III family channel protein [Shewanella loihica PV-4]
146318648 12..213    |Streptococcus suis 05ZYH33                    |B|Firmicutes Lactobacillales         | >gi|146318648|ref|YP_001198360.1| hypothetical protein SSU05_0994 [Streptococcus suis 05ZYH33]
148379423 7..208     |Clostridium botulinum A str. ATCC 3502        |B|Firmicutes Clostridia              | >gi|148379423|ref|YP_001253964.1| hemolysin III [Clostridium botulinum A str. ATCC 3502]
170758667 7..208     |Clostridium botulinum A3 str. Loch Maree      |B|Firmicutes Clostridia              | >gi|170758667|ref|YP_001786874.1| hemolysin III [Clostridium botulinum A3 str. Loch Maree]
187779897 7..208     |Clostridium sporogenes ATCC 15579             |B|Firmicutes Clostridia              | >gi|187779897|ref|ZP_02996370.1| hypothetical protein CLOSPO_03493 [Clostridium sporogenes ATCC 15579]
226948787 7..208     |Clostridium botulinum A2 str. Kyoto           |B|Firmicutes Clostridia              | >gi|226948787|ref|YP_002803878.1| hemolysin III [Clostridium botulinum A2 str. Kyoto]
148984809 12..213    |Streptococcus pneumoniae SP3-BS71             |B|Firmicutes Lactobacillales         | >gi|148984809|ref|ZP_01818062.1| hemolysin [Streptococcus pneumoniae SP3-BS71]
148992650 12..213    |Streptococcus pneumoniae SP9-BS68             |B|Firmicutes Lactobacillales         | >gi|148992650|ref|ZP_01822318.1| hemolysin [Streptococcus pneumoniae SP9-BS68]
149007263 12..213    |Streptococcus pneumoniae SP18-BS74            |B|Firmicutes Lactobacillales         | >gi|149007263|ref|ZP_01830921.1| hemolysin [Streptococcus pneumoniae SP18-BS74]
149011694 12..213    |Streptococcus pneumoniae SP19-BS75            |B|Firmicutes Lactobacillales         | >gi|149011694|ref|ZP_01832890.1| hemolysin [Streptococcus pneumoniae SP19-BS75]
15901316  12..213    |Streptococcus pneumoniae TIGR4                |B|Firmicutes Lactobacillales         | >gi|15901316|ref|NP_345920.1| hemolysin [Streptococcus pneumoniae TIGR4]
168483923 12..213    |Streptococcus pneumoniae CDC1873-00           |B|Firmicutes Lactobacillales         | >gi|168483923|ref|ZP_02708875.1| hemolysin-3 (Hemolysin III) (Hly-III) [Streptococcus pneumoniae CDC1873-00]
194397266 12..213    |Streptococcus pneumoniae G54                  |B|Firmicutes Lactobacillales         | >gi|194397266|ref|YP_002038099.1| hemolysin [Streptococcus pneumoniae G54]
225859227 12..213    |Streptococcus pneumoniae 70585                |B|Firmicutes Lactobacillales         | >gi|225859227|ref|YP_002740737.1| hemolysin-3 (Hemolysin III) (Hly-III) [Streptococcus pneumoniae 70585]
270293033 12..213    |Streptococcus sp. M143                        |B|Firmicutes Lactobacillales         | >gi|270293033|ref|ZP_06199244.1| hemolysin [Streptococcus sp. M143]
289168220 12..213    |Streptococcus mitis B6                        |B|Firmicutes Lactobacillales         | >gi|289168220|ref|YP_003446489.1| hlyIII superfamily protein [Streptococcus mitis B6]
149190440 10..211    |Vibrio shilonii AK1                           |B|Proteobacteria Gammaproteobacteria | >gi|149190440|ref|ZP_01868711.1| hemolysin, putative [Vibrio shilonii AK1]
149592771 34..235    |Ornithorhynchus anatinus                      |E|Metazoa Chordata                   | >gi|149592771|ref|XP_001521664.1| PREDICTED: hypothetical protein [Ornithorhynchus anatinus]
281343964 16..217    |Ailuropoda melanoleuca                        |E|Metazoa Chordata                   | >gi|281343964|gb|EFB19548.1| hypothetical protein PANDA_015108 [Ailuropoda melanoleuca]
291405762 24..225    |Oryctolagus cuniculus                         |E|Metazoa Chordata                   | >gi|291405762|ref|XP_002719328.1| PREDICTED: monocyte to macrophage differentiation-associated [Oryctolagus cuniculus]
296202413 24..225    |Callithrix jacchus                            |E|Metazoa Chordata                   | >gi|296202413|ref|XP_002748449.1| PREDICTED: monocyte to macrophage differentiation protein-like isoform 1 [Callithrix jacchus]
52630445  24..225    |Homo sapiens                                  |E|Metazoa Chordata                   | >gi|52630445|ref|NP_036461.2| monocyte to macrophage differentiation protein precursor [Homo sapiens]
56090321  24..225    |Rattus norvegicus                             |E|Metazoa Chordata                   | >gi|56090321|ref|NP_001007674.1| monocyte to macrophage differentiation protein [Rattus norvegicus]
71897109  25..226    |Gallus gallus                                 |E|Metazoa Chordata                   | >gi|71897109|ref|NP_001025875.1| monocyte to macrophage differentiation protein [Gallus gallus]
149908374 20..221    |Moritella sp. PE36                            |B|Proteobacteria Gammaproteobacteria | >gi|149908374|ref|ZP_01897037.1| hemolysin, putative [Moritella sp. PE36]
152992268 10..211    |Sulfurovum sp. NBC37-1                        |B|Proteobacteria Epsilonproteobacteria| >gi|152992268|ref|YP_001357989.1| hemolysin III [Sulfurovum sp. NBC37-1]
153802763 9..210     |Vibrio cholerae MZO-3                         |B|Proteobacteria Gammaproteobacteria | >gi|153802763|ref|ZP_01957349.1| hemolysin, putative [Vibrio cholerae MZO-3]
15640072  9..210     |Vibrio cholerae O1 biovar El Tor str. N16961  |B|Proteobacteria Gammaproteobacteria | >gi|15640072|ref|NP_229699.1| hemolysin, putative [Vibrio cholerae O1 biovar El Tor str. N16961]
229508325 7..208     |Vibrio cholerae BX 330286                     |B|Proteobacteria Gammaproteobacteria | >gi|229508325|ref|ZP_04397829.1| hypothetical protein VCF_003560 [Vibrio cholerae BX 330286]
229530174 7..208     |Vibrio cholerae 12129(1)                      |B|Proteobacteria Gammaproteobacteria | >gi|229530174|ref|ZP_04419563.1| hypothetical protein VCG_003285 [Vibrio cholerae 12129(1)]
258622984 7..208     |Vibrio mimicus VM573                          |B|Proteobacteria Gammaproteobacteria | >gi|258622984|ref|ZP_05717999.1| hemolysin, putative [Vibrio mimicus VM573]
261213235 7..208     |Vibrio sp. RC341                              |B|Proteobacteria Gammaproteobacteria | >gi|261213235|ref|ZP_05927517.1| hypothetical protein VCJ_003515 [Vibrio sp. RC341]
262166808 7..208     |Vibrio mimicus VM223                          |B|Proteobacteria Gammaproteobacteria | >gi|262166808|ref|ZP_06034545.1| hypothetical protein VMA_003276 [Vibrio mimicus VM223]
262402054 7..208     |Vibrio sp. RC586                              |B|Proteobacteria Gammaproteobacteria | >gi|262402054|ref|ZP_06078618.1| hypothetical protein VOA_000018 [Vibrio sp. RC586]
262172806 2..198     |Vibrio mimicus MB451                          |B|Proteobacteria Gammaproteobacteria | >gi|262172806|ref|ZP_06040484.1| hypothetical protein VII_003638 [Vibrio mimicus MB-451]
156544594 77..278    |Nasonia vitripennis                           |E|Metazoa Arthropoda                 | >gi|156544594|ref|XP_001603607.1| PREDICTED: similar to CG4615-PA [Nasonia vitripennis]
15675135  12..213    |Streptococcus pyogenes M1 GAS                 |B|Firmicutes Lactobacillales         | >gi|15675135|ref|NP_269309.1| hypothetical protein SPy_1159 [Streptococcus pyogenes M1 GAS]
94990503  12..213    |Streptococcus pyogenes MGAS10270              |B|Firmicutes Lactobacillales         | >gi|94990503|ref|YP_598603.1| hypothetical protein MGAS10270_Spy0994 [Streptococcus pyogenes MGAS10270]
209559439 12..171    |Streptococcus pyogenes NZ131                  |B|Firmicutes Lactobacillales         | >gi|209559439|ref|YP_002285911.1| Putative hemolysin III [Streptococcus pyogenes NZ131]
157151576 12..213    |Streptococcus gordonii str. Challis substr. CH1|B|Firmicutes Lactobacillales         | >gi|157151576|ref|YP_001450590.1| hemolysin III-like protein [Streptococcus gordonii str. Challis substr. CH1]
262282886 14..215    |Streptococcus sp. 2_1_36FAA                   |B|Firmicutes Lactobacillales         | >gi|262282886|ref|ZP_06060653.1| hemolysin [Streptococcus sp. 2_1_36FAA]
157372134 21..222    |Serratia proteamaculans 568                   |B|Proteobacteria Gammaproteobacteria | >gi|157372134|ref|YP_001480123.1| hemolysin III family channel protein [Serratia proteamaculans 568]
270264913 22..223    |Serratia odorifera 4Rx13                      |B|Proteobacteria Gammaproteobacteria | >gi|270264913|ref|ZP_06193177.1| hypothetical protein SOD_j01290 [Serratia odorifera 4Rx13]
157375149 22..223    |Shewanella sediminis HAW-EB3                  |B|Proteobacteria Gammaproteobacteria | >gi|157375149|ref|YP_001473749.1| hemolysin III family channel protein [Shewanella sediminis HAW-EB3]
158295408 149..350   |Anopheles gambiae str. PEST                   |E|Metazoa Arthropoda                 | >gi|158295408|ref|XP_316199.4| AGAP006136-PA [Anopheles gambiae str. PEST]
158312688 68..269    |Frankia sp. EAN1pec                           |B|Actinobacteria Actinobacteridae    | >gi|158312688|ref|YP_001505196.1| hemolysin III family channel protein [Frankia sp. EAN1pec]
15894234  8..209     |Clostridium acetobutylicum ATCC 824           |B|Firmicutes Clostridia              | >gi|15894234|ref|NP_347583.1| hemolysin III-like protein [Clostridium acetobutylicum ATCC 824]
159897336 16..217    |Herpetosiphon aurantiacus ATCC 23779          |B|Chloroflexi Herpetosiphonales      | >gi|159897336|ref|YP_001543583.1| hemolysin III family channel protein [Herpetosiphon aurantiacus ATCC 23779]
160902638 11..212    |Petrotoga mobilis SJ95                        |B|Thermotogae Thermotogales          | >gi|160902638|ref|YP_001568219.1| hemolysin III family channel protein [Petrotoga mobilis SJ95]
160915314 13..214    |Eubacterium dolichum DSM 3991                 |B|Firmicutes Erysipelotrichi         | >gi|160915314|ref|ZP_02077527.1| hypothetical protein EUBDOL_01323 [Eubacterium dolichum DSM 3991]
163751760 27..228    |Shewanella benthica KT99                      |B|Proteobacteria Gammaproteobacteria | >gi|163751760|ref|ZP_02158977.1| channel protein, hemolysin III family subfamily [Shewanella benthica KT99]
163753520 11..212    |Kordia algicida OT-1                          |B|Bacteroidetes Flavobacteria        | >gi|163753520|ref|ZP_02160644.1| hypothetical protein KAOT1_15207 [Kordia algicida OT-1]
163851035 17..218    |Methylobacterium extorquens PA1               |B|Proteobacteria Alphaproteobacteria | >gi|163851035|ref|YP_001639078.1| Hly-III family protein [Methylobacterium extorquens PA1]
218529864 17..218    |Methylobacterium chloromethanicum CM4         |B|Proteobacteria Alphaproteobacteria | >gi|218529864|ref|YP_002420680.1| Hly-III family protein [Methylobacterium chloromethanicum CM4]
169333800 13..214    |Anaerofustis stercorihominis DSM 17244        |B|Firmicutes Clostridia              | >gi|169333800|ref|ZP_02860993.1| hypothetical protein ANASTE_00186 [Anaerofustis stercorihominis DSM 17244]
170017987 9..210     |Leuconostoc citreum KM20                      |B|Firmicutes Lactobacillales         | >gi|170017987|ref|YP_001728906.1| hemolysin III-like protein [Leuconostoc citreum KM20]
170590065 14..215    |Brugia malayi                                 |E|Metazoa Nematoda                   | >gi|170590065|ref|XP_001899793.1| Haemolysin-III related family protein [Brugia malayi]
170726940 22..223    |Shewanella woodyi ATCC 51908                  |B|Proteobacteria Gammaproteobacteria | >gi|170726940|ref|YP_001760966.1| hemolysin III family channel protein [Shewanella woodyi ATCC 51908]
170749117 17..218    |Methylobacterium radiotolerans JCM 2831       |B|Proteobacteria Alphaproteobacteria | >gi|170749117|ref|YP_001755377.1| Hly-III family protein [Methylobacterium radiotolerans JCM 2831]
171778299 14..215    |Streptococcus infantarius subsp. infantarius ATCC BAA-102|B|Firmicutes Lactobacillales         | >gi|171778299|ref|ZP_02919505.1| hypothetical protein STRINF_00347 [Streptococcus infantarius subsp. infantarius ATCC BAA-102]
182413042 310..511   |Opitutus terrae PB90-1                        |B|Verrucomicrobia Opitutae           | >gi|182413042|ref|YP_001818108.1| hemolysin III family channel protein [Opitutus terrae PB90-1]
182413504 9..210     |Opitutus terrae PB90-1                        |B|Verrucomicrobia Opitutae           | >gi|182413504|ref|YP_001818570.1| hemolysin III family channel protein [Opitutus terrae PB90-1]
187250756 13..214    |Elusimicrobium minutum Pei191                 |B|Elusimicrobia Elusimicrobia (class)| >gi|187250756|ref|YP_001875238.1| channel protein, hemolysin III family [Elusimicrobium minutum Pei191]
188580885 17..218    |Methylobacterium populi BJ001                 |B|Proteobacteria Alphaproteobacteria | >gi|188580885|ref|YP_001924330.1| Hly-III family protein [Methylobacterium populi BJ001]
189439630 106..307   |Bifidobacterium longum DJO10A                 |B|Actinobacteria Actinobacteridae    | >gi|189439630|ref|YP_001954711.1| hemolysin III-like membrane protein [Bifidobacterium longum DJO10A]
213692642 106..307   |Bifidobacterium longum subsp. infantis ATCC 15697|B|Actinobacteria Actinobacteridae    | >gi|213692642|ref|YP_002323228.1| Hly-III family protein [Bifidobacterium longum subsp. infantis ATCC 15697]
23465583  64..265    |Bifidobacterium longum NCC2705                |B|Actinobacteria Actinobacteridae    | >gi|23465583|ref|NP_696186.1| hypothetical protein BL1014 [Bifidobacterium longum NCC2705]
239621778 95..296    |Bifidobacterium longum subsp. infantis CCUG 52486|B|Actinobacteria Actinobacteridae    | >gi|239621778|ref|ZP_04664809.1| conserved hypothetical protein [Bifidobacterium longum subsp. infantis CCUG 52486]
296453852 106..307   |Bifidobacterium longum subsp. longum JDM301   |B|Actinobacteria Actinobacteridae    | >gi|296453852|ref|YP_003660995.1| Hly-III family protein [Bifidobacterium longum subsp. longum JDM301]
291456475 107..307   |Bifidobacterium breve DSM 20213               |B|Actinobacteria Actinobacteridae    | >gi|291456475|ref|ZP_06595865.1| hemolysin III family protein [Bifidobacterium breve DSM 20213]
46190238  95..210    |Bifidobacterium longum DJO10A                 |B|Actinobacteria Actinobacteridae    | >gi|46190238|ref|ZP_00121751.2| COG1272: Predicted membrane protein, hemolysin III homolog [Bifidobacterium longum DJO10A]
193216071 18..219    |Chloroherpeton thalassium ATCC 35110          |B|Chlorobi Chlorobia                 | >gi|193216071|ref|YP_001997270.1| channel protein, hemolysin III family [Chloroherpeton thalassium ATCC 35110]
195978086 12..213    |Streptococcus equi subsp. zooepidemicus MGCS10565|B|Firmicutes Lactobacillales         | >gi|195978086|ref|YP_002123330.1| hemolysin III HylIII [Streptococcus equi subsp. zooepidemicus MGCS10565]
225868586 12..213    |Streptococcus equi subsp. zooepidemicus       |B|Firmicutes Lactobacillales         | >gi|225868586|ref|YP_002744534.1| haemolysin-III related membrane protein [Streptococcus equi subsp. zooepidemicus]
199598693 11..212    |Lactobacillus rhamnosus HN001                 |B|Firmicutes Lactobacillales         | >gi|199598693|ref|ZP_03212107.1| Predicted membrane protein, hemolysin III related protein [Lactobacillus rhamnosus HN001]
229552191 11..212    |Lactobacillus rhamnosus LMS2-1                |B|Firmicutes Lactobacillales         | >gi|229552191|ref|ZP_04440916.1| hemolysin III [Lactobacillus rhamnosus LMS2-1]
258539606 11..166    |Lactobacillus rhamnosus Lc 705                |B|Firmicutes Lactobacillales         | >gi|258539606|ref|YP_003174105.1| hemolysin III-like protein [Lactobacillus rhamnosus Lc 705]
212639117 14..215    |Anoxybacillus flavithermus WK1                |B|Firmicutes Bacillales              | >gi|212639117|ref|YP_002315637.1| putative membrane protein, hemolysin III [Anoxybacillus flavithermus WK1]
213510750 24..225    |Salmo salar                                   |E|Metazoa Chordata                   | >gi|213510750|ref|NP_001133798.1| Monocyte to macrophage differentiation protein [Salmo salar]
218283538 15..216    |Eubacterium biforme DSM 3989                  |B|Firmicutes Erysipelotrichi         | >gi|218283538|ref|ZP_03489528.1| hypothetical protein EUBIFOR_02118 [Eubacterium biforme DSM 3989]
219118554 346..547   |Phaeodactylum tricornutum CCAP 1055/1         |E|stramenopiles Bacillariophyta      | >gi|219118554|ref|XP_002180047.1| predicted protein [Phaeodactylum tricornutum CCAP 1055/1]
220925691 25..226    |Methylobacterium nodulans ORS 2060            |B|Proteobacteria Alphaproteobacteria | >gi|220925691|ref|YP_002500993.1| Hly-III family protein [Methylobacterium nodulans ORS 2060]
222152055 18..219    |Macrococcus caseolyticus JCSC5402             |B|Firmicutes Bacillales              | >gi|222152055|ref|YP_002561215.1| hypothetical protein MCCL_1812 [Macrococcus caseolyticus JCSC5402]
224282818 87..288    |Bifidobacterium bifidum NCIMB 41171           |B|Actinobacteria Actinobacteridae    | >gi|224282818|ref|ZP_03646140.1| hypothetical protein BbifN4_03219 [Bifidobacterium bifidum NCIMB 41171]
224370470 21..222    |Desulfobacterium autotrophicum HRM2           |B|Proteobacteria Deltaproteobacteria | >gi|224370470|ref|YP_002604634.1| putative hemolysin-III related protein [Desulfobacterium autotrophicum HRM2]
22537466  10..211    |Streptococcus agalactiae 2603V/R              |B|Firmicutes Lactobacillales         | >gi|22537466|ref|NP_688317.1| hemolysin III [Streptococcus agalactiae 2603V/R]
25011431  14..215    |Streptococcus agalactiae NEM316               |B|Firmicutes Lactobacillales         | >gi|25011431|ref|NP_735826.1| hypothetical protein gbs1389 [Streptococcus agalactiae NEM316]
76786976  10..211    |Streptococcus agalactiae A909                 |B|Firmicutes Lactobacillales         | >gi|76786976|ref|YP_329960.1| hemolysin III [Streptococcus agalactiae A909]
76798606  1..184     |Streptococcus agalactiae 18RS21               |B|Firmicutes Lactobacillales         | >gi|76798606|ref|ZP_00780834.1| hemolysin III [Streptococcus agalactiae 18RS21]
77406857  1..184     |Streptococcus agalactiae H36B                 |B|Firmicutes Lactobacillales         | >gi|77406857|ref|ZP_00783885.1| MW2096 [Streptococcus agalactiae H36B]
229555607 21..222    |Listeria grayi DSM 20601                      |B|Firmicutes Bacillales              | >gi|229555607|ref|ZP_04443396.1| Hly III family protein [Listeria grayi DSM 20601]
23099720  8..209     |Oceanobacillus iheyensis HTE831               |B|Firmicutes Bacillales              | >gi|23099720|ref|NP_693186.1| hemolysin III [Oceanobacillus iheyensis HTE831]
24373994  13..214    |Shewanella oneidensis MR-1                    |B|Proteobacteria Gammaproteobacteria | >gi|24373994|ref|NP_718037.1| hemolysin III family channel protein [Shewanella oneidensis MR-1]
24379384  14..215    |Streptococcus mutans UA159                    |B|Firmicutes Lactobacillales         | >gi|24379384|ref|NP_721339.1| putative hemolysin III [Streptococcus mutans UA159]
253573490 8..209     |Paenibacillus sp. oral taxon 786 str. D14     |B|Firmicutes Bacillales              | >gi|253573490|ref|ZP_04850833.1| channel protein [Paenibacillus sp. oral taxon 786 str. D14]
254449365 5..206     |gamma proteobacterium HTCC5015                |B|Proteobacteria Gammaproteobacteria.| >gi|254449365|ref|ZP_05062808.1| channel protein, hemolysin III family [gamma proteobacterium HTCC5015]
254481615 5..206     |marine gamma proteobacterium HTCC2148         |B|Proteobacteria Gammaproteobacteria | >gi|254481615|ref|ZP_05094859.1| channel protein, hemolysin III family [marine gamma proteobacterium HTCC2148]
257062777 55..256    |Slackia heliotrinireducens DSM 20476          |B|Actinobacteria Coriobacteridae     | >gi|257062777|ref|YP_003142449.1| channel protein, hemolysin III family [Slackia heliotrinireducens DSM 20476]
257125313 24..225    |Leptotrichia buccalis C-1013-b                |B|Fusobacteria Fusobacteriales       | >gi|257125313|ref|YP_003163427.1| channel protein, hemolysin III family [Leptotrichia buccalis C-1013-b]
259500650 19..220    |Lactobacillus iners DSM 13335                 |B|Firmicutes Lactobacillales         | >gi|259500650|ref|ZP_05743552.1| hemolysin III [Lactobacillus iners DSM 13335]
260889989 22..223    |Leptotrichia hofstadii F0254                  |B|Fusobacteria Fusobacteriales       | >gi|260889989|ref|ZP_05901252.1| hemolysin III [Leptotrichia hofstadii F0254]
261338656 94..295    |Bifidobacterium gallicum DSM 20093            |B|Actinobacteria Actinobacteridae    | >gi|261338656|ref|ZP_05966540.1| hemolysin III family protein [Bifidobacterium gallicum DSM 20093]
261379114 5..206     |Neisseria cinerea ATCC 14685                  |B|Proteobacteria Betaproteobacteria  | >gi|261379114|ref|ZP_05983687.1| hemolysin III [Neisseria cinerea ATCC 14685]
262038089 15..216    |Leptotrichia goodfellowii F0264               |B|Fusobacteria Fusobacteriales       | >gi|262038089|ref|ZP_06011494.1| hemolysin-3 [Leptotrichia goodfellowii F0264]
288553412 8..209     |Bacillus pseudofirmus OF4                     |B|Firmicutes Bacillales              | >gi|288553412|ref|YP_003425347.1| putative hemolysin III family channel protein [Bacillus pseudofirmus OF4]
288798736 12..213    |Desulfurispirillum indicum S5                 |B|Chrysiogenetes Chrysiogenales      | >gi|288798736|ref|ZP_06404283.1| channel protein, hemolysin III family [bacterium S5]
288905463 14..215    |Streptococcus gallolyticus UCN34              |B|Firmicutes Lactobacillales         | >gi|288905463|ref|YP_003430685.1| hypothetical protein GALLO_1262 [Streptococcus gallolyticus UCN34]
290968355 7..208     |Megasphaera genomosp. type_1 str. 28L         |B|Firmicutes Negativicutes           | >gi|290968355|ref|ZP_06559896.1| channel protein, hemolysin III family protein [Megasphaera genomosp. type_1 str. 28L]
291544438 9..210     |Ruminococcus sp. 18P13                        |B|Firmicutes Clostridia              | >gi|291544438|emb|CBL17547.1| channel protein, hemolysin III family [Ruminococcus sp. 18P13]
293394595 36..237    |Serratia odorifera DSM 4582                   |B|Proteobacteria Gammaproteobacteria | >gi|293394595|ref|ZP_06638889.1| hemolysin III [Serratia odorifera DSM 4582]
293400482 20..221    |Erysipelotrichaceae bacterium 5_2_54FAA       |B|Firmicutes Erysipelotrichi         | >gi|293400482|ref|ZP_06644627.1| hemolysin [Erysipelotrichaceae bacterium 5_2_54FAA]
294141144 20..221    |Shewanella violacea DSS12                     |B|Proteobacteria Gammaproteobacteria | >gi|294141144|ref|YP_003557122.1| hemolysin III [Shewanella violacea DSS12]
294640230 13..214    |Ruminococcus albus 8                          |B|Firmicutes Clostridia              | >gi|294640230|ref|ZP_06718228.1| channel protein, hemolysin III family protein [Ruminococcus albus 8]
294790722 98..299    |Scardovia inopinata F0304                     |B|Actinobacteria Actinobacteridae    | >gi|294790722|ref|ZP_06755880.1| membrane protein, hemolysin III-like protein [Scardovia inopinata F0304]
295099736 9..210     |Eubacterium cylindroides T2-87                |B|Firmicutes Erysipelotrichi         | >gi|295099736|emb|CBK88825.1| Predicted membrane protein, hemolysin III homolog [Eubacterium cylindroides T2-87]
295397878 20..221    |Aerococcus viridans ATCC 11563                |B|Firmicutes Lactobacillales         | >gi|295397878|ref|ZP_06807940.1| hemolysin III [Aerococcus viridans ATCC 11563]
296876367 12..213    |Streptococcus parasanguinis ATCC 15912        |B|Firmicutes Lactobacillales         | >gi|296876367|ref|ZP_06900419.1| hemolysin III [Streptococcus parasanguinis ATCC 15912]
298369305 5..206     |Neisseria sp. oral taxon 014 str. F0314       |B|Proteobacteria Betaproteobacteria  | >gi|298369305|ref|ZP_06980623.1| hemolysin III [Neisseria sp. oral taxon 014 str. F0314]
298727447 9..210     |Lysinibacillus fusiformis ZC1                 |B|Firmicutes Bacillales              | >gi|298727447|gb|EFI68019.1| hemolysin-3 [Lysinibacillus fusiformis ZC1]
47204092  30..231    |Tetraodon nigroviridis                        |E|Metazoa Chordata                   | >gi|47204092|emb|CAF92958.1| unnamed protein product [Tetraodon nigroviridis]
74318725  13..214    |Thiobacillus denitrificans ATCC 25259         |B|Proteobacteria Betaproteobacteria  | >gi|74318725|ref|YP_316465.1| hemolysin-like protein [Thiobacillus denitrificans ATCC 25259]
86742577  101..302   |Frankia sp. CcI3                              |B|Actinobacteria Actinobacteridae    | >gi|86742577|ref|YP_482977.1| hemolysin III family channel protein [Frankia sp. CcI3]
88800368  9..210     |Reinekea blandensis MED297                    |B|Proteobacteria Gammaproteobacteria | >gi|88800368|ref|ZP_01115934.1| hemolysin III-like protein [Reinekea sp. MED297]
88802000  10..211    |Polaribacter irgensii 23-P                    |B|Bacteroidetes Flavobacteria        | >gi|88802000|ref|ZP_01117528.1| hypothetical protein PI23P_05037 [Polaribacter irgensii 23-P]
91793124  31..232    |Shewanella denitrificans OS217                |B|Proteobacteria Gammaproteobacteria | >gi|91793124|ref|YP_562775.1| hemolysin III family channel protein [Shewanella denitrificans OS217]
94967783  10..211    |Candidatus Koribacter versatilis Ellin345     |B|Acidobacteria Candidatus Koribacter.| >gi|94967783|ref|YP_589831.1| hemolysin III family channel protein [Candidatus Koribacter versatilis Ellin345]
94984656  9..210     |Deinococcus geothermalis DSM 11300            |B|Deinococcus-Thermus Deinococci     | >gi|94984656|ref|YP_604020.1| hemolysin III family channel protein [Deinococcus geothermalis DSM 11300]
95931224  11..212    |Desulfuromonas acetoxidans DSM 684            |B|Proteobacteria Deltaproteobacteria | >gi|95931224|ref|ZP_01313945.1| channel protein, hemolysin III family [Desulfuromonas acetoxidans DSM 684]
104783967 3..203     |Pseudomonas entomophila L48                   |B|Proteobacteria Gammaproteobacteria | >gi|104783967|ref|YP_610465.1| hemolysin III [Pseudomonas entomophila L48]
170719664 3..203     |Pseudomonas putida W619                       |B|Proteobacteria Gammaproteobacteria | >gi|170719664|ref|YP_001747352.1| hemolysin III family channel protein [Pseudomonas putida W619]
111225550 1..201     |Frankia alni ACN14a                           |B|Actinobacteria Actinobacteridae    | >gi|111225550|ref|YP_716344.1| hypothetical protein FRAAL6206 [Frankia alni ACN14a]
116873299 8..208     |Listeria welshimeri serovar 6b str. SLCC5334  |B|Firmicutes Bacillales              | >gi|116873299|ref|YP_850080.1| hemolysin III family protein [Listeria welshimeri serovar 6b str. SLCC5334]
117619951 8..208     |Aeromonas hydrophila subsp. hydrophila ATCC 7966|B|Proteobacteria Gammaproteobacteria | >gi|117619951|ref|YP_857967.1| hemolysin III [Aeromonas hydrophila subsp. hydrophila ATCC 7966]
119944886 5..205     |Psychromonas ingrahamii 37                    |B|Proteobacteria Gammaproteobacteria | >gi|119944886|ref|YP_942566.1| channel protein, hemolysin III family protein [Psychromonas ingrahamii 37]
120435100 12..212    |Gramella forsetii KT0803                      |B|Bacteroidetes Flavobacteria        | >gi|120435100|ref|YP_860786.1| hemolysin-3 family protein [Gramella forsetii KT0803]
120556045 12..212    |Marinobacter aquaeolei VT8                    |B|Proteobacteria Gammaproteobacteria | >gi|120556045|ref|YP_960396.1| hemolysin III family channel protein [Marinobacter aquaeolei VT8]
121635286 5..205     |Neisseria meningitidis FAM18                  |B|Proteobacteria Betaproteobacteria  | >gi|121635286|ref|YP_975531.1| putative haemolysin [Neisseria meningitidis FAM18]
15677495  5..205     |Neisseria meningitidis MC58                   |B|Proteobacteria Betaproteobacteria  | >gi|15677495|ref|NP_274651.1| putative hemolysin [Neisseria meningitidis MC58]
218768668 5..205     |Neisseria meningitidis Z2491                  |B|Proteobacteria Betaproteobacteria  | >gi|218768668|ref|YP_002343180.1| putative haemolysin [Neisseria meningitidis Z2491]
240014537 5..205     |Neisseria gonorrhoeae DGI18                   |B|Proteobacteria Betaproteobacteria  | >gi|240014537|ref|ZP_04721450.1| Hemolysin, putative [Neisseria gonorrhoeae DGI18]
240116055 5..205     |Neisseria gonorrhoeae PID18                   |B|Proteobacteria Betaproteobacteria  | >gi|240116055|ref|ZP_04730117.1| Hemolysin, putative [Neisseria gonorrhoeae PID18]
254669615 5..205     |Neisseria meningitidis alpha153               |B|Proteobacteria Betaproteobacteria  | >gi|254669615|emb|CBA03649.1| hemolysin III [Neisseria meningitidis alpha153]
261392131 5..205     |Neisseria meningitidis 8013                   |B|Proteobacteria Betaproteobacteria  | >gi|261392131|emb|CAX49631.1| putative hemolysin III (Hly-III) [Neisseria meningitidis 8013]
59801643  5..205     |Neisseria gonorrhoeae FA 1090                 |B|Proteobacteria Betaproteobacteria  | >gi|59801643|ref|YP_208355.1| hypothetical protein NGO1289 [Neisseria gonorrhoeae FA 1090]
161870490 2..182     |Neisseria meningitidis 053442                 |B|Proteobacteria Betaproteobacteria  | >gi|161870490|ref|YP_001599662.1| hemolysin, putative [Neisseria meningitidis 053442]
123472424 8..208     |Trichomonas vaginalis G3                      |E|Parabasalia Trichomonadida         | >gi|123472424|ref|XP_001319406.1| Haemolysin-III related family protein [Trichomonas vaginalis G3]
123504814 12..212    |Trichomonas vaginalis G3                      |E|Parabasalia Trichomonadida         | >gi|123504814|ref|XP_001328839.1| channel protein, hemolysin III family protein [Trichomonas vaginalis G3]
13473356  19..219    |Mesorhizobium loti MAFF303099                 |B|Proteobacteria Alphaproteobacteria | >gi|13473356|ref|NP_104923.1| hemolysin III, novel hemolytic factor [Mesorhizobium loti MAFF303099]
260467045 25..225    |Mesorhizobium opportunistum WSM2075           |B|Proteobacteria Alphaproteobacteria | >gi|260467045|ref|ZP_05813225.1| Hly-III family protein [Mesorhizobium opportunistum WSM2075]
145297880 8..208     |Aeromonas salmonicida subsp. salmonicida A449 |B|Proteobacteria Gammaproteobacteria | >gi|145297880|ref|YP_001140721.1| membrane protein [Aeromonas salmonicida subsp. salmonicida A449]
149179670 8..208     |Bacillus sp. SG-1                             |B|Firmicutes Bacillales              | >gi|149179670|ref|ZP_01858175.1| hemolysin III [Bacillus sp. SG-1]
149372772 5..205     |unidentified eubacterium SCB49                |B|Bacteroidetes environmental samples.| >gi|149372772|ref|ZP_01891793.1| hemolysin [unidentified eubacterium SCB49]
149376026 22..222    |Marinobacter algicola DG893                   |B|Proteobacteria Gammaproteobacteria | >gi|149376026|ref|ZP_01893792.1| hypothetical protein MDG893_03570 [Marinobacter algicola DG893]
152965072 40..240    |Kineococcus radiotolerans SRS30216            |B|Actinobacteria Actinobacteridae    | >gi|152965072|ref|YP_001360856.1| Hly-III family protein [Kineococcus radiotolerans SRS30216]
154249310 11..211    |Fervidobacterium nodosum Rt17-B1              |B|Thermotogae Thermotogales          | >gi|154249310|ref|YP_001410135.1| hemolysin III family channel protein [Fervidobacterium nodosum Rt17-B1]
157126886 162..362   |Aedes aegypti                                 |E|Metazoa Arthropoda                 | >gi|157126886|ref|XP_001660993.1| monocyte to macrophage differentiation protein [Aedes aegypti]
159045450 12..212    |Dinoroseobacter shibae DFL 12                 |B|Proteobacteria Alphaproteobacteria | >gi|159045450|ref|YP_001534244.1| hypothetical protein Dshi_2910 [Dinoroseobacter shibae DFL 12]
163788684 5..205     |Flavobacteriales bacterium ALC-1              |B|Bacteroidetes Flavobacteria        | >gi|163788684|ref|ZP_02183129.1| hypothetical protein FBALC1_10622 [Flavobacteriales bacterium ALC-1]
163791546 14..214    |Carnobacterium sp. AT7                        |B|Firmicutes Lactobacillales         | >gi|163791546|ref|ZP_02185950.1| hemolysin III [Carnobacterium sp. AT7]
16801044  8..208     |Listeria innocua Clip11262                    |B|Firmicutes Bacillales              | >gi|16801044|ref|NP_471312.1| hypothetical protein lin1978 [Listeria innocua Clip11262]
16803904  8..208     |Listeria monocytogenes EGD-e                  |B|Firmicutes Bacillales              | >gi|16803904|ref|NP_465389.1| hypothetical protein lmo1864 [Listeria monocytogenes EGD-e]
46908096  8..208     |Listeria monocytogenes serotype 4b str. F2365 |B|Firmicutes Bacillales              | >gi|46908096|ref|YP_014485.1| hemolysin III [Listeria monocytogenes serotype 4b str. F2365]
224500094 8..201     |Listeria monocytogenes Finland 1988           |B|Firmicutes Bacillales              | >gi|224500094|ref|ZP_03668443.1| hemolysin-3 (Hemolysin III) (Hly-III) [Listeria monocytogenes Finland 1988]
255022350 1..100     |Listeria monocytogenes FSL J1-208             |B|Firmicutes Bacillales              | >gi|255022350|ref|ZP_05294336.1| hemolysin III [Listeria monocytogenes FSL J1-208]
169632477 10..210    |Acinetobacter baumannii SDF                   |B|Proteobacteria Gammaproteobacteria | >gi|169632477|ref|YP_001706213.1| hemolysin III (HLY-III) [Acinetobacter baumannii SDF]
169794800 10..210    |Acinetobacter baumannii AYE                   |B|Proteobacteria Gammaproteobacteria | >gi|169794800|ref|YP_001712593.1| hemolysin III (HLY-III) [Acinetobacter baumannii AYE]
184159427 10..210    |Acinetobacter baumannii ACICU                 |B|Proteobacteria Gammaproteobacteria | >gi|184159427|ref|YP_001847766.1| hemolysin III [Acinetobacter baumannii ACICU]
213157873 6..206     |Acinetobacter baumannii AB0057                |B|Proteobacteria Gammaproteobacteria | >gi|213157873|ref|YP_002320671.1| hemolysin-3 [Acinetobacter baumannii AB0057]
239501728 10..210    |Acinetobacter baumannii AB900                 |B|Proteobacteria Gammaproteobacteria | >gi|239501728|ref|ZP_04661038.1| hemolysin III [Acinetobacter baumannii AB900]
260548838 10..210    |Acinetobacter sp. RUH2624                     |B|Proteobacteria Gammaproteobacteria | >gi|260548838|ref|ZP_05823060.1| hemolysin III [Acinetobacter sp. RUH2624]
294841622 6..206     |Acinetobacter baumannii 6014059               |B|Proteobacteria Gammaproteobacteria | >gi|294841622|ref|ZP_06786305.1| channel protein, hemolysin III family [Acinetobacter sp. 6014059]
126642883 1..184     |Acinetobacter baumannii ATCC 17978            |B|Proteobacteria Gammaproteobacteria | >gi|126642883|ref|YP_001085867.1| putative hemolysin III (HLY-III) [Acinetobacter baumannii ATCC 17978]
171743229 88..288    |Bifidobacterium dentium ATCC 27678            |B|Actinobacteria Actinobacteridae    | >gi|171743229|ref|ZP_02919036.1| hypothetical protein BIFDEN_02357 [Bifidobacterium dentium ATCC 27678]
183601465 85..285    |Bifidobacterium animalis subsp. lactis HN019  |B|Actinobacteria Actinobacteridae    | >gi|183601465|ref|ZP_02962835.1| hypothetical protein BIFLAC_02377 [Bifidobacterium animalis subsp. lactis HN019]
289178778 93..293    |Bifidobacterium animalis subsp. lactis BB-12  |B|Actinobacteria Actinobacteridae    | >gi|289178778|gb|ADC86024.1| Conserved membrane protein (hemolysin III-like protein) [Bifidobacterium animalis subsp. lactis BB-12]
198284096 22..222    |Acidithiobacillus ferrooxidans ATCC 53993     |B|Proteobacteria Gammaproteobacteria | >gi|198284096|ref|YP_002220417.1| hemolysin III family channel protein [Acidithiobacillus ferrooxidans ATCC 53993]
218666490 14..214    |Acidithiobacillus ferrooxidans ATCC 23270     |B|Proteobacteria Gammaproteobacteria | >gi|218666490|ref|YP_002426750.1| channel protein, hemolysin III family [Acidithiobacillus ferrooxidans ATCC 23270]
212716147 88..288    |Bifidobacterium catenulatum DSM 16992         |B|Actinobacteria Actinobacteridae    | >gi|212716147|ref|ZP_03324275.1| hypothetical protein BIFCAT_01062 [Bifidobacterium catenulatum DSM 16992]
225351852 88..288    |Bifidobacterium pseudocatenulatum DSM 20438   |B|Actinobacteria Actinobacteridae    | >gi|225351852|ref|ZP_03742875.1| hypothetical protein BIFPSEUDO_03454 [Bifidobacterium pseudocatenulatum DSM 20438]
223998364 2..202     |Thalassiosira pseudonana CCMP1335             |E|stramenopiles Bacillariophyta      | >gi|223998364|ref|XP_002288855.1| channel protein of hemolysin III family [Thalassiosira pseudonana CCMP1335]
224014818 583..783   |Thalassiosira pseudonana CCMP1335             |E|stramenopiles Bacillariophyta      | >gi|224014818|ref|XP_002297071.1| predicted protein [Thalassiosira pseudonana CCMP1335]
227895955 10..210    |Lactobacillus plantarum subsp. plantarum ATCC 14917|B|Firmicutes Lactobacillales         | >gi|227895955|ref|ZP_04013760.1| hemolysin III [Lactobacillus plantarum subsp. plantarum ATCC 14917]
28379651  13..213    |Lactobacillus plantarum WCFS1                 |B|Firmicutes Lactobacillales         | >gi|28379651|ref|NP_786543.1| hemolysin III [Lactobacillus plantarum WCFS1]
229161219 12..212    |Bacillus cereus R309803                       |B|Firmicutes Bacillales              | >gi|229161219|ref|ZP_04289206.1| Hemolysin-3 [Bacillus cereus R309803]
228972254 31..230    |Bacillus thuringiensis serovar thuringiensis str. T01001|B|Firmicutes Bacillales              | >gi|228972254|ref|ZP_04132867.1| Hemolysin-3 [Bacillus thuringiensis serovar thuringiensis str. T01001]
228991234 31..230    |Bacillus pseudomycoides DSM 12442             |B|Firmicutes Bacillales              | >gi|228991234|ref|ZP_04151192.1| Hemolysin-3 [Bacillus pseudomycoides DSM 12442]
229115720 31..230    |Bacillus cereus Rock1-3                       |B|Firmicutes Bacillales              | >gi|229115720|ref|ZP_04245123.1| Hemolysin-3 [Bacillus cereus Rock1-3]
229127640 31..230    |Bacillus cereus BDRD-Cer4                     |B|Firmicutes Bacillales              | >gi|229127640|ref|ZP_04256629.1| Hemolysin-3 [Bacillus cereus BDRD-Cer4]
65319548  31..230    |Bacillus anthracis str. A2012                 |B|Firmicutes Bacillales              | >gi|65319548|ref|ZP_00392507.1| COG1272: Predicted membrane protein, hemolysin III homolog [Bacillus anthracis str. A2012]
118477673 34..232    |Bacillus thuringiensis str. Al Hakam          |B|Firmicutes Bacillales              | >gi|118477673|ref|YP_894824.1| hemolysin III [Bacillus thuringiensis str. Al Hakam]
152975431 14..212    |Bacillus cytotoxicus NVH 391-98               |B|Firmicutes Bacillales              | >gi|152975431|ref|YP_001374948.1| hemolysin III family channel protein [Bacillus cereus subsp. cytotoxis NVH 391-98]
163940035 14..212    |Bacillus weihenstephanensis KBAB4             |B|Firmicutes Bacillales              | >gi|163940035|ref|YP_001644919.1| hemolysin III family channel protein [Bacillus weihenstephanensis KBAB4]
1708219   14..212    |Bacillus cereus                               |B|Firmicutes Bacillales              | >gi|1708219|sp|P54176.1|HLY3_BACCE RecName: Full=Hemolysin-3; AltName: Full=Hemolysin III; Short=Hly-III
206978245 14..212    |Bacillus cereus H3081.97                      |B|Firmicutes Bacillales              | >gi|206978245|ref|ZP_03239124.1| hemolysin III [Bacillus cereus H3081.97]
218235560 14..212    |Bacillus cereus B4264                         |B|Firmicutes Bacillales              | >gi|218235560|ref|YP_002366941.1| hemolysin III [Bacillus cereus B4264]
22086526  14..212    |Bacillus cereus                               |B|Firmicutes Bacillales              | >gi|22086526|gb|AAM90670.1|AF401361_1 hemolysin HlyIII [Bacillus cereus]
222095839 14..212    |Bacillus cereus Q1                            |B|Firmicutes Bacillales              | >gi|222095839|ref|YP_002529896.1| hemolysin iii [Bacillus cereus Q1]
228920954 14..212    |Bacillus thuringiensis serovar huazhongensis BGSC 4BD1|B|Firmicutes Bacillales              | >gi|228920954|ref|ZP_04084291.1| Hemolysin-3 [Bacillus thuringiensis serovar huazhongensis BGSC 4BD1]
228952583 34..232    |Bacillus thuringiensis serovar kurstaki str. T03a001|B|Firmicutes Bacillales              | >gi|228952583|ref|ZP_04114659.1| Hemolysin-3 [Bacillus thuringiensis serovar kurstaki str. T03a001]
228978868 34..232    |Bacillus thuringiensis Bt407                  |B|Firmicutes Bacillales              | >gi|228978868|ref|ZP_04139235.1| Hemolysin-3 [Bacillus thuringiensis Bt407]
229017555 14..212    |Bacillus cereus AH1273                        |B|Firmicutes Bacillales              | >gi|229017555|ref|ZP_04174454.1| Hemolysin-3 [Bacillus cereus AH1273]
229059915 20..218    |Bacillus cereus AH603                         |B|Firmicutes Bacillales              | >gi|229059915|ref|ZP_04197289.1| Hemolysin-3 [Bacillus cereus AH603]
229085192 14..212    |Bacillus cereus Rock3-44                      |B|Firmicutes Bacillales              | >gi|229085192|ref|ZP_04217436.1| Hemolysin-3 [Bacillus cereus Rock3-44]
229091234 34..232    |Bacillus cereus Rock3-42                      |B|Firmicutes Bacillales              | >gi|229091234|ref|ZP_04222453.1| Hemolysin-3 [Bacillus cereus Rock3-42]
229155828 34..232    |Bacillus cereus ATCC 4342                     |B|Firmicutes Bacillales              | >gi|229155828|ref|ZP_04283930.1| Hemolysin-3 [Bacillus cereus ATCC 4342]
296502809 22..220    |Bacillus thuringiensis BMB171                 |B|Firmicutes Bacillales              | >gi|296502809|ref|YP_003664509.1| hemolysin III [Bacillus thuringiensis BMB171]
30020330  10..208    |Bacillus cereus ATCC 14579                    |B|Firmicutes Bacillales              | >gi|30020330|ref|NP_831961.1| hemolysin III [Bacillus cereus ATCC 14579]
30262255  14..212    |Bacillus anthracis str. Ames                  |B|Firmicutes Bacillales              | >gi|30262255|ref|NP_844632.1| hemolysin III [Bacillus anthracis str. Ames]
42781335  34..232    |Bacillus cereus ATCC 10987                    |B|Firmicutes Bacillales              | >gi|42781335|ref|NP_978582.1| hemolysin III [Bacillus cereus ATCC 10987]
47570570  14..212    |Bacillus cereus G9241                         |B|Firmicutes Bacillales              | >gi|47570570|ref|ZP_00241190.1| hemolysin III [Bacillus cereus G9241]
229817906 98..298    |Bifidobacterium angulatum DSM 20098           |B|Actinobacteria Actinobacteridae    | >gi|229817906|ref|ZP_04448188.1| hypothetical protein BIFANG_03192 [Bifidobacterium angulatum DSM 20098]
237736207 6..206     |Fusobacterium mortiferum ATCC 9817            |B|Fusobacteria Fusobacteriales       | >gi|237736207|ref|ZP_04566688.1| hemolysin III family channel protein [Fusobacterium mortiferum ATCC 9817]
241894969 13..213    |Weissella paramesenteroides ATCC 33313        |B|Firmicutes Lactobacillales         | >gi|241894969|ref|ZP_04782265.1| Hly III family protein [Weissella paramesenteroides ATCC 33313]
253582339 8..208     |Fusobacterium varium ATCC 27725               |B|Fusobacteria Fusobacteriales       | >gi|253582339|ref|ZP_04859562.1| membrane protein [Fusobacterium varium ATCC 27725]
254381980 21..221    |Streptomyces sp. Mg1                          |B|Actinobacteria Actinobacteridae    | >gi|254381980|ref|ZP_04997343.1| integral membrane protein [Streptomyces sp. Mg1]
254419429 26..226    |Brevundimonas sp. BAL3                        |B|Proteobacteria Alphaproteobacteria | >gi|254419429|ref|ZP_05033153.1| Haemolysin-III related subfamily [Brevundimonas sp. BAL3]
254441766 46..246    |Octadecabacter antarcticus 307                |B|Proteobacteria Alphaproteobacteria | >gi|254441766|ref|ZP_05055259.1| Haemolysin-III related subfamily [Octadecabacter antarcticus 307]
254451043 28..228    |Octadecabacter antarcticus 238                |B|Proteobacteria Alphaproteobacteria | >gi|254451043|ref|ZP_05064480.1| hemolysin [Octadecabacter antarcticus 238]
254515065 5..205     |gamma proteobacterium NOR5-3                  |B|Proteobacteria Gammaproteobacteria.| >gi|254515065|ref|ZP_05127126.1| channel protein, hemolysin III family protein [gamma proteobacterium NOR5-3]
255262776 7..207     |Thalassiobium sp. R2A62                       |B|Proteobacteria Alphaproteobacteria | >gi|255262776|ref|ZP_05342118.1| hemolysin-3 [Thalassiobium sp. R2A62]
256769455 30..230    |Streptomyces sp. C                            |B|Actinobacteria Actinobacteridae    | >gi|256769455|ref|ZP_05508629.1| hypothetical protein StreC_23156 [Streptomyces sp. C]
256826579 54..254    |Cryptobacterium curtum DSM 15641              |B|Actinobacteria Coriobacteridae     | >gi|256826579|ref|YP_003150538.1| channel protein, hemolysin III family [Cryptobacterium curtum DSM 15641]
256829511 9..209     |Desulfomicrobium baculatum DSM 4028           |B|Proteobacteria Deltaproteobacteria | >gi|256829511|ref|YP_003158239.1| channel protein, hemolysin III family [Desulfomicrobium baculatum DSM 4028]
258405703 13..213    |Desulfohalobium retbaense DSM 5692            |B|Proteobacteria Deltaproteobacteria | >gi|258405703|ref|YP_003198445.1| channel protein, hemolysin III family [Desulfohalobium retbaense DSM 5692]
260430638 15..215    |Silicibacter lacuscaerulensis ITI-1157        |B|Proteobacteria Alphaproteobacteria | >gi|260430638|ref|ZP_05784610.1| Hly-III family protein [Silicibacter lacuscaerulensis ITI-1157]
260774559 24..224    |Vibrio metschnikovii CIP 69.14                |B|Proteobacteria Gammaproteobacteria | >gi|260774559|ref|ZP_05883472.1| hypothetical protein VIB_003040 [Vibrio metschnikovii CIP 69.14]
261408025 9..209     |Paenibacillus sp. Y412MC10                    |B|Firmicutes Bacillales              | >gi|261408025|ref|YP_003244266.1| hemolysin III family channel protein [Paenibacillus sp. Y412MC10]
262281120 10..210    |Acinetobacter calcoaceticus RUH2202           |B|Proteobacteria Gammaproteobacteria | >gi|262281120|ref|ZP_06058902.1| hemolysin III [Acinetobacter calcoaceticus RUH2202]
298698923 10..210    |Acinetobacter sp. DR1                         |B|Proteobacteria Gammaproteobacteria | >gi|298698923|gb|ADI89488.1| channel protein, hemolysin III family [Acinetobacter sp. DR1]
293611059 11..210    |Acinetobacter sp. SH024                       |B|Proteobacteria Gammaproteobacteria | >gi|293611059|ref|ZP_06693358.1| conserved hypothetical protein [Acinetobacter sp. SH024]
262369211 11..211    |Acinetobacter johnsonii SH046                 |B|Proteobacteria Gammaproteobacteria | >gi|262369211|ref|ZP_06062539.1| conserved hypothetical protein [Acinetobacter johnsonii SH046]
262371729 10..210    |Acinetobacter junii SH205                     |B|Proteobacteria Gammaproteobacteria | >gi|262371729|ref|ZP_06065008.1| hemolysin III [Acinetobacter junii SH205]
269120163 9..209     |Sebaldella termitidis ATCC 33386              |B|Fusobacteria Fusobacteriales       | >gi|269120163|ref|YP_003308340.1| channel protein, hemolysin III family [Sebaldella termitidis ATCC 33386]
269216474 6..206     |Slackia exigua ATCC 700122                    |B|Actinobacteria Coriobacteridae     | >gi|269216474|ref|ZP_06160328.1| hemolysin III [Slackia exigua ATCC 700122]
269794156 56..256    |Sanguibacter keddieii DSM 10542               |B|Actinobacteria Actinobacteridae    | >gi|269794156|ref|YP_003313611.1| hypothetical protein Sked_08260 [Sanguibacter keddieii DSM 10542]
269924404 26..226    |Brevundimonas subvibrioides ATCC 15264        |B|Proteobacteria Alphaproteobacteria | >gi|269924404|ref|ZP_06173292.1| Hly-III family protein [Brevundimonas subvibrioides ATCC 15264]
269957413 19..219    |Xylanimonas cellulosilytica DSM 15894         |B|Actinobacteria Actinobacteridae    | >gi|269957413|ref|YP_003327202.1| Hly-III family protein [Xylanimonas cellulosilytica DSM 15894]
271968900 18..218    |Streptosporangium roseum DSM 43021            |B|Actinobacteria Actinobacteridae    | >gi|271968900|ref|YP_003343096.1| hemolysin III family channel protein [Streptosporangium roseum DSM 43021]
283768296 14..214    |Bulleidia extructa W1219                      |B|Firmicutes Erysipelotrichi         | >gi|283768296|ref|ZP_06341208.1| channel protein, hemolysin III family protein [Bulleidia extructa W1219]
283847329 8..208     |Bacillus cellulosilyticus DSM 2522            |B|Firmicutes Bacillales              | >gi|283847329|ref|ZP_06364775.1| channel protein, hemolysin III family [Bacillus cellulosilyticus DSM 2522]
289435207 8..208     |Listeria seeligeri serovar 1/2b str. SLCC3954 |B|Firmicutes Bacillales              | >gi|289435207|ref|YP_003465079.1| hemolysin III [Listeria seeligeri serovar 1/2b str. SLCC3954]
289643491 1..201     |Frankia symbiont of Datisca glomerata         |B|Actinobacteria Actinobacteridae    | >gi|289643491|ref|ZP_06475609.1| channel protein, hemolysin III family [Frankia symbiont of Datisca glomerata]
291452627 56..256    |Streptomyces albus J1074                      |B|Actinobacteria Actinobacteridae    | >gi|291452627|ref|ZP_06592017.1| integral membrane protein [Streptomyces albus J1074]
239980770 2..196     |Streptomyces albus J1074                      |B|Actinobacteria Actinobacteridae    | >gi|239980770|ref|ZP_04703294.1| hypothetical protein SalbJ_15097 [Streptomyces albus J1074]
291618736 22..222    |Pantoea ananatis LMG 20103                    |B|Proteobacteria Gammaproteobacteria | >gi|291618736|ref|YP_003521478.1| YqfA [Pantoea ananatis LMG 20103]
294786773 103..303   |Parascardovia denticolens F0305               |B|Actinobacteria Actinobacteridae    | >gi|294786773|ref|ZP_06752027.1| membrane protein, hemolysin III-like protein [Parascardovia denticolens F0305]
295400395 4..204     |Geobacillus thermoglucosidasius C56-YS93      |B|Firmicutes Bacillales              | >gi|295400395|ref|ZP_06810374.1| channel protein, hemolysin III family [Geobacillus thermoglucosidasius C56-YS93]
255331842 4..177     |Geobacillus sp. Y4.1MC1                       |B|Firmicutes Bacillales              | >gi|255331842|ref|ZP_05372856.1| channel protein, hemolysin III family [Geobacillus sp. Y4.1MC1]
296126247 19..219    |Brachyspira murdochii DSM 12563               |B|Spirochaetes Spirochaetales        | >gi|296126247|ref|YP_003633499.1| Hly-III family protein [Brachyspira murdochii DSM 12563]
297559721 62..262    |Nocardiopsis dassonvillei subsp. dassonvillei DSM 43111|B|Actinobacteria Actinobacteridae    | >gi|297559721|ref|YP_003678695.1| channel protein, hemolysin III family [Nocardiopsis dassonvillei subsp. dassonvillei DSM 43111]
297572050 16..216    |Arcanobacterium haemolyticum DSM 20595        |B|Actinobacteria Actinobacteridae    | >gi|297572050|ref|YP_003697824.1| channel protein, hemolysin III family [Arcanobacterium haemolyticum DSM 20595]
297582948 7..207     |Bacillus selenitireducens MLS10               |B|Firmicutes Bacillales              | >gi|297582948|ref|YP_003698728.1| hemolysin III family channel protein [Bacillus selenitireducens MLS10]
29826862  63..263    |Streptomyces avermitilis MA-4680              |B|Actinobacteria Actinobacteridae    | >gi|29826862|ref|NP_821496.1| hypothetical protein SAV_322 [Streptomyces avermitilis MA-4680]
299144405 14..214    |Peptoniphilus sp. oral taxon 386 str. F0131   |B|Firmicutes Clostridia              | >gi|299144405|ref|ZP_07037485.1| hemolysin III [Peptoniphilus sp. oral taxon 386 str. F0131]
38234489  55..255    |Corynebacterium diphtheriae NCTC 13129        |B|Actinobacteria Actinobacteridae    | >gi|38234489|ref|NP_940256.1| hypothetical protein DIP1922 [Corynebacterium diphtheriae NCTC 13129]
78485420  5..205     |Thiomicrospira crunogena XCL-2                |B|Proteobacteria Gammaproteobacteria | >gi|78485420|ref|YP_391345.1| hemolysin III family channel protein [Thiomicrospira crunogena XCL-2]
83647492  10..210    |Hahella chejuensis KCTC 2396                  |B|Proteobacteria Gammaproteobacteria | >gi|83647492|ref|YP_435927.1| hypothetical protein HCH_04808 [Hahella chejuensis KCTC 2396]
84684898  9..209     |Maritimibacter alkaliphilus HTCC2654          |B|Proteobacteria Alphaproteobacteria | >gi|84684898|ref|ZP_01012798.1| hemolysin III-like protein [Maritimibacter alkaliphilus HTCC2654]
86130276  12..212    |Dokdonia donghaensis MED134                   |B|Bacteroidetes Flavobacteria        | >gi|86130276|ref|ZP_01048876.1| hemolysin-III related protein [Dokdonia donghaensis MED134]
86135134  10..210    |Polaribacter sp. MED152                       |B|Bacteroidetes Flavobacteria        | >gi|86135134|ref|ZP_01053716.1| hemolysin-III related protein [Polaribacter sp. MED152]
89890352  6..206     |Flavobacteria bacterium BBFL7                 |B|Bacteroidetes Flavobacteria.       | >gi|89890352|ref|ZP_01201862.1| hemolysin III [Flavobacteria bacterium BBFL7]
91216345  6..206     |Psychroflexus torquis ATCC 700755             |B|Bacteroidetes Flavobacteria        | >gi|91216345|ref|ZP_01253312.1| channel protein, hemolysin III family subfamily [Psychroflexus torquis ATCC 700755]
107027636 5..204     |Burkholderia cenocepacia AU 1054              |B|Proteobacteria Betaproteobacteria  | >gi|107027636|ref|YP_625147.1| hemolysin III family channel protein [Burkholderia cenocepacia AU 1054]
206564582 5..204     |Burkholderia cenocepacia J2315                |B|Proteobacteria Betaproteobacteria  | >gi|206564582|ref|YP_002235345.1| haemolysin-III related protein [Burkholderia cenocepacia J2315]
78060952  5..204     |Burkholderia sp. 383                          |B|Proteobacteria Betaproteobacteria  | >gi|78060952|ref|YP_370860.1| hemolysin HylII family protein [Burkholderia sp. 383]
108759382 51..250    |Myxococcus xanthus DK 1622                    |B|Proteobacteria Deltaproteobacteria | >gi|108759382|ref|YP_631359.1| hemolysin III family channel protein [Myxococcus xanthus DK 1622]
109899896 15..214    |Pseudoalteromonas atlantica T6c               |B|Proteobacteria Gammaproteobacteria | >gi|109899896|ref|YP_663151.1| hemolysin III family channel protein [Pseudoalteromonas atlantica T6c]
114563045 25..224    |Shewanella frigidimarina NCIMB 400            |B|Proteobacteria Gammaproteobacteria | >gi|114563045|ref|YP_750558.1| channel protein, hemolysin III family protein [Shewanella frigidimarina NCIMB 400]
115359628 5..204     |Burkholderia ambifaria AMMD                   |B|Proteobacteria Betaproteobacteria  | >gi|115359628|ref|YP_776766.1| hemolysin III family channel protein [Burkholderia ambifaria AMMD]
134292435 5..204     |Burkholderia vietnamiensis G4                 |B|Proteobacteria Betaproteobacteria  | >gi|134292435|ref|YP_001116171.1| hemolysin III family channel protein [Burkholderia vietnamiensis G4]
170697873 5..203     |Burkholderia ambifaria IOP40-10               |B|Proteobacteria Betaproteobacteria  | >gi|170697873|ref|ZP_02888958.1| channel protein, hemolysin III family [Burkholderia ambifaria IOP40-10]
116251135 16..215    |Rhizobium leguminosarum bv. viciae 3841       |B|Proteobacteria Alphaproteobacteria | >gi|116251135|ref|YP_766973.1| hemolysin-related transmembrane protein [Rhizobium leguminosarum bv. viciae 3841]
209548470 13..212    |Rhizobium leguminosarum bv. trifolii WSM2304  |B|Proteobacteria Alphaproteobacteria | >gi|209548470|ref|YP_002280387.1| Hly-III family protein [Rhizobium leguminosarum bv. trifolii WSM2304]
241203733 13..212    |Rhizobium leguminosarum bv. trifolii WSM1325  |B|Proteobacteria Alphaproteobacteria | >gi|241203733|ref|YP_002974829.1| Hly-III family protein [Rhizobium leguminosarum bv. trifolii WSM1325]
118587651 42..241    |Labrenzia aggregata IAM 12614                 |B|Proteobacteria Alphaproteobacteria | >gi|118587651|ref|ZP_01545061.1| Hly-III related proteins [Stappia aggregata IAM 12614]
119713104 10..209    |uncultured marine bacterium EB0_49D07         |B|environmental samples.             | >gi|119713104|gb|ABL97173.1| putative channel protein hemolysin III family protein [uncultured marine bacterium EB0_49D07]
119964465 10..209    |Arthrobacter aurescens TC1                    |B|Actinobacteria Actinobacteridae    | >gi|119964465|ref|YP_947073.1| membrane protein, hemolysin III-like protein [Arthrobacter aurescens TC1]
121606259 3..202     |Polaromonas naphthalenivorans CJ2             |B|Proteobacteria Betaproteobacteria  | >gi|121606259|ref|YP_983588.1| hemolysin III family channel protein [Polaromonas naphthalenivorans CJ2]
121606440 12..211    |Polaromonas naphthalenivorans CJ2             |B|Proteobacteria Betaproteobacteria  | >gi|121606440|ref|YP_983769.1| hemolysin III family channel protein [Polaromonas naphthalenivorans CJ2]
146280891 3..202     |Pseudomonas stutzeri A1501                    |B|Proteobacteria Gammaproteobacteria | >gi|146280891|ref|YP_001171044.1| hemolysin III family channel protein [Pseudomonas stutzeri A1501]
148273421 44..243    |Clavibacter michiganensis subsp. michiganensis NCPPB 382|B|Actinobacteria Actinobacteridae    | >gi|148273421|ref|YP_001222982.1| HlyIII family membrane protein [Clavibacter michiganensis subsp. michiganensis NCPPB 382]
170782748 44..243    |Clavibacter michiganensis subsp. sepedonicus  |B|Actinobacteria Actinobacteridae    | >gi|170782748|ref|YP_001711082.1| putative integral membrane protein [Clavibacter michiganensis subsp. sepedonicus]
150020000 8..207     |Thermosipho melanesiensis BI429               |B|Thermotogae Thermotogales          | >gi|150020000|ref|YP_001305354.1| hemolysin III family channel protein [Thermosipho melanesiensis BI429]
150395829 12..211    |Sinorhizobium medicae WSM419                  |B|Proteobacteria Alphaproteobacteria | >gi|150395829|ref|YP_001326296.1| Hly-III family protein [Sinorhizobium medicae WSM419]
152983522 3..202     |Pseudomonas aeruginosa PA7                    |B|Proteobacteria Gammaproteobacteria | >gi|152983522|ref|YP_001350873.1| putative lipoprotein [Pseudomonas aeruginosa PA7]
15600026  3..202     |Pseudomonas aeruginosa PAO1                   |B|Proteobacteria Gammaproteobacteria | >gi|15600026|ref|NP_253520.1| hypothetical protein PA4833 [Pseudomonas aeruginosa PAO1]
218893927 3..202     |Pseudomonas aeruginosa LESB58                 |B|Proteobacteria Gammaproteobacteria | >gi|218893927|ref|YP_002442796.1| putative hemolyin III [Pseudomonas aeruginosa LESB58]
49079288  3..202     |synthetic construct                           |?|artificial sequences.              | >gi|49079288|gb|AAT49876.1| PA4833 [synthetic construct]
154507711 23..222    |Actinomyces odontolyticus ATCC 17982          |B|Actinobacteria Actinobacteridae    | >gi|154507711|ref|ZP_02043353.1| hypothetical protein ACTODO_00192 [Actinomyces odontolyticus ATCC 17982]
293189384 23..222    |Actinomyces odontolyticus F0309               |B|Actinobacteria Actinobacteridae    | >gi|293189384|ref|ZP_06608107.1| hemolysin III [Actinomyces odontolyticus F0309]
15964742  12..211    |Sinorhizobium meliloti 1021                   |B|Proteobacteria Alphaproteobacteria | >gi|15964742|ref|NP_385095.1| hypothetical protein SMc00455 [Sinorhizobium meliloti 1021]
160932739 22..221    |Clostridium leptum DSM 753                    |B|Firmicutes Clostridia              | >gi|160932739|ref|ZP_02080128.1| hypothetical protein CLOLEP_01580 [Clostridium leptum DSM 753]
161519796 5..204     |Burkholderia multivorans ATCC 17616           |B|Proteobacteria Betaproteobacteria  | >gi|161519796|ref|YP_001583223.1| hemolysin III family channel protein [Burkholderia multivorans ATCC 17616]
221200630 5..204     |Burkholderia multivorans CGD2M                |B|Proteobacteria Betaproteobacteria  | >gi|221200630|ref|ZP_03573671.1| hemolysin III [Burkholderia multivorans CGD2M]
221210413 5..204     |Burkholderia multivorans CGD1                 |B|Proteobacteria Betaproteobacteria  | >gi|221210413|ref|ZP_03583393.1| hemolysin III [Burkholderia multivorans CGD1]
163816112 49..248    |Coprococcus eutactus ATCC 27759               |B|Firmicutes Clostridia              | >gi|163816112|ref|ZP_02207480.1| hypothetical protein COPEUT_02296 [Coprococcus eutactus ATCC 27759]
238925727 49..248    |Eubacterium rectale ATCC 33656                |B|Firmicutes Clostridia              | >gi|238925727|ref|YP_002939244.1| putative membrane protein, hemolysin IIIrelated protein [Eubacterium rectale ATCC 33656]
291527885 49..248    |Eubacterium rectale M104/1                    |B|Firmicutes Clostridia              | >gi|291527885|emb|CBK93471.1| Predicted membrane protein, hemolysin III homolog [Eubacterium rectale M104/1]
295093423 49..248    |Coprococcus sp. ART55/1                       |B|Firmicutes Clostridia              | >gi|295093423|emb|CBK82514.1| Predicted membrane protein, hemolysin III homolog [Coprococcus sp. ART55/1]
163841777 26..225    |Renibacterium salmoninarum ATCC 33209         |B|Actinobacteria Actinobacteridae    | >gi|163841777|ref|YP_001626182.1| membrane protein (hemolysin III-like protein) [Renibacterium salmoninarum ATCC 33209]
163848926 11..210    |Chloroflexus aurantiacus J-10-fl              |B|Chloroflexi Chloroflexales         | >gi|163848926|ref|YP_001636970.1| hemolysin III family channel protein [Chloroflexus aurantiacus J-10-fl]
167588978 5..204     |Burkholderia ubonensis Bu                     |B|Proteobacteria Betaproteobacteria  | >gi|167588978|ref|ZP_02381366.1| channel protein, hemolysin III family [Burkholderia ubonensis Bu]
167623828 30..229    |Shewanella halifaxensis HAW-EB4               |B|Proteobacteria Gammaproteobacteria | >gi|167623828|ref|YP_001674122.1| hemolysin III family channel protein [Shewanella halifaxensis HAW-EB4]
170065195 153..352   |Culex quinquefasciatus                        |E|Metazoa Arthropoda                 | >gi|170065195|ref|XP_001867839.1| monocyte to macrophage differentiation factor 2 [Culex quinquefasciatus]
190575497 8..207     |Stenotrophomonas maltophilia K279a            |B|Proteobacteria Gammaproteobacteria | >gi|190575497|ref|YP_001973342.1| putative transmembrane hemolysin protein [Stenotrophomonas maltophilia K279a]
194366826 10..209    |Stenotrophomonas maltophilia R551-3           |B|Proteobacteria Gammaproteobacteria | >gi|194366826|ref|YP_002029436.1| hemolysin III family channel protein [Stenotrophomonas maltophilia R551-3]
190890938 13..212    |Rhizobium etli CIAT 652                       |B|Proteobacteria Alphaproteobacteria | >gi|190890938|ref|YP_001977480.1| hemolysin III protein [Rhizobium etli CIAT 652]
218459980 26..225    |Rhizobium etli Kim 5                          |B|Proteobacteria Alphaproteobacteria | >gi|218459980|ref|ZP_03500071.1| putative hemolysin III protein [Rhizobium etli Kim 5]
218514645 13..212    |Rhizobium etli 8C-3                           |B|Proteobacteria Alphaproteobacteria | >gi|218514645|ref|ZP_03511485.1| putative hemolysin III protein [Rhizobium etli 8C-3]
86356874  13..212    |Rhizobium etli CFN 42                         |B|Proteobacteria Alphaproteobacteria | >gi|86356874|ref|YP_468766.1| hemolysin III protein [Rhizobium etli CFN 42]
218662819 16..210    |Rhizobium etli IE4771                         |B|Proteobacteria Alphaproteobacteria | >gi|218662819|ref|ZP_03518749.1| putative hemolysin III protein [Rhizobium etli IE4771]
218673908 6..146     |Rhizobium etli GR56                           |B|Proteobacteria Alphaproteobacteria | >gi|218673908|ref|ZP_03523577.1| putative hemolysin III protein [Rhizobium etli GR56]
196157942 17..216    |Alteromonas macleodii str. 'Deep ecotype'     |B|Proteobacteria Gammaproteobacteria | >gi|196157942|ref|YP_002127431.1| putative hemolysin III [Alteromonas macleodii 'Deep ecotype']
239992997 23..222    |Alteromonas macleodii ATCC 27126              |B|Proteobacteria Gammaproteobacteria | >gi|239992997|ref|ZP_04713521.1| putative hemolysin III [Alteromonas macleodii ATCC 27126]
209884206 27..226    |Oligotropha carboxidovorans OM5               |B|Proteobacteria Alphaproteobacteria | >gi|209884206|ref|YP_002288063.1| hemolysin-3 (Hemolysin III) (Hly-III) [Oligotropha carboxidovorans OM5]
219847711 11..210    |Chloroflexus aggregans DSM 9485               |B|Chloroflexi Chloroflexales         | >gi|219847711|ref|YP_002462144.1| channel protein, hemolysin III family [Chloroflexus aggregans DSM 9485]
221485987 89..288    |Toxoplasma gondii GT1                         |E|Alveolata Apicomplexa              | >gi|221485987|gb|EEE24257.1| hemolysin, putative [Toxoplasma gondii GT1]
237834855 89..288    |Toxoplasma gondii ME49                        |E|Alveolata Apicomplexa              | >gi|237834855|ref|XP_002366725.1| hemolysin, putative [Toxoplasma gondii ME49]
221639959 12..211    |Rhodobacter sphaeroides KD131                 |B|Proteobacteria Alphaproteobacteria | >gi|221639959|ref|YP_002526221.1| Hly-III family protein [Rhodobacter sphaeroides KD131]
77464094  12..211    |Rhodobacter sphaeroides 2.4.1                 |B|Proteobacteria Alphaproteobacteria | >gi|77464094|ref|YP_353598.1| hypothetical protein RSP_0525 [Rhodobacter sphaeroides 2.4.1]
222085287 13..212    |Agrobacterium radiobacter K84                 |B|Proteobacteria Alphaproteobacteria | >gi|222085287|ref|YP_002543817.1| hemolysin III protein [Agrobacterium radiobacter K84]
222147954 12..211    |Agrobacterium vitis S4                        |B|Proteobacteria Alphaproteobacteria | >gi|222147954|ref|YP_002548911.1| Hemolysin III [Agrobacterium vitis S4]
226947154 3..202     |Azotobacter vinelandii DJ                     |B|Proteobacteria Gammaproteobacteria | >gi|226947154|ref|YP_002802227.1| hemolysin III [Azotobacter vinelandii DJ]
226954402 6..205     |Acinetobacter sp. ATCC 27244                  |B|Proteobacteria Gammaproteobacteria | >gi|226954402|ref|ZP_03824866.1| hemolysin III (HLY-III) [Acinetobacter sp. ATCC 27244]
294651415 20..219    |Acinetobacter haemolyticus ATCC 19194         |B|Proteobacteria Gammaproteobacteria | >gi|294651415|ref|ZP_06728729.1| hemolysin III family protein [Acinetobacter haemolyticus ATCC 19194]
227528354 23..222    |Lactobacillus ruminis ATCC 25644              |B|Firmicutes Lactobacillales         | >gi|227528354|ref|ZP_03958403.1| hemolysin III [Lactobacillus ruminis ATCC 25644]
227821323 12..211    |Sinorhizobium fredii NGR234                   |B|Proteobacteria Alphaproteobacteria | >gi|227821323|ref|YP_002825293.1| putative hemolysin-related transmembrane protein [Sinorhizobium fredii NGR234]
227891642 7..206     |Lactobacillus salivarius ATCC 11741           |B|Firmicutes Lactobacillales         | >gi|227891642|ref|ZP_04009447.1| hemolysin III [Lactobacillus salivarius ATCC 11741]
90961389  14..197    |Lactobacillus salivarius UCC118               |B|Firmicutes Lactobacillales         | >gi|90961389|ref|YP_535305.1| hypothetical protein LSL_0412 [Lactobacillus salivarius UCC118]
229593106 3..202     |Pseudomonas fluorescens SBW25                 |B|Proteobacteria Gammaproteobacteria | >gi|229593106|ref|YP_002875225.1| putative hemolysin [Pseudomonas fluorescens SBW25]
239817023 17..216    |Variovorax paradoxus S110                     |B|Proteobacteria Betaproteobacteria  | >gi|239817023|ref|YP_002945933.1| channel protein, hemolysin III family [Variovorax paradoxus S110]
239917048 34..233    |Micrococcus luteus NCTC 2665                  |B|Actinobacteria Actinobacteridae    | >gi|239917048|ref|YP_002956606.1| channel protein, hemolysin III family [Micrococcus luteus NCTC 2665]
289704935 333..532   |Micrococcus luteus SK58                       |B|Actinobacteria Actinobacteridae    | >gi|289704935|ref|ZP_06501352.1| channel protein, hemolysin III family protein [Micrococcus luteus SK58]
242017752 43..242    |Pediculus humanus corporis                    |E|Metazoa Arthropoda                 | >gi|242017752|ref|XP_002429351.1| monocyte to macrophage differentiation protein, putative [Pediculus humanus corporis]
253702577 12..211    |Geobacter sp. M21                             |B|Proteobacteria Deltaproteobacteria | >gi|253702577|ref|YP_003023766.1| channel protein, hemolysin III family [Geobacter sp. M21]
254254761 5..204     |Burkholderia dolosa AUO158                    |B|Proteobacteria Betaproteobacteria  | >gi|254254761|ref|ZP_04948078.1| hypothetical protein BDAG_04078 [Burkholderia dolosa AUO158]
254283849 5..204     |gamma proteobacterium NOR51-B                 |B|Proteobacteria Gammaproteobacteria.| >gi|254283849|ref|ZP_04958817.1| channel protein, hemolysin III family [gamma proteobacterium NOR51-B]
254380614 44..243    |Streptomyces sp. Mg1                          |B|Actinobacteria Actinobacteridae    | >gi|254380614|ref|ZP_04995980.1| integral membrane protein [Streptomyces sp. Mg1]
254524265 8..207     |Stenotrophomonas sp. SKA14                    |B|Proteobacteria Gammaproteobacteria | >gi|254524265|ref|ZP_05136320.1| hemolysin-III family membrane protein [Stenotrophomonas sp. SKA14]
255066579 19..218    |Neisseria sicca ATCC 29256                    |B|Proteobacteria Betaproteobacteria  | >gi|255066579|ref|ZP_05318434.1| hemolysin III [Neisseria sicca ATCC 29256]
255319629 11..210    |Acinetobacter radioresistens SK82             |B|Proteobacteria Gammaproteobacteria | >gi|255319629|ref|ZP_05360839.1| hemolysin-3 [Acinetobacter radioresistens SK82]
256833107 69..268    |Jonesia denitrificans DSM 20603               |B|Actinobacteria Actinobacteridae    | >gi|256833107|ref|YP_003161834.1| Hly-III family protein [Jonesia denitrificans DSM 20603]
257468404 5..204     |Fusobacterium ulcerans ATCC 49185             |B|Fusobacteria Fusobacteriales       | >gi|257468404|ref|ZP_05632498.1| hemolysin III family channel protein [Fusobacterium ulcerans ATCC 49185]
257870141 11..210    |Enterococcus gallinarum EG2                   |B|Firmicutes Lactobacillales         | >gi|257870141|ref|ZP_05649794.1| hemolysin III [Enterococcus gallinarum EG2]
260577204 12..211    |Rhodobacter sp. SW2                           |B|Proteobacteria Alphaproteobacteria | >gi|260577204|ref|ZP_05845180.1| Hly-III family protein [Rhodobacter sp. SW2]
261364055 3..202     |Neisseria mucosa ATCC 25996                   |B|Proteobacteria Betaproteobacteria  | >gi|261364055|ref|ZP_05976938.1| hemolysin III [Neisseria mucosa ATCC 25996]
262377484 16..215    |Acinetobacter lwoffii SH145                   |B|Proteobacteria Gammaproteobacteria | >gi|262377484|ref|ZP_06070706.1| hemolysin-3 [Acinetobacter lwoffii SH145]
269219221 32..231    |Actinomyces sp. oral taxon 848 str. F0332     |B|Actinobacteria Actinobacteridae    | >gi|269219221|ref|ZP_06163075.1| channel protein, hemolysin III family [Actinomyces sp. oral taxon 848 str. F0332]
27382363  27..226    |Bradyrhizobium japonicum USDA 110             |B|Proteobacteria Alphaproteobacteria | >gi|27382363|ref|NP_773892.1| hypothetical protein bll7252 [Bradyrhizobium japonicum USDA 110]
283842457 27..226    |Rhodopseudomonas palustris DX-1               |B|Proteobacteria Alphaproteobacteria | >gi|283842457|ref|ZP_06359994.1| Hly-III family protein [Rhodopseudomonas palustris DX-1]
39934655  27..226    |Rhodopseudomonas palustris CGA009             |B|Proteobacteria Alphaproteobacteria | >gi|39934655|ref|NP_946931.1| hemolysin III [Rhodopseudomonas palustris CGA009]
291279539 7..206     |Deferribacter desulfuricans SSM1              |B|Deferribacteres Deferribacterales  | >gi|291279539|ref|YP_003496374.1| channel protein, hemolysin III family [Deferribacter desulfuricans SSM1]
291297969 12..211    |Stackebrandtia nassauensis DSM 44728          |B|Actinobacteria Actinobacteridae    | >gi|291297969|ref|YP_003509247.1| Hly-III family protein [Stackebrandtia nassauensis DSM 44728]
292492830 11..210    |Nitrosococcus halophilus Nc4                  |B|Proteobacteria Gammaproteobacteria | >gi|292492830|ref|YP_003528269.1| Hly-III family protein [Nitrosococcus halophilus Nc4]
294507227 32..231    |Salinibacter ruber M8                         |B|Bacteroidetes Sphingobacteria      | >gi|294507227|ref|YP_003571285.1| Conserved hypothetical protein, membrane [Salinibacter ruber M8]
83816485  29..228    |Salinibacter ruber DSM 13855                  |B|Bacteroidetes Sphingobacteria      | >gi|83816485|ref|YP_445346.1| hemolysin, [Salinibacter ruber DSM 13855]
296116113 14..213    |Gluconacetobacter hansenii ATCC 23769         |B|Proteobacteria Alphaproteobacteria | >gi|296116113|ref|ZP_06834731.1| hemolysin III protein [Gluconacetobacter hansenii ATCC 23769]
297567021 4..203     |Meiothermus silvanus DSM 9946                 |B|Deinococcus-Thermus Deinococci     | >gi|297567021|ref|YP_003685993.1| hemolysin III family channel protein [Meiothermus silvanus DSM 9946]
297626869 43..242    |Propionibacterium freudenreichii subsp. shermanii CIRM-BIA1|B|Actinobacteria Actinobacteridae    | >gi|297626869|ref|YP_003688632.1| hypothetical protein PFREUD_17000 [Propionibacterium freudenreichii subsp. shermanii CIRM-BIA1]
299132087 27..226    |Afipia sp. 1NLS2                              |B|Proteobacteria Alphaproteobacteria | >gi|299132087|ref|ZP_07025282.1| Hly-III family protein [Afipia sp. 1NLS2]
42522243  13..212    |Bdellovibrio bacteriovorus HD100              |B|Proteobacteria Deltaproteobacteria | >gi|42522243|ref|NP_967623.1| hemolysin III [Bdellovibrio bacteriovorus HD100]
42525120  7..206     |Bdellovibrio bacteriovorus HD100              |B|Proteobacteria Deltaproteobacteria | >gi|42525120|ref|NP_970500.1| hemolysin III [Bdellovibrio bacteriovorus HD100]
50955272  59..258    |Leifsonia xyli subsp. xyli str. CTCB07        |B|Actinobacteria Actinobacteridae    | >gi|50955272|ref|YP_062560.1| hemolysin III family protein [Leifsonia xyli subsp. xyli str. CTCB07]
70733095  3..202     |Pseudomonas fluorescens Pf-5                  |B|Proteobacteria Gammaproteobacteria | >gi|70733095|ref|YP_262868.1| hemolysin III [Pseudomonas fluorescens Pf-5]
71281079  18..217    |Colwellia psychrerythraea 34H                 |B|Proteobacteria Gammaproteobacteria | >gi|71281079|ref|YP_267470.1| hemolysin III [Colwellia psychrerythraea 34H]
77461513  3..202     |Pseudomonas fluorescens Pf0-1                 |B|Proteobacteria Gammaproteobacteria | >gi|77461513|ref|YP_351020.1| HylII [Pseudomonas fluorescens Pf0-1]
83592000  10..209    |Rhodospirillum rubrum ATCC 11170              |B|Proteobacteria Alphaproteobacteria | >gi|83592000|ref|YP_425752.1| Hly-III-like protein [Rhodospirillum rubrum ATCC 11170]
86751048  27..226    |Rhodopseudomonas palustris HaA2               |B|Proteobacteria Alphaproteobacteria | >gi|86751048|ref|YP_487544.1| Hly-III related proteins [Rhodopseudomonas palustris HaA2]
91978164  27..226    |Rhodopseudomonas palustris BisB5              |B|Proteobacteria Alphaproteobacteria | >gi|91978164|ref|YP_570823.1| Hly-III related proteins [Rhodopseudomonas palustris BisB5]
88856092  42..241    |marine actinobacterium PHSC20C1               |B|Actinobacteria.                    | >gi|88856092|ref|ZP_01130753.1| hemolysin III family protein [marine actinobacterium PHSC20C1]
89902628  5..204     |Rhodoferax ferrireducens T118                 |B|Proteobacteria Betaproteobacteria  | >gi|89902628|ref|YP_525099.1| hemolysin III family channel protein [Rhodoferax ferrireducens T118]
90425742  32..231    |Rhodopseudomonas palustris BisB18             |B|Proteobacteria Alphaproteobacteria | >gi|90425742|ref|YP_534112.1| Hly-III related proteins [Rhodopseudomonas palustris BisB18]
91790555  3..202     |Polaromonas sp. JS666                         |B|Proteobacteria Betaproteobacteria  | >gi|91790555|ref|YP_551507.1| hemolysin III family channel protein [Polaromonas sp. JS666]
116491855 12..210    |Pediococcus pentosaceus ATCC 25745            |B|Firmicutes Lactobacillales         | >gi|116491855|ref|YP_803590.1| hemolysin III-like protein [Pediococcus pentosaceus ATCC 25745]
118097813 34..232    |Gallus gallus                                 |E|Metazoa Chordata                   | >gi|118097813|ref|XP_414787.2| PREDICTED: similar to Monocyte to macrophage differentiation-associated 2 [Gallus gallus]
119473335 8..206     |Alteromonadales bacterium TW-7                |B|Proteobacteria Gammaproteobacteria | >gi|119473335|ref|ZP_01614944.1| putative hemolysin III [Alteromonadales bacterium TW-7]
119774997 37..235    |Shewanella amazonensis SB2B                   |B|Proteobacteria Gammaproteobacteria | >gi|119774997|ref|YP_927737.1| hemolysin III family channel protein [Shewanella amazonensis SB2B]
124265724 12..210    |Methylibium petroleiphilum PM1                |B|Proteobacteria Betaproteobacteria  | >gi|124265724|ref|YP_001019728.1| hemolysin III [Methylibium petroleiphilum PM1]
126334538 57..255    |Monodelphis domestica                         |E|Metazoa Chordata                   | >gi|126334538|ref|XP_001368371.1| PREDICTED: monocyte to macrophage differentiation factor 2-like [Monodelphis domestica]
154759275 34..232    |Homo sapiens                                  |E|Metazoa Chordata                   | >gi|154759275|ref|NP_940685.3| monocyte to macrophage differentiation factor 2 isoform 2 [Homo sapiens]
146277309 12..210    |Rhodobacter sphaeroides ATCC 17025            |B|Proteobacteria Alphaproteobacteria | >gi|146277309|ref|YP_001167468.1| Hly-III family protein [Rhodobacter sphaeroides ATCC 17025]
146338516 28..226    |Bradyrhizobium sp. ORS278                     |B|Proteobacteria Alphaproteobacteria | >gi|146338516|ref|YP_001203564.1| hemolysin III [Bradyrhizobium sp. ORS278]
148257892 28..226    |Bradyrhizobium sp. BTAi1                      |B|Proteobacteria Alphaproteobacteria | >gi|148257892|ref|YP_001242477.1| hemolysin III [Bradyrhizobium sp. BTAi1]
148550062 5..203     |Pseudomonas putida F1                         |B|Proteobacteria Gammaproteobacteria | >gi|148550062|ref|YP_001270164.1| hemolysin III family channel protein [Pseudomonas putida F1]
167036025 5..203     |Pseudomonas putida GB-1                       |B|Proteobacteria Gammaproteobacteria | >gi|167036025|ref|YP_001671256.1| hemolysin III family channel protein [Pseudomonas putida GB-1]
26991663  5..203     |Pseudomonas putida KT2440                     |B|Proteobacteria Gammaproteobacteria | >gi|26991663|ref|NP_747088.1| hemolysin III family channel protein [Pseudomonas putida KT2440]
148559130 15..213    |Brucella ovis ATCC 25840                      |B|Proteobacteria Alphaproteobacteria | >gi|148559130|ref|YP_001258113.1| hemolysin III family channel protein [Brucella ovis ATCC 25840]
153007423 15..213    |Ochrobactrum anthropi ATCC 49188              |B|Proteobacteria Alphaproteobacteria | >gi|153007423|ref|YP_001368638.1| Hly-III family protein [Ochrobactrum anthropi ATCC 49188]
17988160  15..213    |Brucella melitensis bv. 1 str. 16M            |B|Proteobacteria Alphaproteobacteria | >gi|17988160|ref|NP_540794.1| hemolysin III [Brucella melitensis bv. 1 str. 16M]
254718287 15..213    |Brucella sp. 83/13                            |B|Proteobacteria Alphaproteobacteria | >gi|254718287|ref|ZP_05180098.1| Hemolysin-3 [Brucella sp. 83/13]
294851473 15..213    |Brucella sp. NVSL 07-0026                     |B|Proteobacteria Alphaproteobacteria | >gi|294851473|ref|ZP_06792146.1| hemolysin III [Brucella sp. NVSL 07-0026]
149201214 10..208    |Roseovarius sp. TM1035                        |B|Proteobacteria Alphaproteobacteria | >gi|149201214|ref|ZP_01878189.1| hypothetical protein RTM1035_16352 [Roseovarius sp. TM1035]
149915896 10..208    |Roseobacter sp. AzwK-3b                       |B|Proteobacteria Alphaproteobacteria | >gi|149915896|ref|ZP_01904420.1| hypothetical protein RAZWK3B_07449 [Roseobacter sp. AzwK-3b]
156084830 38..236    |Babesia bovis T2Bo                            |E|Alveolata Apicomplexa              | >gi|156084830|ref|XP_001609898.1| hypothetical protein [Babesia bovis T2Bo]
157962206 48..246    |Shewanella pealeana ATCC 700345               |B|Proteobacteria Gammaproteobacteria | >gi|157962206|ref|YP_001502240.1| hemolysin III family channel protein [Shewanella pealeana ATCC 700345]
159184527 13..211    |Agrobacterium tumefaciens str. C58            |B|Proteobacteria Alphaproteobacteria | >gi|159184527|ref|NP_353925.2| hemolysin III [Agrobacterium tumefaciens str. C58]
166712870 12..210    |Xanthomonas oryzae pv. oryzicola BLS256       |B|Proteobacteria Gammaproteobacteria | >gi|166712870|ref|ZP_02244077.1| hemolysin III [Xanthomonas oryzae pv. oryzicola BLS256]
21243770  12..210    |Xanthomonas axonopodis pv. citri str. 306     |B|Proteobacteria Gammaproteobacteria | >gi|21243770|ref|NP_643352.1| hemolysin III [Xanthomonas axonopodis pv. citri str. 306]
58581437  12..210    |Xanthomonas oryzae pv. oryzae KACC10331       |B|Proteobacteria Gammaproteobacteria | >gi|58581437|ref|YP_200453.1| hemolysin III [Xanthomonas oryzae pv. oryzae KACC10331]
167839379 5..203     |Burkholderia thailandensis MSMB43             |B|Proteobacteria Betaproteobacteria  | >gi|167839379|ref|ZP_02466063.1| hemolysin [Burkholderia thailandensis MSMB43]
53717464  5..203     |Burkholderia mallei ATCC 23344                |B|Proteobacteria Betaproteobacteria  | >gi|53717464|ref|YP_105412.1| hemolysin III [Burkholderia mallei ATCC 23344]
53721828  10..208    |Burkholderia pseudomallei K96243              |B|Proteobacteria Betaproteobacteria  | >gi|53721828|ref|YP_110813.1| hemolysin [Burkholderia pseudomallei K96243]
83716235  5..203     |Burkholderia thailandensis E264               |B|Proteobacteria Betaproteobacteria  | >gi|83716235|ref|YP_439792.1| hemolysin III [Burkholderia thailandensis E264]
170693411 5..203     |Burkholderia graminis C4D1M                   |B|Proteobacteria Betaproteobacteria  | >gi|170693411|ref|ZP_02884570.1| channel protein, hemolysin III family [Burkholderia graminis C4D1M]
187925283 5..203     |Burkholderia phytofirmans PsJN                |B|Proteobacteria Betaproteobacteria  | >gi|187925283|ref|YP_001896925.1| channel protein, hemolysin III family [Burkholderia phytofirmans PsJN]
282885223 5..203     |Burkholderia sp. CCGE1001                     |B|Proteobacteria Betaproteobacteria  | >gi|282885223|ref|ZP_06293810.1| channel protein, hemolysin III family [Burkholderia sp. CCGE1001]
289632935 5..203     |Burkholderia sp. CCGE1003                     |B|Proteobacteria Betaproteobacteria  | >gi|289632935|ref|ZP_06465225.1| putative hemolysin HylII [Burkholderia sp. CCGE1003]
182436350 27..225    |Streptomyces griseus subsp. griseus NBRC 13350|B|Actinobacteria Actinobacteridae    | >gi|182436350|ref|YP_001824069.1| hypothetical protein SGR_2557 [Streptomyces griseus subsp. griseus NBRC 13350]
239943863 24..222    |Streptomyces roseosporus NRRL 15998           |B|Actinobacteria Actinobacteridae    | >gi|239943863|ref|ZP_04695800.1| hypothetical protein SrosN15_22891 [Streptomyces roseosporus NRRL 15998]
291447327 27..225    |Streptomyces roseosporus NRRL 15998           |B|Actinobacteria Actinobacteridae    | >gi|291447327|ref|ZP_06586717.1| integral membrane protein [Streptomyces roseosporus NRRL 15998]
184201412 34..232    |Kocuria rhizophila DC2201                     |B|Actinobacteria Actinobacteridae    | >gi|184201412|ref|YP_001855619.1| hypothetical protein KRH_17660 [Kocuria rhizophila DC2201]
186475196 5..203     |Burkholderia phymatum STM815                  |B|Proteobacteria Betaproteobacteria  | >gi|186475196|ref|YP_001856666.1| hemolysin III family channel protein [Burkholderia phymatum STM815]
189239906 45..243    |Tribolium castaneum                           |E|Metazoa Arthropoda                 | >gi|189239906|ref|XP_970806.2| PREDICTED: similar to monocyte to macrophage differentiation protein [Tribolium castaneum]
[truncated: 176,249 more chars]
