# Supplementary material for: CREST - a large and diverse superfamily of putative transmembrane hydrolases
Source: Biol Direct. 2011 Jul 6;6:37. doi: 10.1186/1745-6150-6-37 (PMC3146951; doi:10.1186/1745-6150-6-37)
Supplement: Additional file 2 — A multiple sequence alignment of CREST domains. This file contains the alignment of CREST domains covering seven predicted core transmembrane segments for sequences shown in Figure 1. [file 1745-6150-6-37-S2.PDF]

Additional data file 2

A multiple sequence alignment of CREST domains for the sequences shown in Figure 1. This alignment covers seven predicted core transmembrane segments (TM1–TM7) marked below it. These sequences are denoted by their gene identification numbers followed by their accession numbers. They are in the same order as in Figure 1. Species abbreviations are shown after sequence names. Starting and ending residue numbers are shown before and after the sequences, respectively. Sequence lengths are shown in brackets. Putative active site residues are shaded in cyan (mutations in them in grey). A conserved position with mainly small residues (mostly G, A and S) in TM6 is shaded in green (the only two large residues in them shaded in grey). Sequence conservation values (scaled from 0 to 9) calculated by the AL2CO program are shown above the alignment (conservation values above 4 are highlighted in red). Species abbreviations are as follows: At, *Arabidopsis thaliana*; Bs, *Bacillus subtilis*; Ca, *Clostridium acetobutylicum*; Ce, *Caenorhabditis elegans*; Cr, *Chlamydomonas reinhardtii*; Cs, *Cyanothece* sp.; Dd, *Dictyostelium discoideum*; Dm, *Drosophila melanogaster*; Fs, *Frankia* sp.; Hs, *Homo sapiens*; Mp, *Micromonas* sp.; Mr, *Methylobacterium radiotolerans*; Mt, *Mycobacterium tuberculosis*; Ol, *Ostreococcus lucimarinus*; Pp, *Photobacterium profundum*; Ps, *Pseudovibrio* sp.; Pt, *Paramecium tetraurelia*; Re, *Ralstonia eutropha*; Sc, *Saccharomyces cerevisiae*; Tb, *Trypanosoma brucei*; Tc, *Tribolium castaneum*.

Sequence	Species	Start	Alignment (TM1–TM2 region)	Alignment (middle)	Alignment (TM7 region)	End	Length
Conservation:			12123325532133121012211100100110000000000000000000000100011012212222132282249211000000000000000000000000	[illegible]	0000000000000000202110923693322221211110120		
19424128_NP_597999.1	Hs	26	AEFYNTFSNIPFFIFGPLMMLLMHPYAQK------------------------RSRYIYVVWVLFMIIGLFSMYFHMTLSF	[illegible]	HFFYLHSIWHVLISITFPYGMVTMA	225	[264]
71043498_NP_001010887.2	Hs	31	AEFYNTISNVLFFILPPICMCLFRQYATC------------------------FNSGIYLIWTLLVVVGIGSVYFHATLSF	[illegible]	NFPYLHCMWHILICLAAYLGCVCFA	230	[275]
170932467_NP_060837.3	Hs	32	AEFWNTVSNLIMIIPPMFGAVQSVRDGL------------------------EKRYIASYLALTVVGMGSWCFHMTLKY	[illegible]	IIGITTQFHAWWHILTGLGSYLHILFSL	236	[267]
19921574_NP_610020.1	Dm	41	AEFVNTFSNFLFILLPPVLIMLFKEYGRF------------------------VTPGIHVIWVLLIVVGLSSMYFHATLSL	[illegible]	NFPYLHGFWHIFIFIAAYTVLVLFA	240	[283]
6325170_NP_015238.1	Sc	37	AEWSNTITNSIFLMTAFYSTYSAWRNKL------------------------ETRYILIGMGFSLVGIGSWLFHMTLQY	[illegible]	PLGVLLELHAWWHLLTGTGVYIFVVYLQ	260	[317]
6319660_NP_009742.1	Sc	38	AEWSNTLTNSVFILSAIYTTYSAYKNKL------------------------EKRFLLIGFGYGLVGVGSWLFHMTLKY	[illegible]	PLGVLLEPHGWWHILTGMGIYFYIVSLE	260	[316]
18415901_NP_567660.1	At	30	AEFYNTISNVPGILLALIGLVNALRQRF------------------------EKRFSILHISNMILAIGSMLYHATLQH	[illegible]	WPVNPQGHALWHVFMSFNSYCANTFLM	224	[255]
21361519_NP_057083.2	Hs	133	TETGNIWTHLLGFVLFLFLGILTMLRPNMYFMA-------------------PLQEKVVFGMFFLGAVLCLSFSWLFHTVYCHS	[illegible]	GKFDIWFQSHQIFHVLVVAAAFVHFYGVS	356	[375]
38261973_NP_078827.2	Hs	144	TETGNIWTHLLGCVFFLCLGIFYMFRPNISFVA-------------------PLQEKVVFGLFFLGAILCLSFSWLFHTVYCHS	[illegible]	GKCDIWFHSHQLFHIFVVAGAFVHFHGVS	367	[386]
30410020_NP_848509.1	Hs	70	NEAVNVWTHLLAALVLLLRLALFVETVDFWG-------------------DPHALPLFIIVLASFTYLSFSALAHLLQAKS	[illegible]	GSCHVFGQGHQLFHIFLVLCTLAQLEAVA	294	[346]
94158915_NP_001035292.1	Hs	68	NETVNIWSHLLGFFLFFTLGIYDMTSVLPSASA-------------------SREDFVICSICLFCFQVCMLCSVGYHLFSCHRS	[illegible]	GQLNYLGSSHQIWHILAVVMLYWWHQSTV	293	[311]
38348324_NP_940906.1	Hs	82	NETLNFWTHFIPLLLFLSKFCRLFFLSGGDVPF-------------------HHPWLLPLWCYASGVLLTFAMSCTAHVFSCLS	[illegible]	GLFDIIGHSHQLFHIFTFLSIYDQVYYVE	330	[377]
24649047_NP_651061.1	Dm	202	TETGNIWTHLLGCIAFIGVALYFISRPSVEIQ-------------------TQEKIVFGAFFIGAIVCLGFSFAFHTLSCHS	[illegible]	GKFDIWGQSHQIFHILVIAAAFVHYHGIS	423	[444]
6320699_NP_010780.1	Sc	79	NETVNIYTHLVPAIVYFVFAITLTNYFLIPVFPS-----------------TSWSDYTVINIFLMGAFSCLMCSSCFHCMKQHS	[illegible]	KFDFFGSSHQIFHIMVVLGSVCHLKAII	304	[316]
27808716_NP_014641.2	Sc	78	NESVNIYSHLIPALGFFTVLLLDKSTIKVFATT-------------------TWLDHMVIDLFYSGAFACLILSSSFHCLKSHS	[illegible]	KFDIWGHSHQLFHFLVVIAALCHLRGLL	301	[317]
6323051_NP_013123.1	Sc	256	NETSNIWSHLLGAIYIIYLAIYDFPQSEVWRNSQ-----------------VPPQARWIVFMFLAAALKCMLSSVFWHTFNGTSF	[illegible]	SCPSHAKSFKSLWWVDYFGCSHTFWHFFVVLGVIGHYRAIL	533	[543]
6324471_NP_014540.1	Sc	62	NESLVALIYLLGSMLSFCLLIFFTDFYLIPLFPT-----------------TTTMTDYIVFNFYLLNVFVFCMVHFIYHFVKNIS	[illegible]	KEEGWNHSEYLFHLLISGTAFYHFFILI	292	[312]
15241302_NP_197527.1	At	94	NESLNVWTHLIGFIFFVALTVANIIHHDGFFPVDA---------------KSPGNVTRWPFFVFLGGSMFCLLASSICHLFCCHS	[illegible]	GWFDRVGHSHQIFHVFVLLGALSHYAAAL	321	[332]
52630445_NP_036461.2	Hs	27	EHAANCYTHAFLIVPAIVGSALLHRLSDDC---------------------WEKITAWIYGMGLCALFIVSTVFHIVSWKK	[illegible]	IPFAHAIWHLFVATAAAVHYYAIW	224	[238]
24640324_NP_572382.1	Dm	85	EQVANVITHGIWILPAVFAAIKLFERSSSA---------------------SQYLVAWVYGGALCMLFTVSTFFHCSCYCAEHKPPKNVKAWPCLGWQTYQGL	[illegible]	IPMAHAIWHLFVVLAAGCHYYAIL	301	[313]
16079238_NP_390062.1	Bs	6	EEIANAITHGIGVLLSIPALVFLIIFAANYGS-------------------AWDIVSFTIFGVSMLLLYLSSTLLHSITHK	[illegible]	IPFHHAIWHSFVLGGSAAMFFCVL	203	[213]
15894169_NP_347518.1	Ca	6	KDPVSGITHLVGAVLSIIALVCMVHHSIVADS-------------------KISIAASIIFGVSLIFLYSASASYHLINVS	[illegible]	AKYFGFHEIFHLFVMAGSLFHFIMIY	210	[214]
15608225_NP_215601.1	Mt	36	FRGWIHVYSAGTAVLAGASLVAVSWAVGSA---------------------KAGLTTLAYTAATITMFTVSATYHRVNWKS	[illegible]	PTTFGYHEFFHACTAVAAICHYIAMW	238	[242]
45505180_NP_219487.3	Hs	103	QEPASAVASFLNGLASLVMLCRYRTFVPASS--------------------PMYHTCVAFAWVSLNAWFWSTVFHTRDTD	[illegible]	FWVLDAHAIWHISTIPVHVLFFSFLE	303	[320]
19921698_NP_610223.1	Dm	110	QEPASVIFSCLNFVVHLRLLRKFRREVRPDS--------------------PCYMLTHIFAVTSLNGWIWSAIFHTRDFP	[illegible]	LWILDAHALWHLATIPLASLYYDFMI	310	[330]
6319892_NP_009973.1	Sc	114	QEFFSTIFSIGNFIPHYKGFVKFSRIIREEGDRRR------------KNSRSILIWNYLYVTVAGMLAWTASSVFHCRDLI	[illegible]	EWQIDAHALWHLCTIWPSWVLYDFFL	345	[357]
15219283_NP_173104.1	At	115	QEPASVAFSVLNLAMHFHGWLSFFIMIYYKLPLKQD-----------RTAYYEYVGLWHIYGLLSMNSWFWSAVFHSRDVD	[illegible]	EGYFDAHSIWHAATIPLTILWWSFIR	323	[342]
18424554_NP_568951.1	At	120	QEPVSVAFSALDLAMQFQGWVSYFILVYYKLPLQPN-----------RKTYYEYNGIVHIYAIIVMNSLFWSSICHSRDVE	[illegible]	KGYIDAHALWRGAGIPLSYLWWSFVC	328	[343]
116812584_NP_060169.2	Hs	493	SAFNNILSNLGHVLLGFLFLLIVLRRDILHRRALEAKDI-----FAVEYGIPKHFGLFYAMGIALMMEGVLSACYHVCPNY	[illegible]	LDFFDDHDIWHFLSATALFFSFLVLL	810	[827]
94721340_NP_001035545.1	Hs	498	SAFNNILSNLGYILLGLLFLLIILQREINHNRALLRNDL-----CALECGIPKHFGLFYAMGTALMMEGLLSACYHVCPNY	[illegible]	LDFFDDHDIWHFLSSIAMFGSFLVLL	815	[832]
157694508_NP_001099012.1	Tc	434	SDFNHIFSNVGYIIVGILFLGVVLHRQTKIPNSS--------------TGIPVHYGVYYAMGIALIIEGILSACYHICPSQ	[illegible]	MSFYDKHDVWHLLSAPALYLTFMFLL	740	[757]
17570717_NP_509489.1	Ce	423	VAFNNVVSNGGYVYFGTLIIVMNYCRERSFRRLFAVQPT-----LAERYGLPQHSGLMTAIGLAVIMEGISSATYHVCPNN	[illegible]	LNFYDNHDLWHLSSAFAIFFSFTAIN	739	[756]
166240207_XP_001733041.1	Dd	107	PSFNNVLSNLFYLIAGTSFILITHFTSHQEDGIH---------------GLHTDLSLYYSLGLTIILEGIFSALYHVCPSR	[illegible]	FNYWDTHDLWHFLSAIGLFSIMAIVY	415	[432]
25154290_NP_504372.2	Ce	478	RAWNNIITNIGYTLYGAIFIVLSICRRGRHEYSHV-------------FGTYECTLLDVTIGVFMVLQSIASATYHICPSD	[illegible]	LGFFGSHDLWHIFGALAGLFTFIFVS	759	[776]
133965429_NP_510761.2	Ce	399	KSFNSMTSASSLAVIGVLNLIIVFREKIFSFKIPRLP---------TTHGLQQRDAPKVVCFLGLVSMGILWTITNNCPHR	[illegible]	LPGIDLNDLRHYSCAVDCFLFICLMD	722	[739]
157676334_NP_067082.2	Hs	547	ATLLLTLSNLMFLAPIAVSVR------------------------------RFFLVEASVYAYTMFFSTFYHACDQPGE--------------AVLCILS	[illegible]	YYYTHSIWHILLAGSAALLLPPPD	741	[771]
110556642_NP_001036054.1	Hs	235	STLLLCLSNLMFLPPVVLAIR------------------------------SRYVLEAAVYTFTMFFSTFYHACDQPGI--------------VVFCIMD	[illegible]	YFYIHSIWHMLIAGSVGFLLPPRA	429	[472]
122937402_NP_001073952.1	Hs	7	KLLLPTLSSLAFLPTVSIAAK------------------------------RRFHMEAMVYLFTLFFVALHHACNGPGLSVLC--------------FMR	[illegible]	DYTYVHSFYHCALAMSFVLLLPKVN	199	[221]
116008425_NP_651350.2	Dm	793	ATLLLTLSNLLFFPSIYMAVK------------------------------RRYYTEGVIYFFAMFFSIFYHACDAGEDE-------------YSFCLVK	[illegible]	YHIVHSIWHMVMALSILCLLPSRK	987	[993]
30690202_NP_850444.1	At	564	QSIALIASNAAALLPAYWALR------------------------------QREYPEWVLFTSSGISSALYHACDVGTWC---------------VLS	[illegible]	SYWMWHSIWHFTIYTSSFFFLCSKI	780	[807]
15223970_NP_177275.1	At	45	PNFLNVISNFPFLIIGLIGLILCFYPEDYFSFS-----------------LRGEKIGWTCFYIGVAAVAFGSSYYHLHPND	[illegible]	HHIISGHSLKHLCAAMVPVFLTLMLA	244	[281]
15239801_NP_196748.1	At	56	PNTLNVMTNFPFLIVGVLGFVLCIGGSFFNIS------------------LNGEIWGWTLFYAGIASLAFGSAFYHLKPDD	[illegible]	RYIISGHSLEHLCSAVATLLLTIMLL	254	[262]
113867303_YP_725792.1	Re	52	PHCLDIGSNLLFLLAGAAGLRYLGTTAASRAFI-----------------APAEAWPFRLLFLAVFLVGLGSAYYHLAPDN	[illegible]	GLVSGHTLKHVFAALAVYGVLLRLK	255	[264]
54302125_YP_132118.1	Pp	50	PNFWNVISNLPFLYVGLKGLQLFRKRSVAIE-------------------PNIALQYPFFFFALMLAFFASSYYHLTPND	[illegible]	GGMSGHSIKHVIAAMGAYFIYIGFR	251	[264]
170749327_YP_001755587.1	Mr	20	AEPANALSNAAFLLAAAAAARRAAAADPP-----------------------DRTCLGLAALIAAVGVGSFLFHTLAVY	[illegible]	LPTGTHPLWHVLNAVVLYALVATAI	221	[228]
254471798_ZP_05085199.1	Ps	20	SEPLNAVSNVAFIIAALFALQIWLNARSNAKNTAS-------------TQTNAQDNAALVLIALLAIIGIGSFLFHTFATL	[illegible]	LPVGTHSLWHILNATVLFILIRAFI	221	[232]
220906069_YP_002481380.1	Cs	18	NEPLNALSNLSFFIAAWAMWRLAGHERKL-----------------------SKSVWIIIGLSGSIGVGSFLFHTFATR	[illegible]	GIGTHFLWHIFNGILLYLSARALI	206	[214]
86741083_YP_481483.1	Fs	1	VRQFDAVSNLAYLTAAFALWRLLRRHSGV-----------------------PASVAFMPPLAVLIGVGSFAFHTLATR	[illegible]	FPIGTHFIWHLLTELLIFVVGRALA	189	[218]
66825793_XP_646251.1	Dd	15	QAFIAFSTNLSVIPVVYHCLK-------------------------------RGSAYEGIIGISCALASMMYHVGEVFENTWWFK-----------LSGMT	[illegible]	DYLRINHGIWHFFIGISFYFIFLSKE	208	[220]
72391880_XP_846234.1	Tb	13	VCILSVVSHATLVPTVYHFLR-------------------------------KRYAYESVLGIFGLTASLMYHICEAFDAKF---------------FMS	[illegible]	HFRMYHSTWHFFCGIASYLMWTLLK	200	[214]
145515449_XP_001443624.1	Pt	20	MVIIITAVSNGVMIPQIIYTYK------------------------------ISNAHTLFLSVLLFVSSFMYHLSESIGVEP--------------IILK	[illegible]	DYLRLWHGLWHLAMNQAYFHLYQLFN	208	[227]
79350419_NP_173724.2	At	81	ETAANVATSLPFIFLGMQAPRK------------------------------NLNTKVYANSLIGVGIASSLYHASRGK----------------------L	[illegible]	TPFLHAGWHLAAAIGVGTCNKLLE	248	[248]
18409704_NP_565005.1	At	77	ETAANVITSIPFIFLGMQAPRK------------------------------NLNMKVYANSLIGVGIASSLYHSSRGK----------------------F	[illegible]	TPFIHAGWHLAAAIGVGTCNKLLQ	244	[244]
159490668_XP_001703295.1	Cr	52	ERCCNVATSTPYIACGCHALKHRKT---------------------------ASGKAWGASVVAAGAASMVFHSSYGS----------------------F	[illegible]	LPLVHSAWHLLSSVSVATLNHLMH	221	[348]
255083963_XP_002508556.1	Ms	69	RGWFHALTAFIFTALIALDAHTLS----------------------------PGPVTWFAVVTFVIPYWASTALHCVPWRRR-------------------TA	[illegible]	WLPLWDGVWSPHENWHCAILVLHCLQLHAVS	288	[312]
145346762_XP_001417852.1	Ol	31	RPLMRGWVHFACVVAALTGRGLGYFKNM------------------------PERASLFVQCTMLTYLGSTCLHMVPWKTA------------------LG	[illegible]	FFEIQ-GVWGPHENWHVIILVVHLLQLKALL	236	[241]
			==========TM1======= ==========TM2=======	==========TM3======= ==========TM4======= ==========TM5======= ==========TM6=======	=========TM7========		
