# Supplementary figures and images for: CREST - a large and diverse superfamily of putative transmembrane hydrolases
Source: Biol Direct. 2011 Jul 6;6:37. doi: 10.1186/1745-6150-6-37 (PMC3146951; doi:10.1186/1745-6150-6-37)

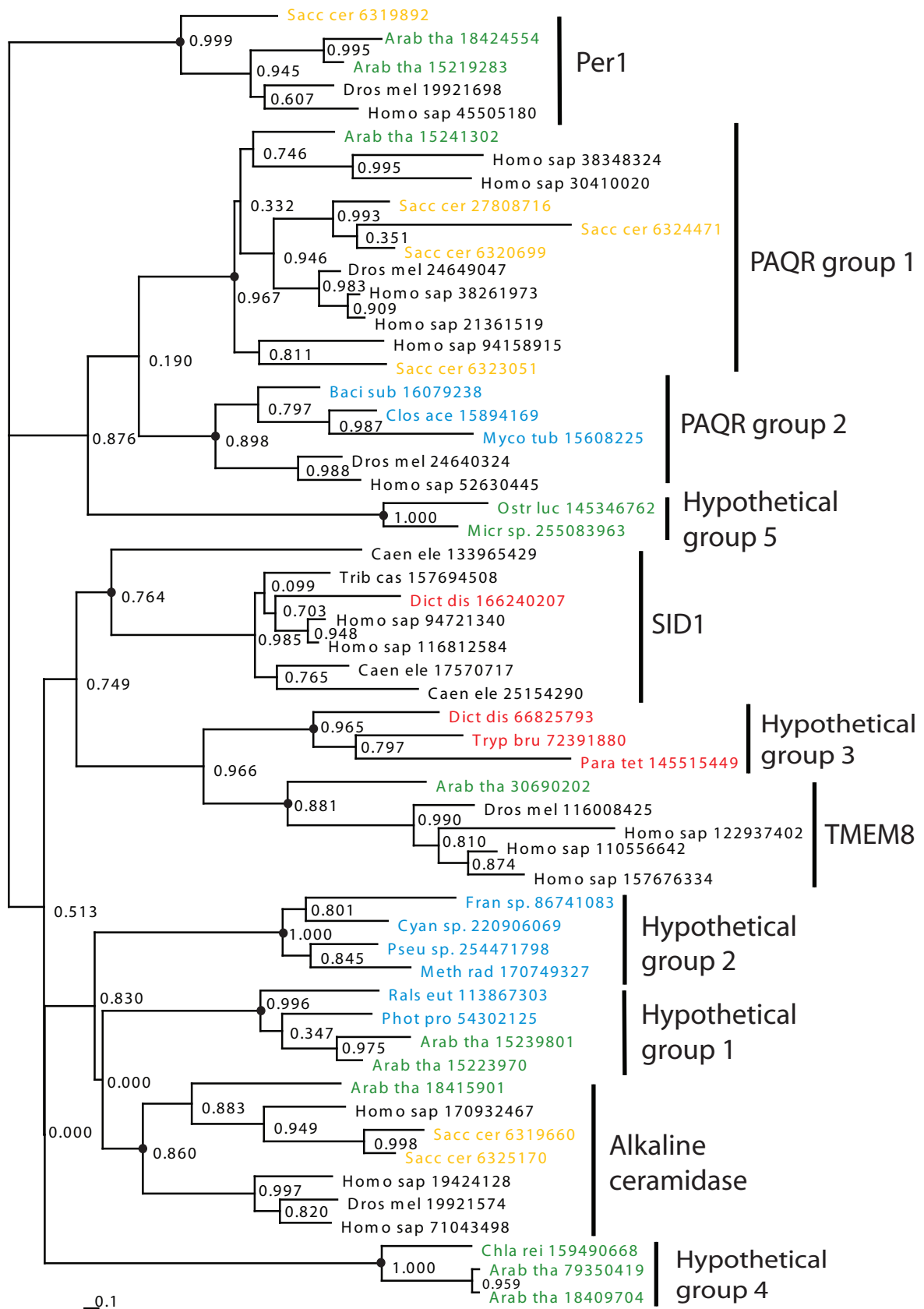

Supplement: Additional file 3 — Phylogenetic reconstruction by PhyML for representative CREST domains. This file contains the phylogenetic tree generated by PhyML (version 3.0) (see Methods) for sequences shown in Figure 1. The eleven CREST groups are labeled to the right of the tree. The root of each group is marked by a black circle. Species abbreviations and coloring schemes are the same as those described in the legend to Figure 3. [file 1745-6150-6-37-S3.PDF]
